# Non-invasive and high-throughput interrogation of exon-specific isoform expression

**DOI:** 10.1038/s41556-021-00678-x

**Published:** 2021-06-03

**Authors:** Dong-Jiunn Jeffery Truong, Teeradon Phlairaharn, Bianca Eßwein, Christoph Gruber, Deniz Tümen, Enikő Baligács, Niklas Armbrust, Francesco Leandro Vaccaro, Eva-Maria Lederer, Eva Magdalena Beck, Julian Geilenkeuser, Simone Göppert, Luisa Krumwiede, Christian Grätz, Gerald Raffl, Dominic Schwarz, Martin Zirngibl, Milica Živanić, Maren Beyer, Johann Dietmar Körner, Tobias Santl, Valentin Evsyukov, Tabea Strauß, Sigrid C. Schwarz, Günter U. Höglinger, Peter Heutink, Sebastian Doll, Marcus Conrad, Florian Giesert, Wolfgang Wurst, Gil Gregor Westmeyer

**Affiliations:** 1 Institute for Synthetic Biomedicine, Helmholtz Zentrum München, Oberschleißheim, Germany; 2 Department of Chemistry and TUM School of Medicine, Technical University of Munich, Munich, Germany; 3 Institute of Developmental Genetics, Helmholtz Zentrum München, Oberschleißheim, Germany; 4 Department of Internal Medicine I, University Hospital Regensburg, Regensburg, Germany; 5 German Center for Neurodegenerative Diseases (DZNE), Munich, Germany; 6 Department of Neurology, Technical University Munich, Munich, Germany; 7 Department of Neurology, Hannover Medical School, Hannover, Germany; 8 Department for Neurodegenerative Diseases, Hertie Institute for Clinical Brain Research, University of Tübingen, Tübingen, Germany; 9 German Center for Neurodegenerative Diseases (DZNE), Tübingen, Germany; 10 Institute of Metabolism and Cell Death, Helmholtz Zentrum München, Oberschleißheim, Germany; 11 Laboratory of Experimental Oncology, National Research Medical University, Moscow, Russia; 12 TUM School of Life Sciences, Technical University of Munich, Freising, Germany

**Keywords:** CRISPR-Cas systems, High-throughput screening, Synthetic biology, RNA splicing, Biological techniques

## Abstract

Expression of exon-specific isoforms from alternatively spliced mRNA is a fundamental mechanism that substantially expands the proteome of a cell. However, conventional methods to assess alternative splicing are either consumptive and work-intensive or do not quantify isoform expression longitudinally at the protein level. Here, we therefore developed an exon-specific isoform expression reporter system (EXSISERS), which non-invasively reports the translation of exon-containing isoforms of endogenous genes by scarlessly excising reporter proteins from the nascent polypeptide chain through highly efficient, intein-mediated protein splicing. We applied EXSISERS to quantify the inclusion of the disease-associated exon 10 in microtubule-associated protein tau (*MAPT*) in patient-derived induced pluripotent stem cells and screened Cas13-based RNA-targeting effectors for isoform specificity. We also coupled cell survival to the inclusion of exon 18b of *FOXP1*, which is involved in maintaining pluripotency of embryonic stem cells, and confirmed that MBNL1 is a dominant factor for exon 18b exclusion. EXSISERS enables non-disruptive and multimodal monitoring of exon-specific isoform expression with high sensitivity and cellular resolution, and empowers high-throughput screening of exon-specific therapeutic interventions.

## Abstract (web summary)

Truong et al. developed a cell-based reporter system, EXSISERS, that enables non-invasive quantification of the protein expression levels of exon-specific isoforms via intein-mediated protein splicing.

## Main

Alternative splicing occurs in >90% of genes, and its impairment is associated with diseases such as spinal muscular atrophy and Parkinson’s disease. Established methods for analysing splicing isoforms measure mRNA by end-point-labelling (quantitative PCR with reverse transcription (RT–qPCR), single-molecule fluorescence in situ hybridization (smFISH)^1^ and RNA sequencing^2^), measure protein using single-timepoint immunochemistry (immunoblot analysis, immunofluorescence staining) or seek to mimic the genetic regulations using minigene analysis^3–5^.

Current protein-level methods for detecting isoform-specific expression are limited by the availability of exon-specific antibodies. In comparison, analyses at the mRNA level can be misleading because post-transcriptional and co-translational regulation does not necessarily change mRNA levels, for example, in cases of translation-arrested^6^, ribosomal-frameshift-regulated^7^ or locally translated mRNA^8,9^. Furthermore, RT–qPCR and RNA-FISH are inherently consumptive, preventing longitudinal analyses of living cells.

In a classical minigene experiment, fragments of the genomic sequence, particularly exon–intron fragments, are copied into a plasmid driven by a constitutive promoter and expressed in a cell line of interest. The minigene’s splice behaviour is then read out by RT–qPCR or an embedded reporter gene. Although this method can efficiently provide valuable insights into alternative splicing, it may not always reflect the physiological processes because partial intron/exon motifs may be overexpressed at unnatural levels, while essential regulatory sequences may be truncated.

For example, the alternatively spliced gene encoding microtubule-associated protein tau (*MAPT*) contains several mutations that are involved in the pathogenesis of Parkinsonian disorders located in introns up to 47 kb away from downstream or upstream exonic sequences^10–12^. Moreover, many vertebrate genes are recursively spliced, which may not be recapitulated by the truncated introns in minigenes^13^. Thus, the length of introns is an important parameter to predict the validity of the splicing behaviour observed in minigenes^14^, in contrast to the exon size, which has had only a small role in experimental settings in vivo^15^. As a consequence, there is a trend towards increasing the size of the constructs by including large genomic fragments with multiple exons and full-length introns to better recapitulate splicing defects^16^. However, these ‘midigenes’ are cumbersome to assemble, usually requiring bacterial artificial chromosomes, and their sizes limit reasonable efficiencies in plasmid transfections.

It has also been suspected that, during plasmid-based overexpression of minigenes, splice factors may be competitively bound away, leading to depletion from endogenous sites and thus unphysiological splicing of collateral genes^17^. Consequently, it is improbable that the regulatory machinery can be faithfully recapitulated outside of the precise three-dimensional genomic architecture at the endogenous sites.

Thus, we developed EXSISERS, which non-invasively quantifies endogenous exon usage at the protein level through a scarless post-translational excision of an exon-resident effector domain using intein-mediated protein splicing.

Here, we demonstrate how different self-excising reporter and effector proteins can be conveniently integrated into an exon of interest (EOI) using CRISPR–Cas9 (ref. ^18^) to enable longitudinal quantification and cellular imaging of exon-specific isoform expression. We also show a high-throughput analysis of exon-specific interventions and enrichment of cells expressing an EOI for rapid identification of splicing regulators.

## Results

### Scarless excision of exon inclusion reporters

**Fig. 1:**
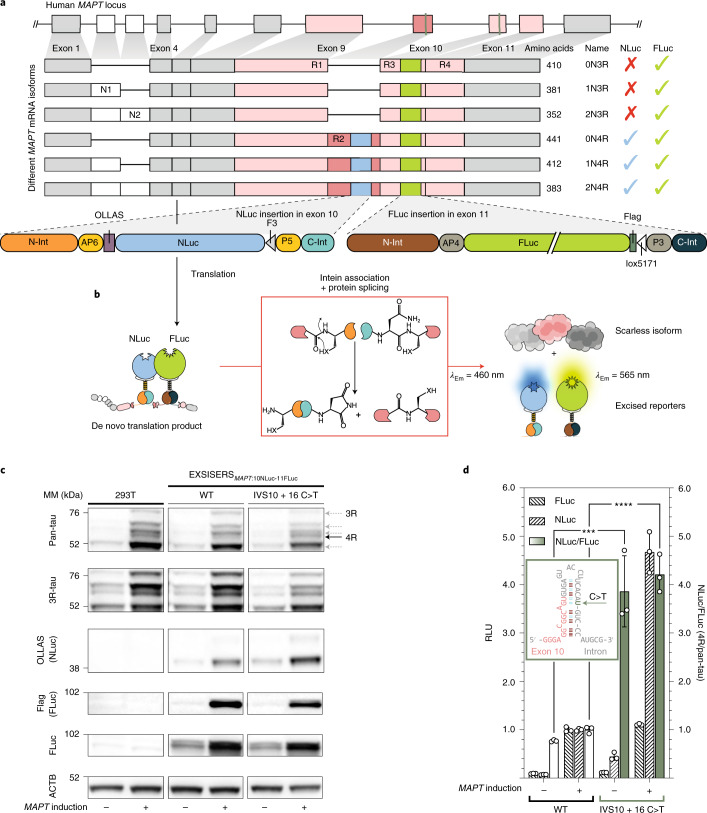
Self-excising reporters for ratiometric readout of exon-10-specific isoforms of human *MAPT*. **a**, Scheme of the genomic organization of the human *MAPT* locus with the EXSISERS reporter NLuc inserted into the alternatively spliced exon 10 and FLuc inserted into the constitutive exon 11, both flanked with CCs and inteins, resulting in the ratiometric reporter system (EXSISERS_*MAPT*:10NLuc-11FLuc_), which detects the fractional expression of 4R referenced against total tau (pan-tau). **b**, Schematic of the post-translational splicing of 4R-tau; the intein-flanked NLuc and FLuc are spliced out post-translationally. NLuc specifically reports 4R-tau, and FLuc reports the presence of pan-tau. The excised luciferases can be read out independently through orthogonal substrates. **c**, Immunoblot analysis of dephosphorylated lysate from unmodified HEK293T cells and EXSISERS_*MAPT*:10NLuc-11FLuc_ clonal cell lines containing either WT *MAPT* or the pathological IVS10+16 C>T mutation confirmed that both FLuc and NLuc were present only after *MAPT* induction. Anti-pan-tau antibodies revealed the pattern of all tau isoforms. The 4R-tau band was identified by excluding the bands that stained positive for 3R-tau. Data represent two independent experiments. MM, molecular mass. **d**, RLU of FLuc and NLuc luciferase was measured from both EXSISERS_*MAPT*:10NLuc-11FLuc_ clones (WT and IVS10+16 C>T) without and with *MAPT* induction and normalized to the average RLU of the reference condition (WT with *MAPT* induction; striped bars, left *y* axis). The ratio of NLuc to FLuc (empty bars, right *y* axis) was then calculated for each sample (striped bars, left *y* axis). Data are mean ± s.d., *n* = 3 biological replicates. Selected results of Bonferroni MCT after two-way ANOVA are indicated by asterisks; ***P < 0.001, ****P < 0.0001 (full statistical results are provided in Supplementary Table 1). Source data are available online. Source data

The core concept of EXSISERS is that, after inclusion of an EOI, a reporter or effector of choice is co-translated and rapidly released by an efficient split–intein^19,20^ protein splicing event resulting in an unmodified protein isoform and thereby preserving the original isoform ratios (Fig. 1a,b).

To demonstrate non-invasive monitoring of an EOI within the natural genomic context and without changing the natural gene expression products, we first generated dual-luciferase EXSISERS lines for ratiometric monitoring of *MAPT* exon 10 (Fig. 1a and Supplementary Figs. 1 and 2).

*MAPT* is primarily expressed in neurons and mediates microtubule polymerization and stabilisation^21^. In the adult human central nervous system, tau is expressed in six isoforms produced by alternative splicing of *MAPT* exons 2, 3 and 10 (Fig. 1a). Depending on the exclusion or inclusion of exon 10, tau isoforms contain three (3R-tau) or four (4R-tau) tandem repeats of a microtubule-binding motif^22^. An abundance of 4R-tau isoforms is implicated in a group of neurodegenerative diseases termed 4R-tauopathies^23^.

Thus, strategies to specifically reduce 4R-tau isoforms are investigated as therapeutic approaches. The development of such interventions could be facilitated using high-throughput screening systems if appropriate read-outs to monitor total tau and 4R-tau expressions were available.

### Ratiometric quantification of tau isoform expression

**Extended Data Fig. 1:**
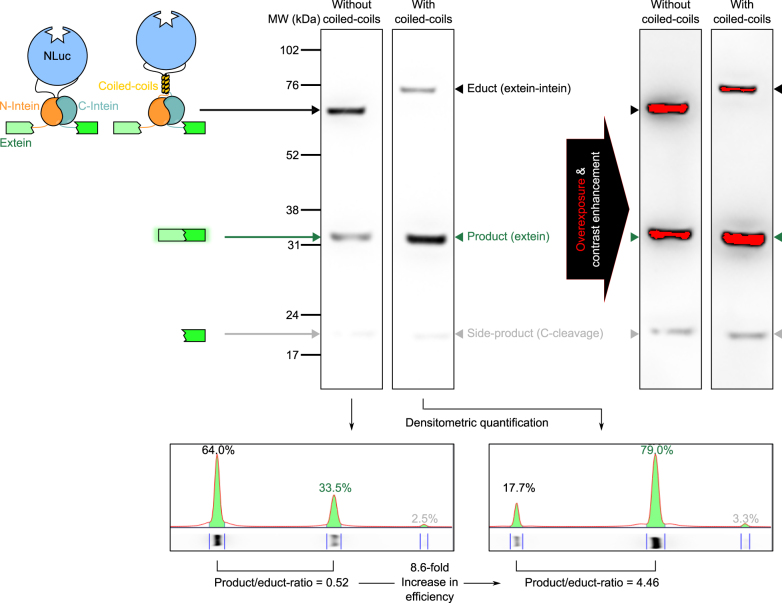
Improving internal split-inteins with coiled-coils as heterodimerization domains to support excision of the reporter module. The schematic on the top left panel depicts the construct containing split-inteins without (left) and with (right) addition of coiled-coils (yellow) flanked by the two halves of split-mNeonGreen (green rectangular shapes) serving as a model for exteins with extremely fast folding rates of less than 10 minutes. The immunoblot analysis of mNeonGreen (FLAG-tag, 24 hours post-transfection) showed a substantial increase of the resulting extein for the construct with the coiled-coils. Strong overexposure reveals a small fraction of side products (C-cleavage product C-mNeonGreen). Data shown represent 2 independent experiments. Unprocessed blots are provided in Source Data Extended Data Fig. 1. Source data

Using CRISPR–Cas9, we inserted NanoLuc luciferase (NLuc)^24^ homozygously into *MAPT* exon 10 of HEK293T cells flanked by the recently discovered ultra-fast splicing split–intein pair gp41-1 (ref. ^19^) (Supplementary Figs. 1 and 2; details are provided at the Protocol Exchange^25^). Importantly, to further accelerate intein splicing, we introduced bioorthogonal anti-parallel coiled-coil (CC) domains, which enhance the co-folding of the split–intein binary complex^26^ (Extended Data Fig. 1).

To enable ratiometric read-out of exon 10 containing tau isoforms referenced against total tau (pan-tau), we homozygously inserted firefly luciferase (FLuc), flanked by a second bioorthogonal set^27^ of fast splicing inteins (NrdJ-1)^19^, into the non-alternatively spliced exon 11 (Fig. 1a,b). As a consequence, the NLuc signal from the ratiometric EXSISERS_*MAPT*:10NLuc-11FLuc_ cells represents the expression of exon-10-specific 4R isoforms, whereas FLuc luminescence from a bioorthogonal substrate indicates the cumulative expression of all tau isoforms (Fig. 1a,b).

**Extended Data Fig. 2:**
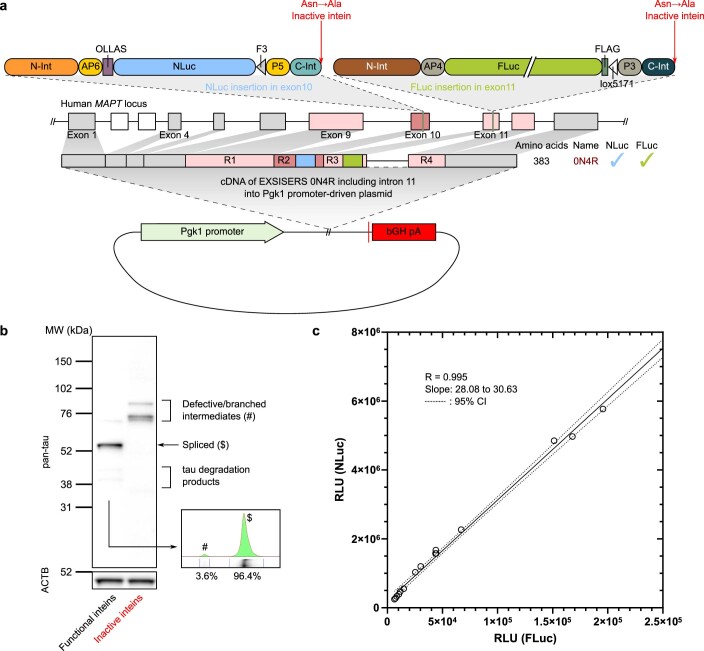
Characterization of dual-luciferase EXSISERS_*MAPT*:10NLuc-11Fluc_. **a**, EXSISERS_*MAPT*:10NLuc/11FLuc_, 0N4R CDS containing the constitutive intron 11-12 was cloned into a mouse Pgk1 promoter-driven plasmid including the intron between exon 11 and 12. Additionally, mutations that disrupt intein splicing (C-terminal Asn→Ala mutation) were created to show their importance for efficient protein splicing. **b**, Immunoblots against pan-tau from cells expressing the two constructs described in **a** with CCs, either with an active or a catalytically inactive intein. Data shown represent 2 independent experiments. **c**, Increasing amounts of Pgk1 promoter-driven EXSISERS_*MAPT*:10NLuc/11FLuc_ 0N4R CDS, driving the expression of both luciferases at 1:1 stoichiometry, were transfected into HEK293T cells. The resulting NLuc signal (RLU) is plotted against the FLuc signal (RLU) with a linear fit through the data points, revealing that inclusion of both exons results in NLuc signals that are ~30-fold (95% confidence interval (CI) from 28.08 to 30.63) stronger than FLuc. The reference curve was obtained from one experiment. Unprocessed blots are provided in Source Data Extended Data Fig. 2. Source data

To confirm that intein splicing does not affect isoform expression, we cloned the corresponding 0N4R isoform cDNA into a plasmid for strong overexpression of the dual-luciferase reporter system. A clear 0N4R band but hardly any unspliced protein was detected in contrast to catalytically inactive inteins (C-gp41-1_N37A_, C-NrdJ-1_N40A_; Extended Data Fig. 2a,b). To assess the relative bioluminescence signal strength of NLuc and FLuc, we transfected cells with increasing amounts of plasmids to express both luciferases in a 1:1 stoichiometry. We found a linear relationship between the relative luminescence units (RLU) over a wide range of values and a ~30-fold brighter signal for NLuc over FLuc (Extended Data Fig. 2c).

In addition to the stable EXSISERS line for wild-type *MAPT*, we also generated a line for the well-known *MAPT* IVS10+16 C>T mutation^28–30^, which shifts the ratio of 4R/pan-tau towards 4R (EXSISERS_*MAPT*:10NLuc-11FLuc_ IVS10+16 C>T). When *MAPT* expression was stimulated in these EXSISERS lines by dCas9 transactivators^31^, immunofluorescence showed the typical cytosolic tau staining as in unmodified HEK293T cells (Supplementary Fig. 3a).

Immunoblot analysis confirmed that the insertion of the reporter did not change the splicing pattern compared to unmodified HEK293T cells (left lane), indicating scarless post-translational excision of the reporters (Fig. 1c and Supplementary Figs. 3–5). Addition of the disease-associated IVS10+16 C>T hairpin-destabilizing mutation led to a visible increase of the 4R isoform (Fig. 1c (right lane) and Supplementary Fig. 3b), which was also validated in independent clones (Supplementary Figs. 4 and 5). The amount of excised NLuc (OLLAS-tagged) was substantially increased in the IVS10+16 C>T reporter line, corresponding to a relative increase in the 4R-tau isoform.

This pattern was reproduced by the dual-luciferase read-out that revealed an approximately fourfold increase in the NLuc/FLuc ratio (*P* = 0.0001 for uninduced and *P* < 0.0001 for induced; two-way analysis of variance (ANOVA) with Bonferroni multiple-comparisons test (MCT)) for the C>T hairpin modification, consistent with the literature, which reports an increase of twofold to sixfold^32,33^ (Fig. 1d and Supplementary Table 1 (results of the statistical tests)).

Protein splicing enhanced by CCs was very efficient in EXSISERS_*MAPT*:10NLuc-11FLuc_ lines, and prespliced products could only be detected after heavy overexposure and contrast enhancement (Supplementary Fig. 5). All other clones with the IVS10+16 C>T mutation also showed increased 0N4R expression compared with 0N3R. We also analysed additional unmodified HEK293T subclones besides the parental HEK293T and confirmed that 4R tau bands were only faintly visible (Supplementary Fig. 6).

Furthermore, an alternative insertion, 2 amino acids (6 nucleotides) downstream of the original insertion site in exon 10 did not yield any obvious difference in splicing pattern compared to unmodified HEK29T cells, indicating the robustness of the method (Supplementary Fig. 7).

**Extended Data Fig. 3:**
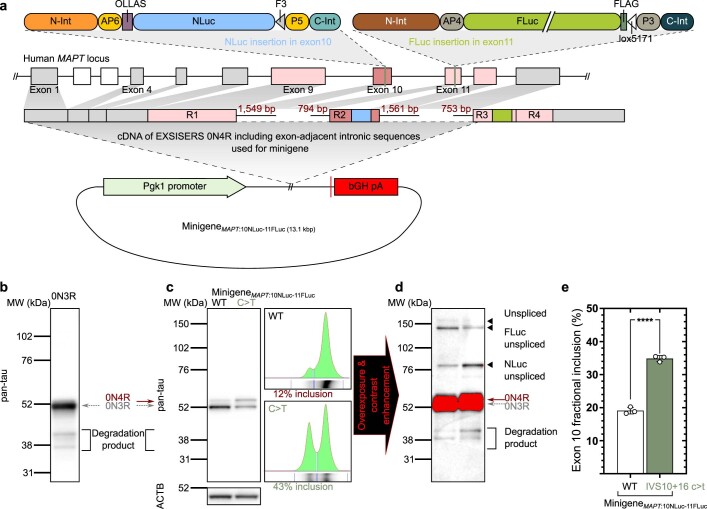
Characterization of minigene variants of *MAPT* with integrated dual-luciferase EXSISERS constructs. **a**, A *MAPT* 4R-minigene was created by amplifying exon-adjacent intronic sequences between exon 9 and 10, and between 10 and 11. **b**, Immunoblot analysis (anti-pan-tau) of HEK293T cells, transiently transfected with a Pgk1 promoter-driven plasmid overexpressing *MAPT* 0N3R CDS yield a single 0N3R protein species and minor tau degradation products. **c**, Immunoblot analysis (anti-pan-tau) of HEK293T cells transfected with a minigene described in **a**) and its corresponding densitometric analysis (dephosphorylated lysate). Please note that it is challenging to obtain sufficiently separated bands for tau isoforms to perform precise densitometric quantification. Data shown in b and c represent 2 independent experiments. **d**, Overexposed and contrast-enhanced images of **c**) showed minor amounts of tau breakdown products and unspliced variants of the overexpressed luciferases. **e**, Fractional inclusion of exon 10 in HEK293T cells transfected with the minigene constructs described in **a**. By applying the conversion for the relative luminescence brightness of FLuc and NLuc (Extended Data Fig. 2c), the fractional inclusion of exon 10 for the WT-minigene variant is ~19% an 35% for the IVS10+16 C>T minigene. Data shown represent the mean ± s.d (*n* = 3 biological replicates), **** denotes a *p*-value smaller than 0.0001 in a two-tailed unpaired t-test (full statistical results are available in Supplementary Table 1). Unprocessed blots and numerical source data are provided in Source Data Extended Data Fig. 3. Source data

As a further control, we also created a minigene version of EXSISERS (driven by mouse *Pgk1* promoter), similar to previous research^34,35^, in which only the exon-flanking portions between exon 9 and 10 and between exon 10 and 11 were cloned into the tau CDS and expressed transiently (Extended Data Fig. 3a). Consistent with previous reports^34,35^, we observed greatly increased inclusion of exon 10 (NLuc) for the minigene version of 12% compared with unmodified HEK293T cells, which showed only ~3–4% fractional exon 10 inclusion after *MAPT* induction (Extended Data Fig. 3b–e). By contrast, EXSISERS_*MAPT*:10NLuc-11FLuc_ showed a fractional exon 10 inclusion of ~4% on the basis of immunoblot and bioluminescence analysis, similar to the parental HEK293T cells (Fig. 1c and Supplementary Fig. 3b,c).

### Single-cell and longitudinal monitoring of *MAPT* exon 10 usage

**Fig. 2:**
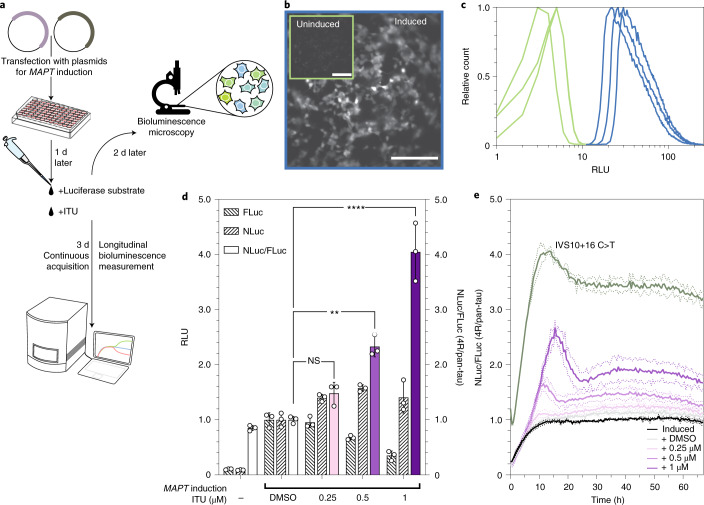
Live imaging and longitudinal readout of *MAPT* isoform expression. **a**, EXSISERS_*MAPT*:10NLuc-11FLuc_ HEK293T cells were transfected with CRISPR-dCas9-VPR plasmids to induce *MAPT* expression. One day after transfection, luciferase substrates and ITU were added at the indicated concentrations. Two days later, cells were imaged using a bioluminescence live imaging system, or cells were measured longitudinally for 3 d. **b**, Bioluminescence imaging enabled longitudinal monitoring of 4R-tau in the *MAPT-*exon-10-tagged HEK293T line at cellular resolution. Respective images were taken 72 h after induction/transfection. Scale bars, 200 μm. **c**, Histograms of the luminescence signals obtained from three cell dishes without and with *MAPT* induction. **d**, RLU of FLuc and NLuc (normalized to DMSO as the vehicle control), and their ratio as a function of increasing the concentrations of the DYRK1A/GSK3A inhibitor ITU in EXSISERS_*MAPT*:10NLuc-11FLuc_ HEK293T cells. Data are mean ± s.d., *n* = 3 biological replicates. Selected results of Bonferroni MCT after one-way ANOVA are shown; **P < 0.01, ****P < 0.0001; NS, not significant (full statistical results are provided in Supplementary Table 1). **e**, Pan-tau and 4R-tau expression was monitored longitudinally through FLuc and NLuc in the double-luciferase EXSISERS HEK293T cell line. Furthermore, 24 h after transfection, ITU at the indicated concentrations, d-luciferin (FLuc substrate) and endurazine (NLuc pro-substrate) were added and measured over a timecourse of 67 h. Data are mean ± s.d. (dotted lines), *n* = 3 biological replicates. Source data are available online. Source data

We next assessed EXSISERS for non-invasive imaging and multi-time-point analysis of *MAPT* exon 10 usage (Fig. 2a). Using bioluminescence microscopy, we resolved individual cells 2 d after the addition of luciferase substrate to plot the distribution of the luminescence signal over cell populations without or with *MAPT* induction (Fig. 2b,c).

**Extended Data Fig. 4:**
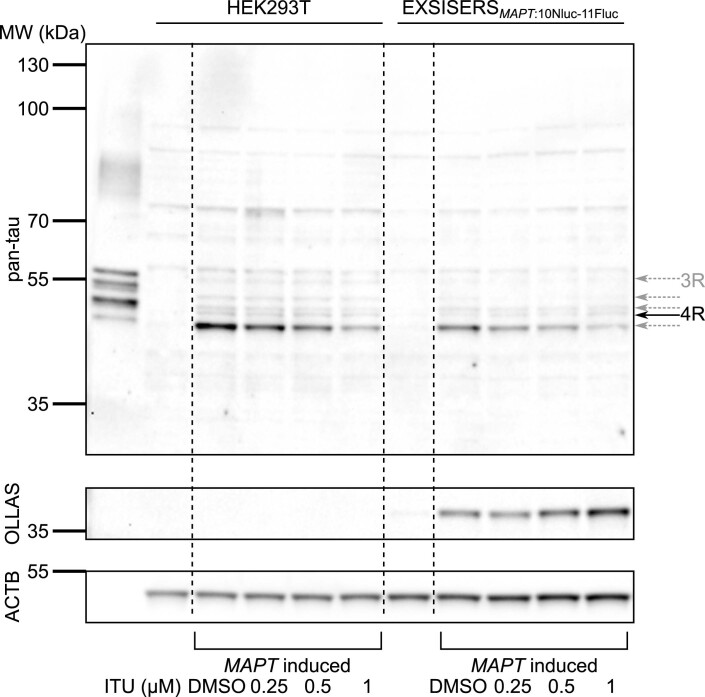
Uncropped immunoblot analysis of 5-iodotubercidin (ITU) effect on tau isoform expression in HEK293T and EXSISERS_*MAPT*:10NLuc-11FLuc_. *MAPT* was induced in the indicated cells, which were subsequently treated with increasing concentrations of ITU. Anti-pan-tau and anti-OLLAS ( = 4R reporter) immunoblot analysis showed a concentration-dependent inclusion of exon 10 in both unmodified HEK293T and EXSISERS_*MAPT*:10NLuc-11FLuc_ cells (dephosphorylated cell lysates). Data shown for unmodified HEK293T cells or EXSISERS lines represent 3 or 4 independent experiments with similar results, respectively. Unprocessed blots are provided in Source Data Extended Data Fig. 4. Source data

Next, we tested whether the effect of small molecules on exon 10 inclusion can be reliably followed by ratiometric EXSISERS_*MAPT*:10NLuc-11FLuc_. We chose 5-iodotubercidin (ITU)^36–38^, a DYRK1A and GSK3A inhibitor that stimulates exon 10 inclusion through SRSF2 (also known as SC35)^39–41^. After application of ITU, we observed a concentration-dependent increase of the NLuc/FLuc-ratio to up to fourfold (*P* < 0.0001, one-way ANOVA with Bonferroni MCT; Fig. 2d). Dose-dependent expression of 4R-tau was validated using immunoblot analysis of unmodified HEK293T and EXSISERS_*MAPT*:10NLuc-11FLuc_ cells (Extended Data Fig. 4 and Supplementary Fig. 8).

We next examined the effect of ITU over time and observed an increase in the 4R/pan-tau ratio during *MAPT* induction, followed by a dose-dependent offset of the ratio, reaching a plateau after 1 d. However, the maximum effect of ITU was still only approximately half of that measured for the IVS10+16 C>T mutation without ITU treatment (Fig. 2e).

### EXSISERS lines from hiPSCs for screenings

**Fig. 3:**
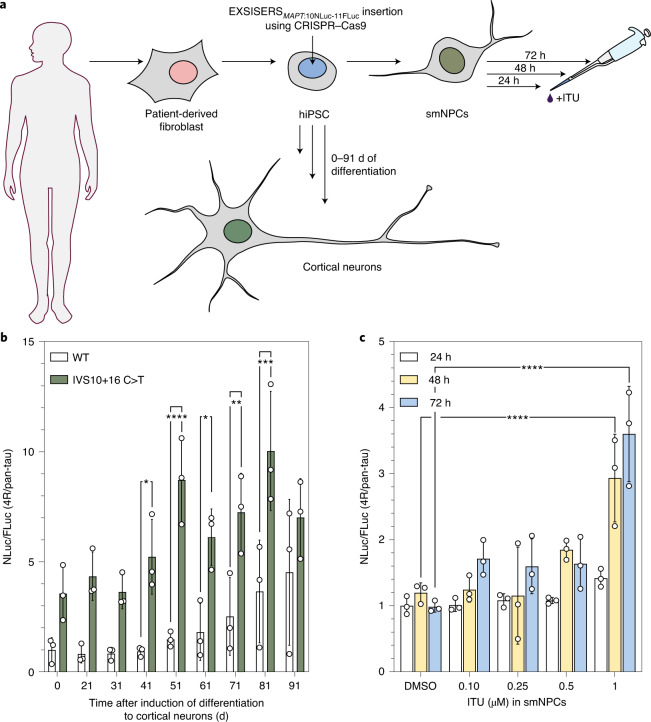
hiPSC-derived EXSISERS lines for pharmacological screening. **a**, smNPCs were generated from hiPSCs into which EXSISERS was introduced into exon 10 and 11 (EXSISERS_*MAPT*:10NLuc-11FLuc_). **b**, WT and IVS10+16 C>T hiPSCs were differentiated into cortical neurons over three months. The Nluc/Fluc ratios are shown normalized to WT at day 0. **c**, The NLuc/FLuc ratios (4R/pan-tau) are plotted as a function of increasing concentrations of the DYRK1A/GSK3A inhibitor ITU in EXSISERS_*MAPT*:10NLuc-11FLuc_ smNPCs after 24 h, 48 h and 72 h. For **b** and **c**, data are mean ± s.d., *n* = 3 biological replicates. Selected results of Bonferroni MCT after one-way ANOVA analysis are shown; *P < 0.05, **P < 0.01; ***P < 0.001, ****P < 0.0001 (full statistical results are provided in Supplementary Table 1). Source data are available online. Source data

To assess whether EXSISERS could be used in cells with endogenous tau expression, we knocked-in EXSISERS into *MAPT* of human induced pluripotent stem cells (hiPSCs). We then differentiated the EXSISERS_*MAPT*:10NLuc-11FLuc_ hiPSCs into cortical neurons while measuring the pan/4R-tau expression through FLuc over a timecourse of 3 months (Fig. 3a,b). In WT *MAPT*, we observed a gradual increase in the fractional 4R isoform expression as expected. Interestingly, in the case of the IVS10+16 C>T mutation, we detected an elevated 4R/pan-tau ratio already in the undifferentiated state despite the low pan-tau expression, which is challenging to detect using immunoblot^30^ (Fig. 3b). Subsequently, the fractional isoform expression increased non-monotonically with peaks at 51 d and 81 d.

Given the high sensitivity of isoform detection in this system, we next tested whether sufficient signal could already be obtained from EXSISERS_*MAPT*:10NLuc-11FLuc_ hiPSCs that were predifferentiated to small-molecule-derived neural precursor cells (smNPCs; Fig. 3a,c), which are more cost-effective and convenient to handle. Indeed, a clear effect of 1 µM ITU could be detected already after 48 h (*P* < 0.0001, two-way ANOVA with Bonferroni MCT; Fig. 3c).

These data demonstrate how the ratiometric, highly sensitive protein-level readout of EXSISERS enables cost-effective, multi-time-point quantification of isoform expression in small quantities of precious cell culture models for pharmacological screenings.

### RNA-targeting systems for isoform-specific knockdown of *MAPT*

**Extended Data Fig. 5:**
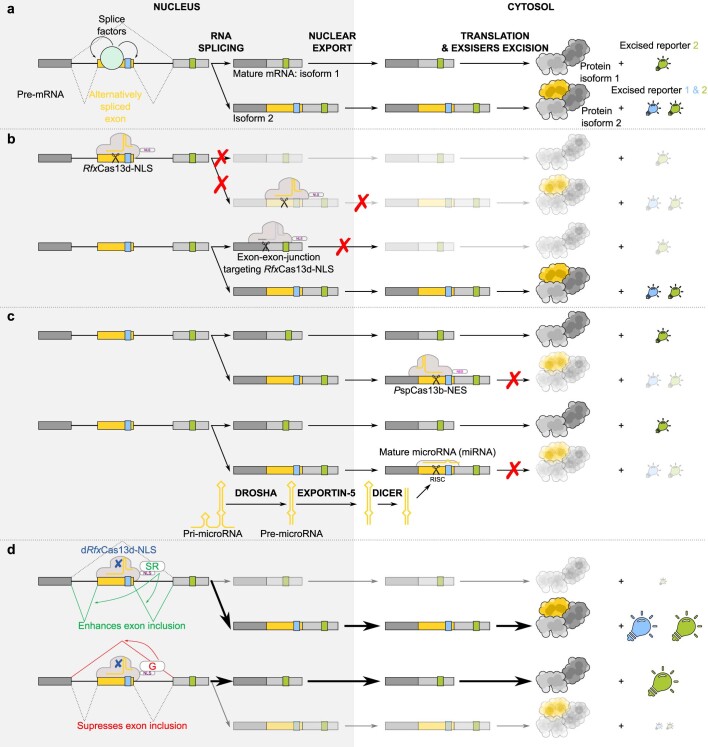
Schematic of the preferred sites of action for the different programmable RNA-targeting systems. Alternative splicing (AS) generates different isoforms of a single pre-mRNA, regulated by splice factors. **b**, RNA-guided perturbation using *Rfx*Cas13d-NLS in the nucleus is expected to be isoform-unspecific but can be made isoform-specific by targeting the exon-exon-junctions. **c**, In contrast, cytosolic *P*spCas13b-NES acts in the cytosolic environment and can thereby degrade isoforms without the necessity for targeting the exon-exon-junctions. Endogenously reprogrammed microRNAs can also efficiently act in the cytosol. In comparison to *P*spCas13b-NES, a few restrictions on the choice of the RNA sequence apply. **d**, In the case of the inactivated variant d*Rfx*Cas13d-NLS, splice enhancers/suppressors can be recruited to an exon of interest to modulate exon in- or exclusion resulting in an isoform shift.

With the ratiometric EXSISERS_*MAPT*:10-NLuc/11-FLuc_ cell lines in hand, we next sought to systematically optimize programmable CRISPR–Cas13 effectors for 4R-tau suppression. The parameters of interest were Cas13 type, subcellular localization, spacer length and targeting site in direct comparison to the latest generation of short hairpin RNAs (shRNAs). These parameters should influence isoform-specific expression at different time points and sites of action in the cell during the mRNA maturation process (Extended Data Fig. 5).

### Cas13d localization and spacer length

**Fig. 4:**
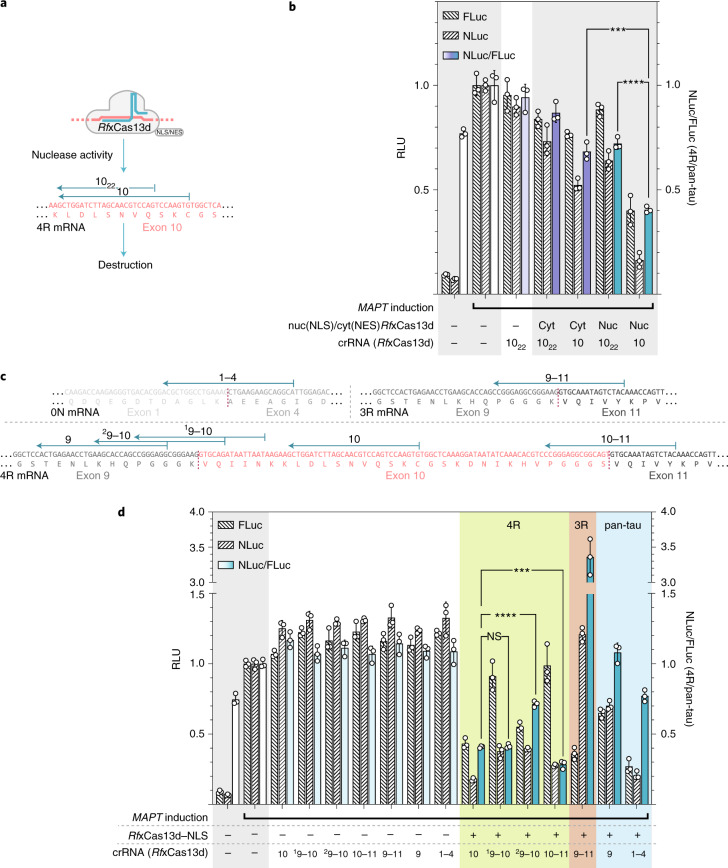
Optimization of Cas13d for isoform-specific perturbation of *MAPT*. **a**, Binding sites of *Rf*xCas13d–NLS/NES with a standard (22 nucleotides; 10_22_) and an extended (30 nucleotides; 10) spacer targeting *MAPT* exon 10. 30 nucleotides was used as the default length for all of the subsequent experiments such that we omit the subscript in the notation. **b**, Optimization of spacer length for *Rf*xCas13d measured through the depletion of induced tau, tracked using bioluminescence from NLuc (4R-tau) and FLuc (pan-tau) 72 h after transfection. RLU of the bioluminescence signals are given for FLuc and NLuc separately and as the NLuc/FLuc ratio calculated from each sample. Nuc, nuclear localization by NLS; Cyt, cytoplasmic localization by NES. **c**, Binding sites of the different *Rf*xCas13d crRNAs on the mature *MAPT* 0N, 3R and 4R mRNAs. **d**, The normalized FLuc, NLuc and NLuc/FLuc values are shown after transfection of EXSISERS_*MAPT*:10NLuc-11FLuc_ cells with different *MAPT*-targeting crRNAs to increase isoform specificity of *Rf*xCas13d–NLS. For **b** and **d**, data are mean ± s.d., *n* = 3 biological replicates. Selected results of Bonferroni MCT after two-way ANOVA (**b**) and one-way ANOVA (**d**) analysis are shown; ***P < 0.001, ****P < 0.0001; NS, not significant (full statistical results are provided in Supplementary Table 1). Source data are available online. Source data

According to recent reports, *Ruminococcus flavefaciens* XPD3002 Cas13d^42^ (*Rf*xCas13d) displayed more potent RNA-silencing activity in the nucleus than in the cytosol^42^ when targeting 4R-tau. We observed a small decrease in the 4R/pan-tau ratio when *Rf*xCas13d was equipped with a nuclear export signal (NES) in comparison to a nuclear localization signal (NLS; Fig. 4a,b). However, the overall knockdown (KD) activity remained unexpectedly weak in our experimental conditions, which were optimized for low levels of plasmids for transfection. Thus, we examined whether the length of the spacer could be improved.

Interestingly, we observed that *Rf*xCas13d suppresses 4R-tau (NLuc) more efficiently with an extended 30-nucleotide spacer compared with a 22-nucleotide spacer^42^, although at the expense of exon 10 specificity (*P* < 0.0001, two-way ANOVA with Bonferroni MCT; Fig. 4b). When combining the extended 30-nucleotide spacer with *Rf*xCas13d–NLS, the strongest reduction in 4R/pan-tau ratio was observed compared with the canonical 22-nucleotide CRISPR RNA (crRNA) (*P* < 0.0001, two-way ANOVA with Bonferroni MCT). As 4R-tau represents ~3–4% of the total tau in HEK293T-derived cells (Supplementary Fig. 3b,c and Supplementary Table 1 (full statistical results)), the 60% reduction in pan-tau (FLuc, *P* < 0.0001, one-way ANOVA with Bonferroni MCT) suggests that also 3R-tau was affected.

**Extended Data Fig. 6:**
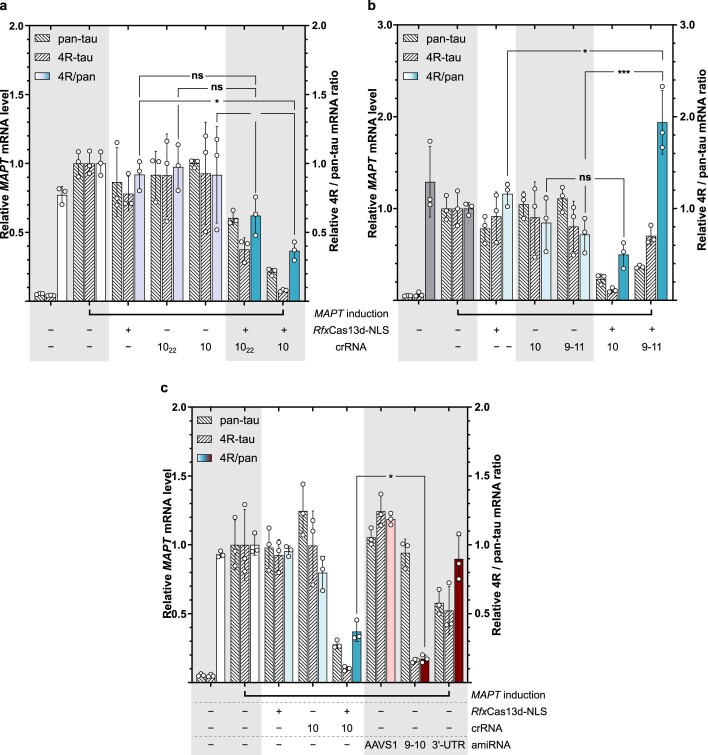
Validation of key results of isoform-specific knockdown of MAPT with RT-qPCR. **a**, Optimization of spacer length for *Rfx*Cas13d with a nuclear localization sequence (NLS) measured via depletion of induced tau, tracked via mRNA levels of 4R-tau and pan-tau 72 h post-transfection. **b**, Targeting of exon-exon junctions to increase isoform specificity of *Rfx*Cas13d-NLS. **c**, Comparison of isoform specificity in cytosolic targeting of mature mRNA with different amiRNAs. Data shown in **a-c** represent the mean values ± s.d. (*n* = 3 biological replicates). Selected results are shown for a two-way ANOVA with Bonferroni MCT (**a**,**b**), or two-tailed unpaired *t*-test between the two exon-10 targeting effectors (**c**), where* and *** denote *p*-values smaller than 0.05 and 0.001 (full statistical results are available in Supplementary Table 1). Numerical source data are provided in Source Data Extended Data Fig. 6. Source data

We validated these results using RT–qPCR on unmodified HEK293T cells and observed a significant reduction in the 4R/pan-tau ratio with an extended 30-bp crRNA (*P* = 0.0397, two-way ANOVA with Bonferroni MCT) but not with a 22-nucleotide crRNA (*P* = 0.9687, two-way ANOVA with Bonferroni MCT; Extended Data Fig. 6a). To confirm this observation independently from tau, we used *Rf*xCas13d–NLS to target a co-transfected plasmid expressing NLuc. We again detected an increased potency with the extended spacer (80% KD versus 30% KD, *P* < 0.0001; Supplementary Fig. 9).

### Targeting exon–exon junctions with nuclear-localized Cas13d

One strategy to render *Rf*xCas13d–NLS more isoform-specific is to target the spliced mRNA, which contains exon–exon junctions (Extended Data Fig. 5b).

When 3R-tau was targeted through the exon 9–11 junction (Fig. 4c), an increase in the NLuc/FLuc-ratio was observed compared with the antisense crRNA-only control, indicating a specific 3R reduction (*P* < 0.0001, one-way ANOVA with Bonferroni MCT; Fig. 4d, red). As expected, targeting the constitutive exon 9 showed no discrimination between both isoforms (Fig. 4d, blue). Similarly, targeting the 0N isoform (exon 1–4 junction) also yielded no isoform specificity, as exons 2 and 3 are exon 10 independent (Fig. 4d, blue).

We next compared crRNA directed against exon 10 with cRNAs targeting either the exon 9–10 junction or the 10–11 junction (Fig. 4d, green). Targeting the exon 9–10 junction symmetrically (^1^9–10; Fig. 4c) did not result in a decrease in the 4R/pan-tau ratio, indicating isoform-specific ablation (*P* > 0.9999). Moreover, an asymmetric junction-spanning cRNA (^2^9–10; Fig. 4c) increased the 4R/pan-tau ratio (*P* < 0.0001, one-way ANOVA with Bonferroni MCT across crRNAs targeting exon 10; Supplementary Table 1).

By contrast, targeting the 10–11 junction showed a significantly better isoform specificity compared with targeting exon 10 directly (*P* = 0.0004, one-way ANOVA with Bonferroni MCT among crRNAs against exon-10-encoding transcripts; Fig. 4d, green).

To confirm that exon-spanning crRNAs lead to better isoform specificity, we independently tested the 3R-specific crRNA (9–11 exon–exon junction) on unmodified HEK293T cells and quantified it using RT–qPCR. As expected, targeting the 9–11 exon junction led to a specific isoform depletion, as seen by the increase in the 4R/pan-ratio (*P* = 0.0133 and *P* = 0.0002, two-way ANOVA with Bonferroni MCT) compared with protein or antisense control (Extended Data Fig. 6b). The increase in the ratio can be explained by the decrease in pan-tau (*P* = 0.0287 and *P* = 0.0002, two-way ANOVA with Bonferroni MCT compared with the protein control or antisense control, respectively) while 4R remained unchanged (*P* > 0.9999 for both the protein and antisense control comparison), indicating that the 3R-tau is specifically depleted. However, when exon 10 was directly targeted, both pan-tau and 4R-tau were depleted compared with the antisense control (*P* < 0.0001 and *P* = 0.0236, respectively, two-way ANOVA with Bonferroni MCT; Supplementary Table 1), while the 4R/pan-tau ratio remained unchanged (*P* > 0.9999; Extended Data Fig. 6b), indicating an unspecific depletion of all isoforms.

**Extended Data Fig. 7:**
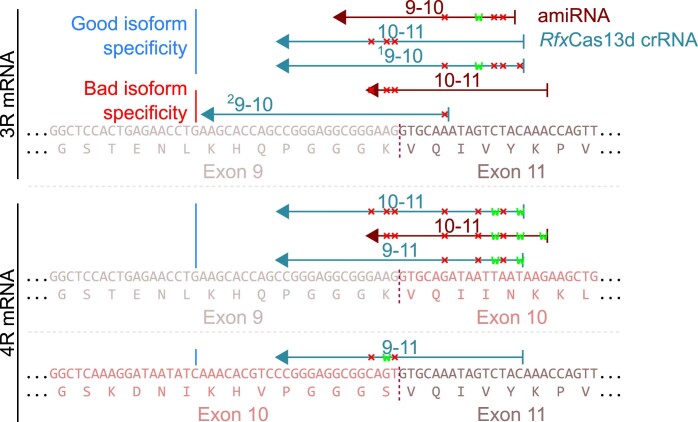
Mapping of potential off-target binding sites of *Rfx*Cas13d/*P*spCas13b crRNAs. The number indicated above the binding sites denotes the targeted exonic region of *MAPT* (*for example*, 10-11 is the intended targeting of the exon-junction 10-11). ‘x’ indicates mismatches on the isoform off-target sites, and ‘w’ indicates wobble base pairs (G-U pairings). 10-11 targeting amiRNA has 3 terminal mismatches, which have been shown to be well-tolerated in shRNAs, resulting in a depletion of both 4R and 3R tau isoforms. ^2^9-10 crRNA (*Rfx*Cas13d-NLS) also matches the 9-11 junction resulting in an unspecific depletion of both 4R and 3R tau.

A possible explanation for why some crRNAs targeting an exon–exon junction are not specific towards the targeted isoform is the asymmetrical positioning of the cRNA on the exon–exon junction (^2^9–10 crRNA, 24 nucleotides on exon 9, 6 nucleotides on exon 10; Fig. 4c and Extended Data Fig. 7) resulting in a greater isoform promiscuity (NLuc/FLuc-ratio of ^2^9–10 crRNA increased compared with ^1^9–10, *P* < 0.0001, one-way ANOVA with Bonferroni MCT across exon10-targeting crRNAs; Fig. 4d (green) and Supplementary Table 1). This result was expected as this particular crRNA also matches the 9–11 (3R) junction with a one-nucleotide terminal mismatch only (Extended Data Fig. 7), which has been reported to be tolerated by Cas13 systems^43,44^.

### Programmable mRNA KD in the cytosol

An alternative strategy to increase isoform specificity is to target mature mRNAs in the cytosol, in which unspliced nuclear mRNAs cannot be affected (Extended Data Fig. 5c). As *Rf*xCas13d shows superior performance in the nucleus (Fig. 4a,b), we chose *Prevotella* sp. P5–125 Cas13b (*P*spCas13b) instead, which has been shown to work in the cytosol^43^.

**Fig. 5:**
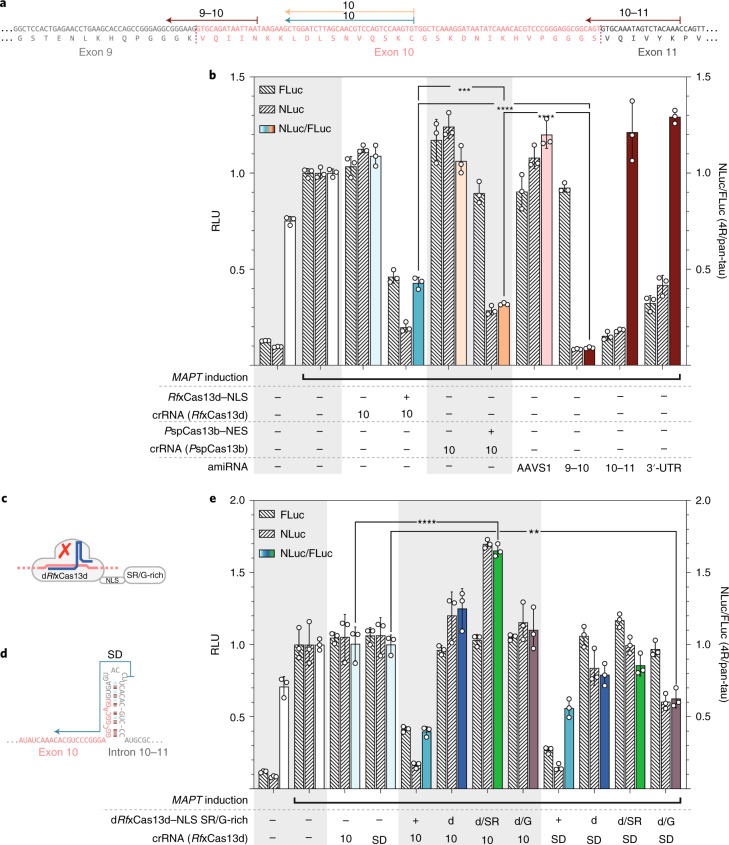
Comparison of nuclear Cas13d with cytosolic RNA-guided RNA-targeting systems and Cas13d-based splicing suppression and enhancement. **a**, Binding sites of *Rf*xCas13d–NLS (cyan), *P*spCas13b–NES (orange) and amiRNA (dark red) on the mature *MAPT* 4R mRNA. **b**, Isoform specificity of cytosolic targeting of mature mRNA by Cas13b compared to amiRNA. The normalized FLuc, NLuc and NLuc/FLuc values after transfection of EXSISERS_*MAPT*:10NLuc-11FLuc_ cells with different RNA systems and positions (shown in **a**) are displayed. **c**, G-rich/SR-rich domains were fused to a nuclease-inactivated version of *Rf*xCas13d–NLS (d*Rf*xCas13d–NLS) to create RNA-guided splice suppressors (G-rich) or enhancers (SR-rich). **d**, The binding site of the splice donor (SD)-targeting crRNA; the position of the crRNA 10 is shown in **a**. **e**, Different d*Rf*xCas13d–NLS fusions were tested for their ability to modulate *MAPT* exon 10 splicing. The normalized FLuc, NLuc and NLuc/FLuc values after transfection of EXSISERS_*MAPT*:10NLuc-11FLuc_ cells with different RNA systems and positions (shown in **a**, **c** and **d**) are displayed. d, d*Rf*xCas13d–NLS; SR-rich, serine/arginine-rich domain of SC35; G-rich, glycine-rich domain from HNRNPA1 isoform A1-B; d–SR, fusion of d*Rf*xCas13d–NLS to the SR-rich domain; d–G, fusion of d*Rf*xCas13d–NLS to the G-rich domain. For **b** and **e**, data are mean ± s.d., *n* = 3 biological replicates. Selected results of Bonferroni MCT after one-way ANOVA are shown; **P < 0.01, ***P < 0.001, ****P < 0.0001 (full statistical results are provided in Supplementary Table 1). Source data are available online. Source data

Indeed, targeting the same region of exon 10 (Fig. 5a), *P*spCas13b–NES showed a better 4R-specificity than *Rf*xCas13d–NLS indicated by a decreased NLuc/FLuc-ratio (*P* = 0.0008, one-way ANOVA with Bonferroni MCT; Fig. 5b (blue bar versus orange) and Supplementary Table 1).

We were next interested in comparing the performance of the Cas13b/d variants with artificial microRNAs (amiRNAs), which mimic the endogenous microRNA biogenesis pathway and preferentially act in the cytosol^45,46^ (Extended Data Fig. 5c).

Indeed, the highest isoform specificity was achieved using the third-generation amiRNA targeting the 9–10 junction (*P* < 0.0001 for 9–10 amiRNA versus 10/13b_NES_ and *P* < 0.0001 for 9–10 amiRNA versus 10/13d_NLS_, one-way ANOVA with Bonferroni MCT of 10/13d_NLS_ versus 10/13b_NES_ versus 9–10 amiRNA; Fig. 5b, left red bar). By contrast, targeting of the 3′ untranslated region or the 10–11 junction resulted in high isoform promiscuity (*P* > 0.9999 for both, one-way ANOVA with Bonferroni MCT; Fig. 5b and Supplementary Table 1), similar to the exon ^2^9–10 asymmetrical-targeting *Rf*xCas13d–NLS (Fig. 4c,d and Extended Data Fig. 7).

RT–qPCR analysis of unmodified HEK293T cells confirmed a significant exon-10-specific isoform ablation, as observed by a significant reduction in the 4R/pan-tau ratio, using both systems—*Rf*xCas13d (*P* < 0.0001 and *P* = 0.001, one-way ANOVA with Bonferroni MCT compared with the protein control or antisense control; Supplementary Table 1) and amiRNA (*P* < 0.0001, one-way ANOVA with Bonferroni MCT compared with the *AAVS1*-targeting control; Supplementary Table 1). When comparing only the conditions targeting exon-10-containing transcripts, we found that amiRNAs are more isoform-specific than *Rf*xCas13–NLS (*P* = 0.0112, two-tailed unpaired *t*-test; Extended Data Fig. 6c).

### d*Rf*xCas13d–NLS as an RNA-guided splicing enhancer or suppressor

We also created additional versions of d*Rf*xCas13d–NLS for use as programmable splice enhancers or suppressors^42^ (Extended Data Fig. 5d). Fusing d*Rf*xCas13d–NLS to the glycine-rich (G-rich) domain of HNRNPA1 (amino acids 187–320 of isoform A1)^42,47^ yielded an RNA-guided splice suppressor, whereas fusion to the serine–arginine-rich (SR-rich) domain of SC35 (amino acids 90–271)^47,48^ yielded a splice enhancer (Fig. 5c and Supplementary Fig. 10a). Two crRNAs were tested, the first targeting exon 10 directly, and the second targeting the splice donor and the regulatory hairpin (Fig. 5d). When directed to exon 10, only the d*Rf*xCas13d–NLS fusion to the SR-rich domain resulted in a significant increase in exon 10 inclusion (*P* < 0.0001, one-way ANOVA with Bonferroni MCT; Fig. 5e, middle, green bar). On the contrary, the combination of the splice donor/hairpin-binding crRNA with the fusion to the G-rich domain resulted in a significant decrease in exon 10 inclusion compared with the crRNA-only control (*P* = 0.0014; Fig. 5e, right, brown bar). These results were replicated in independent clones of EXSISERS_*MAPT*:10NLuc-11FLuc_ (Supplementary Fig. 10a,b) and EXSISERS_*MAPT*:10NLuc-11FLuc_ IVS10+16 C>T (Supplementary Fig. 10a,c).

### Modular reporters and effectors for EXSISERS

**Extended Data Fig. 8:**
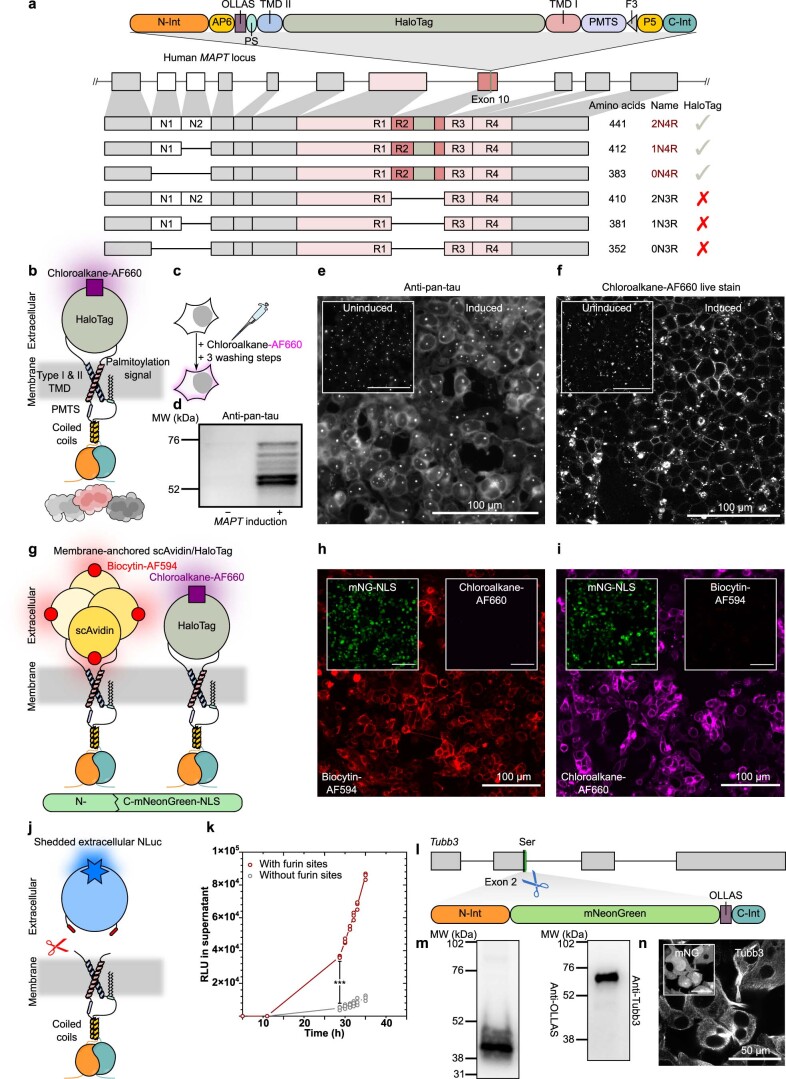
EXSISERS can be programmed with different reporters and effectors. **a**,**b**, HaloTag was flanked by intein-CC domains and type I and type II transmembrane domains (TMDs), resulting in a surface functionalization with a HaloTag inserted into exon 10 of *MAPT*. **c**, Cells were incubated with chloroalkane-functionalized Alexa Fluor 660 (3.5 µM) for 10 min at 37 °C and washed with HBSS. **d**, Anti-pan-tau immunoblot showed the typical tau pattern indicating scarless excision. Data shown represent one independent experiment. **e**, Epifluorescence microscopy image of anti-pan-tau immunocytochemistry of EXSISERS_*MAPT*:10Halo_ cells after *MAPT* induction showing filamentous tau staining. Data shown represent 2 independent experiments **f**, AF660 live-cell-staining after *MAPT*-induction resulted in a distinct membrane staining. Data shown represent 2 independent experiments. **g**, scAvidin was used as a high-affinity binding domain for biotinylated compounds. Test constructs for both designs contained mNG-NLS as surrogate exteins, which are expected to be spatially decoupled after the excision of the intein-domains. **h**,**i**, Surface localization of scAvidin or HaloTag in transfected HEK293T was confirmed by live-staining with biocytin-AF594 and chloroalkane-AF660, while mNeonGreen-NLS (mNG-NLS) was detected in the nucleus. Data shown represent one experiment from 2 cell culture wells. **j**, The binding moiety of the membrane anchors was exchanged for NLuc flanked by furin sites (FS), such that the Golgi-resident furin can cleave off NLuc and releases it into the supernatant. **k**, NLuc activity was measured over time in the supernatant of cells expressing the sheddable NLuc without/with furin sites. Data shown represent the mean values ± s.d. (*n* = 3 biological replicates). *** denotes *p* < 0.001 of selected Bonferroni MCT after two-way ANOVA with repeated measures (full statistical results in Supplementary Table 1). **l**, Intein-flanked mNG was inserted into the mouse *Tubb3* gene. **m**, Immunoblot confirms successful intein splicing of mNG. Data shown represent 7 independent experiments. **n**, Mouse N2a cell line with insertion of the intein-mNG reporter into *Tubb3* shows typical Tubb3 filaments via immunocytochemistry. The fluorescent mNG signal (inset) was observed throughout the cell, indicating successful post-translational splicing. Data shown represent 2 independent experiments. Unprocessed blots and numerical source data are provided in Source Data Extended Data Fig. 8. Source data

In addition to high-sensitivity bioluminescence detection, we also enabled sensitive fluorescence imaging of exon 10 usage by inserting a membrane-presented HaloTag^49^ (EXSISERS_*MAPT*:10-Halo_) or single-chain chicken avidin (scAvidin)^50^ (Extended Data Fig. 8a–i). We also functionalized a transmembrane domain (TMD) with the luciferase NLuc, flanked by furin endoprotease cleavage sites to release the reporter into the extracellular environment enabling convenient longitudinal measurements from the supernatant (Extended Data Fig. 8j,k). Canonical fluorescent proteins can also be used with EXSISERS but require strong expression of the host gene to be reliably detected (compare Extended Data Fig. 8i–n and Supplementary Fig. 11a,b). These EXSISERS modules enable non-invasive live staining, efficient cell enrichment using fluorescence-activated cell sorting (FACS) or magnetic cell separation system (MACS), and convenient sampling from the supernatant for high-throughput screening applications.

### Quantifying co-translational ribosomal frameshift regulation

**Extended Data Fig. 9:**
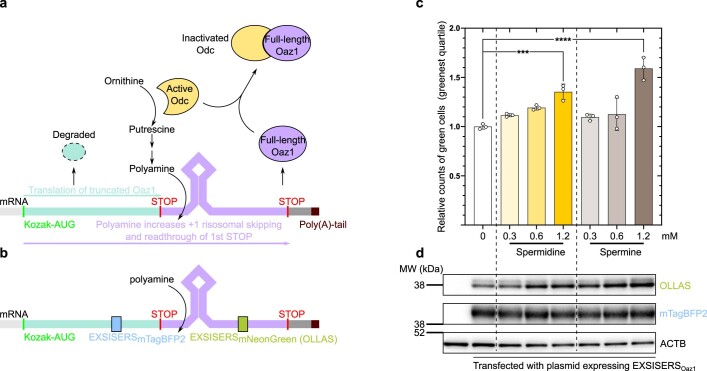
EXSISERS enables non-invasive protein-level quantification of co-translation regulation. **a**, The antizyme Oaz1 is regulated co-translationally by ribosomal frameshifting, which strongly depends on polyamine levels such as spermidine and spermine. Increasing polyamine levels lead to a +1 frameshift and skipping of the in-frame stop codon providing the full-length Oaz1 antizyme. The usage of the in-frame stop codon otherwise will lead to truncated non-functional Oaz1. Full-length Oaz1 binds and inactivates the enzyme Odc, the rate-limiting enzyme in the polyamine biosynthesis pathway, resulting in a product-mediated closed-loop homeostatic regulation of polyamine levels. **b**, EXSISERS_*Oaz1*-mTagBFP2_ and EXSISERS_*Oaz1-*mNeonGreen_ were inserted into a plasmid harboring the full-length *Oaz1* gene up- and downstream of the regulatory hairpin with the in-frame stop codon. **c**, FACS analysis of EXSISERS_*Oaz1*_-transfected cells treated with different polyamine levels. The first gate (blue) ensured analysis of transfected cells only (Supplementary Information). Transfected cells were analyzed by counting the fraction of cells passing the green gate, which was set to contain the greenest 25% in the untreated condition (0 mM). Data points represent the mean values ± s.d. (*n* = 3 biological replicates). **d**, Immunoblot from the lysate of the corresponding samples shown in **c**. ***, and **** denotes *p* < 0.001, and *p* < 0.0001, one-way ANOVA, of Bonferroni MCT against vehicle control (full statistical results are available in Supplementary Table 1). Unprocessed blots and numerical source data are provided in Source Data Extended Data Fig. 9. Source data

Ribosomal frameshift-mediated regulations cannot be monitored by RT–qPCR or other RNA-based quantification methods. Dysregulation of this process in Oaz1, the key enzyme in polyamine biosynthesis (Extended Data Fig. 9a), results in various diseases, such as Snyder–Robinson Syndrome and cancer^51^. We inserted EXSISERS_*Oaz1*-mTagBFP2_ and EXSISERS_*Oaz1*-mNeonGreen_ into full-length *Oaz1* and treated transfected cells with different polyamine concentrations to check whether we can read out frameshift regulation using fluorescence quantification (Extended Data Fig. 9b). FACS analysis revealed that the stop codon readthrough was significantly stimulated by increasing spermidine or spermine concentrations (*P* = 0.0009 for 1.2 mM spermidine, *P* < 0.0001 for 1.2 mM spermine, one-way ANOVA with Bonferroni MCT against vehicle control; Extended Data Fig. 9c), which was also confirmed using immunoblot analysis (Extended Data Fig. 9d).

### Identification of regulators of isoform-specific expression

**Fig. 6:**
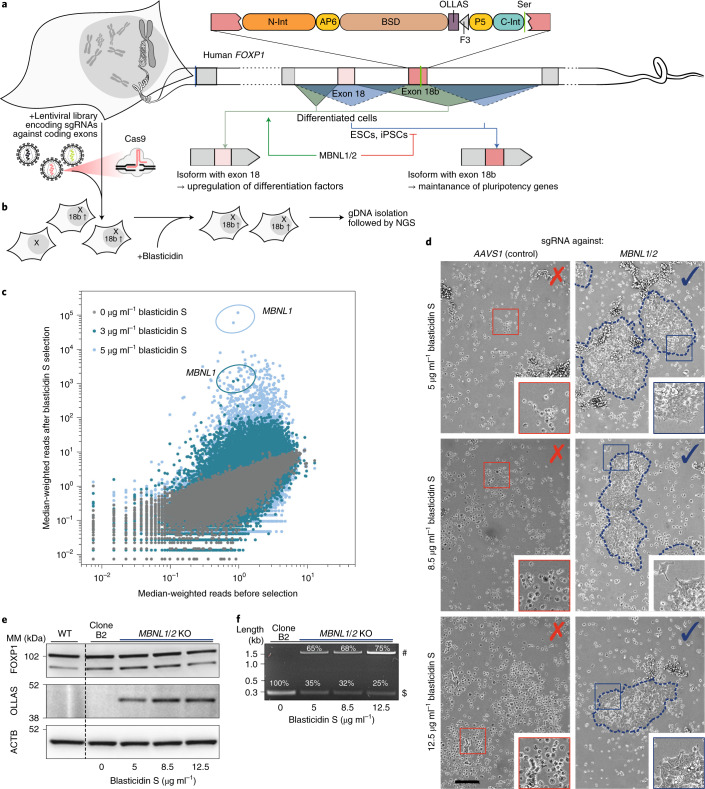
Scarless exon-dependent selection marker for the identification of regulators of exon inclusion and exclusion. **a**, Split–intein–CC-flanked BSD was integrated into *FOXP1* exon 18b of HEK293T cells using CRISPR–Cas9. ESCs, embryonic stem cells. **b**, A coding gene targeting a genome-wide lentiviral CRISPR–Cas9 library was applied and selected with blasticidin S to enrich cells with *FOXP1* exon 18b inclusion. NGS analysis was performed on cells with and without selection to identify the CRISPR–Cas9 spacer leading to cell survival. **c**, The median-weighted reads for all sgRNA targeting after selection with two concentrations of blasticidin S versus before selection. Two *MBNL1*-targeting sgRNAs highly enriched under both selection conditions are encircled. The assay was performed once with two different blasticidin S concentrations. **d**, The results of **b** were confirmed by an independent EXSISERS_*FOXP1*:18bBSD_ clone, transfected with a new set of *MBNL1*-targeting sgRNA together with an *MBNL2*-targeting sgRNA. Representative surviving colonies, selected with increasing blasticidin S concentrations (top to bottom) after co-transfection with *MBNL1* and *MBNL2* targeting CRISPR–Cas9 components. The safe-harbour *AAVS1* locus was targeted (AAVS1-KO control) as a control. Scale bar, 100 μm. Insets show 5× magnification **e**, Immunoblot analysis of the cells selected in **d**. **f**, RT–PCR analysis of the cells selected in **b**. For **e** and **f**, data represent an independent validation of the results of the screening shown in **c**. #, 18b EXSISERS_BSD_ isoform; $, 18b isoform. Source data are available online. Source data

As a further demonstration of the capabilities of EXSISERS, we next generated a variant that enables an unbiased library-based identification of regulators of isoform-specific expression by coupling cell viability to exon inclusion. We inserted blasticidin S deaminase^52^ (BSD) flanked by CC-enhanced inteins into exon 18b of the forkhead family transcription factor (*FOXP1*), which encodes the DNA-binding domain of an embryonic stem cell-specific FOXP1 isoform (Fig. 6a).

We next applied a lentiviral CRISPR–Cas9 knockout (KO) library^53^ to the EXSISERS_*FOXP1*:18bBSD_ HEK293T cells and selected blasticidin-S-resistant cells indicating exon 18b inclusion (Fig. 6b). Next-generation sequencing (NGS) analysis subsequently revealed a dose-dependent enrichment of sgRNAs targeting *MBNL1* (three or four magnitudes of enrichment over the median; Fig. 6c).

We verified the hits independently using a third sgRNA pair targeting *MBNL1* and *MBNL2* (not significantly enriched in the assay) on an independent EXSISERS_*FOXP1*:18bBSD_ clone controlled by a sgRNA targeting the non-essential *AAVS1* locus (*PPP1R12C*).

Titration of blasticidin S resulted in the formation of surviving cell colonies in only the population targeting *MBNL1* and *MBNL2* (Fig. 6d), indicating a functional coupling of the presence of *MBNL* splice factors to cell survival dependent on *FOXP1* exon 18b inclusion. Blasticidin-S-dependent enrichment of cells with exon 18b inclusion was confirmed by immunoblot detection of OLLAS-tagged BSD after KO of *MBNL1*/*2* (Fig. 6e) and using semi-quantitative RT–PCR (Fig. 6f). The integrity of FOXP1 in the transgenic 18b-tagged reporter line was verified for all conditions by immunoblot analysis, further confirming the traceless excision of BSD from the protein precursors (Fig. 6e).

**Extended Data Fig. 10:**
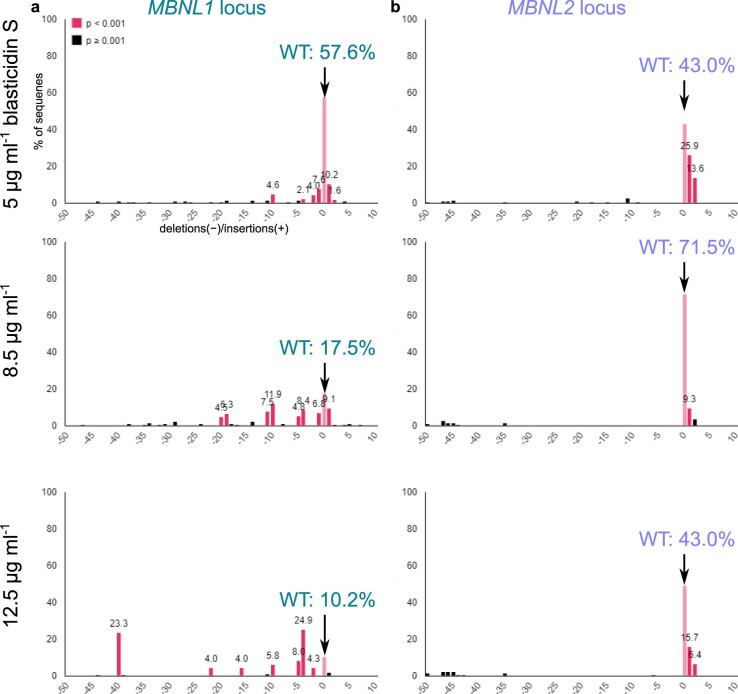
Genomic DNA analysis of cell populations selected with increasing blasticidin S concentration after MBNL1/2-KO. Genomic DNA was isolated from cell populations selected with blasticidin at three different concentrations (top row to bottom row). The frequency of insertions and deletions were estimated for **a**, the *MBNL1* or **b**, the *MBNL2* locus from Sanger sequencing chromatograms. Cells resistant to a blasticidin S in a concentration-dependent manner showed an increased frequency in mutated *MBNL1* alleles confirming the prominent role of MBNL1 in suppressing *FOXP1* exon 18b inclusion. This analysis was conducted once.

Detailed analysis by sequence decomposition^54^ revealed a dose-dependent enrichment of *MBNL1* indels. Only 10.2% of residual *MBNL1* WT allele was detectable in the most-stringent selection condition (12.5 µg ml^−1^ blasticidin S) and 57.6% WT allele in the less-stringent condition, whereas *MBNL2* indels showed no dose dependence (Extended Data Fig. 10). The correlation between *MBNL1* KO and *FOXP1* exon 18b inclusion successfully confirmed that MBNL1 is the major suppressor of exon 18b inclusion^55^.

**Fig. 7:**
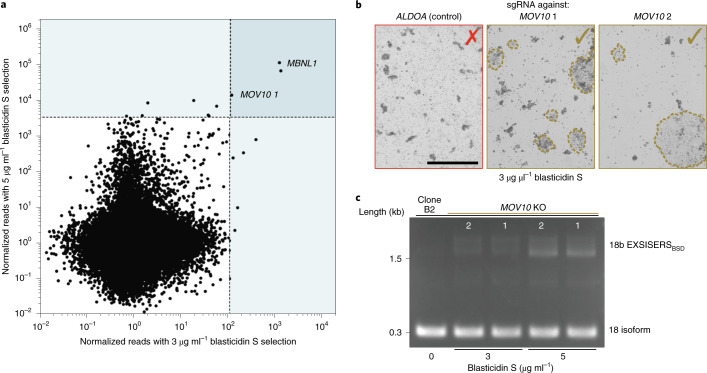
RNA helicase MOV10 is involved in *FOXP1* exon 18b suppression. **a**, The reads (normalized to preselection reads) from both selection conditions (3 µg ml^−1^ and 5 µg ml^−1^ blasticidin S). The areas highlighted in light blue indicate the most strongly enriched sgRNAs for each selection condition. These data are replotted from Fig. 6c. **b**, The enrichment of *MOV10* in the screen was confirmed by an independent *MOV10*-targeting sgRNA 2 (*MOV10* 2), which was not used in the screen. Representative colonies in a T75 flask are shown 2 weeks after selection with 3 µg ml^−1^ blasticidin S and after transfection with an sgRNA against *ALDOA* (unrelated control gene, <10 colonies in T75 flask), the MOV10-targeting sgRNA used in the screen (MOV*10* 1, >100 colonies in T75 flask), or MOV*10* 2 (>200 colonies in T75 flask). Scale bar, 1 mm. **c**, RT–PCR showing the blasticidin-S-concentration-dependent inclusion of *FOXP1* exon 18b from the colonies surviving blasticidin S selection shown in **b** labelled with the respective sgRNAs. The data represent an independent validation of the results shown in **a**. Source data

Besides *MBNL1*, only *MOV10* was substantially enriched in both blasticidin S selection conditions (Fig. 7a). *MOV10* encodes an RNA helicase that is required for microRNA-mediated biogenesis and gene silencing^56^ and the suppression of retroelements^57^, and is upregulated after differentiation of stem cells^58^. However, *MOV10* was not known to be involved in alternative splicing of stem-cell-associated genes, such as *FOXP1*.

To confirm that *MOV10* was indeed responsible for suppressing exon 18b inclusion, we cloned a different sgRNA, *MOV10*-targeting sgRNA 2 (MOV*10* 2), and compared it to *MOV10*-targeting sgRNA 1 (MOV*10* 1), which was enriched in the screen, and to an unrelated sgRNA targeting *ALDOA*. Indeed, only sgRNAs targeting *MOV10* caused a substantial growth of colonies two weeks after transient transfection plasmids targeting the corresponding genes (Fig. 7b). RT–PCR analysis of the pooled colonies revealed that indeed *FOXP1* exon 18b was included (Fig. 7c), but not as prominent as in the *MBNL* KO (Fig. 6f), indicating that *MOV10* has an auxiliary role in exon 18b repression.

Thus, we demonstrated that EXSISERS combined with a CRISPR library enables unbiased and non-invasive functional perturbation screening for splice modulators.

## Discussion

Here we developed a versatile exon-specific isoform expression reporter system (EXSISERS) that translates a specific exon expression event into reporter signals or genetically controlled handles for cell enrichment and selection. These features were made possible by combining fast-splicing inteins with CC domains to ensure the immediate removal of modular effector proteins and scarless translation of the unmodified, endogenous protein product. We applied EXSISERS to optimize RNA-targeting strategies for exon-specific RNA degradation or RNA-splicing modulation, as well as for identifying splicing regulators.

Specifically, we used a selection mechanism to identify splice regulators of *FOXP1* and confirmed that MBNL1 is the dominant suppressor of exon 18b inclusion^55,59^. We also identified that the RNA helicase *MOV10* is an auxiliary factor, which may reflect its possible role in improving the accessibility of splicing machinery.

Furthermore, we used EXSISERS to non-invasively quantify tau isoforms and showed that physiological and disease-relevant effects (increased 4R-tau level) could be read out conveniently using high-throughput-compatible ratiometric luciferase assays and longitudinal live monitoring with cellular resolution.

We demonstrated that the effects of a small-molecule inhibitor on the inclusion of exon 10 of *MAPT* could be longitudinally monitored in HEK293T cells, smNPCs and neurons generated from hiPSCs, demonstrating that EXSISERS enables high-throughput screening of 3R/4R regulation in relevant cellular models.

The precise ratiometric read-out of EXSISERS reporting the fractional 4R-tau expression (normalized to pan-tau) was instrumental for quantifying programmable RNA-targeting systems regarding their potency and isoform specificity.

A detailed comparison of their performance highlighted, in particular, the importance of optimizing crRNA design by targeting exon–exon junctions to spare pre-mRNA from Cas13d activity in the nucleus. Such a design is especially relevant for genes containing repetitive domains, common in genes encoding structural proteins. We also showed how isoform specificity can be increased by selectively targeting mature mRNA in the cytosol by Cas13b, which was superior to cytosolic Cas13d but still was exceeded in performance by optimized amiRNAs.

Furthermore, we showed that EXSISERS could non-invasively measure co-translational ribosomal frameshift regulation, an important mechanism that is not accessible using RNA-based quantification methods.

As EXSISERS monitors the translation of mature mRNAs, it is robust against changes in mRNA levels that do not translate to differences in isoform expression^60^. Thus, the reporter system could become uniquely informative to non-invasively assess exon-specific local protein translation as it occurs in neurons^9^. EXSISERS could also be expanded to build exon-dependent logic circuits in eukaryotic systems using intein-flanked recombinases, CRISPR RNA/DNA effectors, transcription factors or other effector domains without disturbing the host gene. We anticipate that the non-disruptive nature and cellular resolution of EXSISERS will also empower in vivo imaging studies in genetic model organisms to decipher the spatiotemporal patterns of exon-specific expression.

## Methods

### Molecular cloning

#### PCR for molecular cloning

Single-stranded primer deoxyribonucleotides were diluted to 100 μM in nuclease-free water (Integrated DNA Technology (IDT)). PCR reactions with plasmid and genomic DNA template were performed using the Q5 Hot Start High-Fidelity 2× Master Mix or with 5× High-Fidelity DNA Polymerase and 5× GC-enhancer (New England Biolabs (NEB)) according to the manufacturer’s protocol. Samples were purified by DNA agarose gel electrophoresis followed by purification using the Monarch DNA Gel Extraction Kit (NEB).

#### DNA digestion with restriction endonucleases

Samples were digested with NEB restriction enzymes according to the manufacturer’s protocol in a total volume of 40 μl with 2–3 μg of plasmid DNA. Next, fragments were gel-purified using DNA agarose gel electrophoresis and subsequently purified using the Monarch DNA Gel Extraction Kit (NEB).

#### Molecular cloning using DNA ligases and Gibson assembly

Agarose-gel purified DNA fragment concentrations were determined using a spectrophotometer (NanoDrop 1000, Thermo Fisher Scientific). Ligations were performed using 50–100 ng backbone DNA (DNA fragment containing the origin of replication) in a volume of 20 µl, with molar 1:1–3 backbone:insert ratios, using T4 DNA ligase (Quick Ligation Kit, NEB) at room temperature for 5–10 min. Gibson assemblies were performed using 75 ng backbone DNA in a 15 µl reaction volume and a molar 1:1–5 backbone:insert ratios, using the NEBuilder HiFi DNA Assembly Master Mix (2×) (NEB) for 20–60 min at 50 °C.

#### DNA agarose gel electrophoresis

Gels were prepared with 1% agarose (Agarose Standard, Carl Roth) in 1× TAE-buffer and 1:10,000 SYBR Safe stain (Thermo Fisher Scientific), running for 20–40 min at 120 V. For analysis, 1 kb Plus DNA Ladder (NEB) was used. The samples were mixed with gel loading dye (purple, 6×) (NEB).

#### Bacterial strains (*Escherichia coli*) for molecular cloning

Chemically and electrocompetent Turbo/Stable cells (NEB) were used for the transformation of circular plasmid DNA. For plasmid amplification, carbenicillin (Carl Roth) was used as a selection agent at a final concentration of 100 µg ml^−1^. All bacterial cells were incubated in lysogeny broth medium (LB) and on LB agar plates, including the respective antibiotics.

#### Bacterial transformation with plasmid DNA

For electroporation, either 1–5 µl ligation or Gibson reaction was dialyzed against MilliQ water for 10–20 min on an MF-Millipore membrane filter (Merck). Next, 1–5 µl dialysate was mixed with 50 µl of thawed, electrocompetent cells, transferred to a precooled electroporation cuvette (2 mm; Bio-Rad), shocked at 2.5 kV (Gene Pulser Xcell Electroporation Systems, Bio-Rad), and immediately mixed with 950 µl SOC-medium (NEB). The chemical transformation was performed by mixing 1–5 µl of ligation or Gibson reaction with 50 µl thawed, chemically competent cells and incubated on ice for 30 min. Cells were then heat-shocked at 42 °C for 30 s, further incubated on ice for 5 min and finally mixed with 950 µl SOC-medium (NEB). Transformed cells were then plated on agar plates containing an appropriate type of antibiotic and concentrations according to the supplier’s information. Plates were incubated overnight at 37 °C or for 48 h at room temperature.

#### Plasmid DNA purification and Sanger sequencing

Plasmid-DNA-transformed clones were picked and inoculated from agar plates in 2 ml LB medium with the appropriate antibiotics and incubated for about 6 h (NEB Turbo) or overnight (NEB Stable). Plasmid DNA intended for sequencing or molecular cloning was purified with QIAprep Plasmid MiniSpin (QIAGEN) according to the manufacturer’s protocol. Clones intended to be used in cell culture experiments were inoculated in 100 ml antibiotic medium containing the appropriate antibiotic and grown overnight at 37 °C. Plasmid DNA was purified using the Plasmid Maxi Kit (QIAGEN). Plasmids were sent for Sanger sequencing (GATC-Biotech) and analysed using Geneious Prime 2019 (Biomatters) sequence alignments.

### Lentiviral human CRISPR pooled genome-wide KO library

#### Amplification of the plasmid library

The human Brunello CRISPR knockout pooled library was a gift from D. Root and J. Doench (Addgene, 73178)^53^. In brief, the amplification of the plasmid DNA library was achieved by retransforming 100 µl ElectroMAX Stbl4 electrocompetent cells (Invitrogen, Thermo Fisher Scientific) with 400 ng of the plasmid library in four separate repetitions. For each repetition, the transformation was performed using 25 µl of cells and 100 ng of the library DNA with an ice-cold electroporation cuvette (0.1 cm gap size, Bio-Rad). Cells were shocked at 1.8 kV (preset settings, Ec1; Gene Pulser Xcell Electroporation Systems, Bio-Rad) and immediately mixed with 980 µl prewarmed (30 °C) SOC-medium (NEB). The electroporated cells in SOC-medium (4 ml in total) were combined in a ventilated falcon tube, and 6 ml of additional SOC-medium was added. Next, the 10 ml culture was shaken at 30 °C for 1 h, plated on 12 × 15 cm LB agar plates containing 100 µg ml^−1^ carbenicillin and incubated overnight for 16 h at 30 °C. The bacteria were next scraped from the plate using a cell scraper after adding 10 ml ice-cold LB medium per plate. The cell suspension was centrifuged (4 × 30 ml) at 4,000 relative centrifugal force (r.c.f.) for 10 min. The plasmid DNA library was extracted from the pellet using the Plasmid Maxi Kit (QIAGEN).

#### Production of the lentiviral genome-wide KO library

HEK293T cells were seeded in 4× T-225 flasks with 10 × 10^6^ cells per flask in 50 ml medium and incubated for 2 d until reaching 80% confluency. Before transfection, the medium was replaced with fresh medium containing heat-inactivated FBS (56 °C, 30 min). DNA (240 µg in total; 30 µg plasmid library DNA, 20 µg psPAX2 and 10 µg pMD2.G) in 24 ml Opti-MEM I Reduced Serum Medium (Gibco, Thermo Fisher Scientific) was mixed with 720 µl X-tremeGENE HP (Roche) and incubated at room temperature for 20 min. psPAX2 and pMD2.G were gifts from D. Trono (Addgene, 12260 and 12259). Next, this transfection mix was distributed dropwise into the four flasks with HEK293T cells at 80% confluency. Next, 24 h after transfection, the supernatant was collected and replaced with fresh medium containing heat-inactivated FBS. This procedure was repeated for the next 2 d, resulting in a total of 600 ml virus-containing supernatant. The supernatant was centrifuged at 500 r.c.f. for 10 min, and the resulting supernatant was filtered using a syringe filter unit (0.45 µm pore size, polyvinylidene difluoride (PVDF), 33 mm, Millex, Merck) to remove potential remaining cells. The flowthrough was concentrated 10× with Amicon centrifugal units (Ultra-15, PLHK Ultracel-PL Membrane, 100 kDa cut-off, Merck). The concentrated virus supernatant was then aliquoted into 2 ml tubes and frozen at −80 °C. To determine the multiplicity of infection (m.o.i.) per ml supernatant, serial dilutions of the supernatant were performed and combined with 10^6^ HEK293T cells in a total of 3 ml per well on six-well plates (medium with heat-inactivated FBS). Then, 24 h later, the medium was replaced with fresh medium containing 1 µg ml^−1^ puromycin (Gibco, Thermo Fisher Scientific). Next, 48 h after transduction, the wells were compared to the control well without virus transduction and without puromycin. The wells with survival rates of 10–80% were chosen to determine the m.o.i. per ml of the viral supernatant.

#### Lentiviral transduction of HEK293T EXSISERS_*FOXP1*:18bBSD_ with the pooled KO library

Cells (10^8^) in 500 ml medium with heat-inactivated FBS were combined with lentiviral supernatant at an m.o.i. of ~0.3, corresponding to a ~400× coverage of each sgRNA (library contains 76,441 unique sgRNAs) and were plated on 10× T-225 flasks. Next, 24 h after infection, puromycin was added to a final concentration of 1 µg ml^−1^. Then, 3 d after transduction, infected cells (cells surviving puromycin selection) were detached using 10 ml per flask Accutase solution (Gibco, Thermo Fisher Scientific). Cells were counted, and ~36 × 10^6^ cells were immediately frozen after pelleting at 500 r.c.f. for 10 min. The remaining cells were replated again on 12× T-225 flasks with 10 × 10^6^ cells per flask in 50 ml. Four flasks were cultured without antibiotics, whereas four flasks were selected with 3 µg ml^−1^ blasticidin S and another four flasks were selected using 5 µg ml^−1^ blasticidin S (Gibco, Thermo Fisher Scientific). After 2 weeks of selection (the condition without selection was collected 5 d after it reached 100% confluency), the surviving population of each condition was detached using Accutase, pooled and pelleted at 500 r.c.f. for 10 min. The cell pellets were kept at −20 °C until genomic DNA isolation.

#### NGS analysis of integrated lentiviral CRISPR library

Genomic DNA was isolated from the library-transduced HEK293T EXSISERS_*FOXP1*:18bBSD_ cells using the Wizard SV Genomic DNA Purification System (Promega) according to the manufacturer’s protocol. To amplify the CRISPR–Cas9 spacer sequence of the integrated lentivirus, the following barcoded primers with NGS adapters (underlined) were used, comprising an adapter sequence, the barcode (in upper case) and the binding region: BC1: ccatctcatccctgcgtgtctccgactcagCTAAGGTAACggctttatatatcttgtggaaaggacg; BC2: ccatctcatccctgcgtgtctccgactcagTAAGGAGAACggctttatatatcttgtggaaaggacg; BC3: ccatctcatccctgcgtgtctccgactcagAAGAGGATTCggctttatatatcttgtggaaaggacg; BC4: ccatctcatccctgcgtgtctccgactcagTACCAAGATCggctttatatatcttgtggaaaggacg; and a reverse primer composed of the trP1 adapter sequence (underlined) and the binding region: cctctctatgggcagtcggtgatctttcaagacctagctagcgaattc. A primer pair with BC3 was used to amplify genomic DNA from the transduced cells after puromycin selection but before blasticidin S selection. BC1, BC2 and BC4 were used to amplify the condition selected with 0 µg ml^−1^, 3 µg ml^−1^ and 5 µg ml^−1^ blasticidin S. NEBNext Ultra II Q5 Master Mix (NEB) was used to PCR-amplify the spacer sgRNA sequence from the proviral library according to the manufacturer’s protocol. The PCR products were agarose-gel-purified using the Monarch DNA Gel Extraction Kit (NEB). The barcoded PCR products were pooled equimolarly and submitted to the PrimBio Research Institute for NGS analysis. The FASTQ data were presorted according to their barcodes using the software ENCoRE^61^. The reads between different pools were normalized to the corresponding pool median before comparison.

### Mammalian cell culture

#### Cell lines and maintenance

HEK293T cells (ECACC: 12022001, Sigma-Aldrich) were maintained in an H_2_O-saturated atmosphere in Gibco Advanced DMEM (Thermo Fisher Scientific) supplemented with 10% FBS (Gibco, Thermo Fisher Scientific), GlutaMax (Gibco, Thermo Fisher Scientific) and penicillin–streptomycin (Gibco, Thermo Fisher Scientific) at 100 µg ml^−1^ at 37 °C and 5% CO_2_. Cells were passaged at 90% confluency by removing the medium, washing with DPBS (Gibco, Thermo Fisher Scientific) and separating the cells with 2.5 ml of Accutase solution (Gibco, Thermo Fisher Scientific). Cells were then incubated for 5–10 min at room temperature until a visible detachment of the cells was observed. Accutase was subsequently inactivated by adding 7.5 ml prewarmed DMEM, including 10% FBS and all of the supplements. Cells were then transferred into a new flask at an appropriate density or counted and plated in 96-well, 48-well or 6-well format for plasmid transfection.

#### hiPSC maintenance

hiPSCs (HPSI0614i-uilk_2 (ECACC: 77650606) and HPSI0514i-vuna_3 (ECACC: 77650602)) were invariantly incubated in Essential 8 Flex medium (Gibco, Thermo Fisher Scientific) at 37 °C under 5% CO_2_ saturation on (v/v) Geltrex-coated (A1413302, Gibco, Thermo Fisher Scientific) plates. At 70% confluency, the cells were subcultivated with StemMACS Passaging Solution XF (Miltenyi Biotec) by incubating the cells for 6 min at room temperature. Subsequently, StemMACS Passaging Solution XF was aspirated, and cells were collected by adding 1 ml Essential 8 Flex medium to the cells. Collected cells were transferred to a flask containing 2 ml Essential 8 Flex medium and seeded on 96-, 48-, 24- or 6-well plates at the appropriate cell densities.

#### smNPC conversion and maintenance

hiPSCs cultivated in Essential 8 Flex medium were converted into smNPCs by embryoid body formation according to a previously described protocol^62^. In brief, hiPSCs cultured in a six-well format were seeded into a six-well suspension culture plate using gentle cell dissociation reagent (7171, StemCell Technologies) with knockout serum replacement (KSR) medium, consisting of DMEM/F12 medium + GlutaMax ((3331-028, Gibco, Thermo Fisher Scientific), GlutaMax supplement (5 ml, I9278, Gibco, Thermo Fisher Scientific), 20% (v/v) KnockOut Serum Replacement (Gibco, Thermo Fisher Scientific), 1× MEM non-essential amino acids (Gibco, Thermo Fisher Scientific), 100 µM β-mercaptoethanol, (M6250, Sigma-Aldrich) and 1× GlutaMax supplement. KSR medium was supplemented with 1 µM dorsomorphine dihydrochloride (3093, Tocris Bioscience), 10 µM SB431542 (S1067, Selleck Chemicals), 3 µM CHIR99021 (4953, Tocris Bioscience), 0.5 µM purmorphamine (10009634, Cayman Chemicals) and 2 µM thiazovivin (420220, Merck Millipore). The next day, the embryoid bodies were transferred to a new six-well suspension cell culture plate with N2B27 medium, a 1:1 composition of N2 and B27 medium. N2 medium was composed of DMEM/F12 medium, 1× GlutaMax supplement, 1× N2 supplement (Gibco, Thermo Fisher Scientific), 5 µg ml^−1^ insulin, 1× MEM non-essential amino acids, 50 µM β-mercaptoethanol and 0.5× (50 U ml^−1^) penicillin–streptomycin (15140122, Gibco, Thermo Fisher Scientific). B27 medium was composed of neurobasal medium (15140122, Gibco, Thermo Fisher Scientific), 1× B27 supplement without vitamin A (12587010, Gibco, Thermo Fisher Scientific), 1× GlutaMax supplement and 0.5× (50 U ml^−1^) penicillin–streptomycin. N2B27 medium was supplemented with 1 µM dorsomorphine, 10 µM SB431542, 3 µM CHIR99021 and 0.5 µM purmorphamine. On day four, the medium was changed to expansion medium (N2B27 medium supplemented with 3 µM CHIR99021, 0.5 µM purmorphamine and 64 µg ml^−1^ l-ascorbic acid (A4544, Sigma-Aldrich)). Embryoid bodies were transferred to a 1% (v/v) Geltrex-coated plate on day 6. Thereafter, cells were passaged with Accutase (A6964, Sigma-Aldrich) when confluence was reached.

#### hiPSC-derived neurons

HiPSCs were plated on multiwell plates coated with 1% (v/v) Geltrex (A1413302, Gibco, Thermo Fisher Scientific) 24 h before neuronal initiation, maintained in Essential 8 medium (A2858501, Gibco, Thermo Fisher Scientific). For neuronal initiation, smNPC maintenance medium—a 1:1 composition of N2 and B27 medium supplemented with 100 ng µl^−1^ FGF8b (130-095-740, Miltenyi Biotec), 100 nM LDN193189 (130-103-925, Miltenyi Biotec) and 10 µM SB431542 (S1067, Selleck Chemicals)—was added to the cells. N2 medium was composed of DMEM/F12 + GlutaMax (10565018, Gibco, Thermo Fisher Scientific), 1× N2 supplement (17502048, Gibco, Thermo Fisher Scientific), 5 µg ml^−1^ insulin (I9278-5ML, Sigma-Aldrich), 100 µM MEM non-essential amino acids (11140035, Gibco, Thermo Fisher Scientific), 50 µM β-mercaptoethanol (M6250, Sigma-Aldrich) and 0.5× (50 U ml^−1^) penicillin–streptomycin (15140122, Gibco, Thermo Fisher Scientific). B27 medium was composed of neurobasal medium (21103049, Gibco, Thermo Fisher Scientific), 1× B27 supplement (11530536, Gibco, Thermo Fisher Scientific), 1× GlutaMax Supplement (35050038, Life Technologies) and 0.5× (50 U ml^−1^) penicillin–streptomycin. On day 14, the neuroepithelial sheet was replated onto a six-well plate, coated with 15 µg ml^−1^ poly-l-ornithine hydrobromide and 10 µg ml^−1^ laminin. On day 21, medium was aspirated, and cells were washed once with Dulbecco’s phosphate-buffered saline (DPBS) (14190169, Gibco, Thermo Fisher Scientific). Cells were detached using Accutase (6964, Sigma-Aldrich) under incubation for 10 min at 37 °C. The Accutase reaction was stopped by transferring the cells into a 15 ml Falcon containing 6 ml DMEM/F-12, GlutaMax. Cells were sedimented by centrifugation at 200 r.c.f. for 5 min, and cell pellets were resuspended in smNPC maintenance medium supplemented with 100 µM Y-27632 dihydrochloride (ALX-270-333-M005, Enzo Life Sciences) and counted using a Neubauer improved, precision disposable plastic hemocytometer C-chip (PK361, Carl Roth). Appropriate cell densities were seeded and cells were cultured in smNPC maintenance medium supplemented with 100 µM Y-27632 dihydrochloride for 24 h at 37 °C under invariant 5% CO_2_ saturation. The next day, the medium was aspirated and neuronal differentiation was initiated by adding neuronal induction medium—a 1:1 composition of N2 and B27 medium, supplemented with 200 µM ascorbic acid (10389701, Th. Geyer) and 20 ng ml^−1^ brain-derived neurotrophic factor (130-093-811, Miltenyi Biotec). In the case of detachment of the neuronal network, cells were reseeded onto new plates coated with 15 µg ml^−1^ poly-l-ornithine hydrobromide (P3655, Sigma-Aldrich) and 10 µg ml^−1^ laminin (L2020-1MG, Sigma-Aldrich). Neuronal networks were dissociated into single cells using Accutase. Cells were then carefully washed once with DPBS, and incubated with Accutase for 15 min at 37 °C under invariant 5% CO_2_ saturation. The Accutase reaction was stopped by transferring the cell solution into a 15 ml Falcon tube, containing 3 ml neuronal induction medium supplemented with 100 µM Y-27632 dihydrochloride. Cells were released from Y-27632 dihydrochloride by a medium change 24 h after reseeding.

#### Mycoplasma test

All of the cell lines were tested for mycoplasma contamination using MycoAlert Mycoplasma Detection Kit (LT07-318, Lonza). Furthermore, all of the cell lines were tested every 3 months for contamination by Hoechst 3334, which visualizes extranuclear speckles in case of contamination. All of the results shown in the manuscript were obtained from cells that tested negative for mycoplasma.

#### Plasmid transfection

Cells were transfected with X-tremeGENE HP (Roche) according to the manufacturer’s protocol. DNA amounts were kept constant in all of the transient experiments to yield reproducible complex formation and comparable results. In 96-well-plate experiments, a total amount of 100 ng of plasmid DNA was used; in 48-well-plate experiments, a total amount of 300 ng of plasmid DNA was used; and in 6-well-plate experiments, a total amount of 2.4 µg of plasmid DNA was used per well. Cells were plated 1 d before transfection (25,000 cells per well in 100 µl for 96-well plates, 75,000 cells per well in 500 µl for 48-well plates and 600,000 cells per well in 3 ml for 6-well plates). Then, for the 96-well transfections, 24 h after transfection, 100 µl fresh medium was added to each well and, 48 h after transfection, 100 µl medium was removed from each well and replaced with fresh medium.

#### Small-molecule manipulation of alternative splicing and polyamine-mediated frameshifting

For modulation of alternative splicing in HEK293T-derived EXSISERS lines with ITU (Sigma-Aldrich); 24 h after transfection, ITU (in DMSO) was applied to the cells. Control cells received the same volume of DMSO. For small-molecule neuronal precursor cells, 70,000 cells per cm^2^ were seeded in a 96-well format. Cells were maintained in N2B27 medium supplemented with 3 µM CHIR99021, 0.5 µM purmorphamine and 64 µg ml^−1^ l-ascorbic acid (A4544, Sigma-Aldrich). Modulation of alternative splicing by ITU (Sigma-Aldrich) was induced 2 d after seeding at 70% confluence. Cells were treated with ITU for 48 h (unless otherwise stated), with a complete medium change after 24 h before performing the Nano-Glo Dual-Luciferase Reporter Assay (N1610, Promega) according to the manufacturer’s protocol with a 5 s integration time. For the analysis of polyamine-mediated frameshift regulation of *Oaz1*, HEK293T cells were transfected in a 48-well plate with a plasmid harbouring the full-length *Oaz1* gene containing EXSISERS_mTagBFP2_ and EXSISERS_mNeonGreen_ inserted upstream and downstream of the regulatory hairpin with the in-frame stop codon. Cells were treated 24 h after transfection with the indicated polyamines and, 48 h after transfection, cells were detached using Accutase, pelleted (200 r.c.f. for 5 min) and resuspended in 200 µl of ice-cold DPBS containing 2% FBS for FACS analysis. FACS analysis was performed using the BD FACSaria II system (controlled by the BD FACSDiva software (v.6.1.3, BD Biosciences).

#### Generation of stable EXSISERS cell lines with CRISPR–Cas9

To generate a stable cell line (HEK293T, N2a), plasmids expressing a mammalian codon-optimized Cas9 from *Streptococcus pyogenes* (*Sp*Cas9) with a tandem C-terminal SV40 nuclear localization signal (SV40 NLS) (CBh hybrid RNA-polymerase II promoter-driven) and a single-guide-RNA (sgRNA/gRNA, human U6 RNA-polymerase III promoter-driven) with a 19–21 bp (*Sp*Cas9: *Tubb3* exon 2 (between Gly 81 and Ser 82): G+TGTGGAGCGGTACTCACAGG; *MAPT* exon 10: G+CCAGTCCAAGTGTGGCTCAA; *MAPT* exon 11: GTTGCCTAATGAGCCACACT; *FOXP1* exon 18b: AGGATGAGTTTGGGTCCTTT) cloned spacer targeting the EOI were used. The insertion site for EXSISERS was always upstream of serine, cysteine or threonine due to the downstream extein requirements of inteins. We carefully avoided insertion sites close to prolines and tried to choose insertion sites resembling the native extein environment to maximize splicing efficiency^63^. Furthermore, we used NetGene2 (v.2.42)^64^ and Human Splice Finder (v.3.1)^65^ to verify that our insertion did not destroy a potential regulatory sequence or introduced cryptic splice sites. The efficiency of CRISPR–Cas9 for a target site was determined using a T7 endonuclease I assay (NEB) according to the manufacturer’s protocol 48–72 h after transfecting cells with plasmids encoding Cas9 and the targeting sgRNA on a 48-well plate. Optionally, an i53 expression plasmid (a genetically encoded 53bp1 inhibitor^66^) was co-transfected to enhance homologous recombination after the Cas9-mediated double-strand break at the spacer-guided genomic site. The donor DNA plasmid contains the intein-flanked moiety, including the selection cassette to select for cells undergoing successful Cas9-mediated homologous recombination; moreover, the donor DNA plasmid contains homology arms of at least 800 bp flanking the intein–reporter construct. Then, 48 h after transfection (48-well or 6-well format), the medium was replaced with medium containing 50 µg ml^−1^ puromycin, if not otherwise indicated. Cells were observed daily and were detached with Accutase and replated with puromycin when surviving colonies reached a colony size of about 50 cells. This step was repeated until no significant puromycin-mediated cell death could be observed. Those cells were plated without puromycin on a 48-well plate and were transfected with a CAG-hybrid promoter-driven nuclear-localized Cre or Flp recombinase with a low amount of a green fluorescent protein (Xpa-H62Q^67^) at a 10:1 ratio to excise the selection cassette. The green fluorescent protein was co-transfected to enrich cells that were successfully co-transfected with the recombinase-expressing plasmid. Green cells were enriched using the BD FACSaria II system (controlled with the BD FACSDiva Software (v.6.1.3, BD Biosciences)) and replated onto a suitable dish/plate. After one week, enriched cells were single-cell-sorted in 96-well plates and grown monoclonally until the colony size was large enough to be duplicated onto a second 96-well plate containing 2 µg ml^−1^ puromycin. Cells that underwent successful cassette excision should not survive puromycin treatment, indicating that the original clone from which it was duplicated did not anymore contain the puromycin-*N*-acetyltransferase and was a potential candidate for genotyping for zygosity. Those clones were detached and expanded in 48-well plates until confluency, and half of the cell mass was then used subsequently for isolation of genomic DNA using the Wizard Genomic DNA Purification Kit (Promega). Genotyping of the genomic DNA was performed using LongAmp Hot Start Taq 2× Master Mix (NEB) according to the manufacturer’s protocol with primer deoxynucleotides pairs (IDT) with at least one primer binding outside of the homology arms. The PCR products from clones, for which genotyping indicated homozygosity, were sent for Sanger sequencing to verify their sequence integrity. Exemplary genotyping results for EXSISERS_*MAPT*:10Halo_ revealed that all of seven randomly picked clones were EXSISERS-positive for at least one allele (three homozygous, three heterozygous, one hemizygous with one deletion allele; Supplementary Fig. 12). Homozygous targeting is not required in general, but we nevertheless chose homozygous clones to better confirm minimal invasiveness without confounds from WT alleles, for example, for immunoblot results.

#### Generation of a stable human iPSC EXSISER line

The hiPSCs (HPSI0514i-vuna_3, HPSI0614i-uilk_2) were electroporated with a sgRNA-Cas9-i53 expressing vector and a respective donor vector carrying EXSISERS_*MAPT*:10NLuc_ or EXSISERS_*MAPT*:11FLuc_, including the selection cassette to select for cells undergoing successful Cas9-induced homology-directed-repair-mediated insertion. The sgRNA-Cas9-i53 vector expresses a mammalian codon-optimized Cas9 from *S. pyogenes* (*Sp*Cas9) with a tandem C-terminal SV40 nuclear localization signal (SV40 NLS), driven by a CBh hybrid-polymerase II promotor, i53, a genetically encoded 53bp1 inhibitor, and a single guide sgRNA/gRNA driven by a human U6 RNA-Polymerase III promotor with a 21-bp and 20-bp cloned spacer targeting the EOI, respectively: *MAPT* exon 10: G+CCAGTGGAAGTGTGGCTCAA; *MAPT* exon 11: GTTGCCTAATGAGCCACACT. Furthermore, homology arms of 1.5 kb and 3.0 kb flank the intein–reporter construct. hiPSCs were electroporated as single cells in a 100 µl cuvette format, using the program CB-150 of the P3 Primary Cell 4D-Nucleofector X Kit (Lonza Group) and 4D Nucleofector X Unit, according to the manufacturer’s protocol. Cells were incubated in Essential 8 Flex medium supplemented with 10 µM Y27632 and dihydrochloride (ALX-270-333-M005, Enzo Life Sciences) for 24 h after nucleofection. Cells were left to recover with a medium change every other day. Puromycin selection was applied to cells at 70% confluence for 3 d by supplementing Essential 8 Flex medium with 1 µg ml^−1^ puromycin (Gibco, Thermo Fisher Scientific). Selection pressure was removed after 3 d, entering a second recovery phase. Subsequently, cells were monoclonalized by colony separation. Separated clones were seeded in a 24-well format and expanded for genotyping and subsequent excision of the puromycin selection cassette by Flp and Cre Recombinase, respectively. The excision of the selection cassette was verified using a puromycin sensitivity test. Clones that exhibited puromycin sensitivity were used for further targeting and smNPC conversion. Genomic DNA was isolated from cells from a six-well format at 70–80% confluency using the QIAamp DNA Mini Kit (Qiagen). A triple PCR for genotyping was performed using the Q5 Polymerase Hot Start 2× Master Mix (NEB) according to the manufacturer’s protocol. Genotyping for EXSISERS_*MAPT*:10NLuc_ was performed using primer pairs that bind upstream of the 5′ homology arm (5′-CTACCAAGTATAGGTATACAGG-3′) and either inside the 3′ homology arm (5′ -CTACATTCACCCAGAGGTC-3′) or the EXSISERS_*MAPT*:10NLuc_ insertion (5′ -CGAAGTAGTCGATCATGTTTG-3′). Genotyping for EXSISERS_*MAPT*:11FLuc_ was performed using primer pairs that bind upstream of the 5′ homology arm (5′ -CATCCACTCCTCTCCTTTC-3′), and either inside the 3′ homology arm (5′ -CTTTCCAGCCCCTTCTGAAG-3′) or the EXSISERS_*MAPT*:11FLuc_ insertion (5′-GCTTGAAGTCGTACTCGTTG-3′). PCR products of the genotyping PCR from homozygous clones were cut out, purified and verified using Sanger sequencing. The normal karyotype of gene-edited hiPSCs was verified by G-banding (Supplementary Fig. 13a), performed in collaboration with the Institute of Human Genetics, Klinikum rechts der Isar of Technical University of Munich. Verification of the pluripotency of edited cell lines was performed using immunofluorescence staining for the pluripotency markers SOX2, OCT4A and NANOG, using the primary antibodies C70B1 rabbit anti-SOX2 (1:500, 3728S, Cell Signaling Technology), C30A3 rabbit anti-OCT4A (1:500, 2840, Cell Signaling Technology) and goat anti-NANOG (1:500, AF1997, R&D Systems), and the secondary antibodies Alexa Fluor 488-conjugated cross-absorbed goat-anti-rabbit IgG (H+L) (1:500, A11008, Thermo Fisher Scientific) and Alexa Fluor 594-conjugated cross-absorbed donkey-anti-goat IgG (H+L) (1:500, A11058, Thermo Fisher Scientific) (Supplementary Fig. 13b), and by the differentiation of the edited cells into all three germ layers using the STEMdiff Trilineage Differentiation Kit (StemCell Technologies).

#### Gene expression manipulation using CRISPR–Cas9

Gene expression of *MAPT* was induced in HEK293T cells by co-transfecting CAG-driven mammalian-codon optimized nuclease-defective *S. pyogenes* Cas9 (D10A, H840A) fused to a tripartite trans-activation domain and SV40 NLS^31^ and a plasmid mix expressing (human U6 promoter) three spacer-truncated sgRNAs (14–15-nucleotide truncated spacer instead of 19–21 nucleotides)^68,69^ targeting the 5′ upstream region of the *MAPT* transcription start site: G+GAGGGCAGCGCCGAG, GGAGAAGGCTCCCG and GCGAGCCTCCCCAG. An uninduced control was co-transfected with an empty sgRNA cloning plasmid instead.

#### Verification of cellular identities (hiPSCs and smNPCs) using immunofluorescence detection

hiPSCs, smNPCs, germ layer identities and hiPSC-derived neurons were confirmed using immunocytochemistry. C70B1 rabbit anti-SOX2 (1:500, 3728S, Cell Signaling Technology), C30A3 rabbit anti-OCT4A (1:500, 2840, Cell Signaling Technology) and goat anti-NANOG (1:500, AF1997, R&D Systems) antibodies were used to stain the pluripotency markers. Germ-layer-specific markers were stained using goat anti-SOX17 (1:1,000, AF1924, R&D Systems) and P87H4B7 mouse anti-FOXA2 (1:1,000, 685802, BioLegend) antibodies for endoderm; AD2.38 mouse anti-PAX6 (1:200, ab78545, Abcam) and 10C2 mouse anti-nestin (1:250, MA1-110, Thermo Fisher Scientific) antibodies for ectoderm; and EPR18113 rabbit anti-TBXT (1:1,000, ab209665, Abcam) and rabbit anti-NCAM1 (1:200, ab204446, Abcam) antibodies for mesoderm. smNPC identity verification was based on smNPC-specific markers, stained using the following antibodies: AD2.38 mouse anti-PAX6 (1:500, ab78545, Abcam), 10C2 mouse anti-nestin (1:500, MA1-110, Thermo Fisher Scientific), C70B1 rabbit anti-SOX2 (1:500, 3728S, Cell Signaling Technology) and SDL.3D10 mouse anti-TUBB3 (1:500, T5076-200UL, Sigma-Aldrich); and, for negative markers, C30A3 rabbit anti-OCT4A (1:500, 2840, Cell Signaling Technology) and EPR4007 rabbit anti-SOX10 (1:250, ab155279, Abcam). hiPSC-derived neurons were confirmed using SDL.3D10 mouse anti-TUBB3 (1:250, T5076-200UL, Sigma-Aldrich) and rabbit anti-MAP2 (1:250, AB5622, Merck Millipore) antibodies. The secondary antibodies used were Alexa-Fluor-488-conjugated cross-absorbed goat-anti-rabbit IgG (H+L) (1:500, A11008, Thermo Fisher Scientific), Alexa-Fluor-594-conjugated cross-absorbed donkey-anti-goat IgG (H+L) (1:500, A11058, Thermo Fisher Scientific) and Alexa-Fluor-594-conjugated cross-absorbed donkey anti-mouse IgG (H+L) (A21203, Thermo Fisher Scientific). In brief, hiPSCs and smNPCs were seeded at an appropriate density on 1% Geltrex-coated (Gibco, Thermo Fisher Scientific) glass slides in a 24-well plate format, and hiPSC-derived neurons on glass slides coated in 15 µg ml^−1^ poly-l-ornithine hydrobromide (P3655, Sigma-Aldrich) and 10 µg ml^−1^ laminin (L2020-1MG, Sigma-Aldrich) in a 24-well plate format. Fixation by 10% formalin was conducted at a stage of 80% cell confluence. Cells were prefixed for 30 min at 37 °C and 5% CO_2_ saturation by adding 200 µl 10% formalin to wells still containing 500 µl growth medium. Subsequently, the medium was aspirated, and cells were fixed with 10% formalin for 30 min at 37 °C and 5% CO_2_ saturation, followed by three DPBS washing steps. Cells were incubated in 0.5% Triton X-100, 1% FBS diluted in PBS for 10 min at room temperature to improve antibody penetration. The primary antibody was added to cells in 0.5% Triton X-100 and 1% FBS diluted in PBS, and incubated for 2 h at room temperature or overnight at 4 °C. Next, the cells were washed three times with DPBS before incubation with the appropriate secondary antibodies for 1 h at room temperature using Alexa-Fluor-594-conjugated cross-absorbed donkey anti-mouse IgG (H+L) (1:500, A21203, Thermo Fisher Scientific), Alexa-Fluor-488-conjugated cross-absorbed goat-anti-rabbit IgG (H+L) (1:500, A11008, Thermo Fisher Scientific) and Alexa Fluor 488-conjugated cross-absorbed donkey anti-goat IgG (H+L) (1:500, A11055, Thermo Fisher Scientific). Nuclei were visualized using 4′,6-diamidino-2-phenylindole. Imaging was performed using epifluorescence microscopy (ZEISS Axiovert 200 M equipped with AxioCam HRc camera, acquisition was performed using Axiovision v.4.6 (Zeiss)) with the corresponding filter sets. Representative immunocytochemistry images for EXSISERS_*MAPT*:10NLuc-11FLuc_ smNPCs are shown (Supplementary Fig. 13c). Representative immunocytochemistry images for EXSISERS_*MAPT*:10NLuc-11FLuc_ hiPSC-derived neurons (WT and IVS10+16 C>T) are shown in Supplementary Fig. 14.

#### mRNA manipulation using CRISPR–Cas13

CAG-driven mammalian codon-optimized *Rf*xCas13d^42^ with a C-terminal triple NLS (SV40 NLS + MYC NLS + synthetic NLS) or *P*spCas13b^43^ with a C-terminal nuclear export signal from HIV Rev protein were co-transfected with a plasmid encoding the crRNA of the Cas13 system (human U6 RNA polymerase III driven) targeting the RNA of interest indicated in the figures. All constructs were generated using oligodeoxynucleotides (IDT) and gene fragments (gBlocks, IDT).

#### mRNA manipulation with amiRNAs

CAG-driven mammalian codon-optimized iRFP720 was intersected with a modified intron derived from rabbit beta-globin. Within the synthetic intron, the artificial mir-30-based synthetic microRNA backbone containing the critical region for efficient microRNA biogenesis and a type-IIS restriction enzyme cloning site was embedded^45^. Guide and passenger (star) strands were designed using splashRNA^46^ and cloned into the intron-embedded microRNA backbone with type-IIS restriction enzymes. All constructs were generated using oligodeoxynucleotides (IDT) and gene fragments (gBlocks, IDT).

#### KO of *MBNL1*/*MBNL2* and *MOV10* using CRISPR–Cas9

To knockout *MBNL1/2* or *MOV10* in HEK293T cells, which carry a blasticidin resistance gene flanked by inteins within the *FOXP1* exon 18b, plasmids expressing a mammalian codon-optimized *Sp*Cas9–NLS and sgRNAs targeting *MBNL1* (G+TGGTGCCCCATTACAACCCG) and *MBNL2* (G+TCAACCTGACAACTTTTGGG) in parallel, or *MOV10* (1: G+CCGGAGCCTTTGACAGAGT; 2: GCCCGTAACAATGTGCCTCA) were co-transfected. Then, 72 h later, cells were replated in a proper format, and media were supplemented with the indicated blasticidin S concentrations. The control sample was transfected under the same conditions, but the sgRNA targeted the control locus *AAVS1* (*PPP1R12C*: GGGACCACCTTATATTCCCA) or *ALDOA* (G+AAACTTCTGAGTGCAAGCA). Genomic DNA was isolated from blasticidin-S-treated surviving colonies using the Wizard Genomic DNA Purification Kit (Promega) according to the manufacturer’s protocol.

### Protein biochemical analysis

#### Immunoblot analysis

Cells were lysed with the appropriate volume of M-PER (Thermo Fisher Scientific), including protease inhibitors (Halt Protease Inhibitor Cocktail, Thermo Fisher Scientific), according to the manufacturer’s protocol. Cleared lysates were then equalized against the relative protein concentration determined using the NanoDrop 1000 (Thermo Fisher Scientific) and diluted with M-PER. If indicated, samples were also dephosphorylated at 30 °C for 1 h using λ (Lambda) protein phosphatase (NEB) according to the manufacturer’s protocol. Equalized lysates were prepared for SDS–gel-electrophoresis using XT sample buffer (Bio-Rad) and XT reducing agent (Bio-Rad) and denatured at 70 °C for 10 min or 95 °C for 5 min. Samples were loaded in 18-well 4–12% Criterion XT Bis-Tris Protein Gel, and electrophoresis was run at 150 V for 1.5 h in XT MOPS running buffer (Bio-Rad). Molecular mass markers (Amersham ECL Full-Range Rainbow Molecular Weight Markers, GE Healthcare Life Sciences, General Electric) and tau-ladder (T7951, Sigma-Aldrich) were used in parallel to confirm the different protein species. Subsequently, an immunoblot was performed onto an Immobilon-P PVDF membrane (Merck) with a wet blotting system (Criterion Blotter, Bio-Rad) in ice-cold Towbin buffer (Bio-Rad) with 20% (v/v) methanol (Carl Roth) overnight (15 V at 4 °C). Next, the free valences on the PVDF membrane were blocked in blocking buffer containing 5% (m/v) skimmed milk (Carl Roth) in TBS-T (pH 7.6) with 0.1% (v/v) Tween-20 (Sigma-Aldrich) at room temperature for 1 h. Antibodies were diluted at the indicated dilution factors in blocking buffer and either incubated at room temperature for 2 h or overnight at 4 °C, followed by at least three washing steps (room temperature, 5 min, 60 r.p.m.). The HRP-conjugated secondary antibody (Abcam) was also diluted in blocking buffer (1:20,000) and subsequently washed again with TBS-T at least four times. HRP detection was performed using the SuperSignal West Femto Maximum Sensitivity Substrate or SuperSignal West Atto Ultimate Sensitivity Substrate (Thermo Fisher Scientific) on the Fusion FX7/SL advanced imaging system using FusionCapt Advance SL4 v.16.09b (Vilber Lourmat). The primary and secondary antibodies used were as follows: M2 mouse anti-Flag (1:1,000, F1804-200UG, Sigma-Aldrich), L2 rat anti-OLLAS (1:1,000, MA5-16125, Thermo Fisher Scientific), PC1C6 mouse anti-pan-tau (1:200, MAB3420, Merck Millipore), 8E6/C11 mouse anti-3R-tau (1:1,000, 05-803, Merck Millipore), EPR4114 rat anti-FOXP1 (1:1,000, ab134063, Abcam), 32F6 mouse anti-mNeonGreen (1:1,000, 32f6-100, ChromoTek), ab21176 rabbit anti-firefly luciferase (1:1,000, Abcam), D71G9 rabbit anti-TUBB3 (1:1,000, 5568S, Cell Signaling Technology), AC-15 mouse anti-beta-actin (HRP-coupled, 1:100,000, ab6276, Abcam), goat anti-mouse IgG H&L (HRP-coupled, 1:20,000, ab97023, Abcam), goat anti-rat IgG H&L (HRP-coupled, 1:20,000, ab97057, Abcam) and goat anti-rabbit IgG H&L (HRP-coupled, 1:20,000, ab6721, Abcam). Quantification was performed using Image Lab (v.6.1.0 build 7, Bio-Rad). All uncropped immunoblots are shown in Supplementary Fig. 15.

#### DNA analysis

To quantify the genome editing outcome of *MBNL1*/*MBNL2*, the intended genomic double-stranded break region was PCR-amplified using the LongAmp Hot Start Taq 2× Master Mix (NEB) according to the manufacturer’s protocol with primer deoxynucleotides pairs (IDT) flanking the expected mutation region. After DNA agarose gel electrophoresis, the PCR fragments were cut out and purified using the Monarch DNA Gel Extraction Kit (NEB). Indel analysis was performed using TIDE^54^.

### RNA analysis

#### Semi-quantitative RT–PCR

Cells were collected for 5 min at 200*g*, and the RNA was isolated using the RNeasy Mini Kit (QIAGEN) according to the manufacturer’s protocol. Reverse transcription was performed using the SuperScript IV VILO Master Mix (Invitrogen, Thermo Fisher Scientific) according to the manufacturer’s protocol, followed by a semi-quantitative PCR under non-saturating conditions using Q5 polymerase (NEB). The following exon-spanning primers were used for semi-quantitative quantification of *FOXP1* exon 18 or 18b inclusion: RT_FOXP1-Ex16-17_1_fw, TCTTTAATTAGGCAGGCCATTCTCG; RT_FOXP1_Ex19-20_1_rv, CCATTGAAGCCTGTAAAGCTGC.

#### RT–qPCR

The Luna Cell Ready Probe One-Step RT–qPCR Kit (NEB) was used according to the manufacturer’s protocol with slight modifications. Reactions were run in 384-well plates (10 μl per well) in technical duplicates. 20× TaqMan Gene Expression Assays (Thermo Fisher Scientific) for *ACTB*, pan-tau and 4R-tau were used at half concentration (450 nM final per primer and 125 nM final per probe; *ACTB*, Hs01060665_g1; pan-tau, Hs00902194_m1; 4R-tau, Hs00902312_m1). The reaction was performed and monitored using the Applied Biosystems QuantStudio 12K Flex Real-Time PCR system (v.1.4). The acquired data were processed and exported using QuantStudio 12K Flex Software. For some data points, individual baseline correction was performed. The relative *MAPT* mRNA levels of pan-tau- and 4R-tau isoforms were obtained by applying the ${2^{{-\Delta\Delta}{C}_{T}}}$ method; *ACTB* was used as an internal control. All data were normalized to the *MAPT-*induced but untreated control sample, similar to the *MAPT* luciferase assays.

### Cellular and molecular imaging

#### Immunocytochemistry

The medium was aspirated from the cells, and the cells were washed with DPBS (Gibco, Thermo Fisher Scientific) and fixed for 15 min in 10% neutral buffered formalin (Sigma-Aldrich) at room temperature. Primary antibodies at the indicated concentration were diluted 1:1,000 in BSA blocking buffer (only anti-pan-tau antibodies (PC1C6, Merck) were diluted 1:200). Blocking buffer was prepared using DPBS (Gibco, Thermo Fisher Scientific) with 1% BSA (Sigma-Aldrich) containing 0.5% Triton X-100 (Sigma-Aldrich). Cells were washed three times after fixation with DPBS (Gibco, Thermo Fisher Scientific) for 5 min at room temperature, and blocking buffer containing the suitable fluorescent dye coupled secondary antibodies (1:1,000, Thermo Fisher Scientific) was applied for 2 h at room temperature or overnight at 4 °C. The primary and secondary antibodies that we used were as follows: mouse PC1C6 anti-pan-tau (Merck), rabbit D71G9 anti-TUBB3 (Cell Signaling Technology, Cy3-conjugated cross-adsorbed goat anti-mouse IgG (H+L) (Thermo Fisher Scientific), Cy5-conjugated cross-adsorbed goat anti-mouse IgG (H+L) (Thermo Fisher Scientific) and Alexa-Fluor-633-conjugated cross-adsorbed goat anti-rabbit IgG (H+L) (Thermo Fisher Scientific).

#### Epifluorescence microscopy

Epifluorescence microscopy was performed using the EVOS FL Auto Cell Imaging System (Invitrogen, Thermo Fisher Scientific) under identical conditions for all samples across conditions.

#### Confocal microscopy

Confocal microscopy was conducted on a Leica SP5 system (Leica Microsystems). For the life-imaging of cells, warm phenol-red-free DMEM/F12 medium supplemented with HEPES (Gibco, Thermo Fisher Scientific) and 10% FBS was used, and imaging was performed at 37 °C under 5% CO_2_.

#### Bioluminescence microscopy

Bioluminescence life-imaging was performed using the LV200 bioluminescence imaging system (Olympus) in 8-well µ-slides (Ibidi). Transfection conditions of cells on 8-well µ-slides (Ibidi) were identical to 48-well plates. As a substrate solution for NLuc, the Nano-Glo Live Cell Assay System (Promega) was used according to the manufacturer’s protocol. Images were analysed using Fiji ImageJ v.1.52p.

#### Bioluminescence quantification

For bioluminescence bulk quantifications, cells were plated and transfected in 96-well format. All bioluminescence qualifications were performed using the Centro LB 960 (Berthold Technologies) plate reader with the indicated acquisition time using MikroWin 2000 (v.4.34). For bioluminescence detection of secreted/shedded NLuc, the supernatant was sampled (10 µl) at the indicated time points and detected using the Nano-Glo Luciferase Assay System (Promega) with a 0.1 s acquisition time. For dual-luciferase read-out using the Nano-Glo Dual-Luciferase Reporter Assay System (Promega), NLuc and FLuc signals were read out on-plate 72 h after transfection with a 0.5 s acquisition time (5 s for smNPCs) after 10 min addition of reagent 1 (ONE-Glo EX Luciferase) for FLuc and after 20 min addition of reagent 2 (NanoDLR Stop & Glo) for NLuc, which includes a FLuc inhibitor. For each of the paired NLuc and FLuc measurements from each individual sample, the ratio was taken, and the mean and s.d. were calculated across biological replicates. To keep the *y*-axis range consistent across experiments, the RLU for the reference condition (*MAPT* induction without further perturbation) was set to 1. For live-cell NLuc and FLuc quantification, Nano-Glo Endurazine (Promega) Life Cell Substrates (1:200) and 500 µM VivoGlo d-luciferin (Promega) were used for a maximum duration of 65 h diluted in medium. The direct effects of ITU on FLuc or NLuc were excluded by applying ITU to cells after lysis at the same concentrations.

#### Design considerations of EXSISERS constructs

A detailed step-by-step protocol on generating constructs and cell lines for EXSISERS is available at the Protocol Exchange^25^.

All of the constructs were generated using gene fragments (gBlocks) and oligodeoxynucleotides (IDT) and were codon-optimized for mammalian expression. A list of the relevant nucleotide and amino acid sequences used in this study is provided in Supplementary Table 2.

EXSISERS_TMD-HaloTag_: the type-II TMD acts as a start-transfer sequence and translocates the subsequent binding moiety to the luminal/extracellular space, whereas the type-I TMD stops the translocation process; the sequence before and after the type II and type I TMD is therefore located in the cytosolic compartment, and the sequence that is between the TMDs is consequently in the extracytosolic compartments (Extended Data Fig. 8b). Two mutations (C61V and C262A) were also introduced into the HaloTag domain to prevent the accidental formation of disulfide bonds in the oxidative extracytosolic compartments and therefore might induce misfolding as previously shown for many fluorescent proteins^70^. We selected the mouse Fcer2 membrane-spanning region as the type II TMD and also adopted the flanking amino acids as the N-terminally positively charged amino acids on the N-terminal (cytoplasmic) site to ensure proper domain topology (positive-inside rule) and two TMD-preceding palmitoylatable cysteines might also improve membrane association and topology. We chose the human GYPA as the prototypical type-I TMD owing to its positively charged amino acids C-terminally (cytosolic site) adjacent to the TMD. Furthermore, G102L was introduced to disrupt the GxxxG TMD-dimerization motif, and a plasma membrane trafficking signal (PMTS) was also added^71^.

EXSISERS_NLuc/FLuc_: we chose to flank NLuc with P5/AP6 CCs and a gp41-1 split–intein pair, whereas EXSISERS_FLuc_ was created by flanking a quintuple thermostable mutant of firefly luciferase (F14R, L35Q, V182K, I232K, F465R)^72^ with another set of CCs (P3/AP4) and an NrdJ-1 split–intein pair.

EXSISERS_BSD_: BSD was flanked with P5/AP6 CCs and a gp41-1 split–intein pair was used in analogy to the aforementioned constructs.

#### General design and application considerations for EXSISERS constructs

We suggest avoiding insertion sites close to prolines and insertion sites resembling the native extein environment to maximize splicing efficiency^63^. We also recommend using the following inteins: gp41-1, NrdJ-1, IMPDH-1 and HwarPolA^27,63^. Moreover, one should check the regularly updated intein database, which contains more than 1,000 inteins with known native extein sequences (maintained by the Iwaï laboratory, (InBase v.2.0) https://inteins.biocenter.helsinki.fi/index.php). The database enables searches for inteins with a desired native extein sequence to maximize the splicing efficiency. Furthermore, one should check with NetGene2 (v.2.42)^64^ and Human Splice Finder (v.3.1)^65^ whether the insertion might destroy a potential regulatory sequence or might cause the introduction of cryptic splice sites. A sufficient CRISPR–Cas9 efficiency for a target site should be confirmed by a T7 endonuclease I assay (NEB) according to the manufacturer’s protocol 48–72 h after transfection of cells with plasmids encoding Cas9 and the targeting sgRNA on a 48-well plate. Finally, an immunoblot analysis with suitable antibodies should be performed to ensure sufficient protein splicing efficiency. For screening applications, potential hits should be cross-checked with respect to whether the effect might result from direct modulation of FLuc or NLuc.

### Statistics and reproducibility

Statistics were calculated using PRISM (v.8, GraphPad) as specified in each figure. The RLUs of NLuc and FLuc were normalized to the respective reference condition (for example, *MAPT* induction without perturbation) to aid graphical analysis. The ratio (NLuc/FLuc) was computed for each of the paired NLuc and FLuc measurements from each individual sample, and the mean and s.d. were calculated across the ratios from biological replicates.

No statistical method was used to predetermine sample size. The experiments were not randomized. The investigators were not blinded to allocation during experiments and outcome assessment. However, if possible, the experiments were performed with master mixes and multichannel pipettes to exclude unconscious biases during sample preparation.

No data were excluded from the analyses except for the experiments with hiPSCs shown in Fig. 3b,c, for which some technical replicates had to be excluded from calculating the mean value due to obvious technical errors (values shown in italics in the Source Data).

Most experimental findings were reproduced in independent experiments and with complementary techniques, as explained in detail in the main text and figure legends.

The immunoblot results in Fig. 1c, on an unperturbed tau pattern in the presence of EXSISERS, were reproduced in Extended Data Fig. 4, as well as in Supplementary Figs. 3–5 and 7.

The bioluminescence signals after *MAPT* induction in Fig. 1d were reproduced in Figs. 2, 4 and 5, and Supplementary Fig. 10.

The effects of ITU reported in Fig. 2d,e were independently shown in HEK293T cells in Extended Data Fig. 4 and further controlled in Supplementary Fig. 8. The effects were also replicated in smNPC in Fig. 3c.

The effects of isoform-specific perturbation of *MAPT* assessed by dual-luciferase readout of EXSISERS_*MAPT*:10NLuc-11FLuc_ in Figs. 4d and 5b were validated by RT–qPCR (Extended Data Fig. 6b,c).

The results on Cas13d-based splicing suppression and enhancement reported in Fig. 5 were replicated with independent clones in Supplementary Fig. 10.

The finding on enhancing *Rf*xCas13d activity by extending the spacer length shown in Fig. 4b was validated at the RNA level using RT–qPCR in Extended Data Fig. 6a and at the protein level on another target in Supplementary Fig. 9.

The results of the EXSISERS CRISPR screen (Figs. 6c and 7a) were validated using sgRNAs to target an independent EXSISERS_*FOXP1*:18bBSD_ clone.

A table of all statistical tests is provided in Supplementary Table 1.

### Reporting Summary

Further information on research design is available in the Nature Research Reporting Summary linked to this article.

## Online content

Any methods, additional references, Nature Research reporting summaries, source data, extended data, supplementary information, acknowledgements, peer review information; details of author contributions and competing interests; and statements of data and code availability are available at 10.1038/s41556-021-00678-x.

## Supplementary information

Supplementary Information Supplementary Figs. 1–15, Supplementary Data on FACS analysis.

Reporting Summary

Peer Review Information

Supplementary Table 1 Results of statistical tests.

Supplementary Table 2 Sequences of genetic constructs.

## Data Availability

Unprocessed immunoblots are provided in the Source Data for the relevant figures and the Supplementary Information. Results of all of the statistical tests are provided in Supplementary Table 1. Source data are provided with this paper. All other data supporting the findings of this study are available from the corresponding author on reasonable request.

## Source data

Source Data Fig. 1 Unprocessed immunoblot. Source Data Fig. 1 Numerical raw data. Source Data Fig. 2 Numerical raw data. Source Data Fig. 3 Numerical raw data. Source Data Fig. 4 Numerical raw data. Source Data Fig. 5 Numerical raw data. Source Data Fig. 6 Numerical raw data. Source Data Fig. 6 Unprocessed immunoblot. Source Data Fig. 7 Numerical raw data. Source Data Fig. 7 Unprocessed immunoblot. Source Data Extended Data Fig. 1 Unprocessed immunoblot. Source Data Extended Data Fig. 2 Unprocessed immunoblot. Source Data Extended Data Fig. 3 Unprocessed immunoblot. Source Data Extended Data Fig. 3 Numerical raw data. Source Data Extended Data Fig. 4 Unprocessed immunoblot. Source Data Extended Data Fig. 6 Numerical raw data. Source Data Extended Data Fig. 8 Unprocessed immunoblot. Source Data Extended Data Fig. 8 Numerical raw data. Source Data Extended Data Fig. 9 Numerical raw data. Source Data Extended Data Fig. 9 Unprocessed immunoblot.
