# Supplementary material for: Non-invasive and high-throughput interrogation of exon-specific isoform expression
Source: Nat Cell Biol. 2021 Jun 3;23(6):652–63. doi: 10.1038/s41556-021-00678-x (PMC8189919; doi:10.1038/s41556-021-00678-x)
Supplement: Supplementary file 1 — Supplementary Figs. 1–15, Supplementary Data on FACS analysis. [file 41556_2021_678_MOESM1_ESM.pdf]

nature portfolio

https://doi.org/10.1038/s41556-021-00678-x

Supplementary information

Non-invasive and high-throughput interrogation of exon-specific isoform expression

In the format provided by the
authors and unedited

Supplementary Information

Non-invasive and high-throughput interrogation of exon-specific isoform expression

Dong-Jiunn Jeffery Truong[1,2], Teeradon Phlairaharn[1,2], Bianca Eßwein[3], Christoph Gruber[3], Deniz Tümen[1,4], Enikő Baligács[1,2], Niklas Armbrust[1,2], Francesco Leandro Vaccaro[1,2], Eva-Maria Lederer[1,2], Eva Magdalena Beck[1,2], Julian Geilenkeuser[1,2], Simone Göppert[1,2], Luisa Krumwiede[1,2], Christian Grätz[1,2], Gerald Raffl[1,2], Dominic Schwarz[1,2], Martin Zirngibl[1,2], Milica Živanić[1,2], Maren Beyer[1,2], Johann Dietmar Körner[1,2], Tobias Santl[1,2], Valentin Evsyukov[5,6,7], Tabea Strauß[5,6], Sigrid C. Schwarz[5,6], Günter U. Höglinger[5,6,7], Peter Heutink[8,9], Sebastian Doll[10], Marcus Conrad[10,11], Florian Giesert[3], Wolfgang Wurst[3,5,12], Gil Gregor Westmeyer[1,2,✉]

[1]Institute for Synthetic Biomedicine, Helmholtz Zentrum München, 85764 Oberschleißheim, Germany
[2]Department of Chemistry and TUM School of Medicine, Technical University of Munich, 81675 Munich, Germany
[3]Institute of Developmental Genetics, Helmholtz Zentrum München, 85764 Oberschleißheim, Germany
[4]Department of Internal Medicine I, University Hospital Regensburg, Regensburg, Germany
[5]German Center for Neurodegenerative Diseases (DZNE), Munich, Germany
[6]Department of Neurology, Technical University Munich, Munich, Germany
[7]Department of Neurology, Hannover Medical School, Hannover, Germany
[8]Department for Neurodegenerative Diseases, Hertie Institute for Clinical Brain Research, University of Tübingen, 72076 Tübingen, Germany
[9]German Center for Neurodegenerative Diseases (DZNE), 72076 Tübingen, Germany
[10]Institute of Metabolism and Cell Death, Helmholtz Zentrum München, 85764 Oberschleißheim, Germany
[11]National Research Medical University, Laboratory of Experimental Oncology, Ostrovityanova 1, Moscow 117997, Russia.
[12]TUM School of Life Sciences, Technical University of Munich, 85354 Freising, Germany.
✉e-mail: gil.westmeyer@tum.de

Table of Content

Note: Please find each Supplementary Figure on the page number corresponding to the Figure's index or via the links below.

- Supplementary Fig. 1 | Single-step, 3-day protocol for generating the EXSISERS donor/targeting plasmid and the CRISPR-Cas9 plasmid.
- Supplementary Fig. 2 | Schematic protocol for generating an EXSISERS cell line.
- Supplementary Fig. 3 | Anti-pan-tau immunofluorescence staining of unmodified HEK293T cells after MAPT induction and densitometric quantification of tau isoforms.
- Supplementary Fig. 4 | Immunoblots from additional clones carrying EXSISERS in exon 10, 11 and the IVS10+16 C>T mutation homozygously.
- Supplementary Fig. 5 | Full immunoblots from additional clones carrying EXSISERS in exon 10, 11, and the IVS10+16 C>T mutation homozygously.
- Supplementary Fig. 6 | Immunoblots of HEK293T parental cells and different clones of HEK293T with unmodified MAPT locus.
- Supplementary Fig. 7 | Characterization of EXSISERS$_{MAPT\text{:10-NLuc}}$ clonal cell line with an alternative insertion site.
- Supplementary Fig. 8 | Effects of 5-iodotubercidin on tau in unmodified HEK293T cells.
- Supplementary Fig. 9 | *MAPT*-independent validation of enhancing *Rfx*Cas13d activity by extending the spacer length.
- Supplementary Fig. 10 | Replication of key aspects of Cas13d-based splicing suppression and enhancement.
- Supplementary Fig. 11 | Analysis of fluorescent variants of EXSISERS$_{MAPT\text{:10mNG}}$ cells.
- Supplementary Fig. 12 | Genotyping results of EXSISERS$_{MAPT\text{:10Halo}}$ clones.
- Supplementary Fig. 13 | Confirmation of diploid karyotype and cell line identities.
- Supplementary Fig. 14 | Confirmation of neuronal identity in EXSISERS$_{MAPT\text{:10NLuc-FLuc11}}$ human iPSC-derived neurons.
- Supplementary Fig. 15 | Uncropped images.
- Supplementary Data on FACS analysis

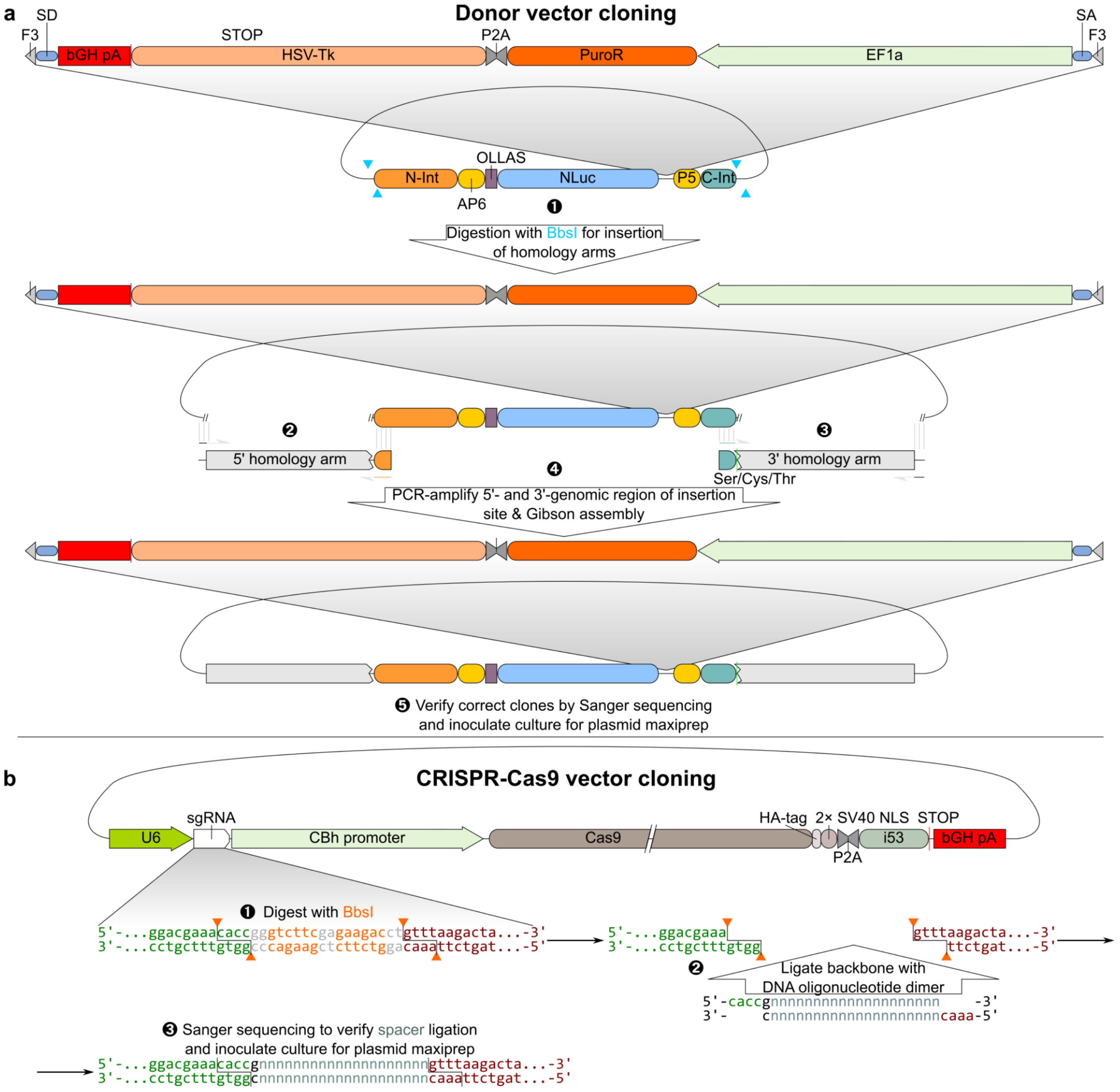

Supplementary Fig. 1 | Single-step, 3-day protocol for generating the EXSISERS donor/targeting plasmid and the CRISPR-Cas9 plasmid. a, The donor/targeting plasmid is created by the digestion of the EXSISERS plasmid with the type IIS restriction enzyme BbsI (isoschizomer: BpiI). The resulting two fragments are assembled via isothermal Gibson assembly together with the 5'- and 3'-homology arms, generated via PCR from the genomic DNA flanking the genomic insertion site. The insertion site must be upstream of the amino acid cysteine, serine, or threonine. The assembled product is transformed into a suitable *E. coli* cloning strain, and colonies are inoculated on a small scale (2 ml) for a plasmid miniprep. The homology arms and the Gibson assembly overlapping nucleotides are sequenced using a standard Sanger protocol on miniprep plasmid DNA. Correct clones are re-inoculated in a 50-100 ml culture overnight for a plasmid midiprep/maxiprep culture at 30/37 °C. If the cell line of choice is endotoxin-sensitive, an endotoxin-free plasmid midi/maxiprep kit should be used to isolate the plasmid DNA. **b,** The CRISPR-Cas9 cloning plasmid is digested with the type IIS restriction enzyme BbsI. The resulting plasmid backbone is ligated with a DNA oligonucleotide dimer. The dimer is created by annealing two complementary ssDNA with a corresponding 4 nt 5'- and 3'-extension complementary to the 5'- and 3'-overhangs from the digested plasmid backbone.

Click here to go back to the Table of Contents

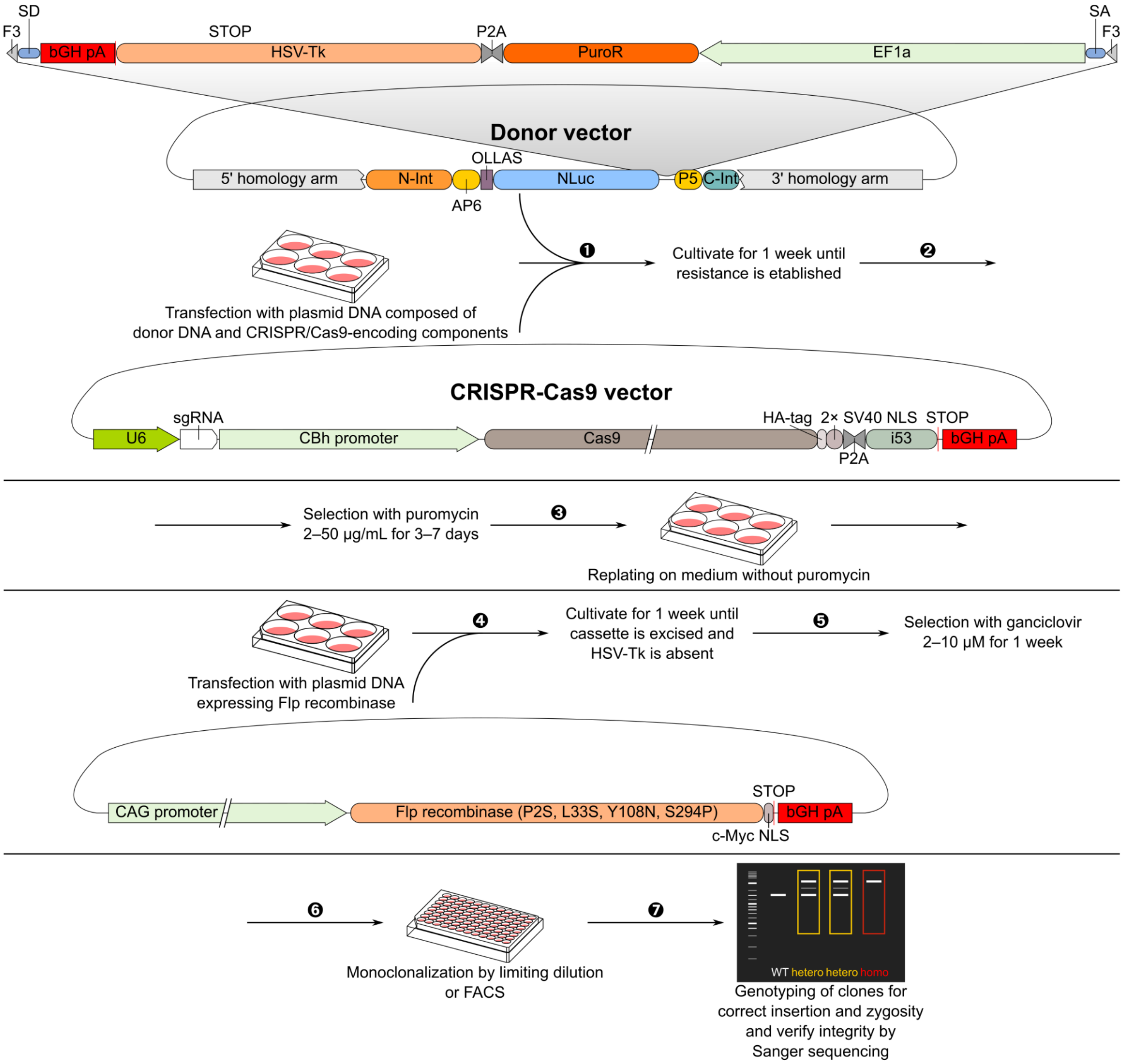

Supplementary Fig. 2 | Schematic protocol for generating an EXSISERS cell line. As a summary, the procedure uses a customized donor/targeting plasmid, a CRISPR-Cas9 plasmid, and a Cre/Flp-recombinase expressing plasmid. Both the DNA donor and the CRISPR-Cas9 plasmids are delivered to the cell line of choice via transfection or electroporation (nucleofection). 2-7 days later, the cells are selected with puromycin (concentration for each cell line has to be determined with a classical kill curve, usually ranging from 2-50 µg/ml) for 3-7 days. The cells are replated without puromycin, and the cells are transfected/electroporated with a plasmid expressing a suitable recombinase (Flp or Cre) to remove the selection cassette. After a week of regeneration, the cells are counter-selected with ganciclovir (typically 2-10 µM) for at least a week. During this time, the medium has to be changed at least every 2 days to avoid the accumulation of toxic ganciclovir triphosphate. Cells that still contain the selection cassette will be eradicated during this selection. Only cells carrying EXSISERS but without the selection cassette will survive. The surviving population is monoclonalized via limiting dilution or FACS, and clones are genotyped for their zygosity. If heterozygous clones are chosen, the WT band must be sent for Sanger sequencing to exclude NHEJ-mediated insertions/deletions in the WT allele. Additional small mutations can be co-introduced efficiently to the genomic site of interest on the EXSISERS donor construct if the mutation is located within 100 nt of the CRISPR-Cas9-mediated double-strand break.

Click here to go back to the Table of Contents

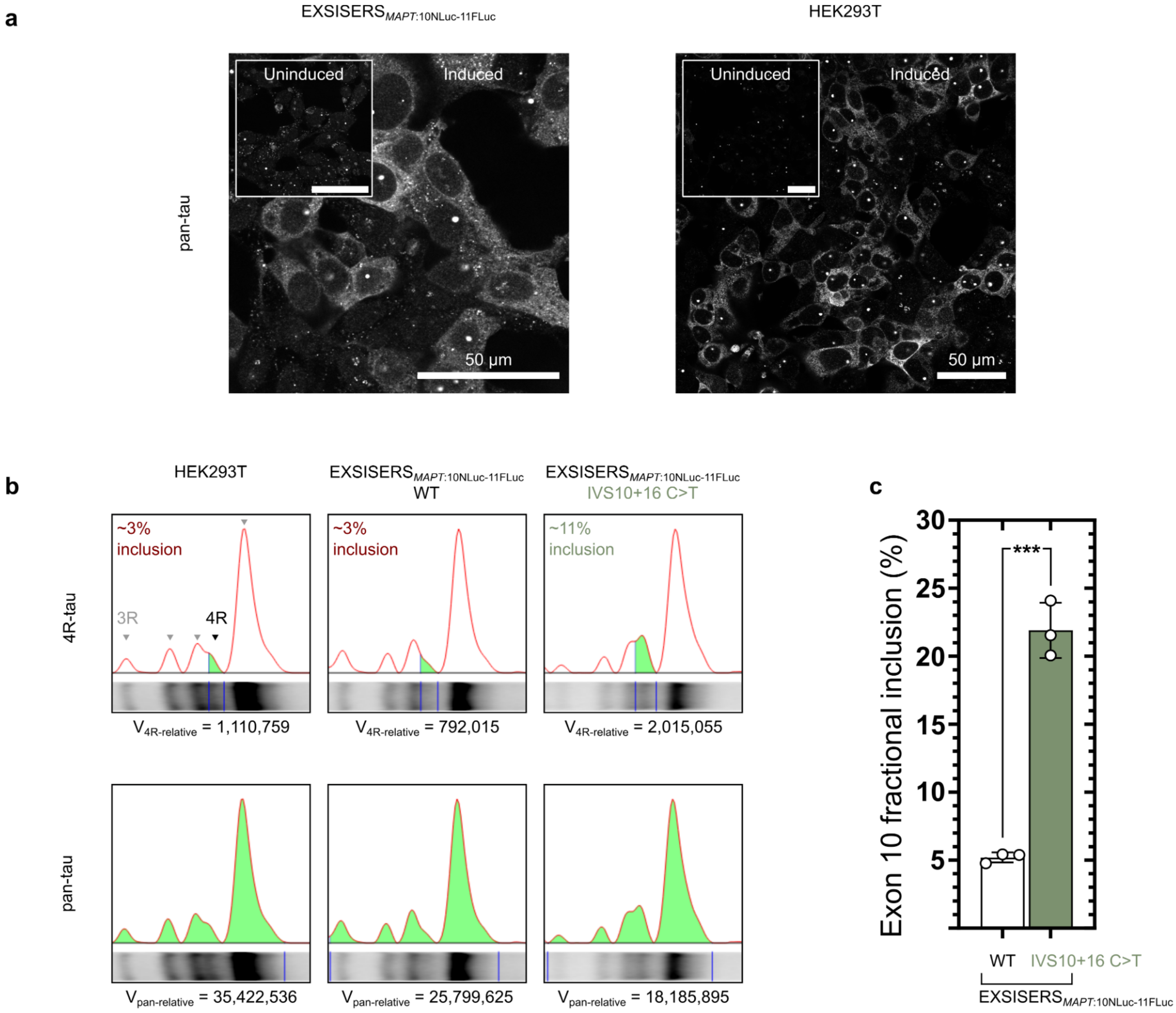

Supplementary Fig. 3 | Anti-pan-tau immunofluorescence staining of unmodified HEK293T cells after *MAPT* induction and densitometric quantification of tau isoforms. a, Immunofluorescence staining (anti-pan-tau) of unmodified HEK293T cells without (left) and with (right) *MAPT* induction. **b,** Densitometric quantification of the immunoblot shown in Fig. 1c to determine the inclusion fraction of Exon 10 (ratio of 4R band (+exon 10) and 3R band (−exon 10)) in HEK293T cells, $EXSISERS_{MAPT:10NLuc-11FLuc}$, and $EXSISERS_{MAPT:10NLuc-11FLuc}$ with the IVS10+16 C>T mutation after *MAPT* induction. The latter resulted in a 3.7-fold increase of exon 10 inclusion. Please note that densitometric quantification of tau isoforms has limited precision due to the partially overlapping bands. **c,** Quantification (mean value ± s.d. (n = 3, biological replicates)) of the corresponding dual-luciferase assay shown in Fig. 1d showed a 4.2-fold increased fractional exon 10 inclusion (computed by adjusting for the relative brightness of the luciferases, see Extended Data Fig. 2c) after *MAPT* induction in $EXSISERS_{MAPT:10NLuc-FLuc}$ IVS10+16 C>T cells compared to WT MAPT. *** denote p-values smaller than 0.001 in a two-tailed unpaired Student's t-test (full statistical results are available in **Supplementary Table 1**).

Click here to go back to the Table of Contents

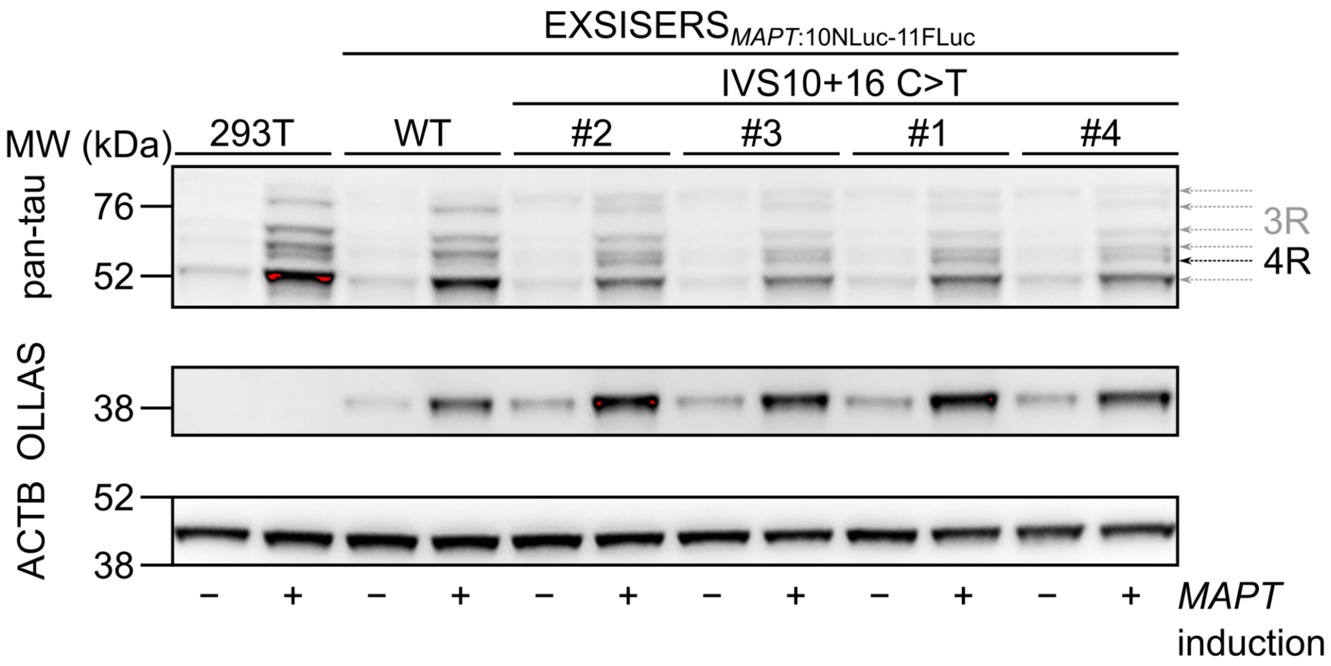

Supplementary Fig. 4 | Immunoblots from additional clones carrying EXSISERS in exon 10, 11 and the IVS10+16 C>T mutation homozygously. Pan-tau and OLLAS (indicative for *MAPT* exon 10) immunoblot from additional homozygous EXSISERS 293T clones with *MAPT* IVS10+16 *vs.* unmutated EXSISERS and WT HEK293T cells (dephosphorylated lysate). Clone #1 is used in Fig. 1c.

Click here to go back to the Table of Contents

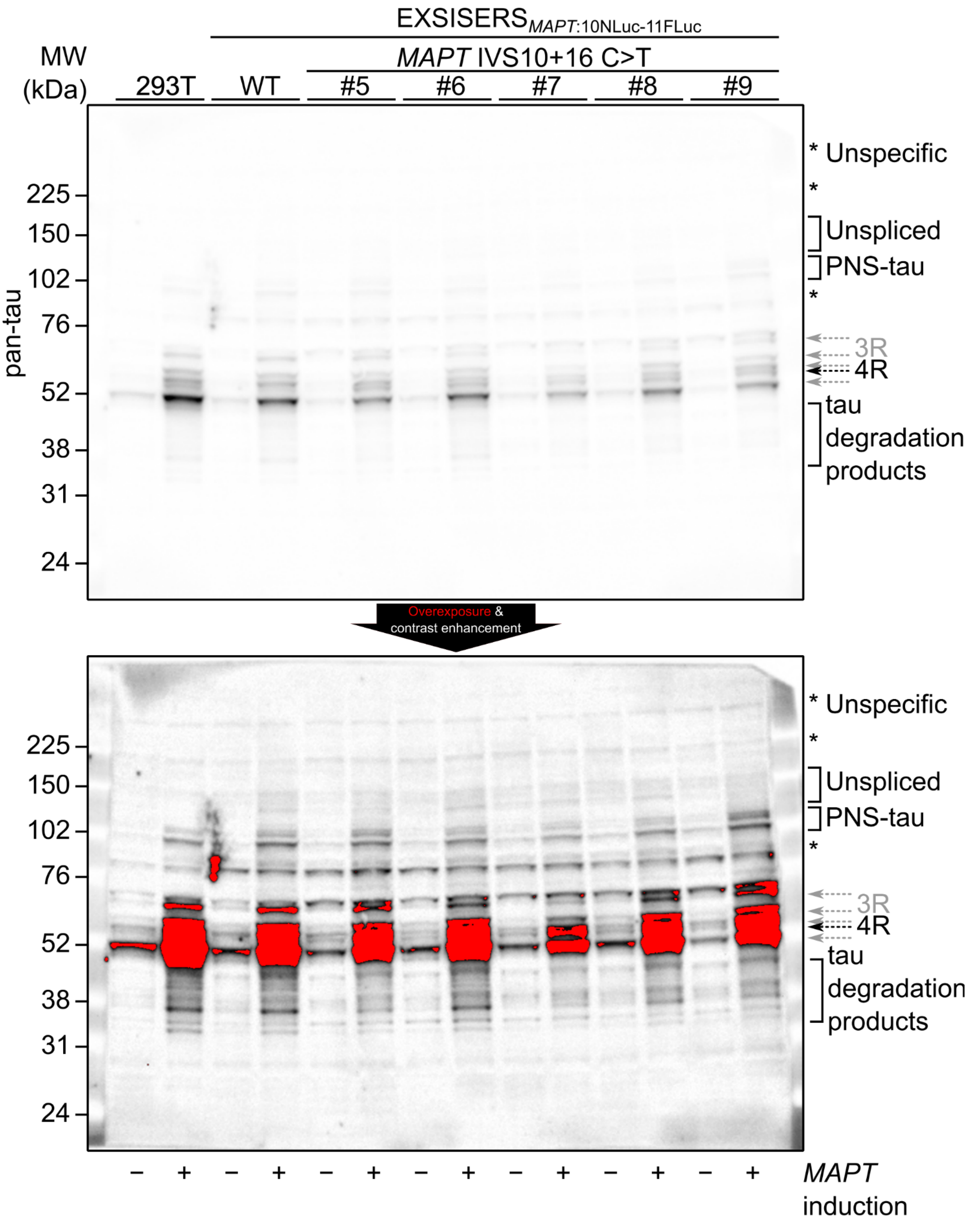

Supplementary Fig. 5 | Full immunoblots from additional clones carrying EXSISERS in exon 10, 11, and the IVS10+16 C>T mutation homozygously. Pan-tau immunoblot from additional homozygous EXSISERS$_{MAPT\text{:10NLuc-11FLuc}}$ 293T clones with *MAPT* IVS10+16 *vs.* unmutated EXSISERS and WT HEK293T cells (dephosphorylated lysate). Unspliced products (probably from *de novo* translation) could only be detected with massive overexposure and contrast enhancement. Please note the large number of bands visible upon overexposure, which are also present in unmodified HEK293T cells.

Click here to go back to the Table of Contents

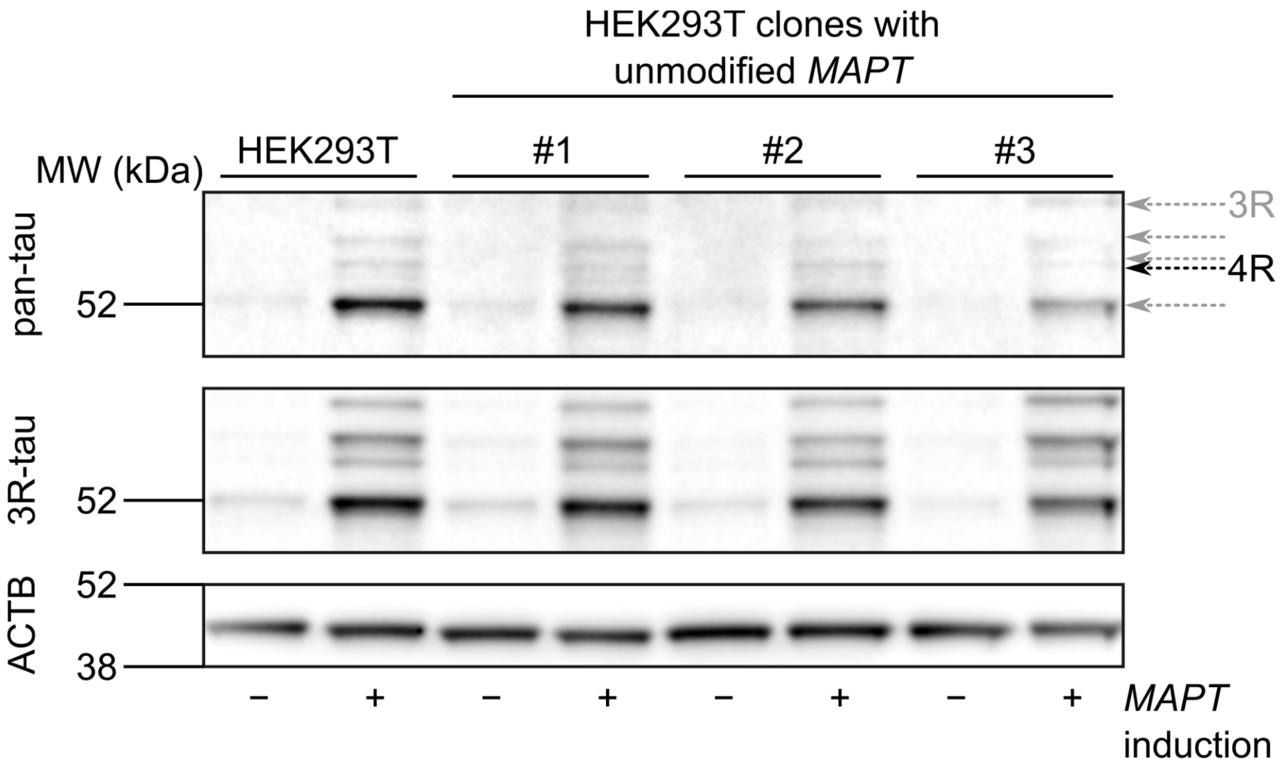

Supplementary Fig. 6 | Immunoblots of HEK293T parental cells and different clones of HEK293T with unmodified *MAPT* locus. *MAPT* was induced in the parental HEK293T cells and in subclones with unmodified *MAPT* locus. 4R and 3R bands are identified via pan- and 3R-tau-specific antibodies (dephosphorylated lysate).

Click here to go back to the Table of Contents

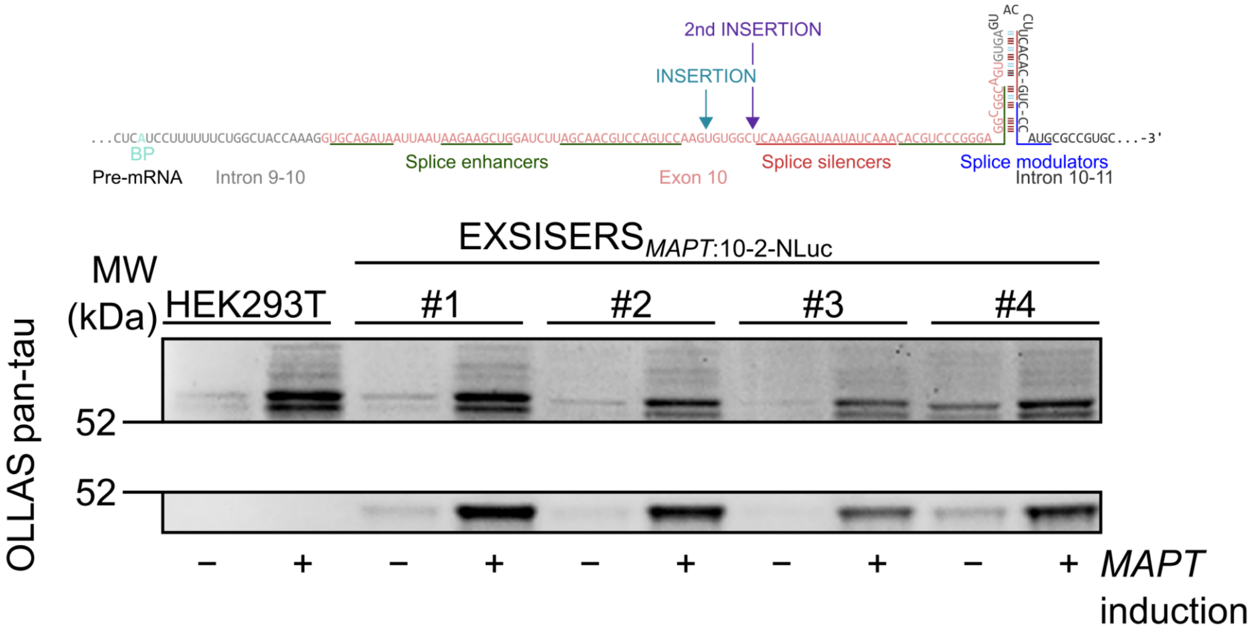

Supplementary Fig. 7 | Characterization of EXSISERS$_{MAPT\text{:10-NLuc}}$ clonal cell line with an alternative insertion site. Location of the alternative insertion site (2nd insertion) with the insertion site used in EXSISERS$_{MAPT\text{:10NLuc-11FLuc}}$. Anti-pan-tau immunoblot analysis of EXSISERS$_{MAPT\text{:10-NLuc}}$ in comparison to unmodified HEK29T revealed no apparent change in the patterning of tau after CRISPR-mediated induction of *MAPT*.

Click here to go back to the Table of Contents

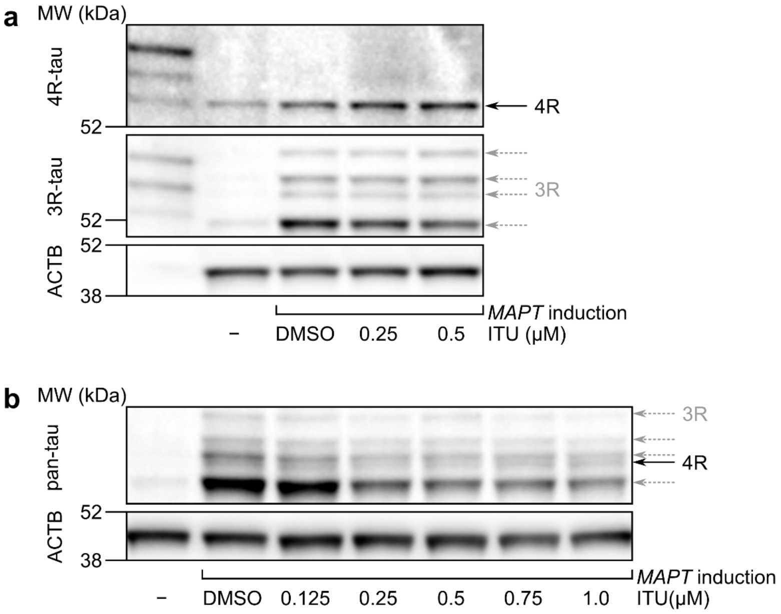

Supplementary Fig. 8 | Effects of 5-iodotubercidin on tau in unmodified HEK293T cells. a, Immunoblot analysis (anti-4R- and anti-3R-tau) of dephosphorylated cell lysates shows a concentration-dependent inclusion of exon 10 (resulting in expression of 4R-tau) as a function of the kinase inhibitor 5-iodotubercidin (ITU). **b,** Immunoblot analysis (pan-tau) showed an ITU concentration-dependent relative inclusion of exon 10 (dephosphorylated cell lysate).

Click here to go back to the Table of Contents

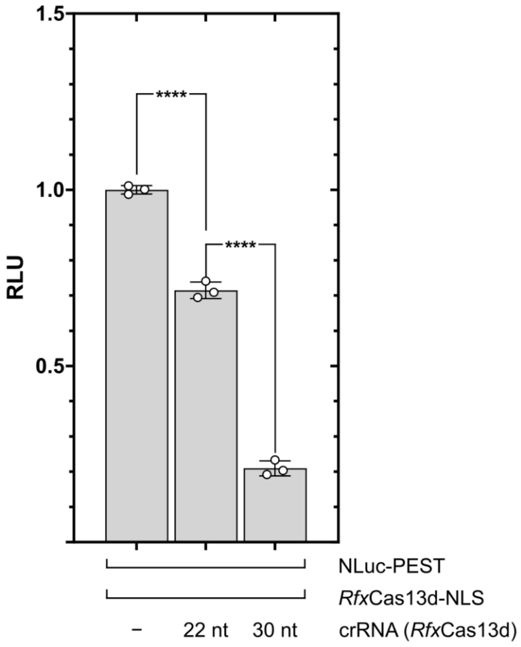

Supplementary Fig. 9 | *MAPT*-independent validation of enhancing *Rfx*Cas13d activity by extending the spacer length. Normalized NLuc luciferase signal (mean value of 3 biological replicates ± s.d.) 48 hours post-transfection from samples transfected with NLuc-PEST and *Rfx*Cas13d-NLS expressing plasmids with an NLuc-targeting crRNA containing the indicated spacer length or non-targeting control (-). Results of ANOVA with Bonferroni MTC shown, where **** denotes *p*-values < 0.0001 (full statistical results are available in **Supplementary Table 1**).

Click here to go back to the Table of Contents

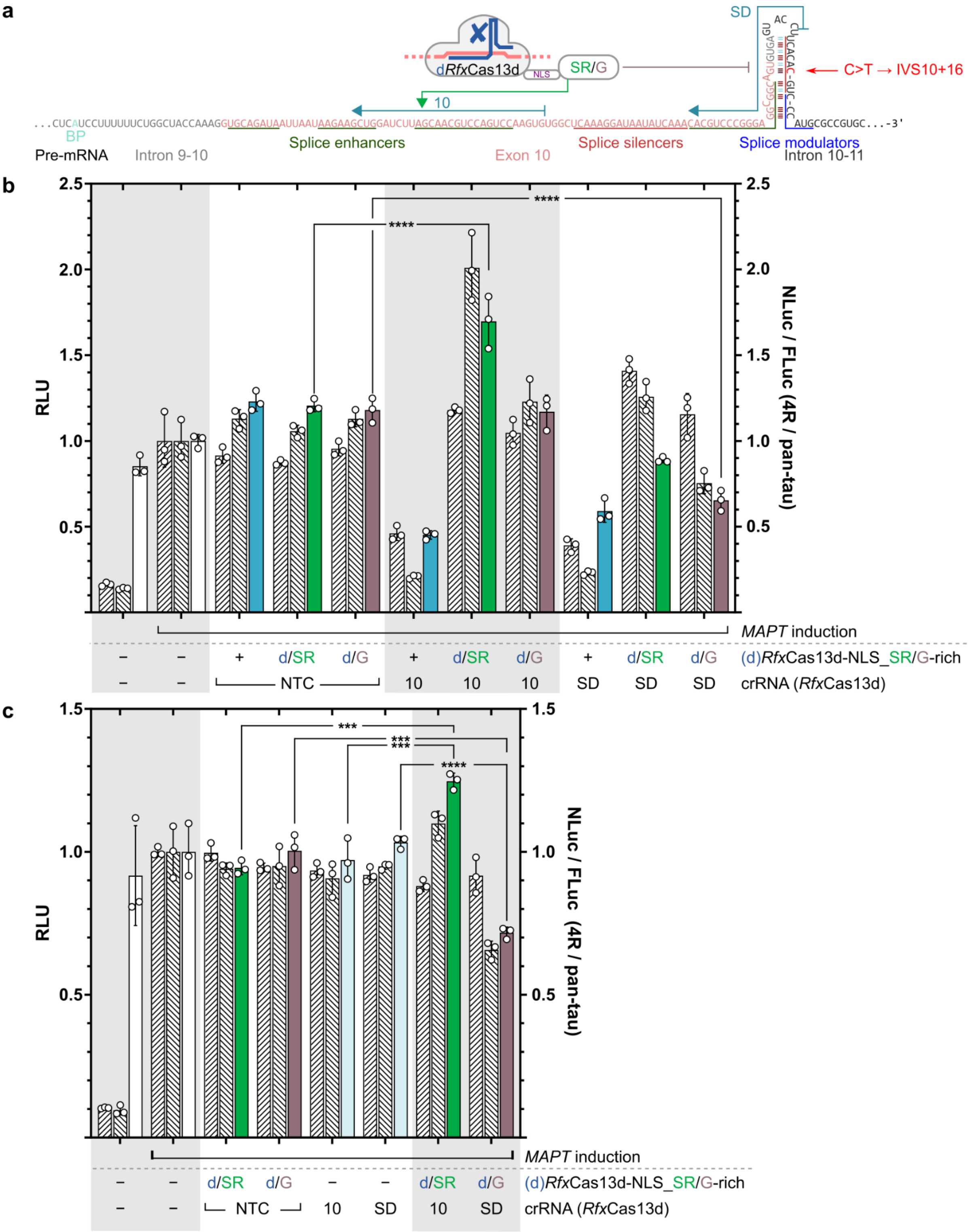

Supplementary Fig. 10 | Replication of key aspects of Cas13d-based splicing suppression and enhancement. a. Binding sites of (d)*Rfx*Cas13d-NLS fusion chimeras on the *MAPT* pre-mRNA. G-rich/SR-rich domains were fused to nuclease-inactivated versions of *Rfx*Cas13d-NLS to create RNA-guided splice suppressors (G-rich) or enhancers (SR-rich). **b,** Replication of key aspects of Fig. 5e with protein-only controls on an independent clone of EXSISERS$_{MAPT:10NLuc\text{-}11FLuc}$. **c,** Repetition of d*Rfx*Cas13d-based effectors on an independent EXSISERS$_{MAPT:10NLuc\text{-}11FLuc}$ ISV10+16 C>T clonal line with non-targeting crRNA controls (NTC). Data points represent the mean value of 3 biological replicates ± s.d. Only the most relevant results of one-way ANOVA with Bonferroni MTC are shown, where *** and **** denote *p*-values smaller than 0.001 and 0.0001 (full statistical results are available in **Supplementary Table 1**).

Click here to go back to the Table of Contents

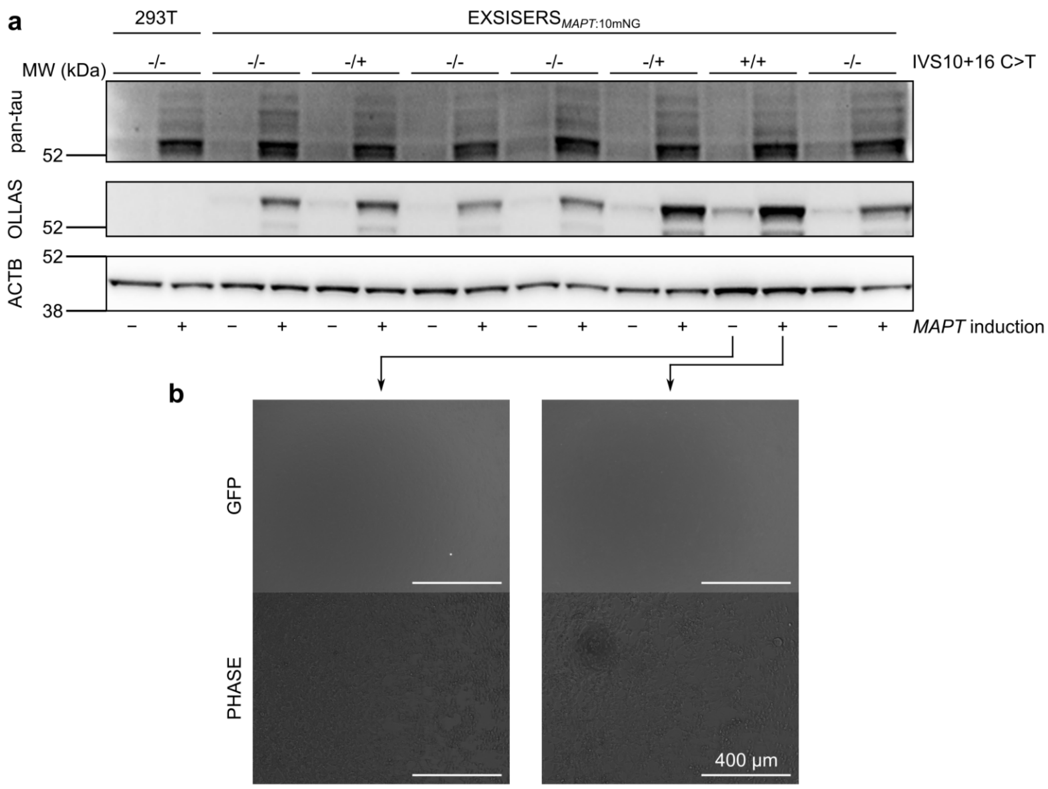

Supplementary Fig. 11 | Analysis of fluorescent variants of EXSISERS$_{MAPT:10mNG}$ cells. a, Immunoblot against pan-tau, OLLAS (mNeonGreen$_{OLLAS}$ in exon 10) of indicated clones; WT indicates EXSISERS$_{mNeonGreen}$ cells without any further mutations; het. indicates heterozygous clones (but homozygous for EXSISERS insertion) for the IVS10+16 C>T mutation within the intron downstream of exon 10; homo. indicates homozygous clones (also homozygous for EXSISERS insertion) for the IVS10+16 C>T mutation within the intron downstream of exon 10. The lysate was not dephosphorylated. **b,** Epifluorescence images of EXSISERS$_{MAPT:10mNG}$ cells with the IVS10+16 C>T mutation homozygously integrated into exon 10. Fluorescent signals without and with tau-induction did not reach sufficient levels to be readily detected without additional immunofluorescence staining.

Click here to go back to the Table of Contents

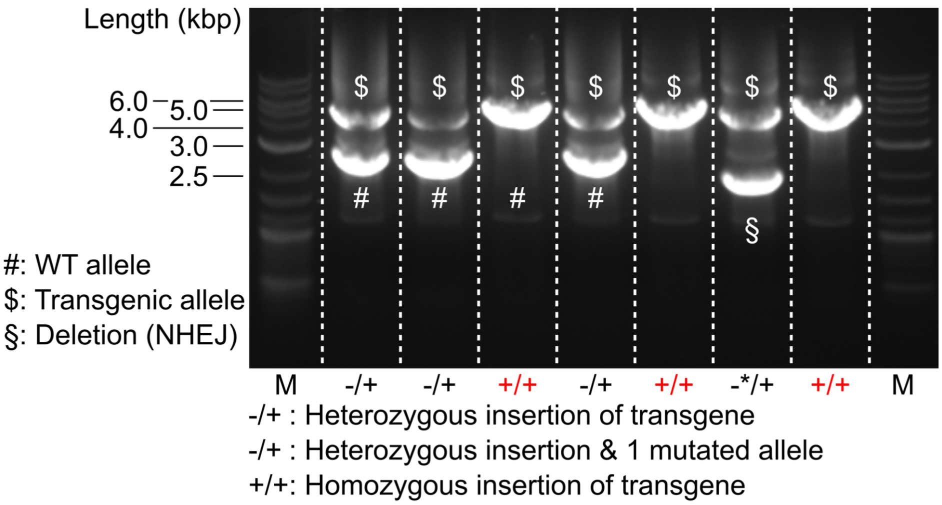

Supplementary Fig. 12 | Genotyping results of EXSISERS$_{MAPT:10Halo}$ clones. Genotyping indicated 3 homozygous (+/+) and 4 heterozygous (-/+) clones for EXSISERS insertion; one heterozygous clone showed an additional NHEJ-induced deletion. M: 1 kb DNA ladder.

Click here to go back to the Table of Contents

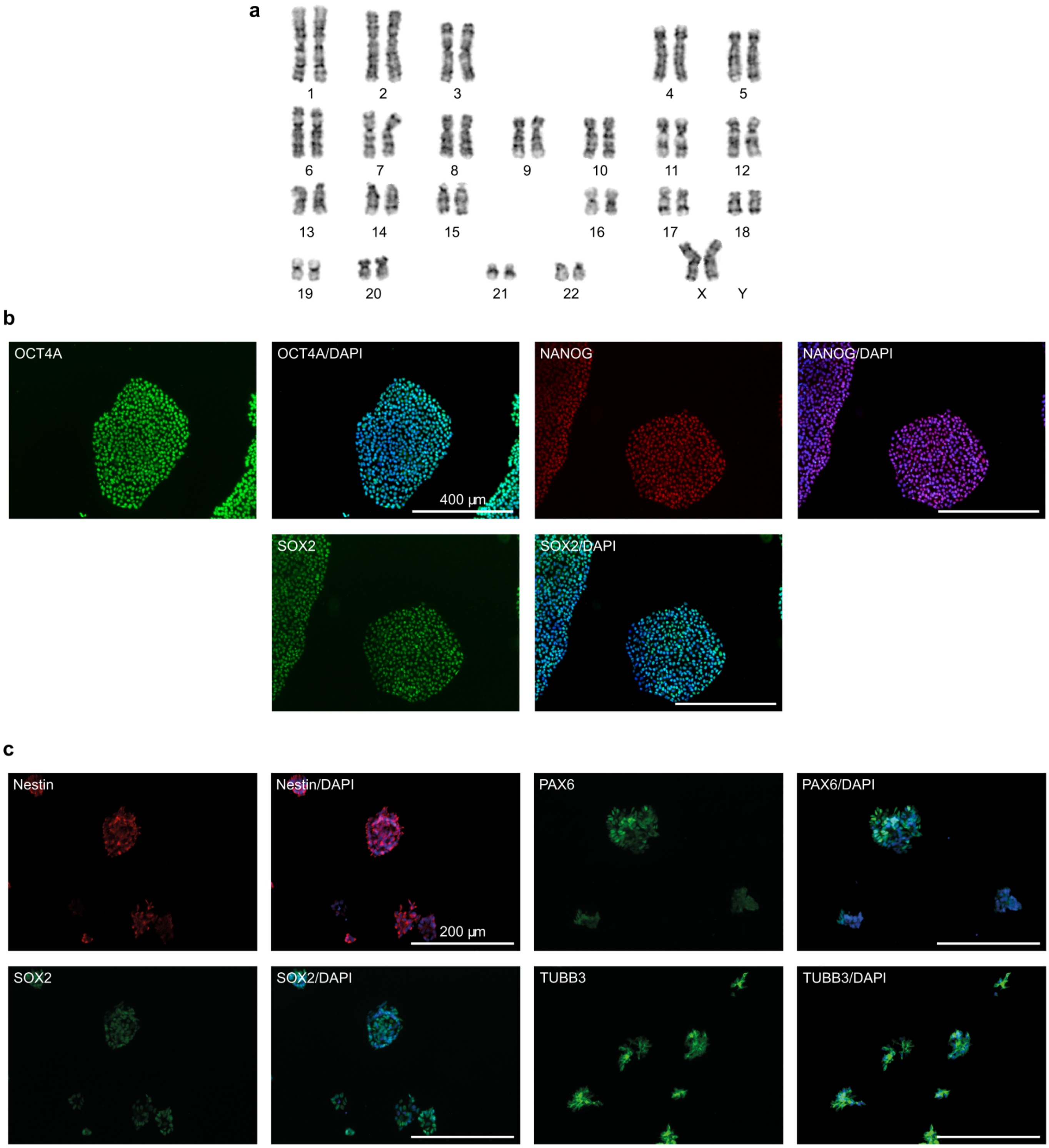

Supplementary Fig. 13 | Confirmation of diploid karyotype and cell line identities. a, G-banding of EXSISERS$_{MAPT:10NLuc/11FLuc}$ hiPSCs confirm the normal female diploid karyotype after CRISPR-Cas9-mediated insertion of EXSISERS in *MAPT* exon 10 and 11. **b,** Immunocytochemistry of OCT4A, NANOG, and SOX2 to confirm pluripotency markers of EXSISERS$_{MAPT:10NLuc/11FLuc}$ human iPSCs. **c,** Immunocytochemistry of Nestin, PAX6, SOX2, and TUBB3 to confirm neural precursor cell identity of EXSISERS$_{MAPT:10NLuc/11FLuc}$ smNPCs.

Click here to go back to the Table of Contents

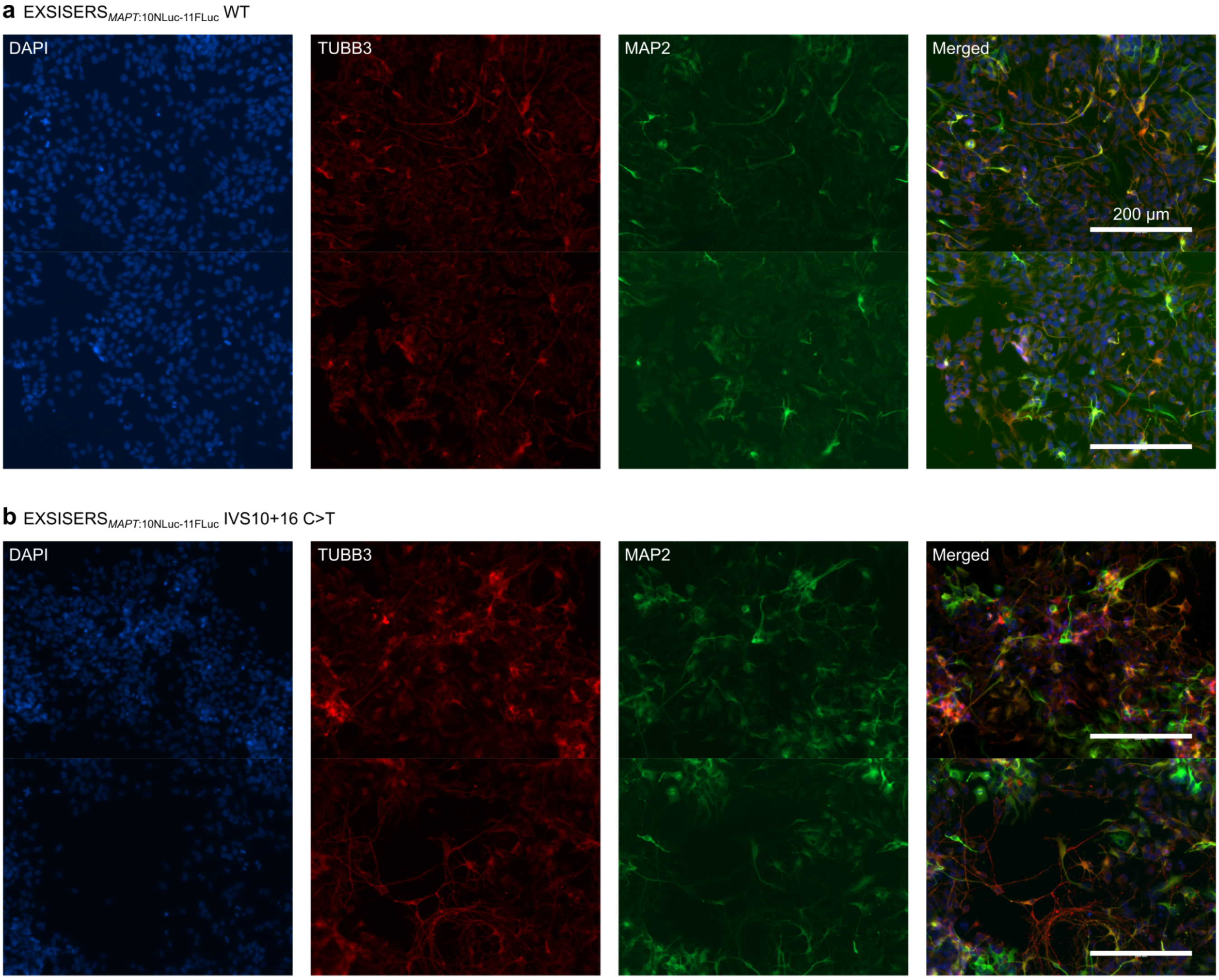

Supplementary Fig. 14 | Confirmation of neuronal identity in EXSISERS$_{MAPT:10NLuc-FLuc11}$ human iPSC-derived neurons. Cells with **a,** WT background or **b,** IVS10+16 C>T mutation were stained at day 70 of *in vitro* differentiation via ICC against TUBB3 and MAP2.

Click here to go back to the Table of Contents

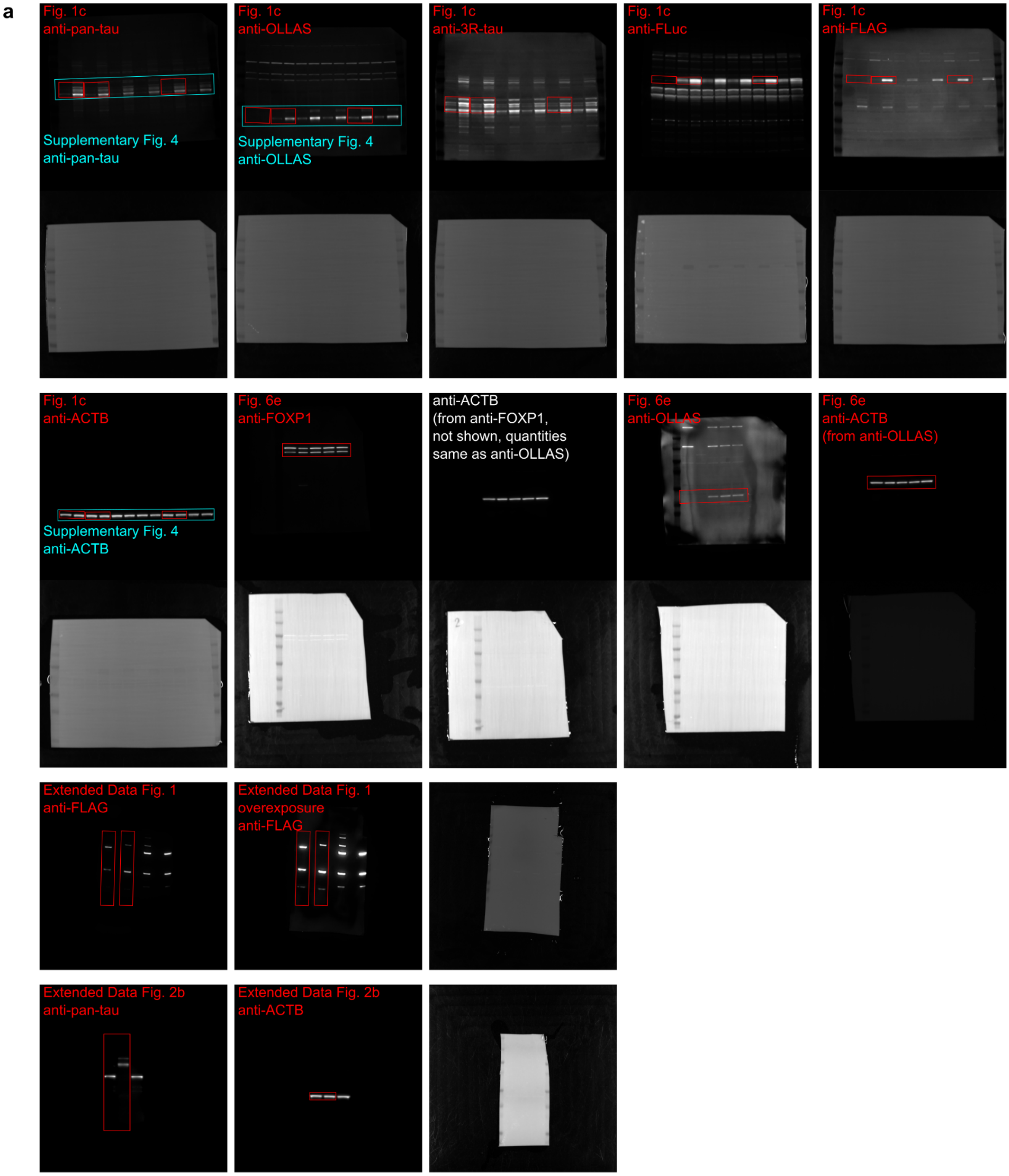

Supplementary Fig. 15 | Uncropped images. a, Uncropped immunoblots. All lysates were dephosphorylated with λ protein phosphatase except immunoblots for Fig. 6e, Extended Data Fig. 1, Extended Data Fig. 3b, Extended Data Fig. 8d, Extended Data Fig. 8m, Extended Data Fig. 9d, and Supplementary Fig. 11a. **b,** Uncropped agarose gel electrophoresis images of semiquantitative RT-PCR analyses.

Click here to go back to the Table of Contents

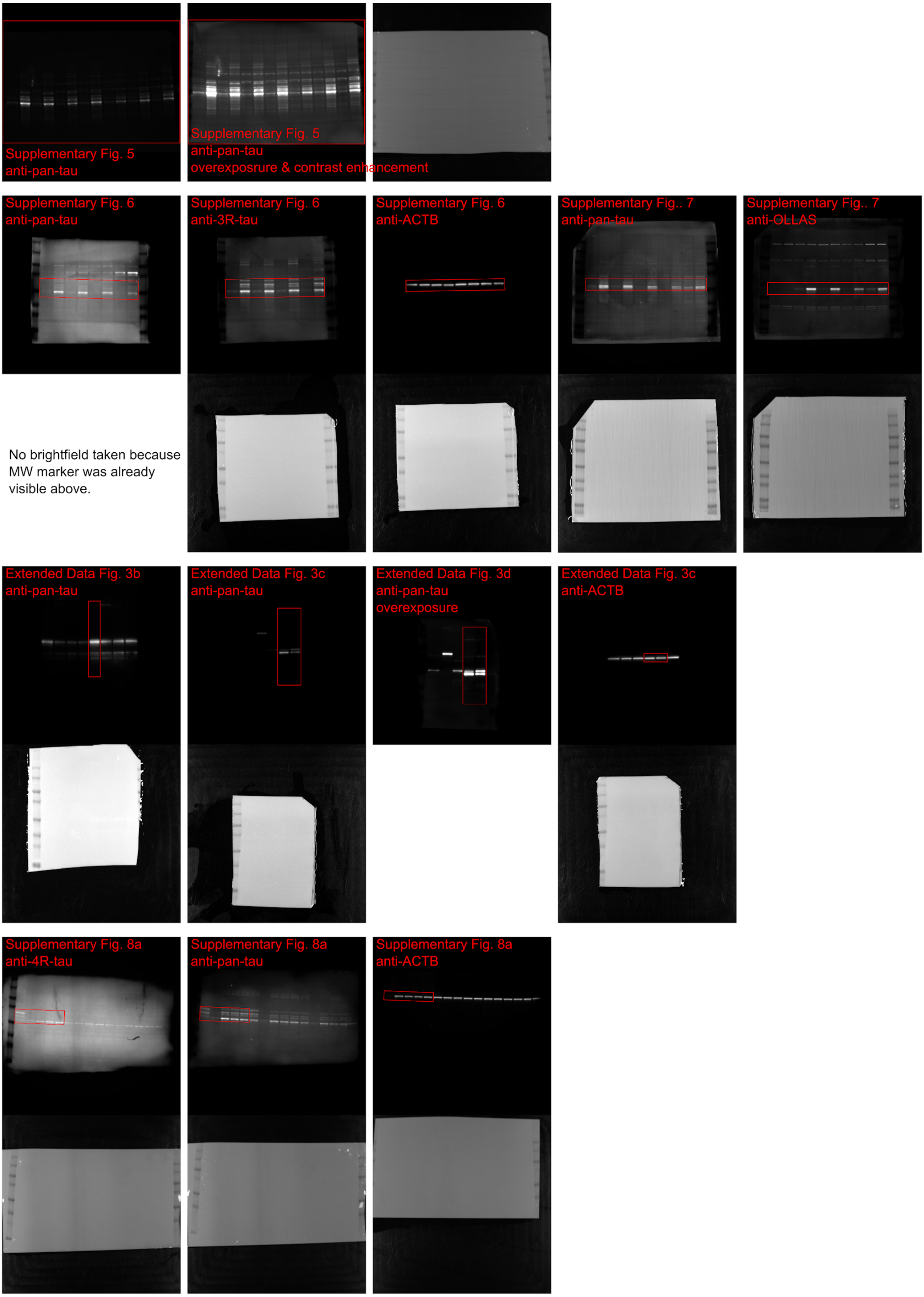

Supplementary Fig. 15 continued

[Click here to go back to the Table of Contents](#)

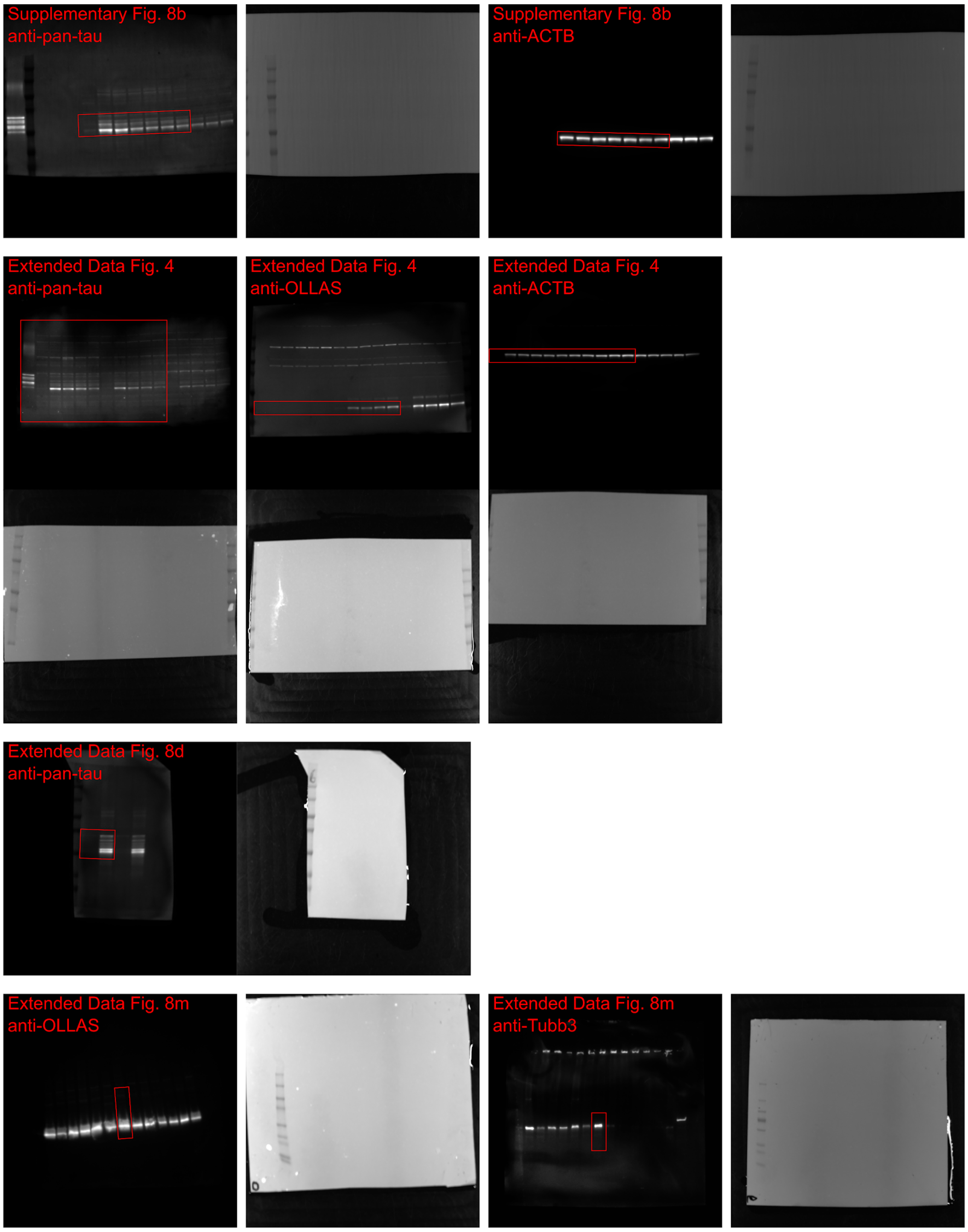

Supplementary Fig. 15 continued

[Click here to go back to the Table of Contents](#)

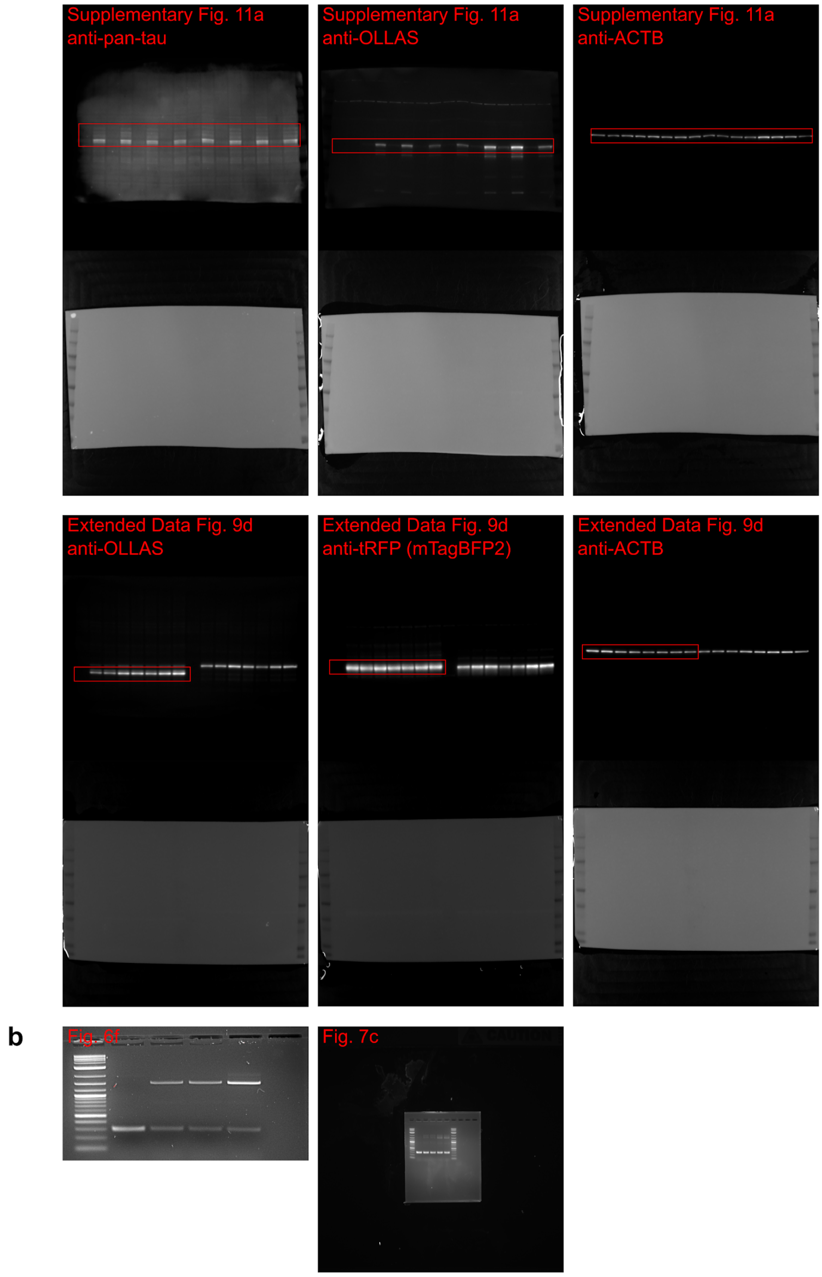

Supplementary Fig. 15 continued

Click here to go back to the Table of Content

Supplementary Data on FACS analysis

Batch Analysis Report

Run Date: 3/22/21 11:35 AM Experiment: 20191104_MmOaz1-EXSISERS_293T

User ID: AllUser

Statistics Output: N/A

Worksheet PDF Output: C:\Documents and Settings\Administrator\Desktop\Jeff\20191104_MmOaz1-

Specimen_001

Tube	Status	Run Time
1-1	OK	3/22/21 11:35 AM
1-2	OK	3/22/21 11:35 AM
1-3	OK	3/22/21 11:35 AM
2-1	OK	3/22/21 11:35 AM
2-2	OK	3/22/21 11:35 AM
2-3	OK	3/22/21 11:35 AM
3-1	OK	3/22/21 11:35 AM
3-2	OK	3/22/21 11:35 AM
3-3	OK	3/22/21 11:36 AM
4-1	OK	3/22/21 11:36 AM
4-2	OK	3/22/21 11:36 AM
4-3	OK	3/22/21 11:36 AM
5-1	OK	3/22/21 11:36 AM
5-2	OK	3/22/21 11:36 AM
5-3	OK	3/22/21 11:36 AM
6-1	OK	3/22/21 11:36 AM
6-2	OK	3/22/21 11:36 AM
6-3	OK	3/22/21 11:36 AM
7-1	OK	3/22/21 11:36 AM
7-2	OK	3/22/21 11:36 AM
7-3	OK	3/22/21 11:36 AM
8-1	OK	3/22/21 11:36 AM
8-2	OK	3/22/21 11:36 AM
8-3	OK	3/22/21 11:36 AM

Samples 2 - 8 correspond to data from Supplementary Fig. 9 c in the same sequence. Control samples (sample 1) are control-transfected cells without blue/green fluorescence to determine the gate settings.

FACSDiva Version 6.1.3

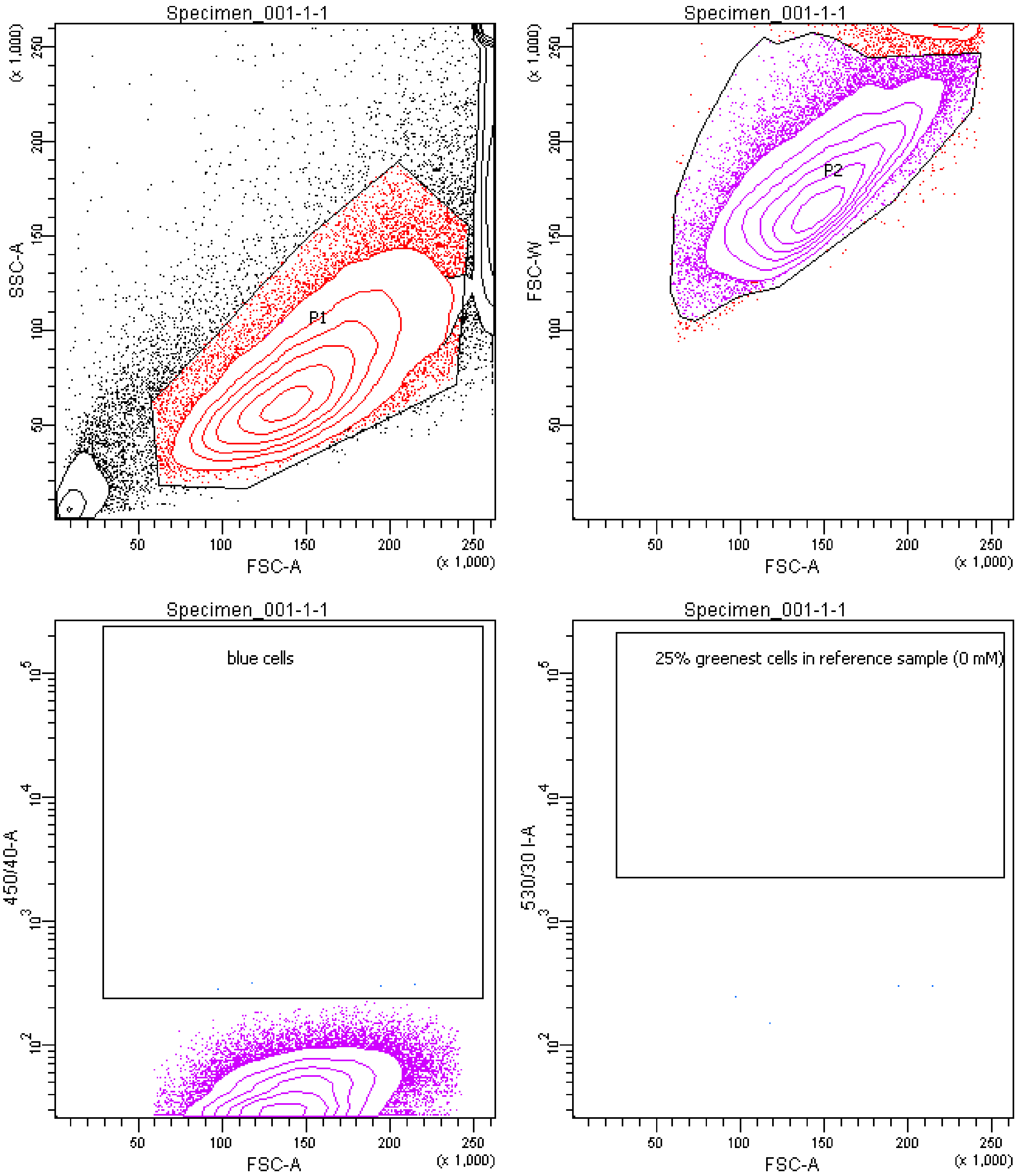

FACSDiva Version 6.1.3

Tube: 1-1

Population	#Events	%Parent	%Total
All Events	50,000	####	100.0
P1	35,428	70.9	70.9
P2	32,977	93.1	66.0
blue cells	4	0.0	0.0
25% greenest cells in reference sample (0 mM)	0	0.0	0.0

FACSDiva Version 6.1.3

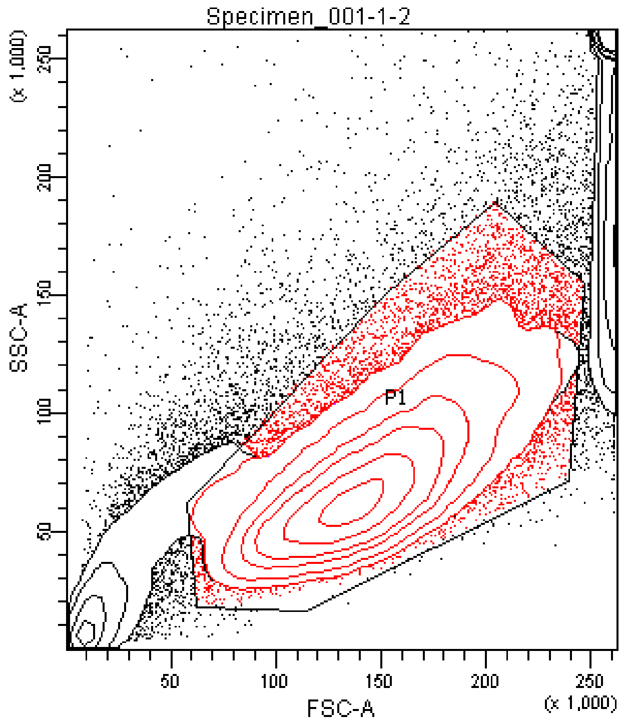

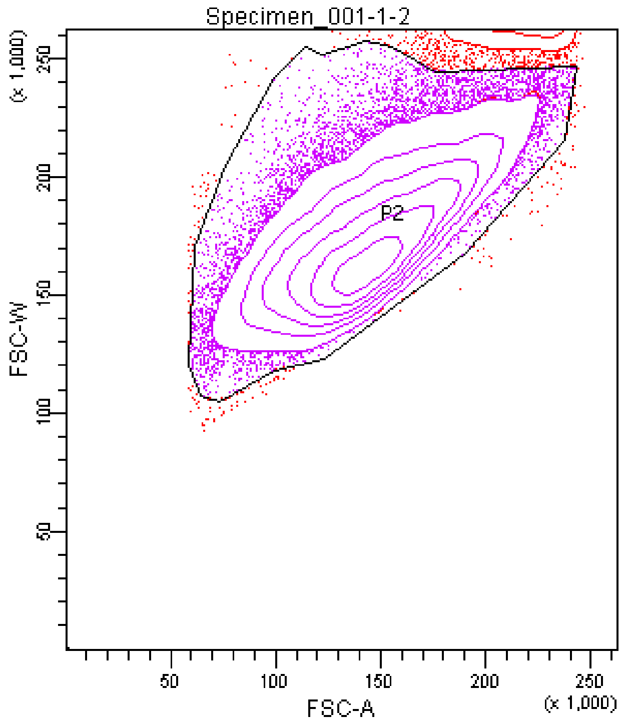

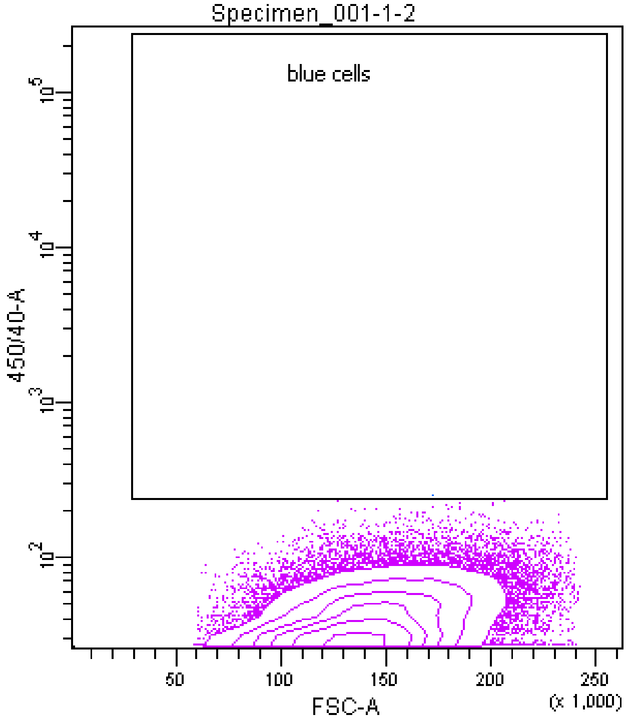

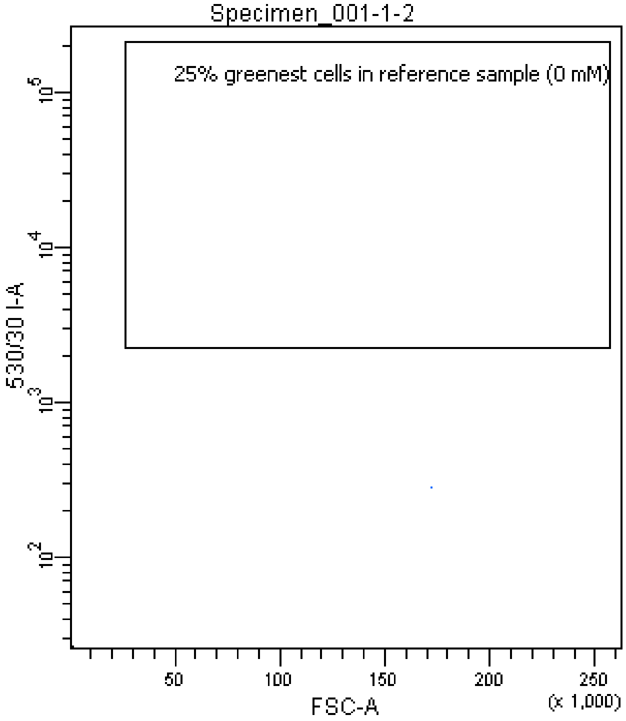

Tube: 1-2

Population	#Events	%Parent	%Total
All Events	50,000	####	100.0
P1	32,821	65.6	65.6
P2	30,345	92.5	60.7
blue cells	1	0.0	0.0
25% greenest cells in reference sample (0 mM)	0	0.0	0.0

FACSDiva Version 6.1.3

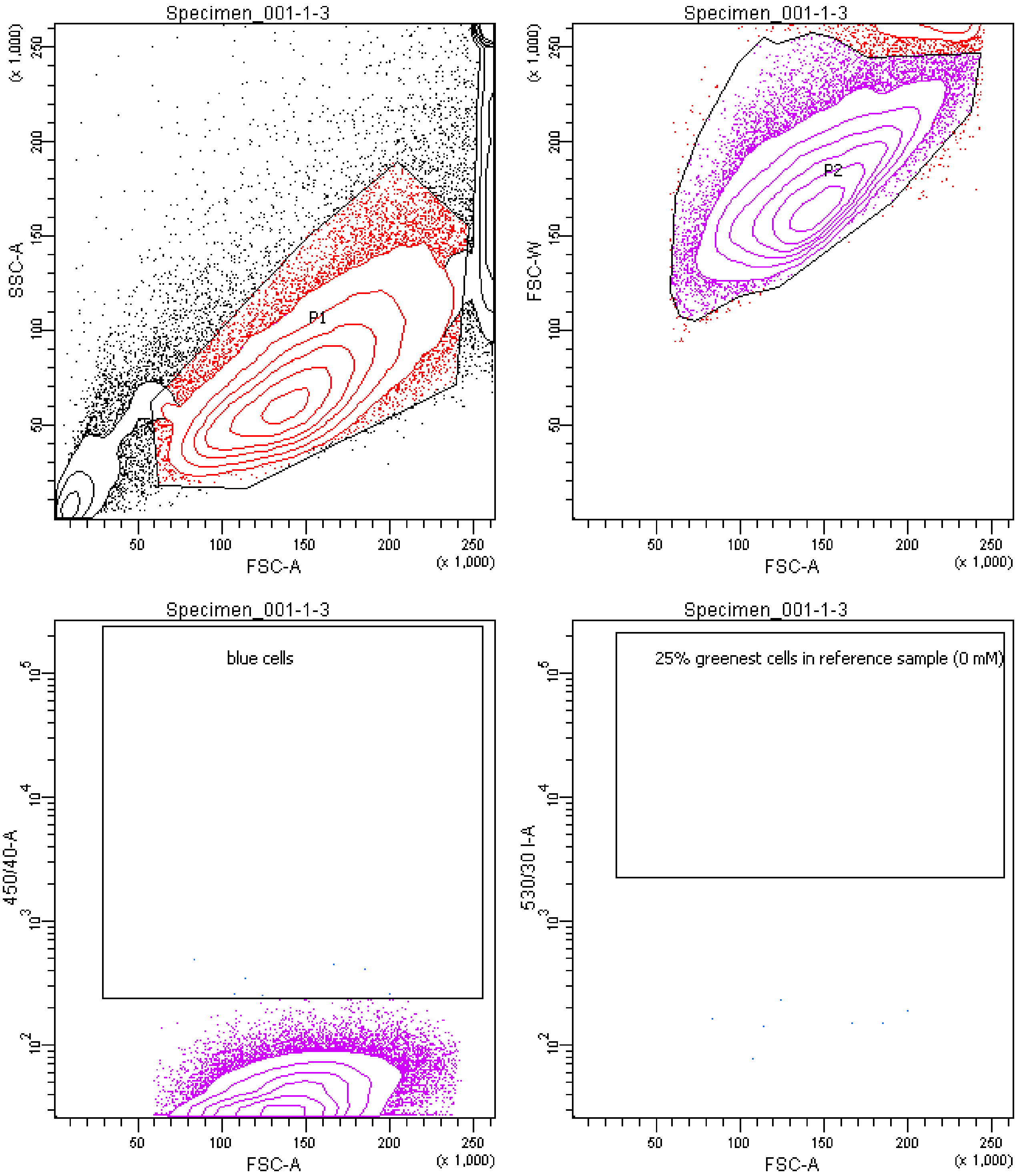

FACSDiva Version 6.1.3

Tube: 1-3

Population	#Events	%Parent	%Total
All Events	50,000	####	100.0
P1	33,664	67.3	67.3
P2	31,034	92.2	62.1
blue cells	7	0.0	0.0
25% greenest cells in reference sample (0 mM)	0	0.0	0.0

FACSDiva Version 6.1.3

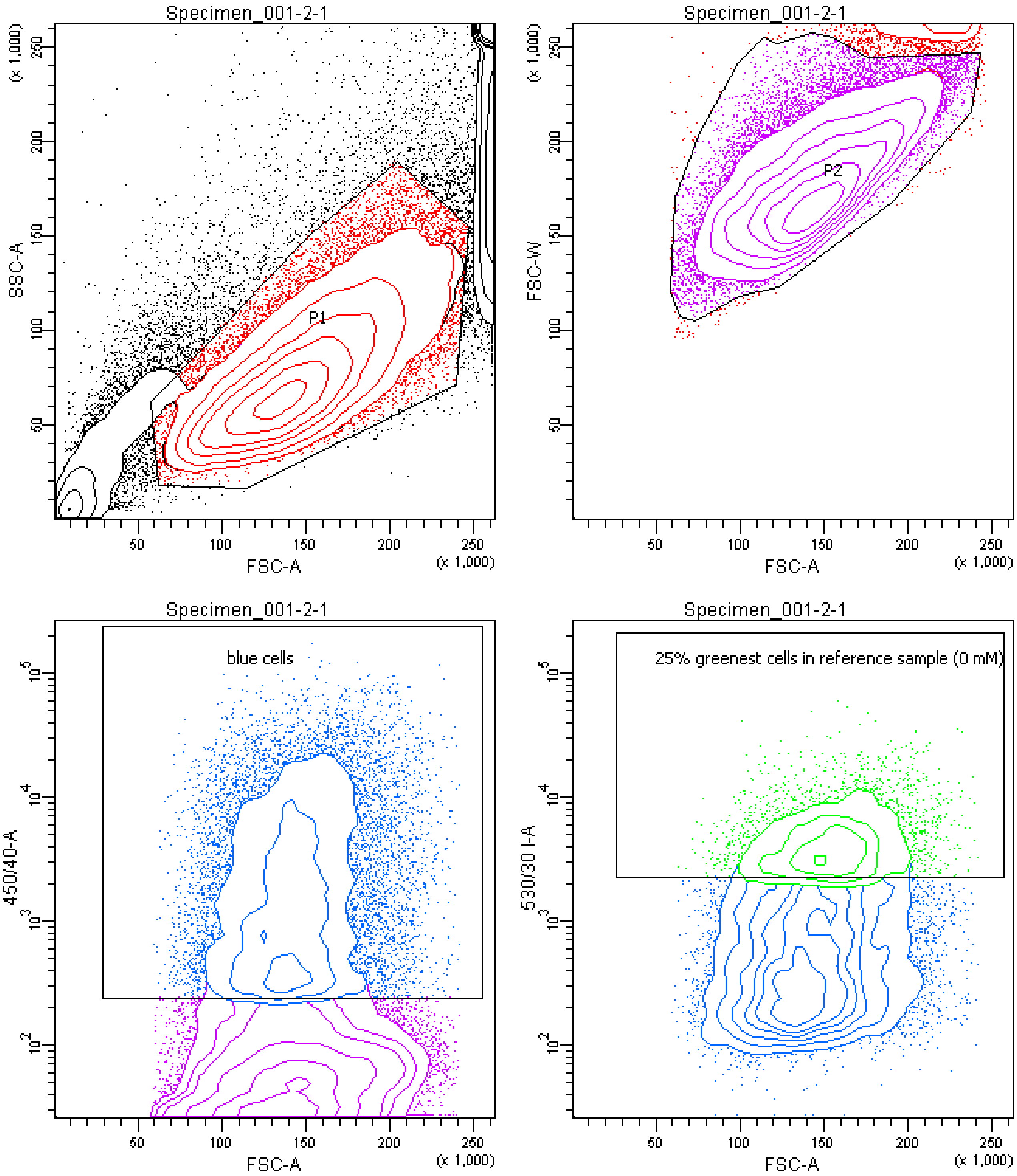

FACSDiva Version 6.1.3

Tube: 2-1

Population	#Events	%Parent	%Total
All Events	50,000	####	100.0
P1	33,280	66.6	66.6
P2	30,631	92.0	61.3
blue cells	10,907	35.6	21.8
25% greenest cells in reference sample (0 mM)	2,673	24.5	5.3

FACSDiva Version 6.1.3

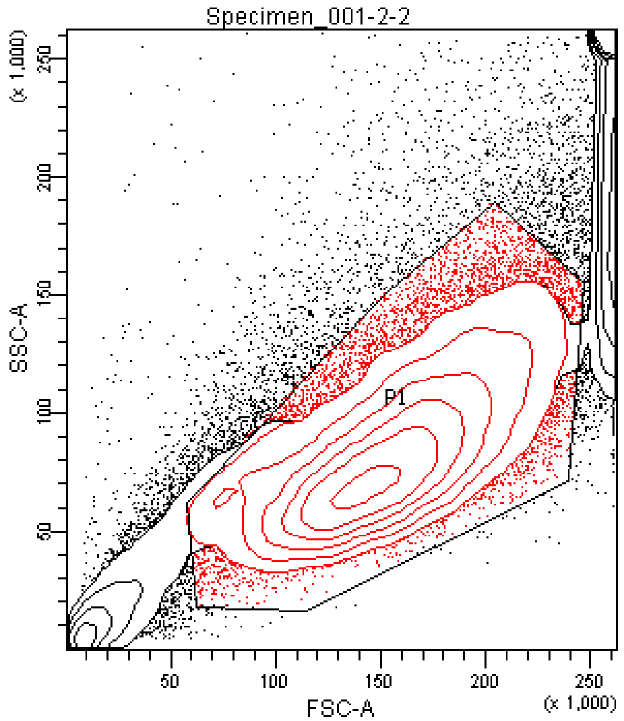

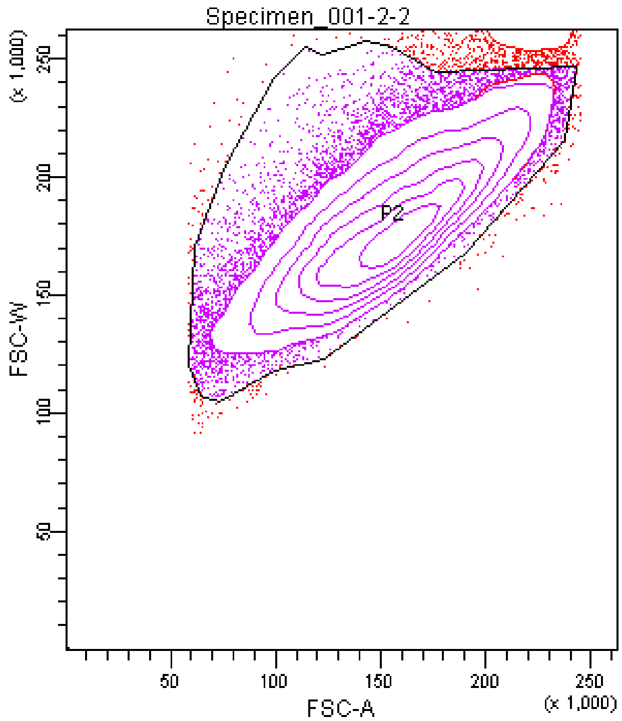

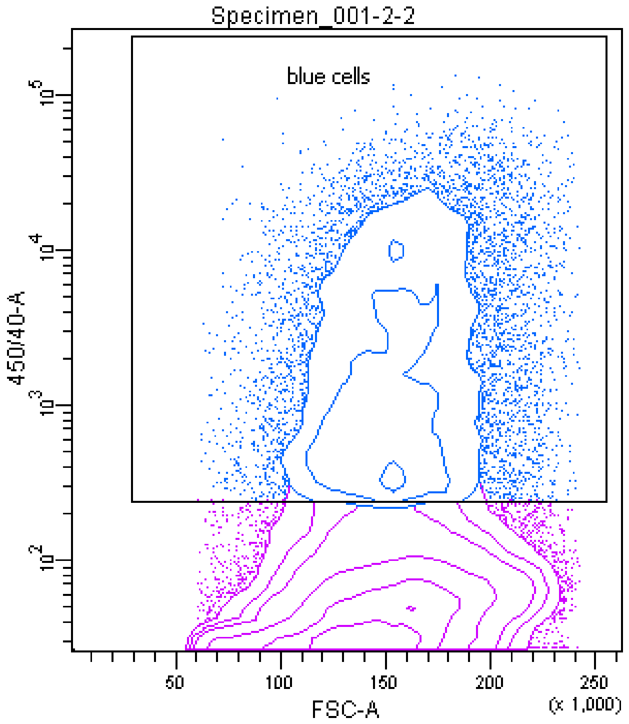

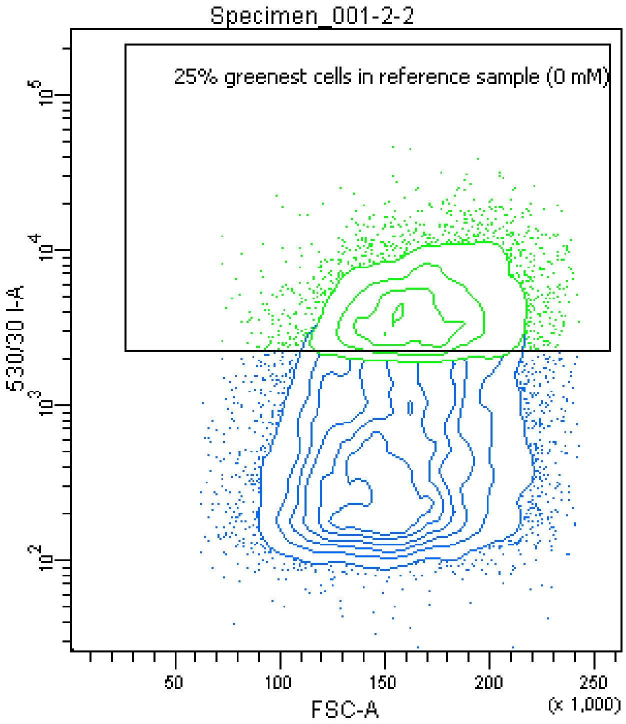

FACSDiva Version 6.1.3

Tube: 2-2

Population	#Events	%Parent	%Total
All Events	50,000	####	100.0
P1	31,672	63.3	63.3
P2	29,691	93.7	59.4
blue cells	10,287	34.6	20.6
25% greenest cells in reference sample (0 mM)	2,654	25.8	5.3

FACSDiva Version 6.1.3

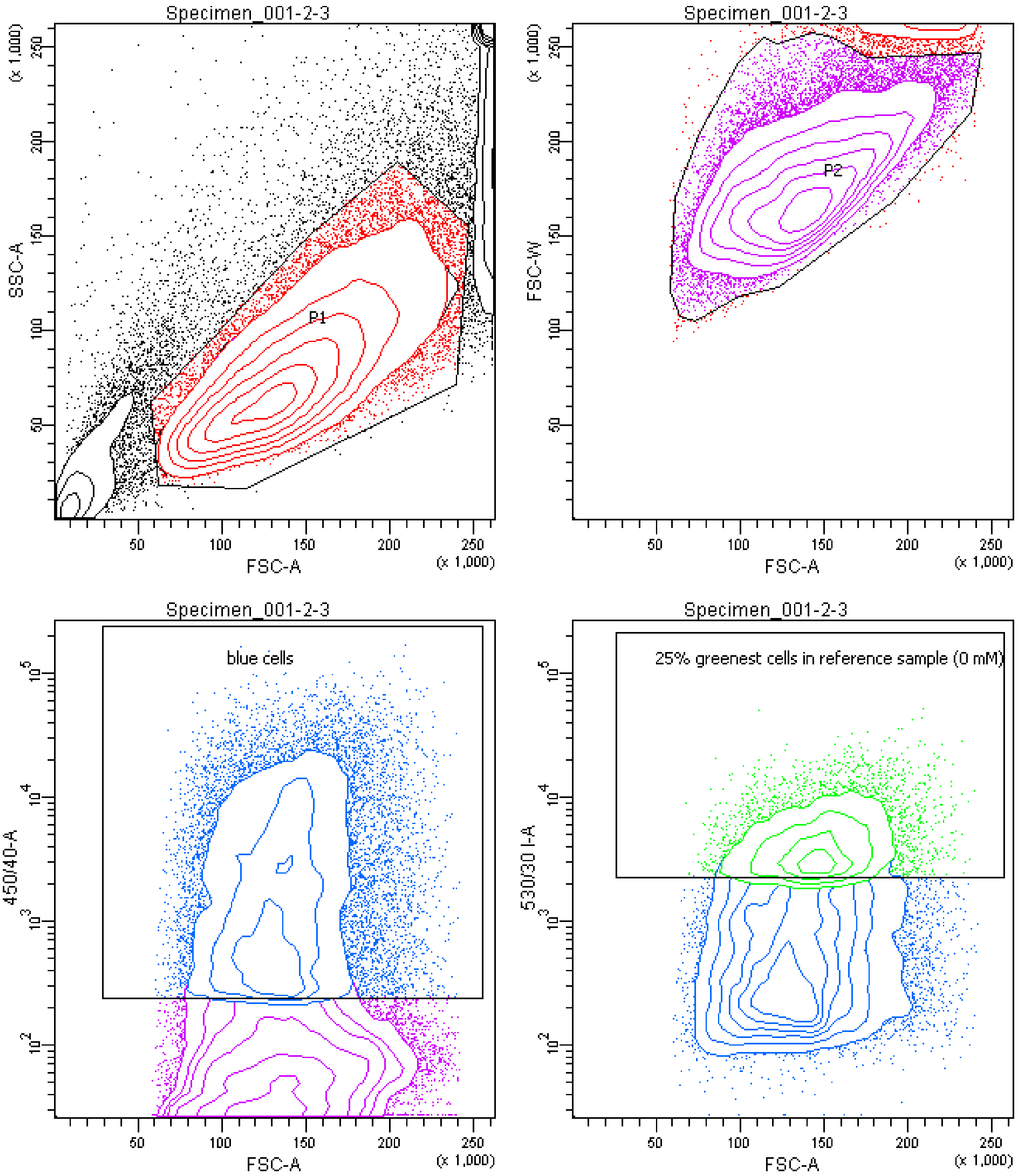

FACSDiva Version 6.1.3

Tube: 2-3

Population	#Events	%Parent	%Total
All Events	50,000	####	100.0
P1	36,315	72.6	72.6
P2	33,324	91.8	66.6
blue cells	13,576	40.7	27.2
25% greenest cells in reference sample (0 mM)	3,390	25.0	6.8

FACSDiva Version 6.1.3

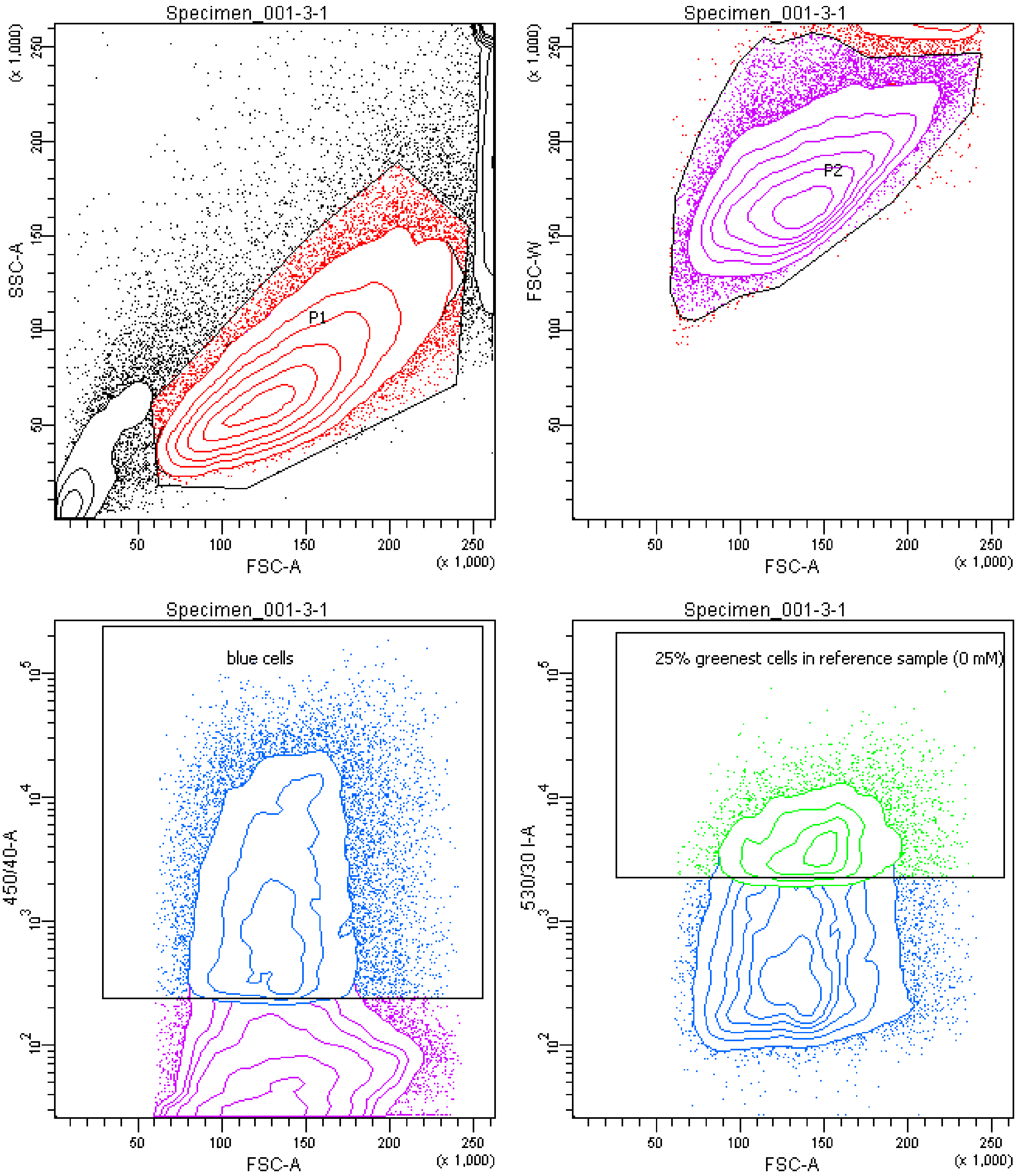

FACSDiva Version 6.1.3

Tube: 3-1

Population	#Events	%Parent	%Total
All Events	50,000	####	100.0
P1	36,487	73.0	73.0
P2	33,518	91.9	67.0
blue cells	13,858	41.3	27.7
25% greenest cells in reference sample (0 mM)	3,877	28.0	7.8

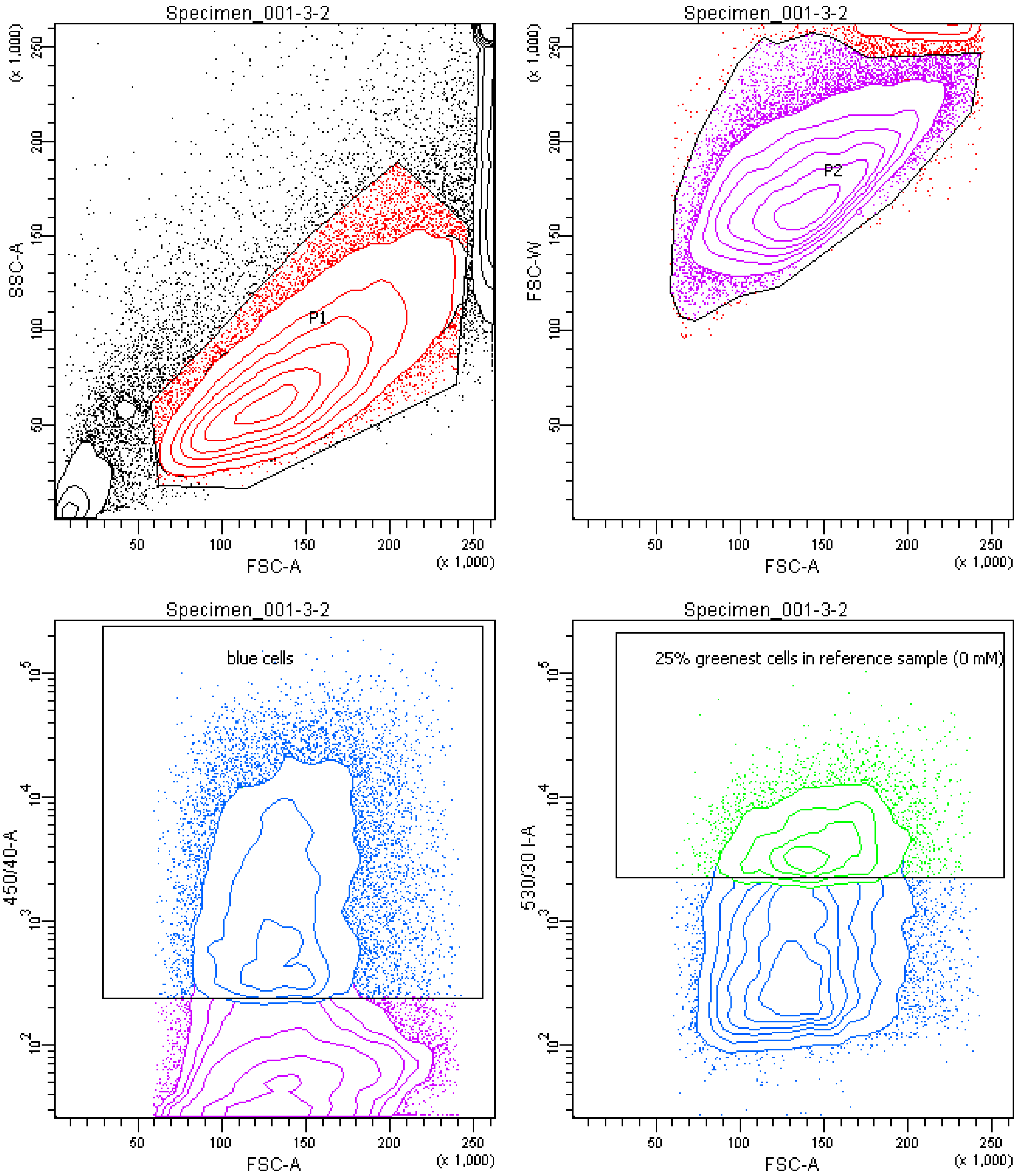

Specimen_001-3-2
SSC-A (x 1,000)
FSC-A (x 1,000)
P1
Specimen_001-3-2
FSC-W (x 1,000)
FSC-A (x 1,000)
P2
Specimen_001-3-2
450/40-A
FSC-A (x 1,000)
blue cells
Specimen_001-3-2
530/30 I-A
FSC-A (x 1,000)
25% greenest cells in reference sample (0 mM)

Tube: 3-2

Population	#Events	%Parent	%Total
All Events	50,000	####	100.0
P1	35,011	70.0	70.0
P2	31,748	90.7	63.5
blue cells	12,571	39.6	25.1
25% greenest cells in reference sample (0 mM)	3,568	28.4	7.1

FACSDiva Version 6.1.3

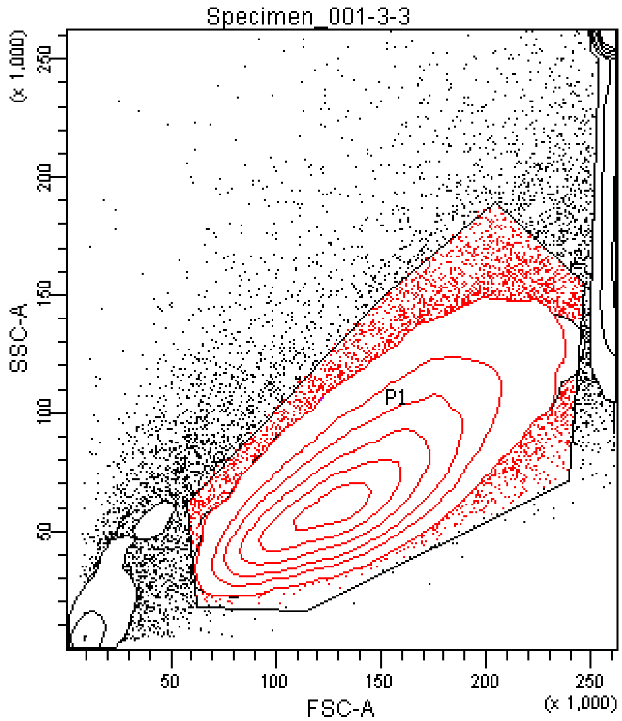

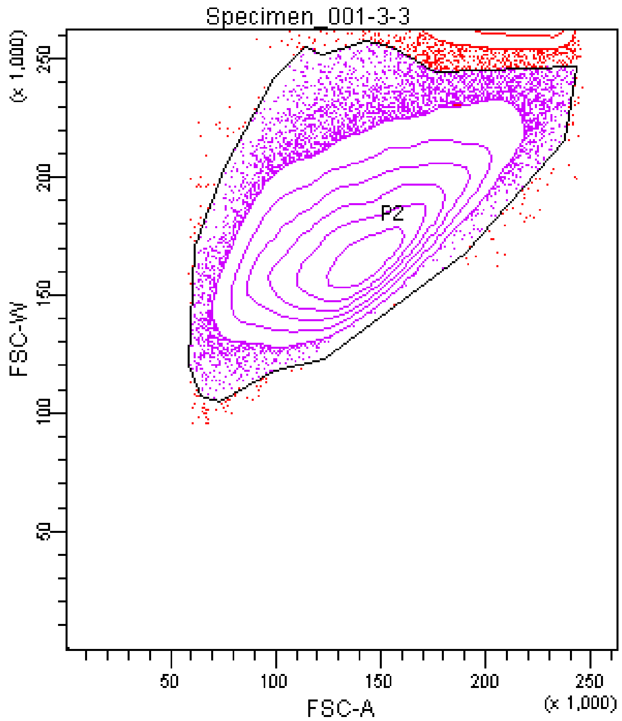

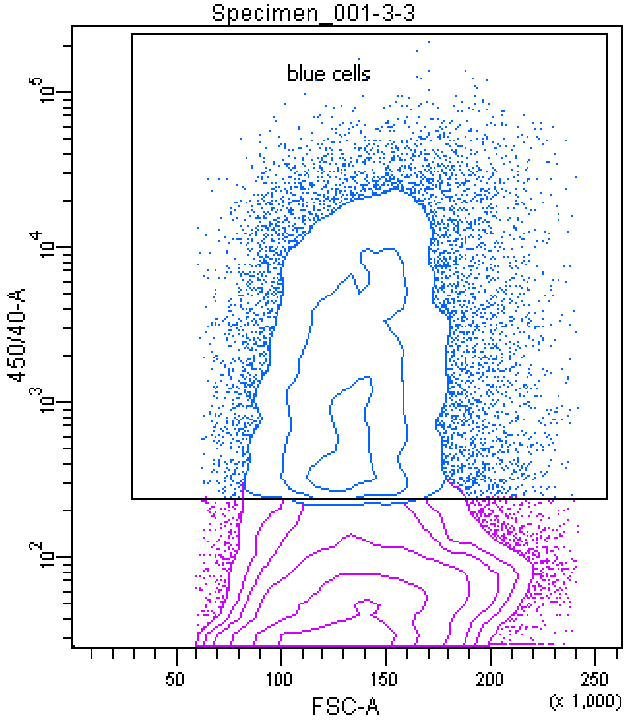

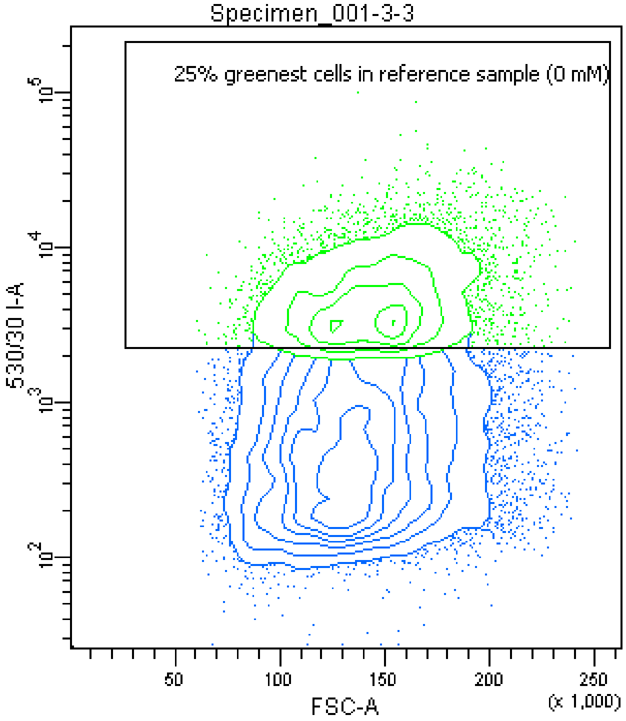

Tube: 3-3

Population	#Events	%Parent	%Total
All Events	50,000	####	100.0
P1	36,508	73.0	73.0
P2	33,599	92.0	67.2
blue cells	12,999	38.7	26.0
25% greenest cells in reference sample (0 mM)	3,584	27.6	7.2

FACSDiva Version 6.1.3

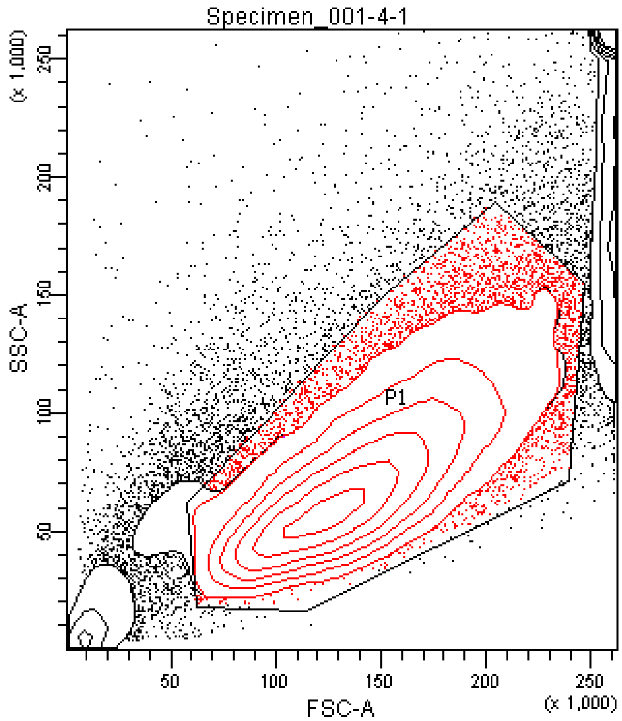

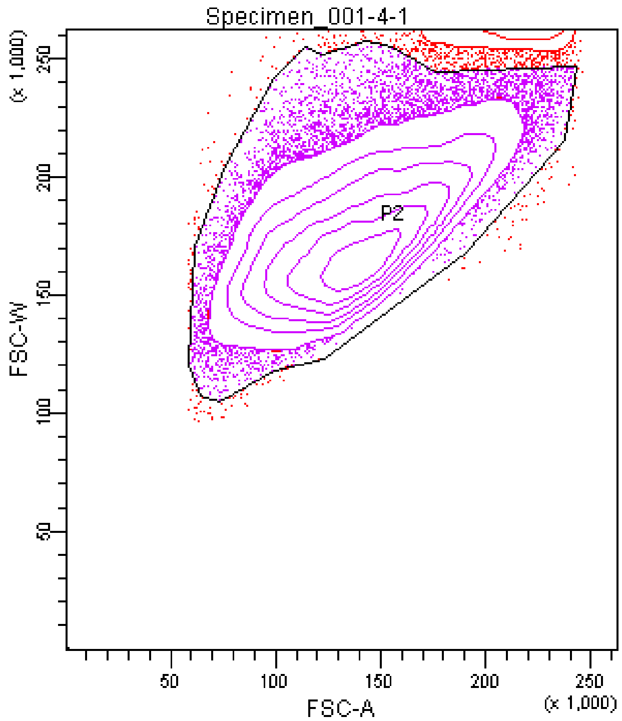

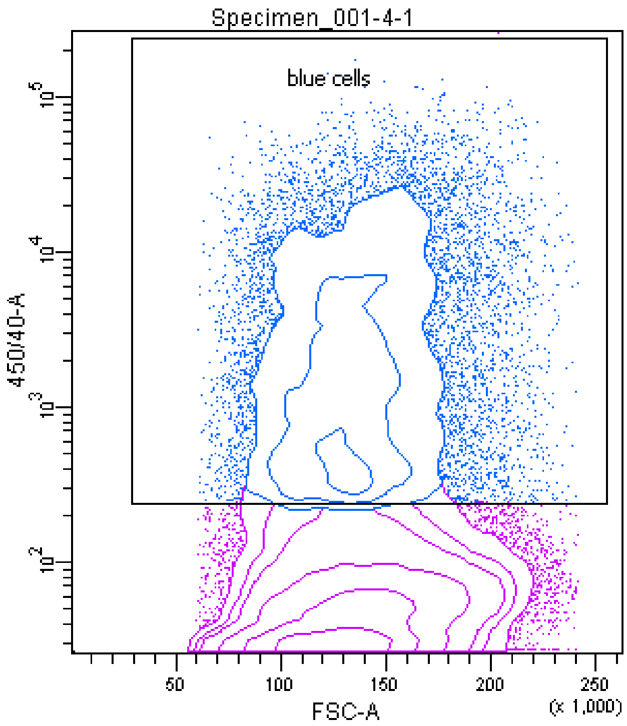

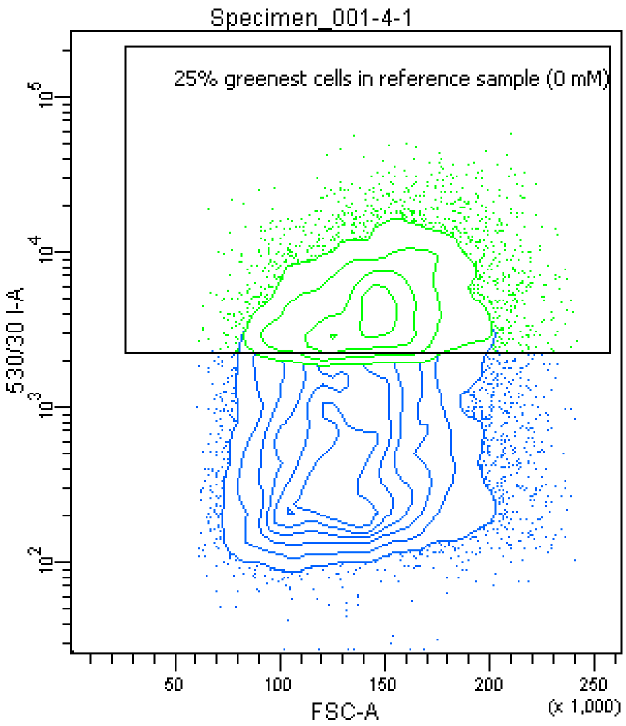

Tube: 4-1

Population	#Events	%Parent	%Total
All Events	50,000	####	100.0
P1	35,979	72.0	72.0
P2	32,951	91.6	65.9
blue cells	11,813	35.9	23.6
25% greenest cells in reference sample (0 mM)	3,613	30.6	7.2

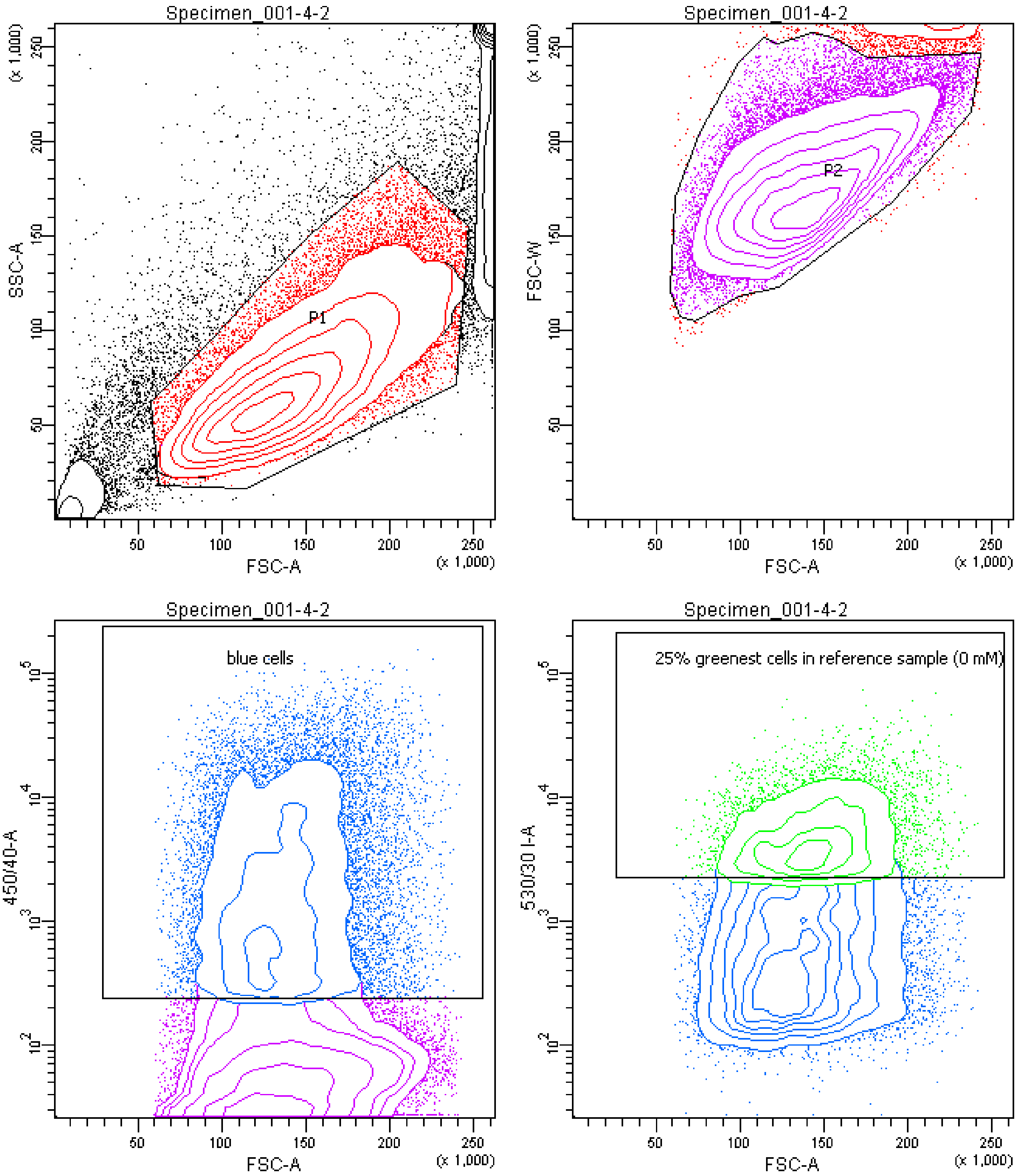
Specimen_001-4-2
SSC-A
(x 1,000)
FSC-A
(x 1,000)
P1
Specimen_001-4-2
FSC-W
(x 1,000)
FSC-A
(x 1,000)
P2
Specimen_001-4-2
450/40-A
FSC-A
(x 1,000)
blue cells
Specimen_001-4-2
530/30 I-A
FSC-A
(x 1,000)
25% greenest cells in reference sample (0 mM)

Tube: 4-2

Population	#Events	%Parent	%Total
All Events	50,000	####	100.0
P1	38,381	76.8	76.8
P2	35,430	92.3	70.9
blue cells	13,042	36.8	26.1
25% greenest cells in reference sample (0 mM)	3,835	29.4	7.7

FACSDiva Version 6.1.3

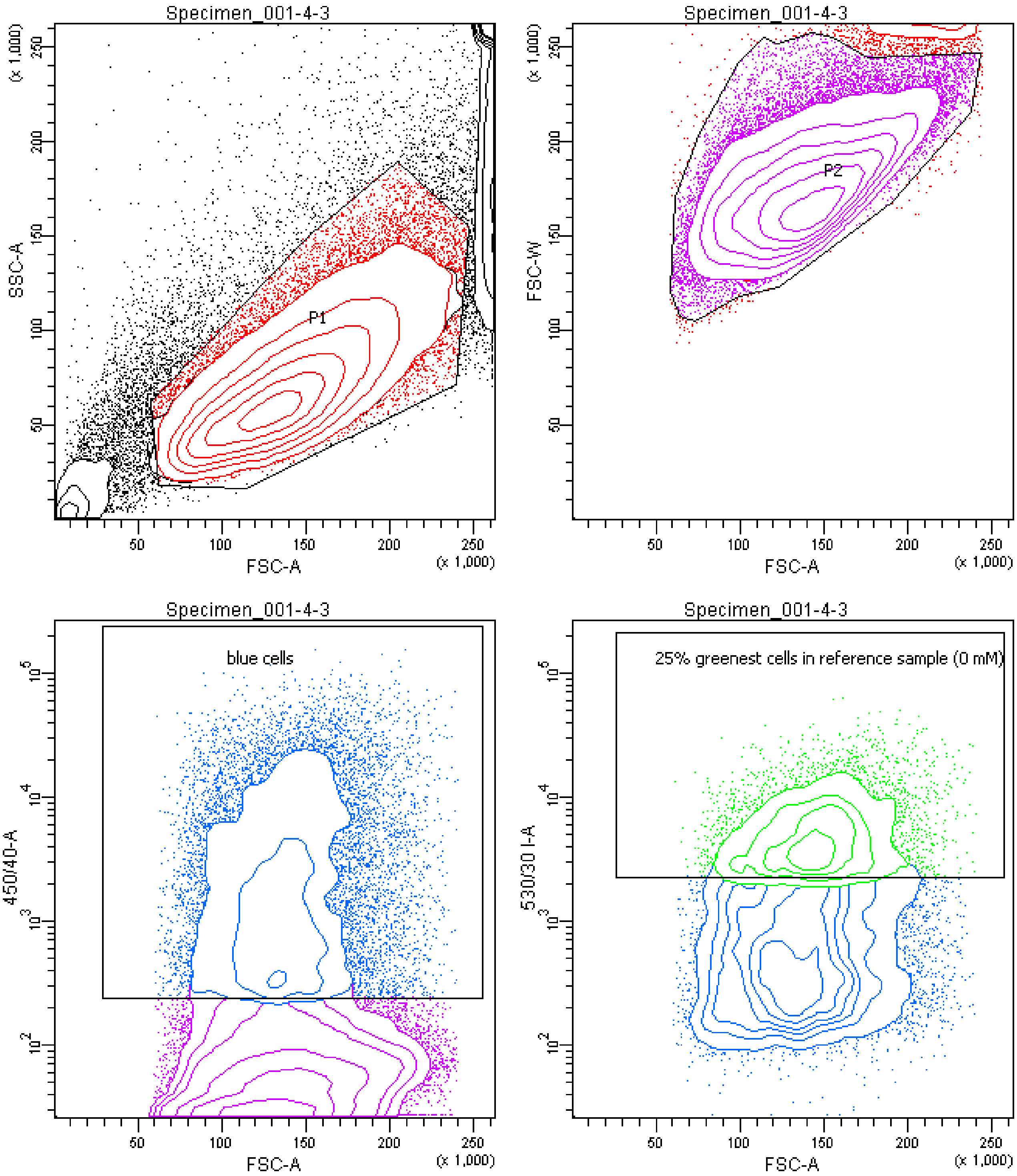

FACSDiva Version 6.1.3

Tube: 4-3

Population	#Events	%Parent	%Total
All Events	50,000	####	100.0
P1	36,844	73.7	73.7
P2	34,075	92.5	68.2
blue cells	11,426	33.5	22.9
25% greenest cells in reference sample (0 mM)	3,406	29.8	6.8

FACSDiva Version 6.1.3

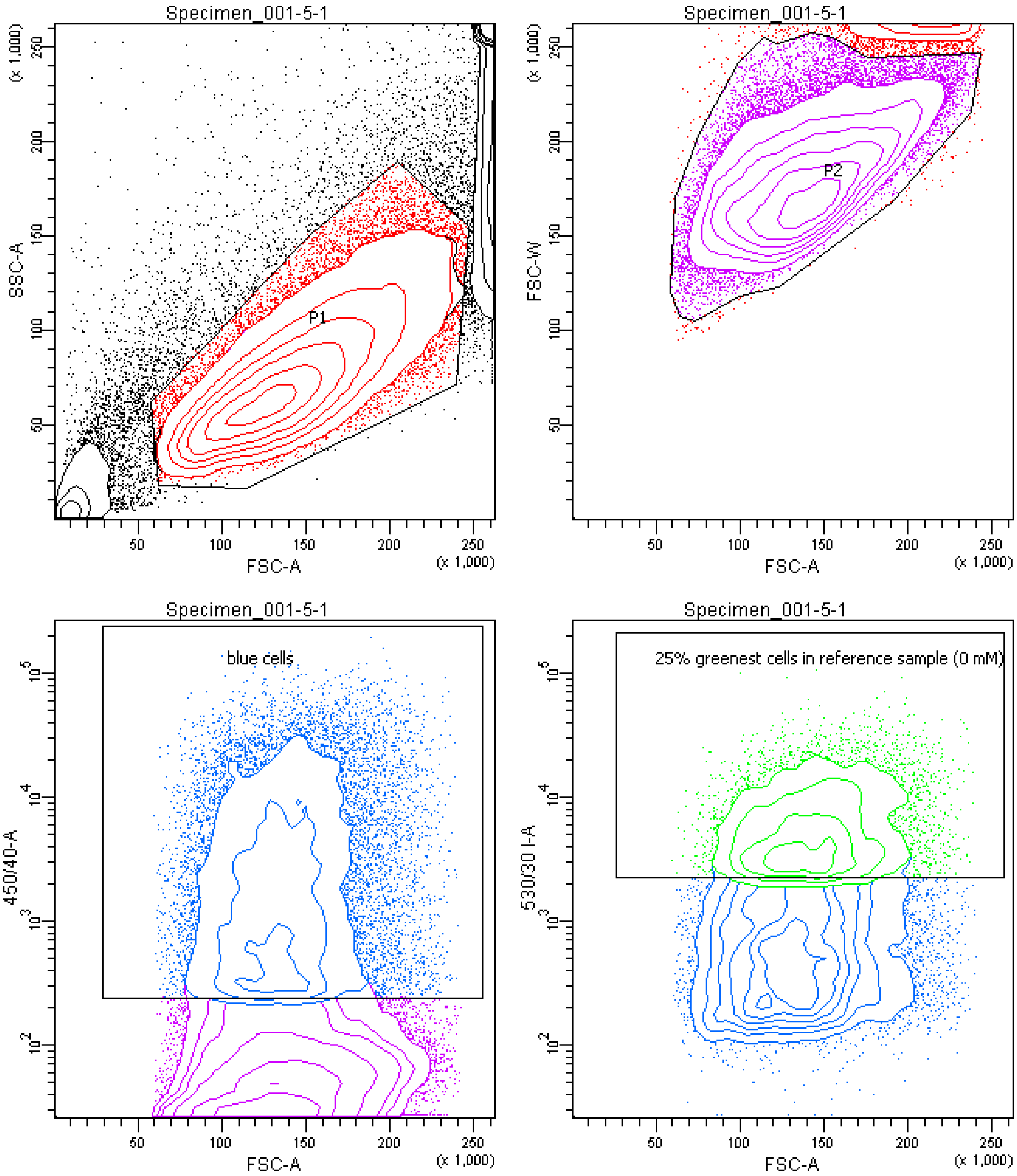

Tube: 5-1

Population	#Events	%Parent	%Total
All Events	50,000	####	100.0
P1	35,595	71.2	71.2
P2	32,114	90.2	64.2
blue cells	12,533	39.0	25.1
25% greenest cells in reference sample (0 mM)	4,349	34.7	8.7

FACSDiva Version 6.1.3

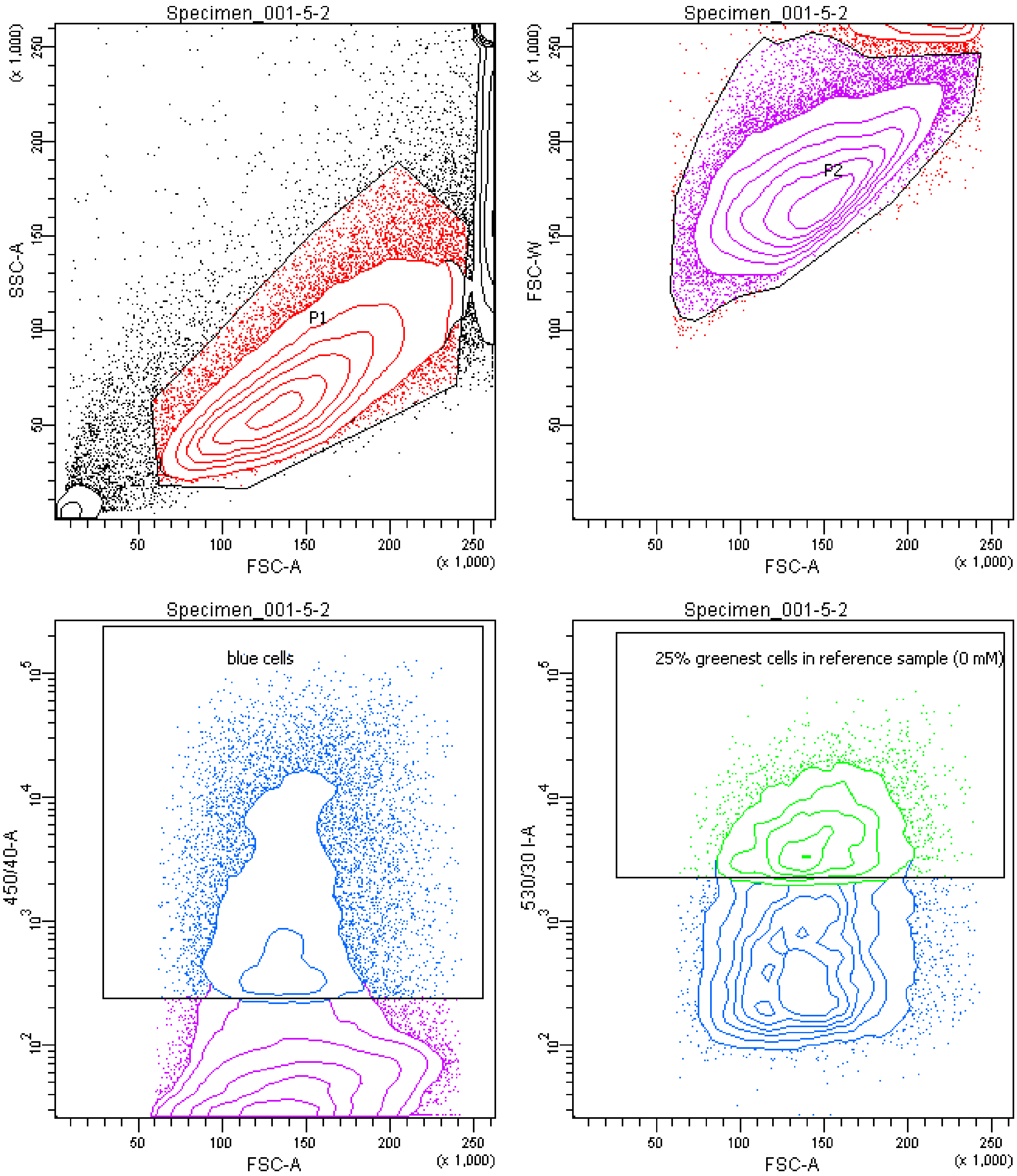

FACSDiva Version 6.1.3

Tube: 5-2

Population	#Events	%Parent	%Total
All Events	50,000	####	100.0
P1	34,256	68.5	68.5
P2	30,755	89.8	61.5
blue cells	8,173	26.6	16.3
25% greenest cells in reference sample (0 mM)	2,589	31.7	5.2

FACSDiva Version 6.1.3

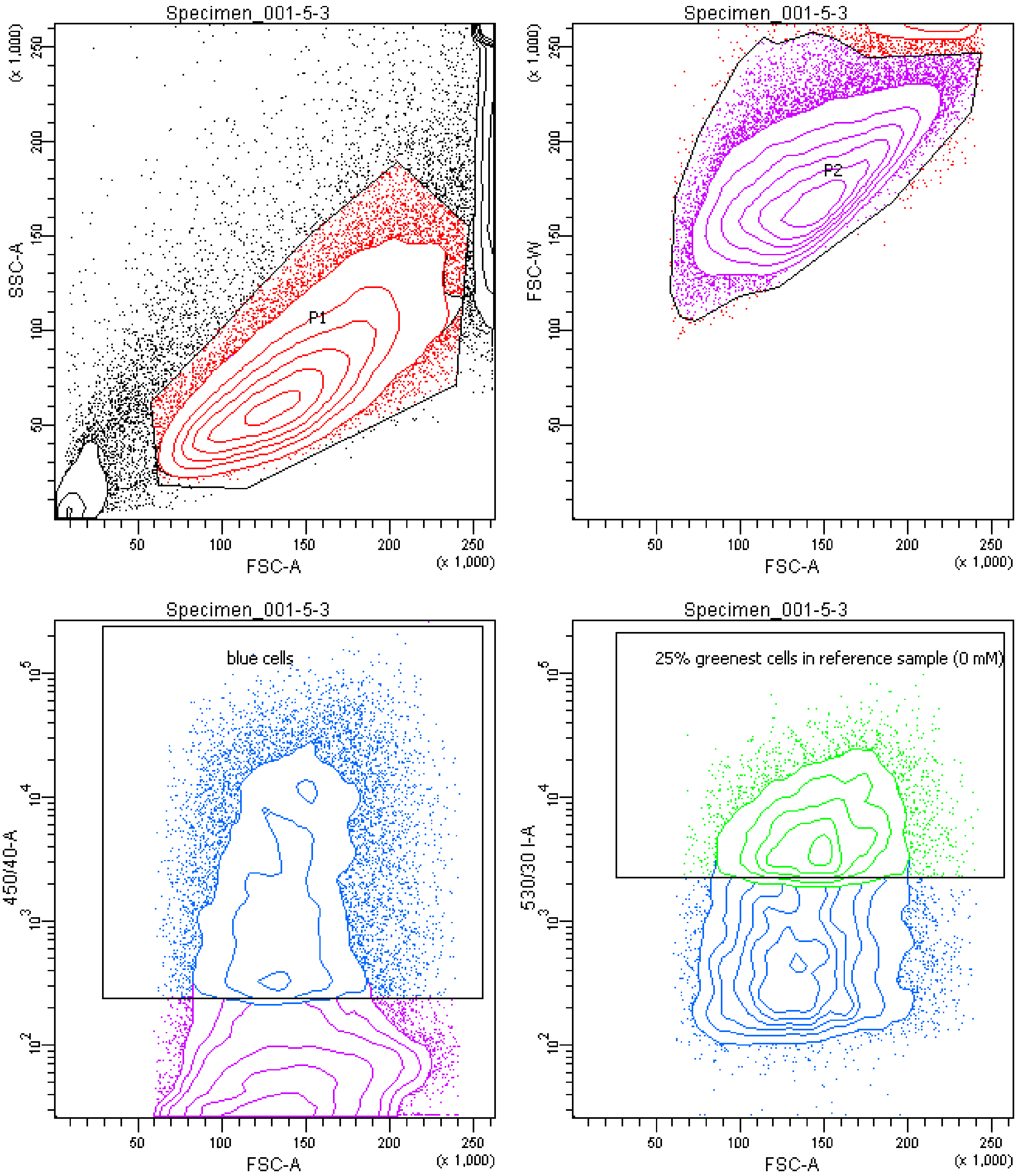

FACSDiva Version 6.1.3

Tube: 5-3

Population	#Events	%Parent	%Total
All Events	50,000	####	100.0
P1	35,350	70.7	70.7
P2	32,451	91.8	64.9
blue cells	11,486	35.4	23.0
25% greenest cells in reference sample (0 mM)	4,080	35.5	8.2

FACSDiva Version 6.1.3

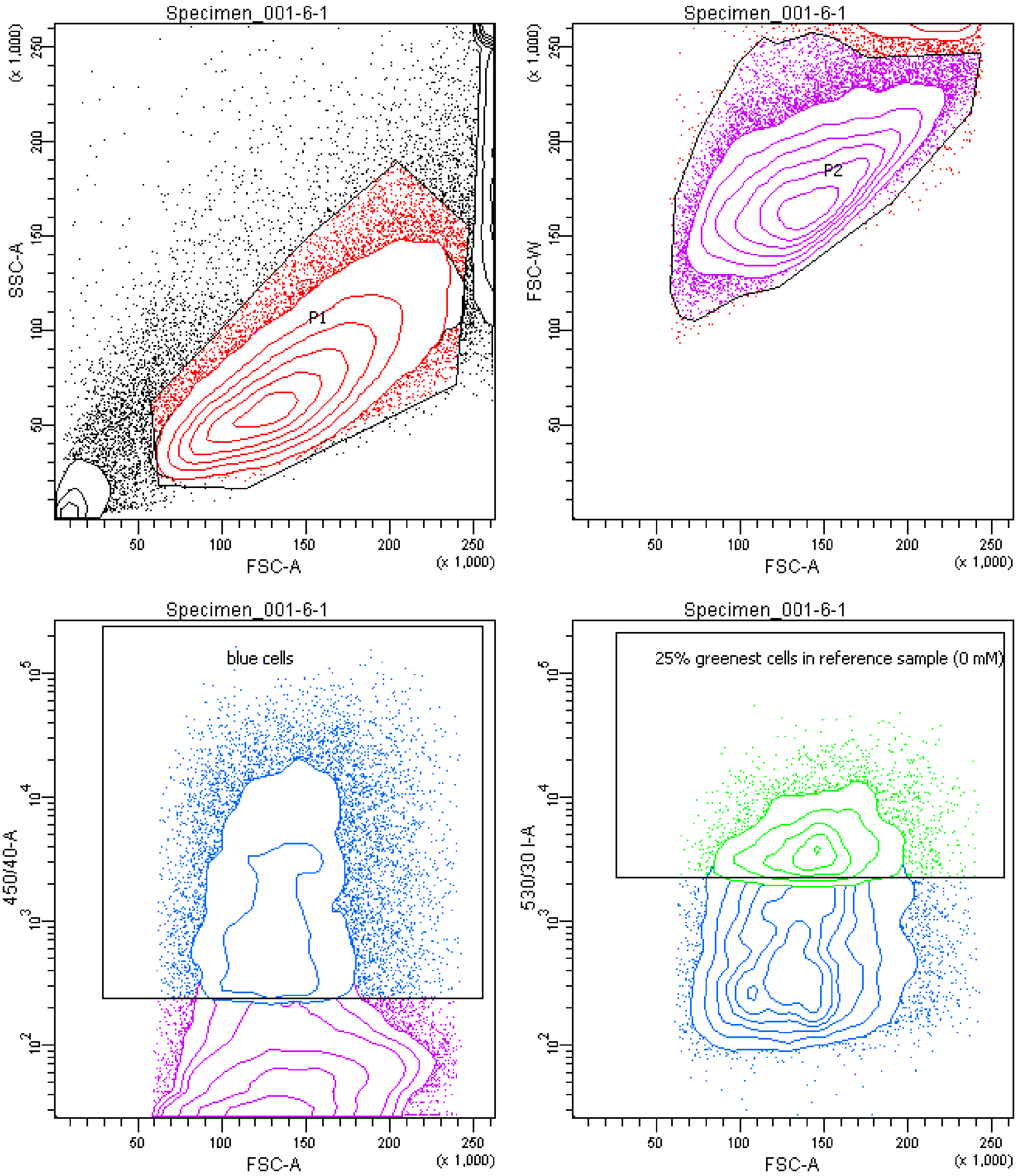

FACSDiva Version 6.1.3

Tube: 6-1

Population	#Events	%Parent	%Total
All Events	50,000	####	100.0
P1	36,819	73.6	73.6
P2	33,834	91.9	67.7
blue cells	11,301	33.4	22.6
25% greenest cells in reference sample (0 mM)	3,139	27.8	6.3

FACSDiva Version 6.1.3

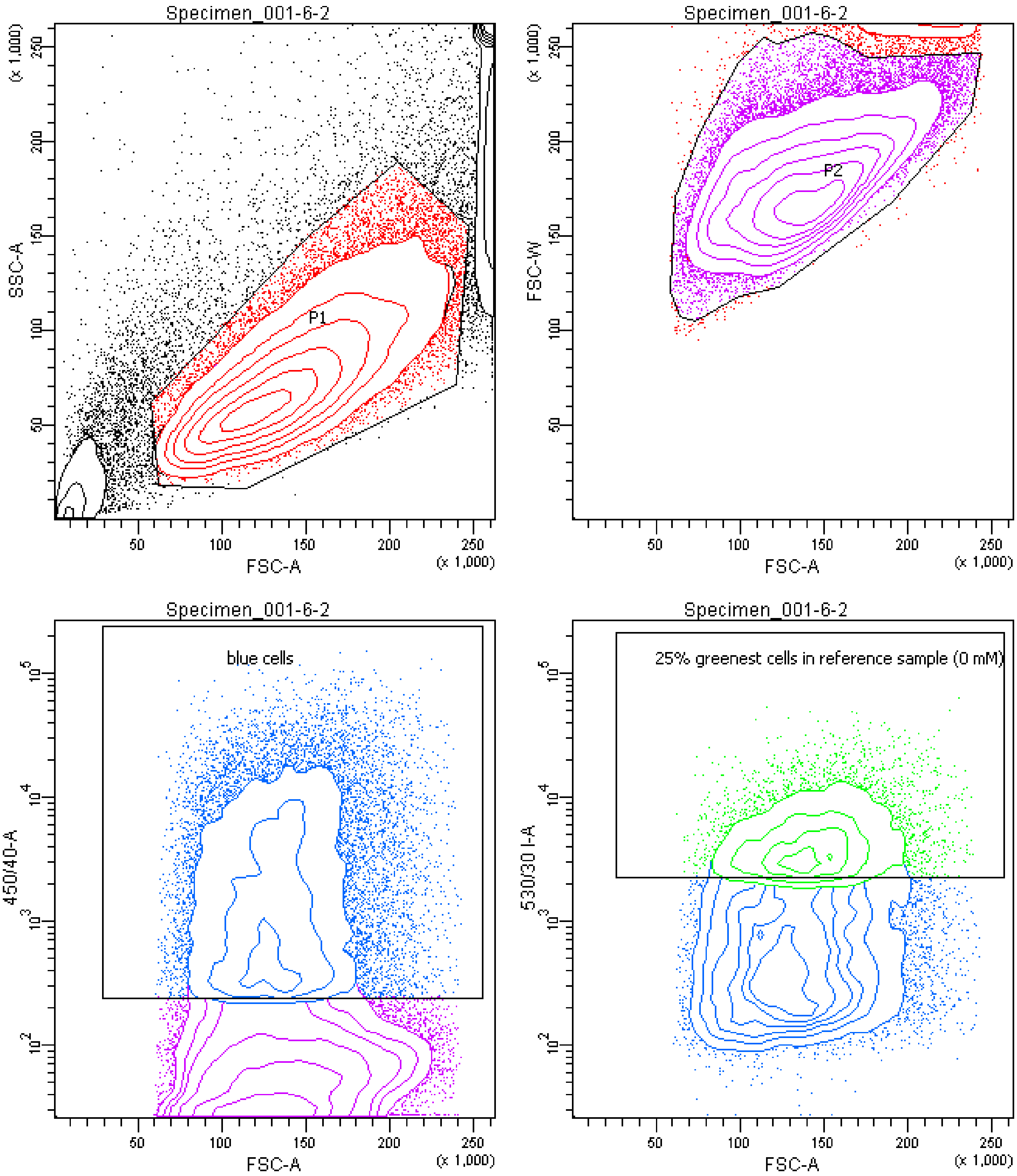

FACSDiva Version 6.1.3

Tube: 6-2

Population	#Events	%Parent	%Total
All Events	50,000	####	100.0
P1	38,056	76.1	76.1
P2	35,187	92.5	70.4
blue cells	13,360	38.0	26.7
25% greenest cells in reference sample (0 mM)	3,759	28.1	7.5

FACSDiva Version 6.1.3

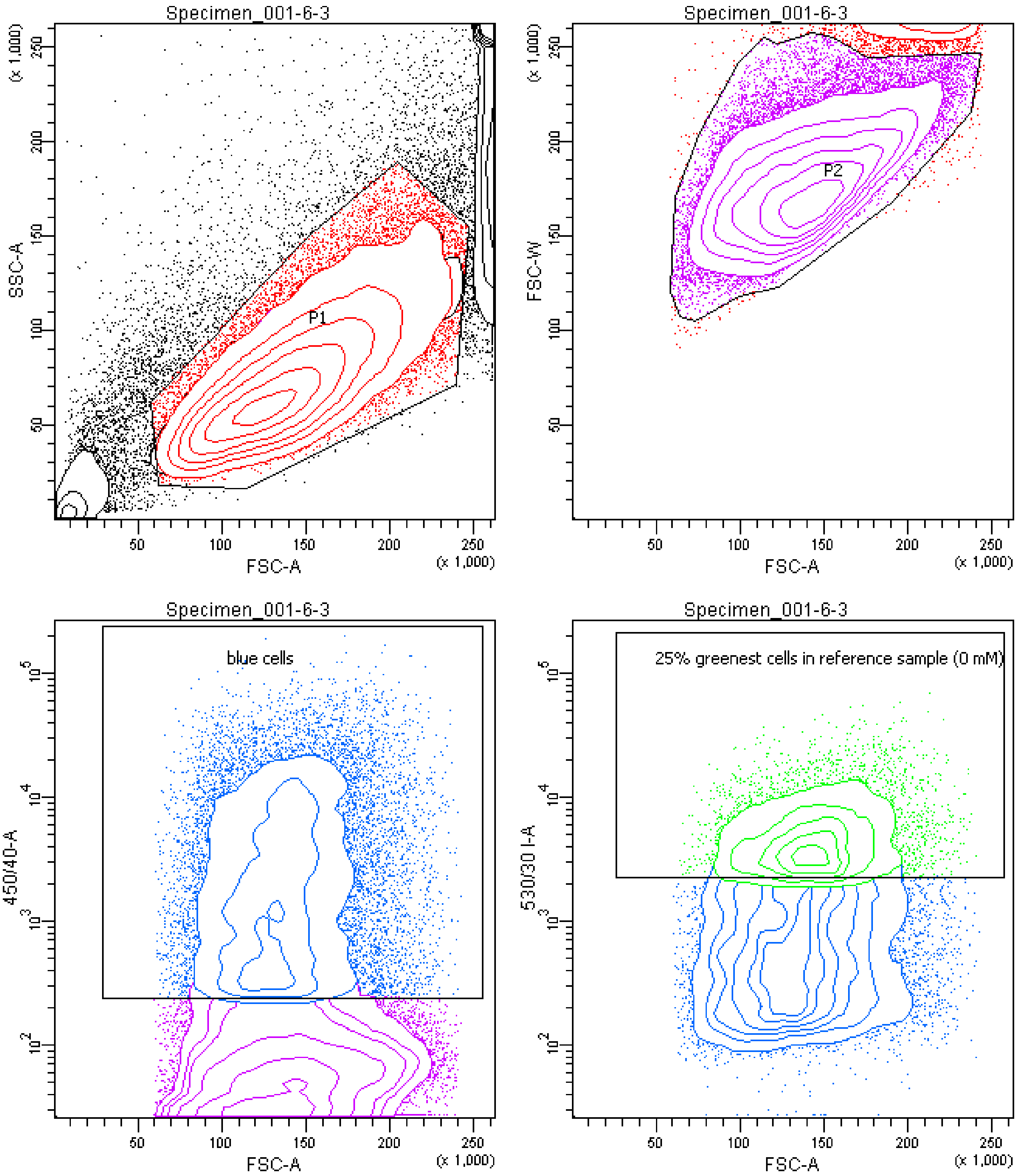

FACSDiva Version 6.1.3

Tube: 6-3

Population	#Events	%Parent	%Total
All Events	50,000	####	100.0
P1	35,671	71.3	71.3
P2	32,645	91.5	65.3
blue cells	12,535	38.4	25.1
25% greenest cells in reference sample (0 mM)	3,769	30.1	7.5

FACSDiva Version 6.1.3

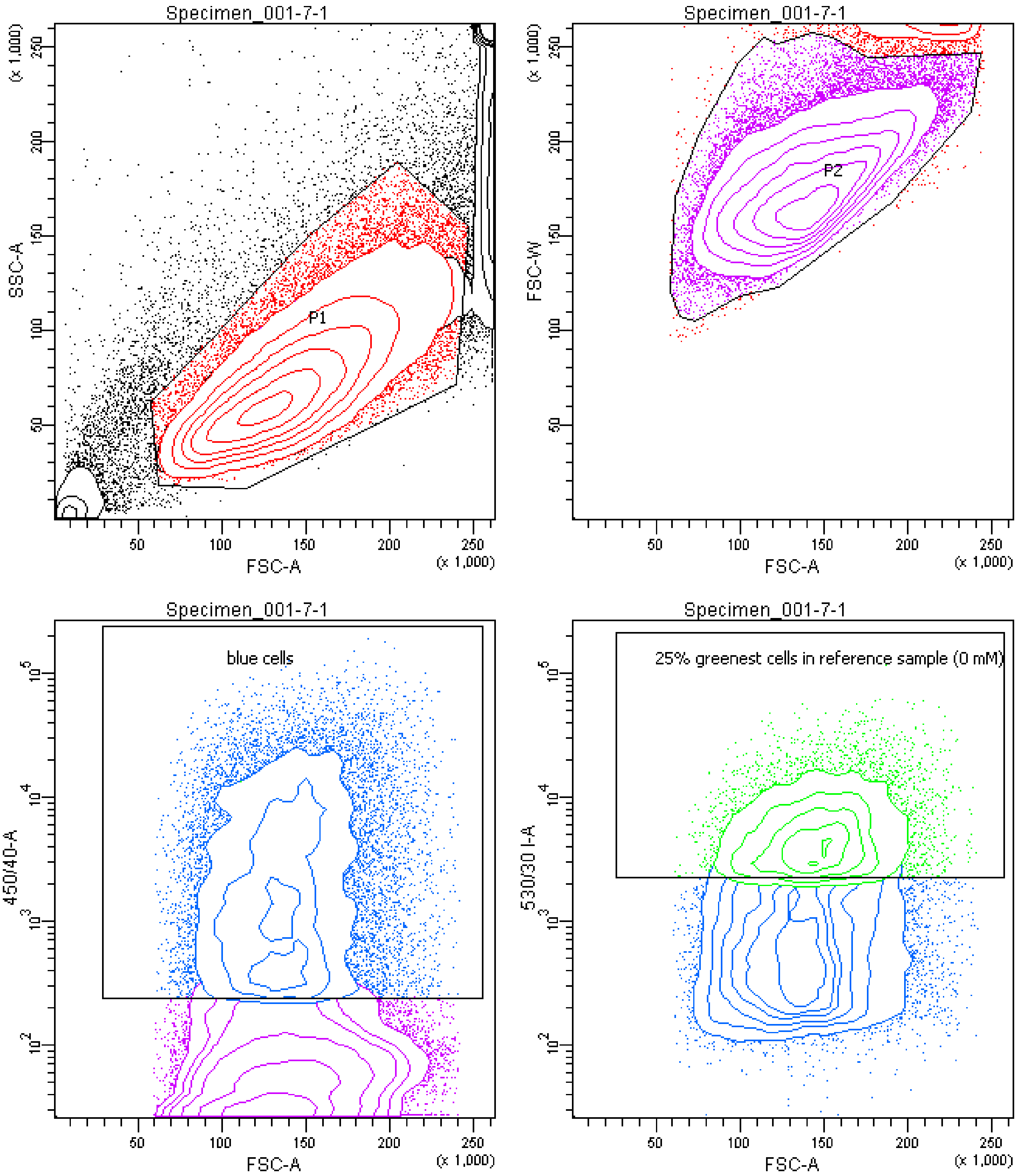

Tube: 7-1

Population	#Events	%Parent	%Total
All Events	50,000	####	100.0
P1	35,214	70.4	70.4
P2	32,074	91.1	64.1
blue cells	12,629	39.4	25.3
25% greenest cells in reference sample (0 mM)	4,300	34.0	8.6

FACSDiva Version 6.1.3

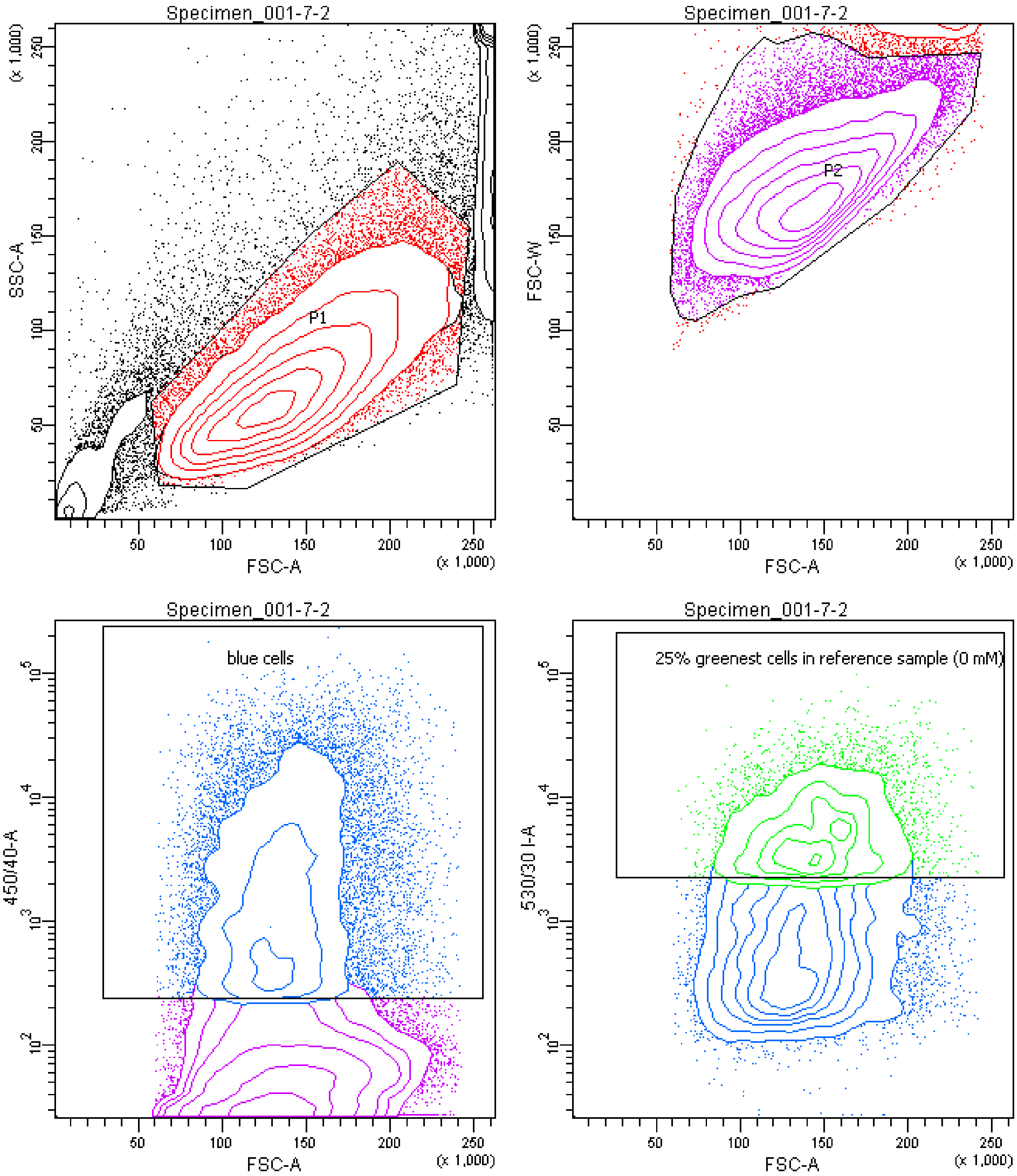

Tube: 7-2

Population	#Events	%Parent	%Total
All Events	50,000	####	100.0
P1	36,968	73.9	73.9
P2	34,266	92.7	68.5
blue cells	12,362	36.1	24.7
25% greenest cells in reference sample (0 mM)	4,145	33.5	8.3

FACSDiva Version 6.1.3

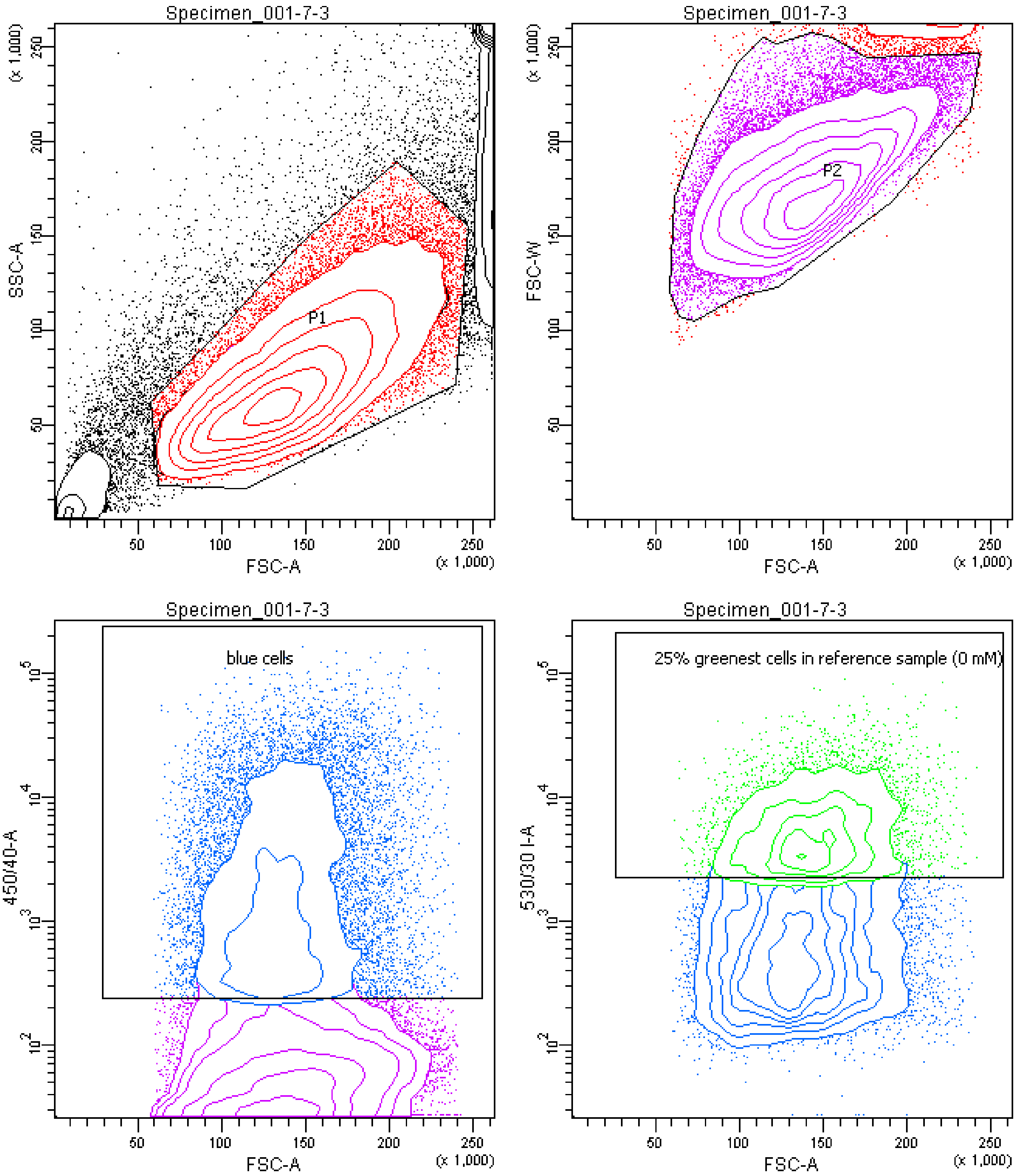

FACSDiva Version 6.1.3

Tube: 7-3

Population	#Events	%Parent	%Total
All Events	50,000	####	100.0
P1	37,669	75.3	75.3
P2	34,931	92.7	69.9
blue cells	11,294	32.3	22.6
25% greenest cells in reference sample (0 mM)	3,698	32.7	7.4

FACSDiva Version 6.1.3

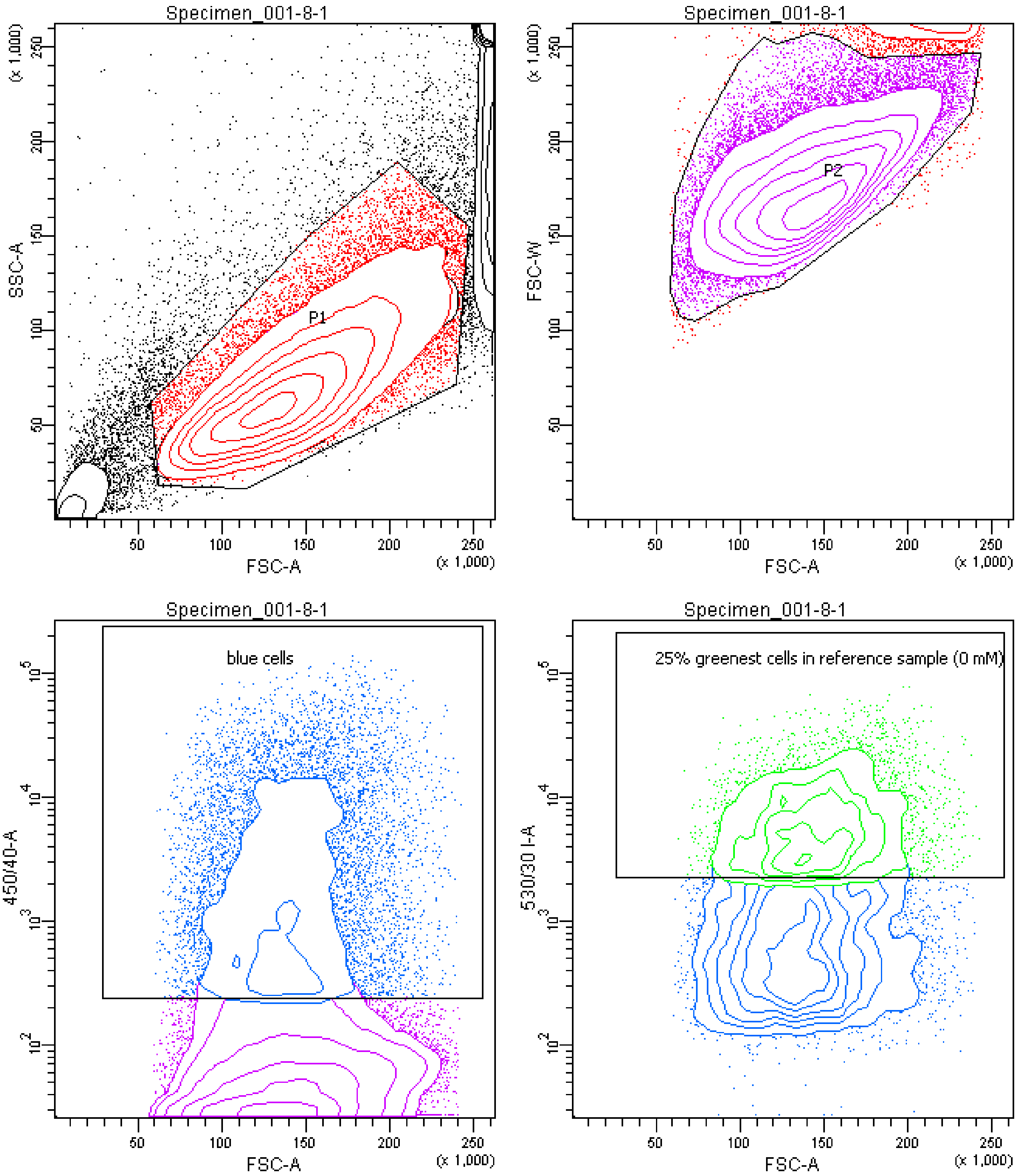

FACSDiva Version 6.1.3

Tube: 8-1

Population	#Events	%Parent	%Total
All Events	50,000	####	100.0
P1	35,017	70.0	70.0
P2	32,068	91.6	64.1
blue cells	8,702	27.1	17.4
25% greenest cells in reference sample (0 mM)	3,209	36.9	6.4

FACSDiva Version 6.1.3

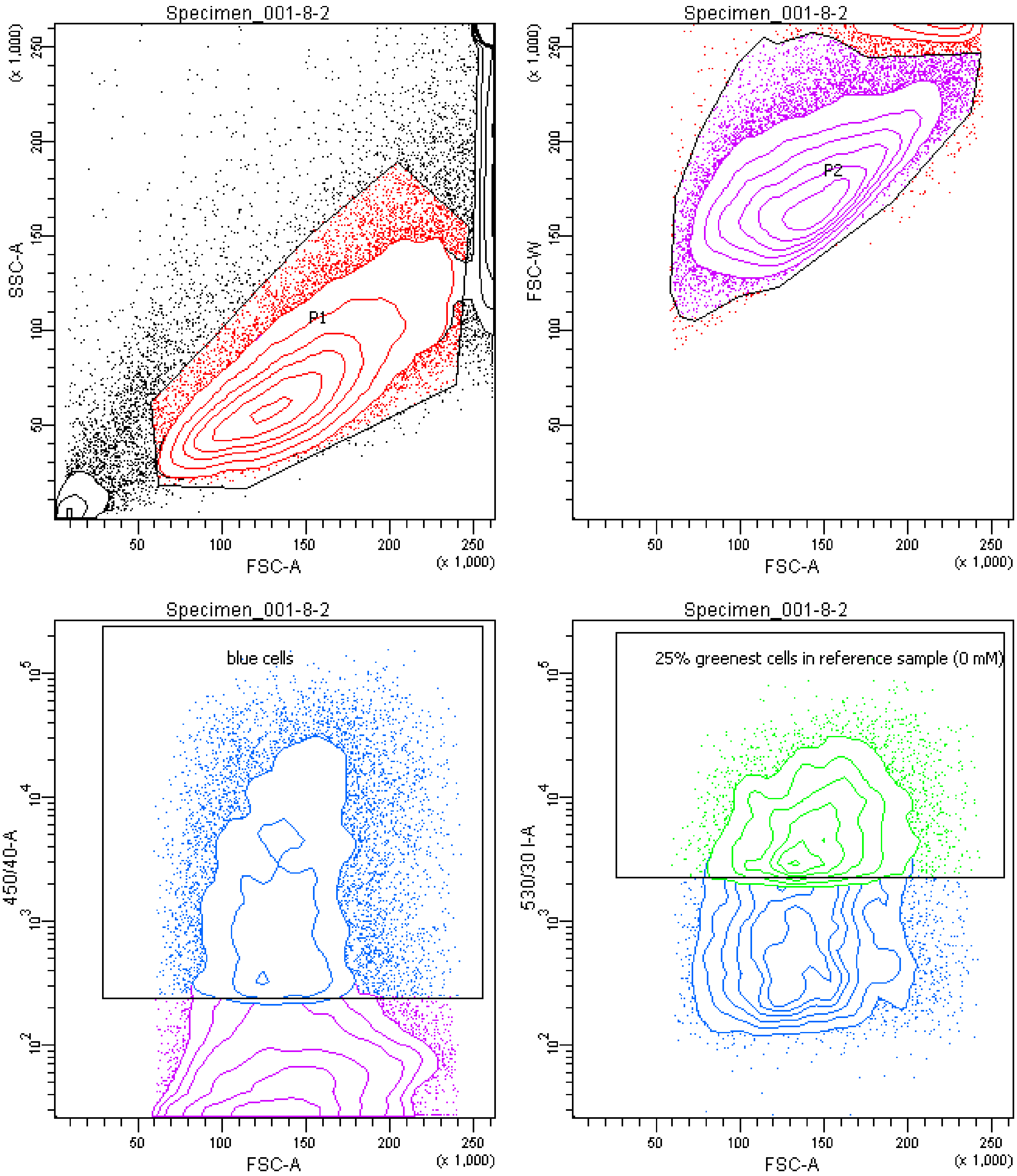

FACSDiva Version 6.1.3

Tube: 8-2

Population	#Events	%Parent	%Total
All Events	50,000	####	100.0
P1	31,439	62.9	62.9
P2	28,428	90.4	56.9
blue cells	9,660	34.0	19.3
25% greenest cells in reference sample (0 mM)	3,882	40.2	7.8

FACSDiva Version 6.1.3

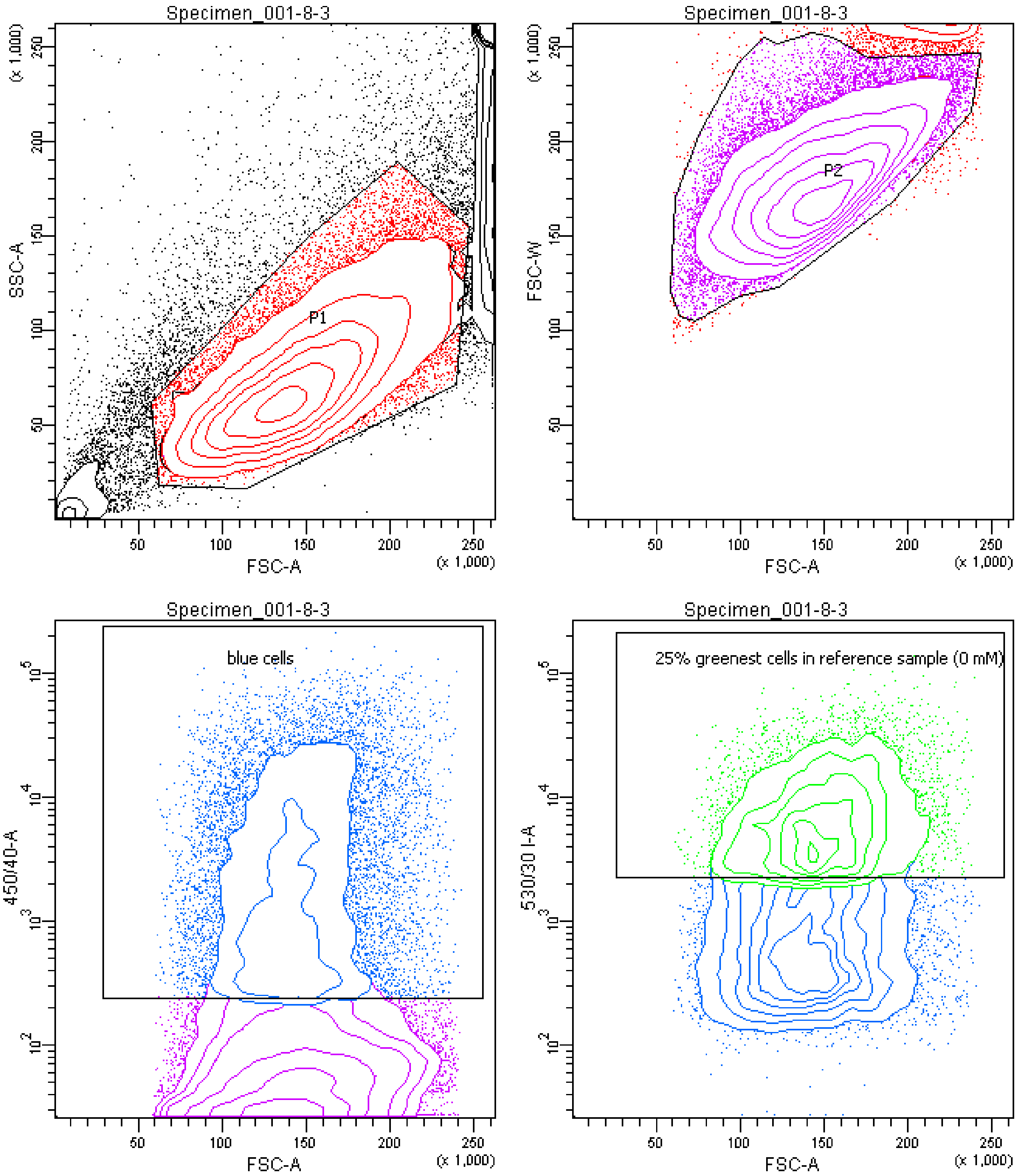

FACSDiva Version 6.1.3

Tube: 8-3

Population	#Events	%Parent	%Total
All Events	50,000	####	100.0
P1	33,953	67.9	67.9
P2	31,107	91.6	62.2
blue cells	10,177	32.7	20.4
25% greenest cells in reference sample (0 mM)	4,344	42.7	8.7

Click here to go back to the Table of Content
