# Supplementary material for: Non-invasive and high-throughput interrogation of exon-specific isoform expression
Source: Nat Cell Biol. 2021 Jun 3;23(6):652–63. doi: 10.1038/s41556-021-00678-x (PMC8189919; doi:10.1038/s41556-021-00678-x)
Supplement: Supplementary file 2 — Reporting Summary [file 41556_2021_678_MOESM2_ESM.pdf]

nature research

Corresponding author(s): Gil Gregor Westmeyer

Last updated by author(s): Mar 30, 2021

Reporting Summary

Nature Research wishes to improve the reproducibility of the work that we publish. This form provides structure for consistency and transparency in reporting. For further information on Nature Research policies, see Authors & Referees and the Editorial Policy Checklist.

Statistics

For all statistical analyses, confirm that the following items are present in the figure legend, table legend, main text, or Methods section.

n/a	Confirmed	
☐	☒	The exact sample size (*n*) for each experimental group/condition, given as a discrete number and unit of measurement
☐	☒	A statement on whether measurements were taken from distinct samples or whether the same sample was measured repeatedly
☐	☒	The statistical test(s) used AND whether they are one- or two-sided<br>*Only common tests should be described solely by name; describe more complex techniques in the Methods section.*
☒	☐	A description of all covariates tested
☐	☒	A description of any assumptions or corrections, such as tests of normality and adjustment for multiple comparisons
☐	☒	A full description of the statistical parameters including central tendency (e.g. means) or other basic estimates (e.g. regression coefficient) AND variation (e.g. standard deviation) or associated estimates of uncertainty (e.g. confidence intervals)
☐	☒	For null hypothesis testing, the test statistic (e.g. *F*, *t*, *r*) with confidence intervals, effect sizes, degrees of freedom and *P* value noted<br>*Give P values as exact values whenever suitable.*
☒	☐	For Bayesian analysis, information on the choice of priors and Markov chain Monte Carlo settings
☒	☐	For hierarchical and complex designs, identification of the appropriate level for tests and full reporting of outcomes
☒	☐	Estimates of effect sizes (e.g. Cohen's *d*, Pearson's *r*), indicating how they were calculated

Our web collection on statistics for biologists contains articles on many of the points above.

Software and code

Policy information about availability of computer code

Data collection	Geneious Prime 2019, FusionCapt Advance SL4 16.09b, MikroWin 2000 4.34, BD FACSDiva 6.1.3, QuantStudio 12K Flex, Axiovision 4.6
Data analysis	PRISM 8, Geneious Prime 2019, Fiji ImageJ (1.52p), FACSDiva (6.1.3), Image Lab software (v6.1.0 build 7, Bio-Rad), QuantStudio 12K Flex (v1.4), ENCoRE (original published version from 2016), Human Splice Finder (v3.1), NetGene2 (v2.42), TIDE (3.2.0.)

For manuscripts utilizing custom algorithms or software that are central to the research but not yet described in published literature, software must be made available to editors/reviewers. We strongly encourage code deposition in a community repository (e.g. GitHub). See the Nature Research guidelines for submitting code & software for further information.

Data

Policy information about availability of data

All manuscripts must include a data availability statement. This statement should provide the following information, where applicable:

- Accession codes, unique identifiers, or web links for publicly available datasets
- A list of figures that have associated raw data
- A description of any restrictions on data availability

Source data are provided with this paper. All other data supporting the findings of this study are available from the corresponding author on request.

Field-specific reporting

Please select the one below that is the best fit for your research. If you are not sure, read the appropriate sections before making your selection.

☒ Life sciences ☐ Behavioural & social sciences ☐ Ecological, evolutionary & environmental sciences

Life sciences study design

All studies must disclose on these points even when the disclosure is negative.

Sample size	Preliminary luciferase-based experiments showed that there was only small variation over independent replicates, so we decided for n>=3.
Data exclusions	No data points were excluded.
Replication	All experiments were successfully replicated with independent biological samples. Please see the detailed statements in the Statistics@Reproducibility section.
Randomization	This technical report used cell culture as the primary method. Cell culture wells were allocated to the different treatment groups specified by the particular experiment without determining any specific property of the cells in a given well for selecting a specific condition.
Blinding	The experiments were performed, if possible, with master mixes and with multichannel pipettes to exclude unconscious biases during sample preparation. However, the individual human experimenter was not blinded for the conditions as this was not feasible.

Reporting for specific materials, systems and methods

We require information from authors about some types of materials, experimental systems and methods used in many studies. Here, indicate whether each material, system or method listed is relevant to your study. If you are not sure if a list item applies to your research, read the appropriate section before selecting a response.

Materials & experimental systems

n/a	Involved in the study	
☐	☒	Antibodies
☐	☒	Eukaryotic cell lines
☒	☐	Palaeontology
☒	☐	Animals and other organisms
☒	☐	Human research participants
☒	☐	Clinical data

Methods

n/a	Involved in the study	
☒	☐	ChIP-seq
☐	☒	Flow cytometry
☒	☐	MRI-based neuroimaging

Antibodies

Antibodies used

Primary antibodies
M2 mouse anti-FLAG (1:1,000, F1804-200UG, Sigma-Aldrich)
L2 rat anti-OLLAS (1:1,000, MA5-16125, Thermo Fisher Scientific)
PC1C6 mouse anti-pan-tau (1:200, MAB3420, Merck Millipore)
8E6/C11 mouse anti-3R-tau (1:1,000, 05-803, Merck Millipore)
EPR4114 rat anti-FOXP1 (1:1,000, ab134063, abcam)
32F6 mouse anti-mNeonGreen (1:1,000, 32f6-100, ChromoTek)
ab21176 rabbit anti-firefly luciferase (1:1,000, abcam)
D71G9 rabbit anti-TUBB3 (1:1,000, 5568S, Cell Signaling Technology (CST))
AC-15 mouse anti-beta-Actin (HRP-coupled) (1:100,000, ab6276, abcam)

Secondary antibodies (HRP-coupled)
goat anti-mouse IgG H&L (HRP-coupled) (1:20,000, ab97023, abcam)
goat anti-rat IgG H&L (HRP-coupled) (1:20,000, ab97057, abcam)
goat anti-rabbit IgG H&L (HRP-coupled) (1:20,000, ab6721, abcam)

Secondary antibodies (dye-labeled)
Cy3-conjugated cross-adsorbed goat anti-mouse IgG (H+L) (1:1,000, A10521, Thermo Fisher Scientific)
Cy5-conjugated cross-adsorbed goat anti-mouse IgG (H+L) (1:1,000, A10524, Thermo Fisher Scientific)
Alexa Fluor 633-conjugated cross-adsorbed goat anti-rabbit IgG (H+L) (1:1,000, A21070, Thermo Fisher Scientific)
Alexa Fluor 594-conjugated cross-absorbed donkey anti-mouse IgG (H+L) (1:1,000, A21203, Thermo Fisher Scientific)
Alexa Fluor 488-conjugated cross-absorbed goat-anti-rabbit IgG(H+L) (1:1,000, A11008, Thermo Fisher Scientific)
Alexa Fluor 488-conjugated cross-absorbed donkey anti-goat IgG (H+L) (1:1,000, A11055, Thermo Fisher Scientific)

Pluripotency markers
C70B1 rabbit anti-SOX2 (1:500, 3728S, Cell Signaling Technology)
C30A3 rabbit anti-OCT4A (1:500, 2840, Cell Signaling Technology)
Goat anti-NANOG (1:500, AF1997, R&D Systems)

Germ layer-specific markers
Endoderm:
Goat anti-SOX17 (1:1,000, AF1924, R&D Systems)
P87H4B7 mouse anti-FOXA2 (1:1,000, 685802, BioLegend)

Ectoderm:
AD2.38 mouse anti-PAX6 (1:200, ab78545, abcam)
10C2 mouse anti-Nestin (1:250, MA1-110, Thermo Fisher Scientific)

Mesoderm:
EPR18113 rabbit anti-TBXT (1:1,000, ab209665, abcam)
Rabbit anti-NCAM1 (1:200, ab204446, abcam)

smNPC identity verification
AD2.38 mouse anti-PAX6 (1:500, ab78545, abcam)
10C2 mouse anti-Nestin (1:500, MA1-110, Thermo Fisher Scientific)
C70B1 rabbit anti-SOX2 (1:500, 3728S, Cell Signaling Technology)
SDL.3D10 mouse anti-TUBB3 (1:500, T5076-200UL, Sigma-Aldrich)

Neuronal markers:
SDL.3D10 mouse anti-TUBB3 (1:500, T5076-200UL, Sigma-Aldrich)
Rabbit anti-MAP2 (1:250, AB5622, Merck Millipore)

negative markers:
C30A3 rabbit anti-OCT4A (1:500, 2840, Cell Signaling Technology)
EPR4007 rabbit anti-SOX10 (1:250, ab155279, abcam)

Validation

All antibodies were purchased from respected manufacturers that provide detailed validation. If applicable, specificity was validated using controls without the corresponding antigens (e.g., via control transfections).

M2 mouse anti-FLAG (F1804-200UG, Sigma-Aldrich) https://www.sigmaaldrich.com/life-science/proteomics/recombinant-protein-expression/purification-detection/flag-antibodies.html
L2 rat anti-OLLAS (MA5-16125, Thermo Fisher Scientific) https://www.thermofisher.com/order/genome-database/dataSheetPdf?producttype=antibody&productsubtype=antibody_primary&productId=MA5-16125&version=137
PC1C6 mouse anti-pan-tau (MAB3420, Merck Millipore) https://www.sigmaaldrich.com/catalog/product/mm/mab3420?lang=de&region=DE
8E6/C11 mouse anti-3R-tau (05-803, Merck Millipore) https://www.sigmaaldrich.com/catalog/product/mm/05803?lang=de&region=DE
EPR4114 rat anti-FOXP1 (ab134063, abcam) https://www.abcam.com/ab134063.pdf?
32F6 mouse anti-mNeonGreen (32f6-100, ChromoTek) https://www.chromotek.com/fileadmin/content/PDFs/Protocols/mNeonGreen_32F6.pdf
rabbit anti-firefly luciferase (ab21176, abcam) https://www.abcam.com/firefly-luciferase-antibody-ab21176.html
D71G9 rabbit anti-TUBB3 (5568S, Cell Signaling Technology (CST)) https://www.cellsignal.com/products/primary-antibodies/b3-tubulin-d71g9-xp-rabbit-mab/5568
AC-15 mouse anti-beta-Actin (HRP-coupled) (ab6276, abcam) https://www.abcam.com/beta-actin-antibody-ac-15-ab6276.html
goat anti-mouse IgG H&L (HRP-coupled) (ab97023, abcam) https://www.abcam.com/goat-mouse-igg-hl-hrp-ab97023.html
goat anti-rat IgG H&L (HRP-coupled) (ab97057, abcam) https://www.abcam.com/goat-rat-igg-hl-hrp-ab97057.html
goat anti-rabbit IgG H&L (HRP-coupled) (ab6721, abcam) https://www.abcam.com/goat-rabbit-igg-hl-hrp-ab6721.html
Cy3-conjugated cross-adsorbed goat anti-mouse IgG (H+L) (A10521, Thermo Fisher Scientific) https://www.thermofisher.com/order/genome-database/dataSheetPdf?producttype=antibody&productsubtype=antibody_secondary&productId=A10521&version=137
Cy5-conjugated cross-adsorbed goat anti-mouse IgG (H+L) (A10524, Thermo Fisher Scientific) https://www.thermofisher.com/order/genome-database/dataSheetPdf?producttype=antibody&productsubtype=antibody_secondary&productId=A10524&version=137
Alexa Fluor 633-conjugated cross-adsorbed goat anti-rabbit IgG (H+L) (A21070, Thermo Fisher Scientific) https://www.thermofisher.com/order/genome-database/dataSheetPdf?producttype=antibody&productsubtype=antibody_secondary&productId=A-21070&version=137
Alexa Fluor 594-conjugated cross-absorbed donkey anti-mouse IgG (H+L) (A21203, Thermo Fisher Scientific) https://www.thermofisher.com/order/genome-database/dataSheetPdf?producttype=antibody&productsubtype=antibody_secondary&productId=A-21203&version=137
Alexa Fluor 488-conjugated cross-absorbed goat-anti-rabbit IgG(H+L) (A11008, Thermo Fisher Scientific) https://www.thermofisher.com/order/genome-database/dataSheetPdf?producttype=antibody&productsubtype=antibody_secondary&productId=A-11008&version=137
Alexa Fluor 488-conjugated cross-absorbed donkey anti-goat IgG (H+L) (A11055, Thermo Fisher Scientific) https://www.thermofisher.com/order/genome-database/dataSheetPdf?producttype=antibody&productsubtype=antibody_secondary&productId=A-11055&version=137
C70B1 rabbit anti-SOX2 (3728S, Cell Signaling Technology) https://www.cellsignal.com/products/primary-antibodies/sox2-c70b1-rabbit-mab-ihc-preferred/3728
C30A3 rabbit anti-OCT4A (2840, Cell Signaling Technology) https://www.cellsignal.com/products/primary-antibodies/oct-4a-c30a3-rabbit-mab/2840
Goat anti-NANOG (AF1997, R&D Systems) https://www.rndsystems.com/products/human-nanog-antibody_af1997#product-

details
Goat anti-SOX17 (AF1924, R&D Systems) https://www.rndsystems.com/products/human-sox17-antibody_af1924
P87H4B7 mouse anti-FOXA2 (685802, BioLegend) https://www.biolegend.com/de-de/products/purified-anti-foxa2-antibody-13176
AD2.38 mouse anti-PAX6 (ab78545, abcam) https://www.abcam.com/pax6-antibody-ad238-ab78545.html
EPR18113 rabbit anti-TBXT (ab209665, abcam) https://www.abcam.com/brachyury--bry-antibody-epr18113-ab209665.html
Rabbit anti-NCAM1 (ab204446, abcam) https://www.abcam.com/ncam1-antibody-ab204446.html
10C2 mouse anti-Nestin (MA1-110, Thermo Fisher Scientific) https://www.thermofisher.com/order/genome-database/dataSheetPdf?producttype=antibody&productsubtype=antibody_primary&productId=MA1-110&version=137
SDL.3D10 mouse anti-TUBB3 (T5076-200UL, Sigma-Aldrich) https://www.sigmaaldrich.com/catalog/product/sigma/t5076?lang=de&region=DE
Rabbit anti-MAP2 (AB5622, Merck Millipore) https://www.sigmaaldrich.com/catalog/product/mm/ab5622?lang=de&region=DE
EPR4007 rabbit anti-SOX10 (ab155279, abcam) https://www.abcam.com/sox10-antibody-epr4007-ab155279.html

Eukaryotic cell lines

Policy information about cell lines

Cell line source(s)
HEK293T (human), Neuro2a/N2a (mouse) from ATCC, hiPSCs HPSI0514i-vuna_3 (77650602, ECACC) and hiPSCs HPSI0614i-uilk_2 (ECACC: 77650606) from the European Collection of Authenticated Cell Cultures.

Authentication
HEK293T and Neuro-2a were bought from ATCC and were not additionally authenticated. hiPSCs and smNPCs were authenticated using typical pluripotency or NPC markers (see methods section: Verification of cellular identities (iPSCs and smNPCs) using immunofluorescence detection). Chromosomal aberration was excluded by G-Banding (see methods section: Verification of cellular identities (iPSCs and smNPCs) using immunofluorescence detection).

Mycoplasma contamination
All cell lines were tested for mycoplasma contamination using MycoAlertTM Mycoplasma Detection Kit (LT07-318, Lonza). In addition, all cell lines were tested every 3 months for contamination by Hoechst 3334, which visualizes extranuclear speckles in case of contamination. All results shown in the manuscript were from cells tested negative for mycoplasma.

Commonly misidentified lines
(See ICLAC register)
No commonly misidentified cell lines were used in this study.

Flow Cytometry

Plots

Confirm that:

☒ The axis labels state the marker and fluorochrome used (e.g. CD4-FITC).

☒ The axis scales are clearly visible. Include numbers along axes only for bottom left plot of group (a 'group' is an analysis of identical markers).

☒ All plots are contour plots with outliers or pseudocolor plots.

☒ A numerical value for number of cells or percentage (with statistics) is provided.

Methodology

Sample preparation
Adherent cells were dissociated with Accutase and centrifuged at 200 rcf for 5 min at RT, and cell pellets were resuspended in ice-cold PBS with 2% FBS. The resuspended cells were transferred into conical 5 ml polystyrene round-bottom tubes, including a cell-strainer cap, and were kept on ice.

Instrument
BD FACSaria II (BD Biosciences)

Software
BD FACSDiva Software (Version 6.1.3, BD Biosciences)

Cell population abundance
For FACS analysis, at least 50,000 events were recorded per condition. The smallest fraction of analyzed cells was >5% of total events.

Gating strategy
The main population of cells was gated according to their forward scatter and sideward scatter. Afterward, single cells were chosen according to their FSC-A and FSC-W. Subsequently, the transfected cells were gated according to their blue fluorescence (450 nm). These fluorescence gates were determined using blue- and green-negative cells by control-transfection with iRFP720 (infrared fluorescence). The 530 nm quartile gates were set in the DMSO control condition such that 25% of cells were passing the gate. These gates were not changed for all subsequent experimental conditions.

☒ Tick this box to confirm that a figure exemplifying the gating strategy is provided in the Supplementary Information.
