# Supplementary figures and images for: Non-invasive and high-throughput interrogation of exon-specific isoform expression
Source: Nat Cell Biol. 2021 Jun 3;23(6):652–63. doi: 10.1038/s41556-021-00678-x (PMC8189919; doi:10.1038/s41556-021-00678-x)

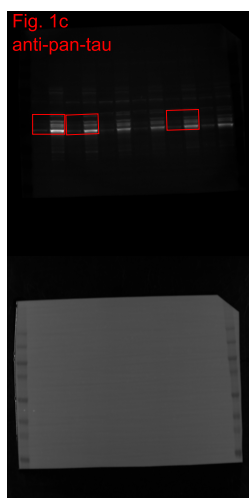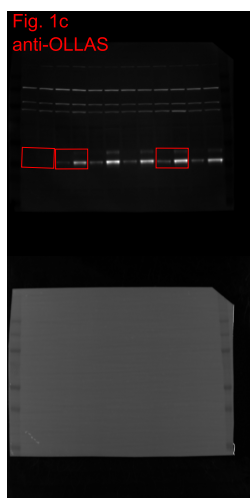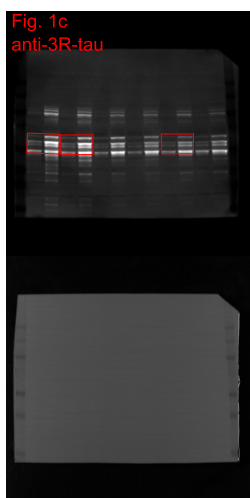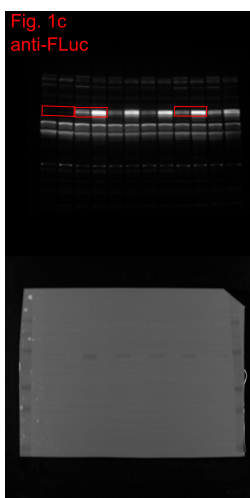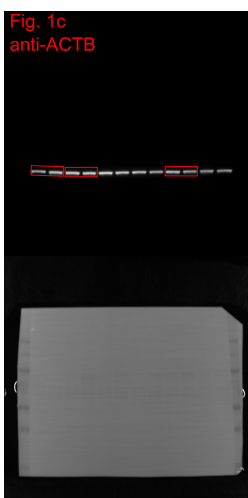

Supplement: Source Data Fig. 1 — Unprocessed immunoblot. [file 41556_2021_678_MOESM6_ESM.pdf]

Fig. 6e  
anti-FOXP1

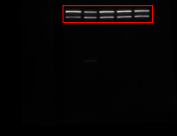

Fig. 6e  
anti-OLLAS

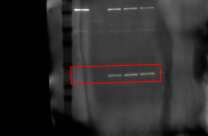

Fig. 6e  
anti-ACTB  
(from anti-OLLAS)

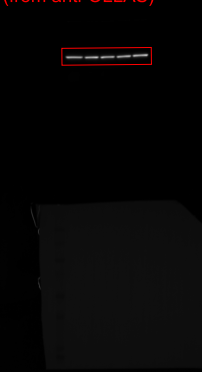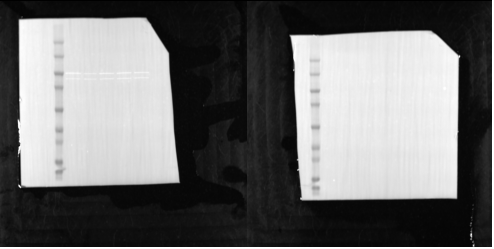

Fig. 6f

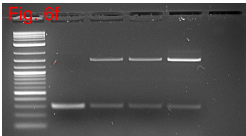

Supplement: Source Data Fig. 6 — Unprocessed immunoblot. [file 41556_2021_678_MOESM13_ESM.pdf]

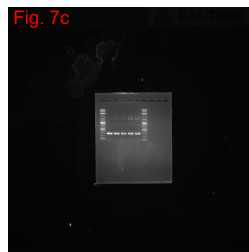

Supplement: Source Data Fig. 7 — Unprocessed immunoblot. [file 41556_2021_678_MOESM15_ESM.pdf]

Extended Data Fig. 1  
anti-FLAG

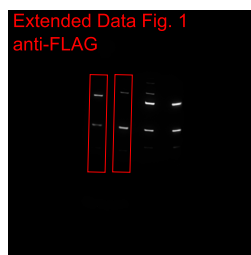

Extended Data Fig. 1  
overexposure  
anti-FLAG

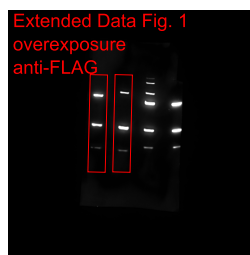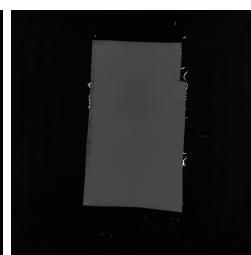

Supplement: Source Data Extended Data Fig. 1 — Unprocessed immunoblot. [file 41556_2021_678_MOESM16_ESM.pdf]

Extended Data Fig. 2b  
anti-pan-tau

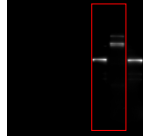

Extended Data Fig. 2b  
anti-ACTB

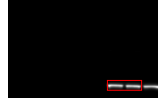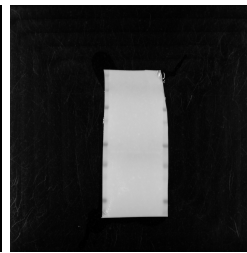

Supplement: Source Data Extended Data Fig. 2 — Unprocessed immunoblot. [file 41556_2021_678_MOESM17_ESM.pdf]

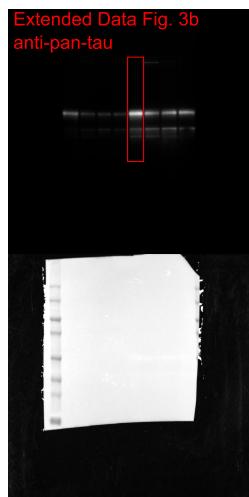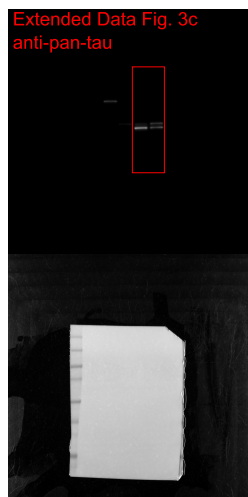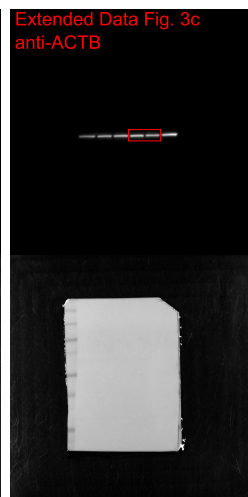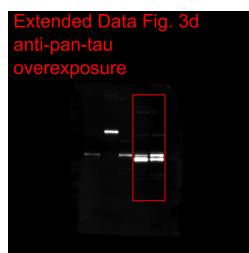

Supplement: Source Data Extended Data Fig. 3 — Unprocessed immunoblot. [file 41556_2021_678_MOESM18_ESM.pdf]

Extended Data Fig. 4  
anti-pan-tau

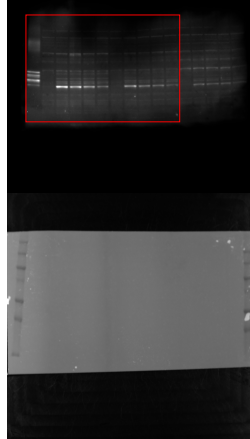

Extended Data Fig. 4  
anti-OLLAS

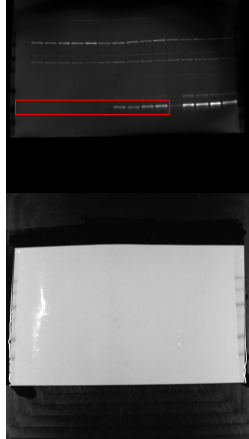

Extended Data Fig. 4  
anti-ACTB

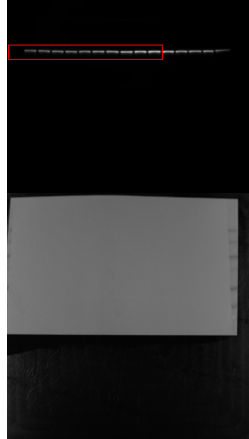

Supplement: Source Data Extended Data Fig. 4 — Unprocessed immunoblot. [file 41556_2021_678_MOESM20_ESM.pdf]

Extended Data Fig. 8d  
anti-pan-tau

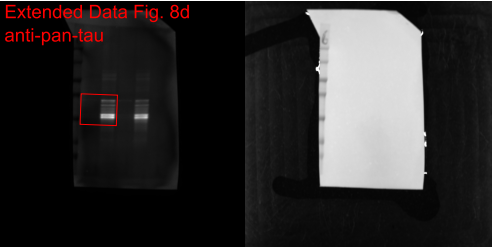

Extended Data Fig. 8m  
anti-OLLAS

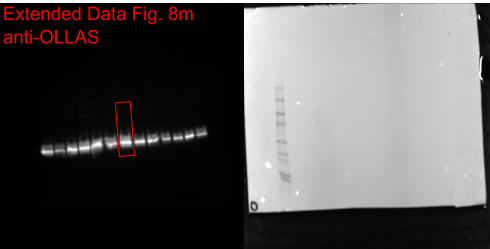

Extended Data Fig. 8m  
anti-Tubb3

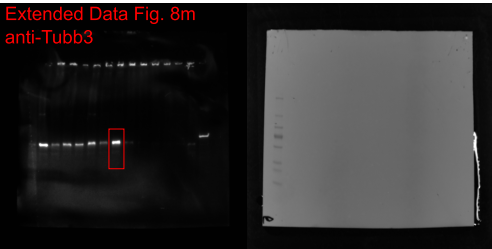

Supplement: Source Data Extended Data Fig. 8 — Unprocessed immunoblot. [file 41556_2021_678_MOESM22_ESM.pdf]

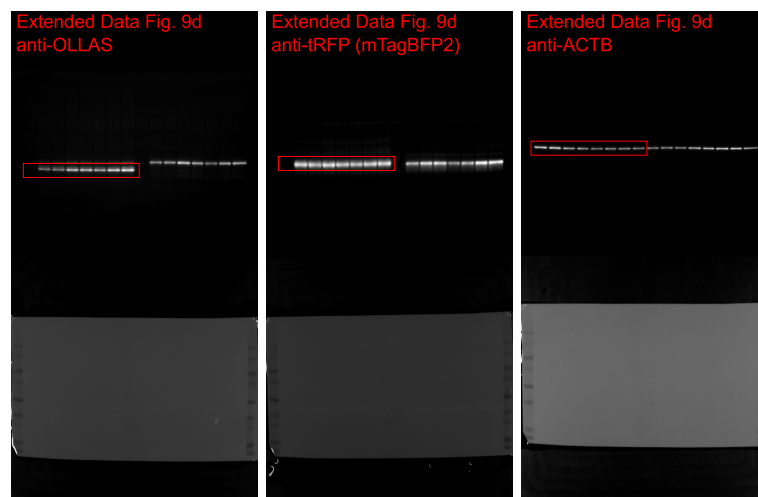

Supplement: Source Data Extended Data Fig. 9 — Unprocessed immunoblot. [file 41556_2021_678_MOESM25_ESM.pdf]
