# Supplementary material for: Non-invasive and high-throughput interrogation of exon-specific isoform expression
Source: Nat Cell Biol. 2021 Jun 3;23(6):652–63. doi: 10.1038/s41556-021-00678-x (PMC8189919; doi:10.1038/s41556-021-00678-x)
Supplement: Supplementary file 3 — Peer Review Information [file 41556_2021_678_MOESM3_ESM.pdf]

nature research

Peer Review Information

Journal: Westmeyer
Manuscript Title: Non-invasive and high-throughput interrogation of exon-specific isoform expression
Corresponding author name(s): Gil Westmeyer

Reviewer Comments & Decisions:

Decision Letter, initial version:

Date: 12th June 19 07:47:25
Last Sent: 12th June 19 07:47:25
Triggered By: Jie Wang
From: jie.wang@nature.com
To: gil.westmeyer@tum.de
Subject: Decision on presubmission enquiry NCB-W40046
Message: Dear Prof Westmeyer,

Thank you for your interest in submitting your work to Nature Cell Biology. The study sounds interesting and appropriate for our journal, and we would like to send the manuscript for formal review as a Technical Report.

Please use this link to submit the complete manuscript:

[REDACTED]

We would like to receive the full submission as soon as possible, ideally within two weeks. If submission is substantially delayed, we would still be happy to consider the manuscript, however, we will consider the published literature at the time of submission to assess its impact on the level of advance provided by the study.

Please feel free to contact me if you have any questions. We look forward to receiving the submission.

Kind regards,

Jie Wang

Jie Wang, PhD

Senior Editor
Nature Cell Biology

Tel: +44 (0) 207 843 4924
email: jie.wang@nature.com

Decision Letter, first revision:

Date: 20th February 20 21:55:40
Last Sent: 20th February 20 21:55:40
Triggered By: Jie Wang
From: jie.wang@nature.com
To: gil.westmeyer@tum.de
CC: NCB@springernature.com
Subject: Decision on Nature Cell Biology submission NCB-W40046A
Message: Dear Prof Westmeyer,

Your manuscript "Non-invasive and high-throughput interrogation of exon-specific isoform expression", has now been seen by 4 referees, who are experts in RNA splicing (referees 1 and 2); RNA biology and CRISPR (referee 3); and inteins (referee 4), and whose comments are pasted below. In light of their advice, we regret that we cannot offer to publish the study in Nature Cell Biology.

As you will see, although the reviewers find this work interesting, they raise extensive concerns that question the technical advance that these findings represent over previous work, and the strength of the data and of the novel conclusions that can be drawn at this stage.

In particular, among the limitations of the dataset the referees note the insufficiently demonstrated technical advance over existing methods to monitor RNA splicing (general comments by referee 2 and major point 1 by referee 3), potential undesired effects of the system (points 2 and 3 by referee 1), and technical issues with intein-mediated protein trans-splicing (point C by referee 4).

We would be open to the possibility of considering a revised manuscript that would fully address the referee concerns. However, any decision to re-review such a revised study would depend on the strength of the revisions and the published literature at the time of resubmission.

We are very sorry that we could not be more positive on this occasion, but we thank you for the opportunity to consider this work.

With kind regards,

Jie Wang

Jie Wang, PhD
Senior Editor
Nature Cell Biology

Tel: +44 (0) 207 843 4924
email: jie.wang@nature.com

Reviewers' comments:

Reviewer #1 (Remarks to the Author):

In this manuscript, Truong et al. describe a new tool, EXSISERS, to assess changes in splicing isoforms at the protein level and/or tag cells with specific protein splicing isoforms. The authors are taking advantage of the capacity of inteins to splice themselves out at the protein level, without affecting the RNA or coding sequence where there are integrated in. Using these inteins, they have shown with a wide set of examples, how they can insert at the endogenous level, in the alternatively spliced exon of choice, a reporter that can be spliced out at the protein level by specific inteins. With this system, by looking at expression of the protein reporter, which can be a luciferase protein, a blasticidine resistant gene, a fluorescent protein, an halo tag that goes to the membrane for cell sorting, one can identify, quantify, cell sort, live image cells expressing a specific splicing isoform of interest without the need of artificial reporters, splicing-specific antibodies, or the need to rely on RNA-based methodologies that most of the times are not impacting proteins at the same level. With this new system, one can assess the real splicing isoforms that exist at the protein level, follow them, manipulate them and even use them as a read out for CRISPR screening, imaging and sorting. It is extremely versatile and useful for studying many mechanisms and more importantly the biological relevance of a particular splicing variant at the protein level, and not the RNA level as we usually do (which underestimates all the post-transcriptional effects that could come from the new splice variant). Moreover, in the manuscript, the use of RfxCas13d and PspCas13b to specifically knock down one specific splicing isoform is also studied, bringing light to this also new and poorly understood tool. Key aspects of the crRNA design and if it is better to target the nascent pre-mRNA or the mature mRNA are shown.
Overall the manuscript is clear, robust and full of insightful new tools and recommendations to work with specific splicing isoforms at all possible levels. It is therefore of great interest for the scientific community and deserves publication if some concerns are addressed first.
Comments:
1) Since this is a manuscript selling a new tool, it would be nice if the authors comment whether it is difficult to endogenously tag at the homozygous level such reporter sequences. Have they tried many different type of cells? Which is the size of the biggest reporter they successfully inserted? I say this, because it is known that not all cells are easy to CRISPR tag and it is even more difficult to tag the two alleles, and even more two regions of the same gene at the two alleles. What happens in cells with more than two alleles? Is it really important to tag all alleles? All this could be commented to reinforce feasibility.

2) It is also important to prove that there is no effect on the endogenous transcript nor protein. That splicing occurs normally and the protein levels are not affected by insertion of these reporters and inteins. I don't think the authors have done this properly in the manuscript. Actually in Fig.2d, there is more 4R isoform in HEK than in the WT-EXISERS clone. Shouldn't these two cells be comparable? It is important to show that splicing patterns are not affected by insertion of these constructs, that protein levels are not affected, that function is not affected and that splicing could even change if necessary, such as in their iPS differentiation system. Also, can inteins have off target effects? This is not mentioned nor proved.

3) A kind of related question: can you insert the NLuc/FLuc reporter anywhere in the exon regardless of the regulatory splicing sequences? And how come increasing considerably the exon size has no effect on exon recognition and recruitment of the splicing machinery? As a splicing expert, it surprises me…

4) In Fig.2e, why there are equal levels of NLuc and FLuc in WT induced cells? If the exon is not included, NLuc should be lower than FLuc, right? Then with the use of 5-iodotubercidin, which induces e10 inclusion, in suppl Fig.6 there is increase of both 4R (+ex10) and 3R(-ex10) isoforms. How come? 3R should not increase…

5) Are the two splicing intein proteins equally efficient splicing out the Luc proteins (Gp41-1 and NrdJ-1)? Maybe Suppl Fig 5 was intended to study this, but I don't understand the results. Looks like for each NLuc signal there are 30 of FLuc, which makes FLuc more efficiently spliced. Was this corrected in the main figures? It is kind of important since usually we look at the relative levels of the alternatively spliced isoform vs total protein. If one intein is more efficient than the other, it will affect interpretation of results. Also, can inteins splice out all the mRNAs translated? In a screening, can inteins be inhibited leading to indirect effects (no blasticidin not because there is no exon inclusion, but intein is inhibited or translation inhibited)?

6) Taking into consideration that the RNA is affected by using Cas13. It is important to show that the « protein » splicing effects observed with the inteins are also true at the RNA level by qRT-PCRs. e10 and total MAPT RNA levels should be affected accordingly in Fig.3. It is an important control.

7) In Fig.3c, why crRNA 10-11 is not affecting total MAPT levels but 9-10 is ? More puzzling, why the use of shRNAs to mimic miRNAs pathway has the opposite effect, it is the 9-10 that is more isoform specific than 10-11 ?

8) Fig3f, dCasRX-SR effect is just 1,6x-fold. I don't think this is going to be biologically meaningful. The control in which there is dCasRx-SR or dCasRX-hnRNPA1 but not crRNA is missing (to make sure there are no indirect effects).

9) Again, the effect on Suppl Fig 12 seems very low too, 1,5x-fold. Is this sufficient to claim what the authors claim?

10) Why are the IFs in Fig2c and Supplementary Figure 11d,f so dotted at the nuclear level? Is this related to the reporter?

11) For Fig.4, the CRSPR screening, it is important to know how many clones resisted to the blasticidin to know the false-positive rate of the system. The authors only show the positive MBNL1 clone, but this was already well known. Was the finding straightforward? It does not invalidate the proof-of-concept but it can give perspective on the feasibility of the system. It is known that some cells can escape the blasticidin selection. Were the authors using a higher amount of antibiotic that what is used for clone selection (1-10 ug/mL depending on the cell type)?

Reviewer #2 (Remarks to the Author):

In this manuscript, the authors developed a new type of cell-based reporter system, exon-specific isoform expression reporter system (EXSISERS), which enables non-invasive detection of alternative spicing and exon-specific translation via intein-mediated protein splicing. They construct generated dual-luciferase (NLuc and Fluc) EXSISERS lines for ratiometric monitoring of different Tau protein isoforms, 3R-tau and 4R-tau. As designed, the system can recapitulate the expected change of different tau protein isoforms. The application of this reporter system was further demonstrated in several scenarios: 1. Screening of the effective guide RNAs in CRISPR/Cas-13 system that can achieve isoform-specific gene silencing; 2. Testing the activity of designer splicing enhancer or suppressor using the dCas-13 fusion protein containing SR domain or Gly-rich domain; 3. Measuring the co-translation ribosomal frameshift regulation. Finally, they generated an EXSISERS reporter for alternative splicing of exon 18b in FOXP1 and use the reporter to identify the regulators for isoform-specific expression of this exon via genome-wide CRISPR/Cas9 screen. Given their results the authors propose that it will be possible for an unbiased and non-invasive functional screening for splice modulators.
Overall I find the approaches employed in this study is valuable for characterizing and manipulating the intrinsic functionality of the exon-specific protein isoforms. However, the system is cumbersome to use and require a large amount of time for consecutive steps of CRISPR-cas insertion, which will limit its usefulness. In addition, some of the application did not perform as efficiently as previous system that was much simpler to generate. For example, the designer splicing enhancer and silencer using aCas-13 in EXSISERS reporter (Fig. 3f and 3g) was not as efficient as the engineered splicing factors using PUF fusion proteins (Wang Y et al, 2009 Nature Method, Wang Y et al 2013 NSMB), which is much simpler system to use. The authors should acknowledge such limitation and compare their system with previous system.

Specific concerns:
1. The intein used in this study were shown to have high splicing efficiency (Supplementary Fig. 1) in their system, however I am curious about how efficiently the intein works in different cell lines. Additional quantification should be performed to measure the intein excision rather than assuming it is always 100% excised.
2. In Fig.2, since the study is focusing on the exon-specific isoforms of tau protein, the authors should use an exon10 specific tau antibody (or pan antibody for tau) to calibrate the system. This is to make sure that the results obtained from luciferase measurement correlate well with direct measurement of tau isoforms.

3. In Fig.4, I feel that this part lacks an important analysis on transcriptome level for the MBNL1/2-KO cells and the exon 18b inclusion cells after blasticidin selection. MBNL1/2 are key regulator in RNA splicing, and knock-out of these two genes should cause significant change of splicing in the level of entire transcriptome. I am wondering whether knock-out of these two genes could cause more exon-specific protein changes besides FOXP1.
4. I think this paper may present a powerful tool to track and study exon-specific protein isoform. However, the authors should use it to investigate on new biological questions rather than only to confirm the conclusion people have already made.

Minor concern:
Overall the figures are poorly prepared with low resolution and confusing color scheme, more specifically:
1. The picture quality of Fig.2c and Fig.2g should be improved. The color and style of this figure should be modified to make it more reader friendly. In addition, Fig.2c and 2g should be showed in color to help understand.
2. The picture quality of Fig.4c and Fig.4d should be improved. And the part (Identification of regulators for isoform-specific expression) and Fig.4 need be carefully reviewed, because the figure and the main text are not consistent.
3. Supplementary Fig.8b need to be updated, as the resolution is very low.
4. Similar to Fig. 2c, the supplementary Fig.11 and Fig.13 should be improved.

Reviewer #3 (Remarks to the Author):

Truong et al. develop a minimally invasive isoform-specific expression reporter system (EXSISERS) that incorporates translated and subsequently excised fast-splicing inteins with CC-domains into genes of interest. The authors demonstrate the utility of EXSISERS in a number of applications, ranging from the optimization of RNA-targeting strategies for exon-specific RNA degradation of MAPT mRNA, to the quantification of ribosomal frameshift-mediated regulations unmeasurable by RT-qPCR, to a phenotypic readout for a high-throughput screen of FOXP1 exon 18b inclusion that validates existing literature. Altogether, the presented work is a valuable addition to the isoform-specific RNA monitoring toolkit. While the generation of EXSISERS may be an involved process, nevertheless for some applications it might prove more useful than alternative methodologies, such as minigenes. I have a few major criticisms.
Major points:
1. The authors do not perform any head-to-head comparisons of EXSISERS to minigenes, which are comparatively much simpler and faster to generate. This should be done. If there is no clear advantage of EXSISERS, then it is worth wondering whether other researchers will adopt the new methodology.
2. The authors use CRISPR-Cas9 to integrate EXSISERS into areas of interest in the genome. When such knock-ins are performed and analyzed, typically researchers will generate multiple clonal cell lines, in case behavior in one cell line may be biased by unique Cas9-induced indel and/or template insertion off-target events. The authors should re-perform the experiments featured in Figures 3 and 4 (and associated supplemental figures) with at least one additional clonal cell line to demonstrate the generalizability of EXSISERS.

Minor points:
1. The introduction would benefit from a reference to work on minigenes, as they are the main methodological competitor to EXSISERS.
2. The sentence should read “greater reduction”: Expression of cytosolic PspCas13b-NES directed against the same region of exon 10 (Fig. 3e, orange bar) resulted in a greater reduction of FLuc as compared with the corresponding RfxCas13d-NLS ($p<0.0001$, post-hoc tests of one-way ANOVA) with comparable NLuc signal ($p>0.05$) (Fig. 3e, blue bar).
3. The sentence should read “4f”: Meanwhile, the enrichment of MBNL2 indels showed no dose-dependence (Fig. 4f).

Reviewer #4 (Remarks to the Author):

A. This work elegantly solves the current issues in quantifying protein expression levels by RNA-based approaches by incorporating a newly developed reporter system termed an exon-specific isoform expression reporter system (EXSISERS). The authors incorporated two EXSISERS into exons of interest (EOIs) by CRISPR/Cas9 and monitored the alternative splicing involved disease-associated exon inclusion of the patient-driven iPSC cells and screened RNA interference sequence for the isoform-specific expression to identify splice-regulators. Additionally, the authors similarly developed a survival reporter system for isoform-specific Blasticidin-S resistance marker. This article proposes the new exon-specific isoform expression reporter system would be a new tool for monitoring spatiotemporal exon-specific expression by imaging techniques.

B.
This work is highly original and innovative with potential impacts in identifying splicing regulators and drug screening. Notably, this method could address the problems associated with protein expression level determined by RNA-based quantification methods. Thus, it is of significant importance and could be a game-changer for current RNA-based approaches if it is robust and reliable.

C.
In this system, there are several critical assumptions have not been controlled in this manuscript, which should be addressed in the manuscript before publications.

1. The manuscript is described as if protein trans-splicing has 100% efficiency (like Fig 2a, 2b). The splicing efficiency by protein trans-splicing is strongly affected by the junction sequence and the foreign exteins used. A single mutation near the junctions could abolish or deceased the splicing activity significantly, missing the controls to check the protein splicing efficiency.
2. Another assumption is similar to the previous one, FLuc and NLuc inserted in inteins fold into active equally with the same efficiency, yet having the same degradation rate in cells. The authors need to provide such experimental controls.
3. NLuc has 13-236 fold brighter than Fluc, according to the literature. All the data reported by normalized with the assumption, I believe.
4. The main caveat of this system easily overlooked by non-experts is the assumption that protein splicing by two split inteins has 100% or close to 100% efficiency. Particularly such high splicing activity for two orthogonal inteins has not

been achieved in the past with an artificial system to my best knowledge. The reported efficiency of 95% in the cited ref.17 would result in the 90% efficiency for two orthogonal inteins. This assumption could determine the outcome of the analysis based on NLuc/FLuc quantification drastically.

D.
NLuc usually has 13-236 >times brighter than Fluc according to the literature, which is consistent with the data presented with Figure 2e. The NLuc/Fluc error bars cannot be smaller than each of them. However, Figure 2j and all other data presented in Figure 3 do not make any sense, statistically.
The error estimation (P-value analysis) needs to be reconsidered. There are two types of errors mixed: (1) Errors from the detection (readout values) and (2) errors from individual samples or measurements. Even when the calculated error estimated from 3 samples is small, the accuracy of the measurement cannot be better than the precision of the detection errors.

E.
As suggested in section C, D, and F, the validity of this system needs to be validated by additional controls. The authors should describe what would be potential pitfalls by the use of this reporter system. The current presentation does not provide sufficiently clear data to judge the validity and reliability of the system.

F.
• There is no estimation of protein splicing efficiency for none of their protein splicing constructs except for mNG shown in Supplemental Fig. 1 by immunoblot. This data also does not give any estimate of the fully spliced vs by-products (non-spliced, N- and C-cleaved products). The supplemental Fig. 1 should be supplemented by immunoblotting and/or CBB-stained SDS-gels using, for example, anti-Ollas and Flag antibodies. The quantification by NLuc/Fluc ration will be strongly affected by the ligation efficiency, which is strongly dependent on the foreign extein as well as the splicing junctions.
• What is the correlation between the quantification by immunoblotting (and/or mRNA quantification) vs NLuc/FLuc ratio for different constructs? Does it correlate well? if not, do they have a similar trend, which could be explained to some extent?
• See also section D on the statistical data analysis.
• Fig.2d needs controls for protein-splicing deficient constructs by Ser-to-Ala and/or Asn-to-Ala.
• The authors claim “bio-orthogonal pair” of two inteins, but there is no such experimental evidence provided, including cited ref. 17. Trans-splicing is strongly dependent on the exteins, the authors could provide such data as a control, as this will affect the interpretation of the ratiometric data significantly. The orthogonality of two split intein should be demonstrated by using their systems because protein splicing by inteins is strongly extein-dependent.
• The author provided only one experimental data in Supplemental Fig 1 of immunoblotting and did not disclose any further sequence in detail. At least Supplemental Fig. 1 could be supplemented by covering all possible products using anti-Olla and Flag antibodies and provide the protein splicing efficiency quantitated for each of the two splicing steps. In theory, cleaved products might not interfere with NLuc/Fluc ratio. Do the authors have any evidence to assume that is the case?
• The main claims generally focus on the Ratio-metric assay using NLuc/Fluc, the survival system using BSD could be more confusing for readers than making it

clear to understand the reporter system as currently written.

H.

• The abstract is concise and clear.
• There are several misleading statements in the introduction, the authors claim "fast" protein splicing but no speed or relevant time scale is given. Protein splicing is strongly context-dependent, has to be investigated for each extein. This claim is thus not validated in the manuscript. Moreover, there is no information about "trace-less" because the authors do not disclose the protein sequence for junction regions. "Traceless" should mean the spliced sequence is identical to the original protein sequence without a single mutation. Is this the case?
• The current data is not sufficiently supporting the conclusion because of several assumptions and lacks critical controls to verify each of the critical assumptions.

**Although we cannot offer to publish your paper in Nature Cell Biology, the work may be appropriate for another journal in the Nature Research portfolio. If you wish to explore suitable journals and transfer your manuscript to a journal of your choice, please use our manuscript transfer portal. If you transfer to Nature-branded journals or to the Communications journals, you will not have to re-supply manuscript metadata and files. This link can only be used once and remains active until used.
All Nature Research journals are editorially independent, and the decision to consider your manuscript will be taken by their own editorial staff. For more information, please see our manuscript transfer FAQ page.

**For Nature Research general information and news for authors, see http://npg.nature.com/authors.

Author Rebuttal, second revision:

Reviewer #1 (Remarks to the Author):

In this manuscript, Truong et al. describe a new tool, EXSISERS, to assess changes in splicing isoforms at the protein level and/or tag cells with specific protein splicing isoforms. The authors are taking advantage of the capacity of inteins to splice themselves out at the protein level, without affecting the RNA or coding sequence where there are integrated in. Using these inteins, they have shown with a wide set of examples, how they can insert at the endogenous level, in the alternatively spliced exon of choice, a reporter that can be spliced out at the protein level by specific inteins. With this system, by looking at expression of the protein reporter, which can be a luciferase protein, a blasticidine resistant gene, a fluorescent protein, an halo tag that goes to the membrane for cell sorting, one can identify, quantify, cell sort, live image cells expressing a specific splicing isoform of interest without the need of artificial reporters, splicing-specific antibodies, or the need to rely on RNA-based methodologies that most of the times are not impacting proteins at the same level. With this new system, one can assess the real splicing isoforms that exist at the protein level, follow them, manipulate them and even use them as a read out for CRISPR screening, imaging and sorting. It is extremely versatile and useful for studying many mechanisms and more importantly the biological relevance of a particular splicing variant at the protein level, and not the RNA level as we usually do (which underestimates all the post-transcriptional effects that could come from the new splice variant). Moreover, in the manuscript, the use of RfxCas13d and PspCas13b to specifically knock down one specific splicing isoform is also studied, bringing light to this also new and poorly understood tool. Key aspects of the crRNA design and if it is better to target the nascent pre-mRNA or the mature mRNA are shown.
Overall the manuscript is clear, robust and full of insightful new tools and recommendations to work with specific splicing isoforms at all possible levels. It is therefore of great interest for the scientific community and deserves publication if some concerns are addressed first.

Comments:

R1P1:

***1)** Since this is a manuscript selling a new tool, it would be nice if the authors comment whether it is difficult to endogenously tag at the homozygous level such reporter sequences. Have they tried many different type of cells? Which is the size of the biggest reporter they successfully inserted? I say this, because it is known that not all cells are easy to CRISPR tag and it is even more difficult to tag the two alleles, and even more two regions of the same gene at the two alleles. What happens in cells with more than two alleles? Is it really important to tag all alleles? All this could be commented to reinforce feasibility.*

Response to R1P1:

We thank the Reviewer for acknowledging the value of EXSISERS for studying isoform-specific expression.
With respect to cell types, we tested HEK293T, Neuro-2a, and several human induced pluripotent stem cell lines. Homozygous knock-ins were also achieved by a collaboration partner using an unrelated gene in HepG2 and HCT116 cells.
Although the efficiency of CRISPR/Cas9 type of gene editing tools will surely further improve and make systems such as EXSISERS even more convenient to use in the future, we have already achieved high single-copy knock-in efficiency and also high homozygous knock-in efficiency using the constructs we describe in detail in **Supplementary Fig. 3 and 4**. As an example, out of randomly chosen 7 puromycin resistant clones, all were positive on at least one allele for EXSISERS$_{MAPT:10HaloTag}$ (new **Supplementary Fig. 21**).

$EXSISERS_{MAPT:10HaloTag}$ is our most complex construct (2.1 kbp without selection cassette and 4.4 kbp with selection cassette) containing two transmembrane segments and an extracellular HaloTag domain. Of the 7 positives, 3 were homozygous for $EXSISERS_{MAPT:10HaloTag}$.

With respect to ploidy, HEK293T cells, like many cell lines, are often triploid for most of the chromosomes, including chromosome 17, where *MAPT* is located. This property did not complicate CRISPR-Cas9-mediated genomic integration of the EXSISERS constructs.
Also, knock-in efficiency was good in human induced pluripotent stem cells (hiPSCs), which are known to be more difficult to modify by CRISPR, using the very same optimized components. When targeting exon 10, out of 21 picked clones, 14 (67%) were heterozygous, and 2 were homozygous (10%) for $EXSISERS_{MAPT:10NLuc}$, resulting in a total targeting efficiency of 76%. Similar targeting efficiency was achieved for exon 11 in hiPSCs for 15 picked clones with 11 clones being heterozygous (73%) and 3 clones (20%) being homozygous for $EXSISERS_{MAPT:11FLuc}$ (93% total efficiency).

As the Reviewer has already pointed out, homozygous targeting is indeed not necessary. We only used homozygous lines for subsequent analysis to show that EXSISERS is minimally invasive. Else, one could argue from the immunoblot analysis that the bands shown in, *e.g.*, **Fig. 2**, are from the untargeted WT allele. Thus, we can definitively conclude that the bands in our experiments are indeed the result of protein splicing. For standard experiments, heterozygous insertions can already be sufficient and can be obtained with high targeting efficiency.

R1P2:

2) *It is also important to prove that there is no effect on the endogenous transcript nor protein. That splicing occurs normally and the protein levels are not affected by insertion of these reporters and inteins. I don't think the authors have done this properly in the manuscript. Actually in Fig.2d, there is more 4R isoform in HEK than in the WT-EXISERS clone. Shouldn't these two cells be comparable? It is important to show that splicing patterns are not affected by insertion of these constructs, that protein levels are not affected, that function is not affected and that splicing could even change if necessary, such as in their iPS differentiation system. Also, can inteins have off target effects? This is not mentioned nor proved.*

Response to R1P2:

We have verified all EXSISERS lines carefully at the RNA and protein level and have added immunoblot (new **Supplementary Fig. 5,6,7,8,9 and 12**) and RT-qPCR (new **Supplementary Fig. 14**) data to show that there are no obvious detectable alterations in of the expressed isoforms and that all results from the EXSISERS reporters are in line with the data acquired on RNA level.

Concerning the variability of *MAPT* isoform patterns from different cells, it is important to mention that HEK293T is not a clonal cell line and showed some population variability. Analysis of HEK293T clones without *MAPT* modification showed only minor expression of 0N4R within a certain biological variation (**Supplementary Fig. 8**).

As per **Fig. 2d**, we have now performed densitometry on the 0N3R and 0N4R bands from an 16-bit uncompressed tiff file using the automated analysis from Image Lab (v6.1.0 build 7, Bio-Rad) and did not observe any obvious change in exon 10 inclusion between HEK293T

WT cells and EXSISERS$_{MAPT:10NLuc\text{-}11FLuc}$ cells, which both showed ~3% inclusion of 4R-tau (new **Supplementary Fig. 5b)**.

In comparison, the pathologic mutation IVS10+16 c>t increased the fractional inclusion by ~3.7-fold), which is comparable to what we see from dual-luciferase EXSISERS (~4-fold), new **Supplementary Fig. 10e**) and also in accordance with the literature (2–6-fold, DOI:10.1074/jbc.274.21.15134 and DOI:10.1016/j.molbrainres.2005.02.014).

In addition, we also included a new immunoblot where we showed that EXSISERS$_{MAPT:10NLuc\text{-}11FLuc}$ cells are also comparable to the parental HEK293T cells in its response towards small molecule splicing modulators, such as 5'-iodotubercidin (ITU) (new **Supplementary Fig. 11 and 12**).

As further evidence for the reporter lines' physiological state, we had shown in main **Fig. 2c** that the tau filaments are formed in EXSISERS$_{MAPT:10NLuc\text{-}11FLuc}$ cells. Since we chose to use only homozygous EXSISERS cell lines for all experiments, those filaments must be formed from tau proteins that underwent protein splicing.
Also, the functional aspects on RNA-level, such as the regulatory hairpin of *MAPT*, were functional after EXSISERS insertion, as the well-characterized hairpin-destabilizing IVS10+16 c>t mutation led to a dramatic increase of exon 10 inclusion. As seen in main **Fig. 2d,e, and h**, and the new **Supplementary Fig. 5b,6,7, and 8** all other clones of EXSISERS$_{MAPT\text{-}IVS10+16:10NLuc\text{-}11FLuc}$ IVS10+16 c>t always showed a more prominent inclusion of exon 10 (4R isoform) compared to the WT counterpart, unmodified HEK293T cells and their clones.

The behavior of the EXSISERS construct used to screen for splicing modulators of FOXP1 (EXSISERS$_{FOXP1:18b\text{-}BSD}$) also indicates that splicing was not affected, as cells with homozygous insertion of EXSISERS$_{BSD}$ exon 18b did not show a changed blasticidin S sensitivity compared to HEK293T WT cells (data not shown). Since a minimal lethal blasticidin S concentration of 3 µg/mL was applied, even a minor increase in exon 18b inclusion would result in a surviving population of cells. As the exon 18b inclusion rate was already 0 % for cells lacking a MBNL1/2 KO in EXSISERS$_{FOXP1:18b\text{-}BSD}$ and HEK293T WT cells (**Main Fig. 4d, e**), a decrease of the inclusion rate would not have been possible.

Importantly, in the case of EXSISERS, we do not have to predict where a splice modulator, such as MBNL1 could bind, as the entire gene locus is present.
This stands in stark contrast to minigenes, where only those parts of a gene that are suspected to be involved in the splicing regulation are included in an artificial reporter system, resulting in a biased or knowledge-based screen. Please also see the comparison of EXSISERS with minigenes as part of our response to Reviewer 3 (R3P1 and R3P2).

Regarding off-targets of intein-splicing: Inteins originated from prokaryotes, archaea, algal cells, yeast, and other fungi. The protein splicing mechanism relies on autocatalysis and thus does not use up or interfere with any host proteins, nucleic acids, or any other host factors. Inteins are used in all kingdoms of life for biotechnological applications such as heterologous utilization in vertebrates, including mammals that do not have any inteins in the genome natively. This heterologous usage in mammals, *e.g.*, to split Cas9 using protein trans-splicing in for rAAV delivery into pigs, did not show any side- or off-target effects on the organisms *in vivo* (doi:10.1038/s41551-019-0501-5 and doi:10.1038/s41591-019-0738-2). We have not made any observations in any of our EXSISERS implementations that would indicate such off-target effects.

R1P3:

3) A kind of related question: can you insert the NLuc/FLuc reporter anywhere in the exon regardless of the regulatory splicing sequences?
And how come increasing considerably the exon size has no effect on exon recognition and recruitment of the splicing machinery? As a splicing expert, it surprises me…

Response to R1P3:

In general, we carefully designed all EXSISERS constructs on the nucleotide level: we did use not only optimal mammalian codons but also avoided stable RNA secondary structures, and removed potential cryptic splice sites that may cause problems. We now included references to the software packages (Human Splice Finder v3.1 and NetGene2) in the Methods section under "Generation of stable EXSISERS cell lines with CRISPR/Cas9").

Regarding the insertion site, we emphasize the technical requirement for a Cys, Thr, or Ser in downstream of the insertion site (Ser and Thr are commonly found in regions containing loops and flexible linker amino acids). Furthermore, we paid attention to not modify any potential exonic splice enhancers and silencers/suppressors. For *MAPT* exon 10, there are 5 exonic splice modulators (doi: 10.1186/1750-1326-3-8), which were left intact upon insertion (see **Supplementary Fig. 9** for the insertion site of EXSISERS). We have also added a note to the method section that the insertion should be placed as distal as possible from exon-intron junctions to prevent undesired effects on RNA-splicing.

We also included data from an alternative insertion site (IS) of the alternatively spliced exon 10, which lies two amino acids (6 nt) downstream to the first IS. Again we took care not to disrupt known or potential splice enhancer/silencer motifs. The corresponding immunoblot did not reveal any obvious changes upon EXSISERS insertion at the 2nd site compared to unmodified HEK293T cells (**Supplementary Fig. 9**).

With respect to exon size, it has been suggested that large exon sizes are not a limiting factor in the identification of exons in alternative splicing (doi:10.1128/mcb.14.3.2140), which is in line with our experimental data. The prerequisite was that the inserted coding sequence did not contain any potential cryptic splice sites inducing aberrant splicing. In contrast, it has been suggested that the intron length has a major influence on alternative splicing, such as in the case of CD44 (doi:10.1128/mcb.18.10.5930).

We also designed our sgRNA in a way that the insertion of EXSISERS is sufficient to prevent Cas9 recutting, such that 'silent' synonymous codon substitutions are avoided, which can have unwanted side-effects as reported by Xiang *et al.* (10.1186/s13024-018-0280-6).

R1P4 and R1P5:

4) In Fig.2e, why there are equal levels of NLuc and FLuc in WT induced cells? If the exon is not included, NLuc should be lower than FLuc, right? Then with the use of 5-iodotubercidin, which induces e10 inclusion, in suppl Fig.6 there is increase of both 4R (+ex10) and 3R(-ex10) isoforms. How come? 3R should not increase…
5) Are the two splicing intein proteins equally efficient splicing out the Luc proteins (Gp41-1 and NrdJ-1)? Maybe Suppl Fig 5 was intended to study this, but I don't understand the results. Looks like for each NLuc signal there are 30 of FLuc, which makes FLuc more efficiently spliced. Was this corrected in the main figures? It is kind of important since usually we look at the relative levels of the alternatively spliced isoform vs total protein. If one intein

is more efficient than the other, it will affect interpretation of results. Also, can inteins splice out all the mRNAs translated? In a screening, can inteins be inhibited leading to indirect effects (no blasticidin not because there is no exon inclusion, but intein is inhibited or translation inhibited)?

Response to R1P4 and R1P5:

To adjust for the difference in the signal from FLuc and NLuc (due to differences in translation, half-life-time, enzyme activity, and brightness of the substrates), we expressed 0N4R-isoform from EXSISERS$_{MAPT:10NLuc-11FLuc}$ in which the two luciferases are driven at 1:1 stoichiometry by a Pgk1 promoter.

By transfecting increasing amounts of this plasmid, we established a linear relationship between the relative luminescence signals from FLuc and NLuc and determined that for our experimental settings, 30 RLUs of FLuc correspond to 1 RLU of NLuc, i.e., NLuc is 30-fold brighter than FLuc (original **Supplementary Figure 5b**, now **Supplementary Figure 2c**). As can be seen in the immunoblot (**Supplementary Fig. 2b**), this factor is not due to a difference in splice efficiency but rather a difference in substrate-dependent turnover rate and substrate/detection sensitivity.

In the new **Supplementary Fig. 5c**, we used this factor to adjust for the relative brightness and calculated the fraction of exon 10 inclusion to be ~5% in HEK293T-derived cells, in accordance with tau immunoblots (**Supplementary Fig. 5b**). The IVS10+16 c>t mutation led to a ~4-fold increase in exon 10 inclusion in the luciferase-based readout (**Supplemental Fig. 5c**), which matched the 3.7-fold increase, determined by immunoblot (**Supplemental Fig. 5b**).

Since the experiments of **Figure 3** are designed to show differential effects of pharmacological and genetic modulation of isoform expression, we have normalized all NLuc/FLuc data from EXSISERS$_{MAPT:10NLuc-11FLuc}$ to the control/baseline condition (induced *MAPT* but w/o perturbation), such that absolute differences in brightness are compensated, and differences due to the experimental perturbation can be directly read off the graphs.

We explained this normalization procedure in the figure legend, in the methods and statistics section.

Please also see our answers to R4PC2 and R4PC3.

With respect to your comment on the original Supplementary Fig. 6 (now embedded as **Supplementary Fig. 11a**), we thank the Reviewer for pointing out the inconsistency; indeed, the caption for this figure was mistakenly set. The caption was shifted by one position to the left; the legend has been corrected, and a new immunoblot has been inserted in the same as subfigure **b** with a finer titration of ITU. We are very sorry about this mistake and replaced the figure with a corrected version. Also, a similar immunoblot in direct comparison with EXSISERS$_{MAPT:10NLuc-11FLuc}$ has been inserted as new **Supplementary Fig. 12**.

R1P6:

6) Taking into consideration that the RNA is affected by using Cas13. It is important to show that the « protein » splicing effects observed with the inteins are also true at the RNA level by qRT-PCRs. e10 and total MAPT RNA levels should be affected accordingly in Fig.3. It is an important control.

Response to R1P6:

We have now performed RT-qPCRs experiments to validate all Cas13 key results of **Fig. 3** at the RNA level, *i.e.*,

a) Cas13d-NLS with an extended spacer is outperforming Cas13d-NLS with the originally published 22 nt spacer regarding general perturbation efficiency.
b) When Cas13d is applied in the nucleus using an isoform-specific spacer, it will still lead to a knock-down (KD) of all isoforms.
c) Cas13d applied on exon-junctions is more specific towards an isoform since it can only bind to the post-RNA-splicing mature mRNA.
d) shRNA is at least comparable if not superior to CRISPR/Cas13d or b, given that the latest miRNA scaffolds and the latest design rules are deployed. It also does not require the co-expression of two components (crRNA and Cas13).

R1P7:

7) In Fig.3c, why crRNA 10-11 is not affecting total MAPT levels but 9-10 is ? More puzzling, why the use of shRNAs to mimic miRNAs pathway has the opposite effect, it is the 9-10 that is more isoform specific than 10-11 ?

Response to R1P7:

We thank the Reviewer for this question regarding the details of **Fig. 3**.

In **Fig. 3c**, crRNA targeting exon 10-11 is clearly knocking-down 4R tau (NLuc) but seemingly not pan tau (FLuc). The reason is that the true fractional expression of 4R tau is very low (around 3-5%, please see R1P4 and R1P5 for details) compared to 3R tau (only very mature primary neurons in a complex 3D culture model are expressing a significant level of 4R tau (doi:10.1016/j.scr.2019.101541), thus even a 100% knock-down (KD) of 4R tau would just lead to an insignificant KD of pan tau.

The ^{2}9-10 crRNA is asymmetrically positioned on the 9-10 junction (=4R, **Fig. 3d**) and thus also matched almost perfectly on the 9-11 junction (3R, **Supplementary Fig. 16**) with only a single-nucleotide terminal mismatch (Cas13 systems tolerate single-nucleotide mismatches) resulting in the KD of all isoforms. For the 3rd generation shRNAs, the 9-10 microRNA (miR) was symmetrically positioned on the 9-10 junction (4R, **Fig. 3d**) and thus was specific for only 4R-tau since; an alignment of the 9-10 miR on the potential matching 9-11 junction (3R, **Supplementary Fig. 16**) showed 3 mismatches in the 5'-seed region (position 2–7) and thus was not activating the RNA-induced silencing complex (RISC) when accidentally bound to 3R.

In contrast, the 9-10 junction targeting miR was asymmetrically positioned onto the 9-10 junction (4R, **Fig. 3d**) due to design constraints of microRNAs and thus was also matching perfectly with its 5'-seed region (position 2–7) onto the 9-11 junction (3R, **Supplementary Fig. 16)** with only mismatches in its 3'-end that is tolerant towards mispairings.

Expectedly, the KD of 3R tau (crRNA targeting 9-11 junction) led to a clear decrease of pan tau signal (FLuc) without changing the 4R tau level (NLuc) in main **Fig. 3c**. This also has been confirmed in RT-qPCR in unmodified 293T cells in the new **Supplementary Fig. 14b**.
In summary, a strong depletion of pan tau (FLuc) in this cell line while trying knocking down 4R tau is clearly a side effect of lack of isoform specificity that can be observed for the exon 10 targeting crRNA and for the asymmetrical 9-10 junction targeting crRNA (^{2}9-10), while the crRNA targeting the 9-10 junction symmetrically (^{1}9-10) and the 10-11 junction are more specific.

R1P8:

8) Fig3f, dCasRX-SR effect is just 1,6x-fold. I don't think this is going to be biologically meaningful. The control in which there is dCasRx-SR or dCasRX-hnRNPA1 but not crRNA is missing (to make sure there are no indirect effects).

Response to R1P8:

The main objective of **Figure 3** is to show how EXSISERS technology can be used to optimize programmable effectors at the RNA level for modulating isoform-specific expression. We found a strong effect of the length of the guide RNA and the localization of the Cas13-effectors, while amiRNA was also very competitive.
To complete the picture, we also added data on the use of dead Cas13 systems for splicing modulation, because it is an application that is not possible with amiRNA.

We have now replicated the results on two independent clones, including the requested non-targeting controls (NTC) on another WT clone and also a clone carrying the IVS10+16 c>t mutation (**Supplementary Fig. 17**).
These results show that also small changes in isoform-specific expression can be quantified reliably with EXSISERS.
We did not express any opinion on whether the observed effects are biologically meaningful but simply suggest that EXSISERS can help to characterize and optimize systems that alter isoform-specific expression.

R1P9:

9) Again, the effect on Suppl Fig 12 seems very low too, 1,5x-fold. Is this sufficient to claim what the authors claim?

Response to R1P9:

We applied EXSISERS on a ribosomal-frameshifting-regulated gene to show EXSISERS' unique capability to monitor co-translational regulations, where RT-qPCR would fail. However, we did not claim a new finding. The observed effects are concentration-dependent and were independently confirmed with two complementary methods (fluorescence-activated cell scanning (FACS) and immunoblot analysis).

R1P10:

10) Why are the IFs in Fig2c and Supplementary Figure 11d,f so dotted at the nuclear level? Is this related to the reporter?

Response to R1P10:

Given that also unmodified HEK293T cells showed the 'nuclear dots' (new **Supplementary Fig. 5a**), they are likely a result of some unspecific binding of the pan-tau antibody (TAU-1 alias PC1C6) to nucleolar proteins in our immunofluorescence staining protocols.

R1P11:

11) For Fig.4, the CRSPR screening, it is important to know how many clones resisted to the blasticidin to know the false-positive rate of the system. The authors only show the positive MBNL1 clone, but this was already well known. Was the finding straightforward? It does not invalidate the proof-of-concept but it can give perspective on the feasibility of the system. It is known that some cells can escape the blasticidin selection. Were the authors using a higher amount of antibiotic than what is used for clone selection (1-10 ug/mL depending on the cell type)?

Response to R1P11:

We performed the experiment with a theoretical ~400-fold coverage of every sgRNA. The library contained ~80,000 sgRNAs against ~20,000 coding genes, including non-targeting control sgRNAs, resulting in 4 sgRNAs per gene. To achieve a ~400-fold coverage, we infected 100 × 10^6 cells with the lentiviral library with a multiplicity of infection (MOI) of ~0.3.
At least several hundred clones survived the most-stringent blasticidin selection condition (5 µg/ml). NGS analysis revealed that in this condition, 28.4% of the clones contained aN *MBNL1*-targeting lentiviral vector (composed of 18.8% and 9.6% of two different sgRNAs targeting *MBNL1*). Under low-pressure selection with the minimal inhibitory concentration of 3 µg/ml blasticidin-S, the flasks were confluent after the same timeframe. Still, based on the NGS analysis, 1.4% of the confluent population contained a lentivirus with a sgRNA targeting MBNL1. Also, based on NGS, only 0.0001% of the unselected control condition contained the same sgRNAs targeting MBNL1. This results in a 4 magnitudes of fractional enrichment in the 3 µg/ml blasticidin S condition and >5 magnitudes fractional enrichment for the more stringent 5 µg/ml blasticidin S condition. In other words, by simply subcloning the PCR product (instead of NGS) of the integrated lentiviral sgRNA expression cassette of the most stringent condition (5 µg/ml), followed by a standard Sanger sequencing of at least 20 clones, one would already expect 5-6 bacterial clones containing an *MBNL1*-targeting sgRNA. We emphasize that two independent sgRNAs targeting MBNL1 were independently enriched by 3 magnitudes (3 µg/ml blasticidin-S) and 4 magnitudes (5 µg/mL blasticidin-S) over the median sgRNA population. Importantly, we validated the screen on a different

EXSISERS$_{FOXP1:18b\text{-}BSD}$ clone using a 3rd independent sgRNA (different from the two enriched *MBNL1*-targeting sgRNAs of the library) targeting a constitutive *MBNL1* coding exon in parallel with a sgRNA targeting *MBNL2,* followed by blasticidin-S selection. Only the condition targeting *MBNL* genes led to blasticidin-S-resistant cells but targeting the control *AAVS1* locus did not. Moreover, when analyzing the surviving population via sequence decomposition of Sanger sequencing results, a dose-dependent accumulation of mutations in MBNL1 with increasing blasticidin-S concentration was indicative of functional coupling of the MBNL1-FOXP1-18b-Bsd-axis. With WT cells expectedly, we could not detect any resistant cells independently of any selection conditions and independently of the gene that was targeted. As described in **Fig. 4**, we used blasticidin-S in a concentration range the Reviewer indicated (3 µg/ml and 5 µg/ml are exactly in the range of 1-10 µg/ml).

Reviewer #2 (Remarks to the Author):

R2P0:

In this manuscript, the authors developed a new type of cell-based reporter system, exon-specific isoform expression reporter system (EXSISERS), which enables non-invasive detection of alternative splicing and exon-specific translation via intein-mediated protein splicing. They construct generated dual-luciferase (Nluc and Fluc) EXSISERS lines for ratiometric monitoring of different Tau protein isoforms, 3R-tau and 4R-tau. As designed, the system can recapitulate the expected change of different tau protein isoforms. The application of this reporter system was further demonstrated in several scenarios: 1. Screening of the effective guide RNAs in CRISPR/Cas-13 system that can achieve isoform-specific gene silencing; 2. Testing the activity of designer splicing enhancer or suppressor using the dCas-13 fusion protein containing SR domain or Gly-rich domain; 3. Measuring the co-translation ribosomal frameshift regulation. Finally, they generated an EXSISERS reporter for alternative splicing of exon 18b in FOXP1 and use the reporter to identify the regulators for isoform-specific expression of this exon via genome-wide CRISPR/Cas9 screen. Given their results the authors propose that it will be possible for an unbiased and non-invasive functional screening for splice modulators.

Overall I find the approaches employed in this study is valuable for characterizing and manipulating the intrinsic functionality of the exon-specific protein isoforms. However, the system is cumbersome to use and require a large amount of time for consecutive steps of CRISPR-cas insertion, which will limit its usefulness. In addition, some of the application did not perform as efficiently as previous system that was much simpler to generate. For example, the designer splicing enhancer and silencer using aCas-13 in EXSISERS reporter (Fig. 3f and 3g) was not as efficient as the engineered splicing factors using PUF fusion proteins (Wang Y et al, 2009 Nature Method, Wang Y et al 2013 NSMB), which is much simpler system to use. The authors should acknowledge such limitation and compare their system with previous system.

Response to R2P0:

We thank the Reviewer for acknowledging the value of EXSISERS to assess exon-specific protein isoform expression.
As we show in **Table R1**, EXSISERS has a unique set of advantages over other methods.

	EXSISERS	minigenes	immunoblot	immuno-cytochemistry	RT-qPCR	RNA-FISH
at endogenous site	☑		☑	☑	☑	☑
protein-level readout	☑		☑	☑		
cellular resolution	☑	☑		☑		☑
coupling of effectors to exon inclusion	☑	☑				
repeated measures	☑	☑				
no cell line needed			☑	☑	☑	☑

Table R1| Advantages of EXSISERS over alternative methods to detect isoform-specific expression

Although it is required to generate stable EXSISERS cell lines to ensure that isoform-specific expression is monitored at physiological levels, it is **not more cumbersome to generate those lines than it is to generate adequate minigenes**. Minigenes also have to be

integrated into the genome to not unphysiologically overload the splicing/expression machinery, which will lead to aberrant alternative splicing behavior, as reported for, *e.g.,* MAPT.
Please see a comparative analysis of two minigene systems for *MAPT* in our response to R3P2.

To ensure maximal convenience in producing EXSISERS lines, we have streamlined the process such that only a **single cloning step** is necessary to generate the all-in-one CRISPR/Cas9 plasmid and the targeting plasmid, that can be inserted into the genome within **2-3 days** (please see **Supplementary Fig. 3**, previous Supplementary Fig. 2). The CRISPR/Cas9-mediated insertion is sufficiently efficient with the plasmids we provide, such that within just **2 months**, clonal EXSISERS cell lines can be generated (**Supplementary Fig. 4**).
With respect to efficiencies using CRISPR-Cas9-mediated insertions, please see the detailed response to R1P1 for targeting efficiencies of EXSISERS.

With respect to Pumilio/PUF-based splicing modulators, we agree that they are powerful and we, therefore, had already cited Wang, Y., Cheong, C., Tanaka Hall, T. *et al*. "Engineering splicing factors with designed specificities." Nat Methods 6, 825–830 (2009), doi:10.1038/nmeth.1379 in our original submission.
Since Cas13-based splice modulators are still currently of broad interest, chose this system to show that EXSISERS can be used to optimize it, but the same is, of course, goes for Pumilio/PUF-based splicing modulators.

R2P1:

Specific concerns:
1. The intein used in this study were shown to have high splicing efficiency (Supplementary Fig. 1) in their system, however I am curious about how efficiently the intein works in different cell lines. Additional quantification should be performed to measure the intein excision rather than assuming it is always 100% excised.

Response to R2P1:

Inteins have indeed been shown to be effective upon heterologous expression in several mammalian cell types *in vitro* and *in vivo*. Most importantly, applications in mammals, such as splitting Cas9 to circumvent the limited packaging capacity of recombinant adeno-associated viruses (rAAVs), a commonly used viral vehicle for gene therapy, by harnessing trans-splicing inteins ('protein ligation' of two co-expressed polypeptides), were effective *in vivo* in pig and mouse models (doi:10.1038/s41591-019-0738-2, doi:10.1038/s41551-019-0501-5).
We have further improved the high splicing efficiency of the fast-splicing inteins (doi:10.1074/jbc.M112.372680) by adding coiled-coils (CCs) to support cooperative folding of the cis-splicing intein halves and its excision. We updated **Supplementary Fig. 1** with data for which we used mNeonGreen as extein as it is known to fold extremely rapidly in much less than 10 minutes. Thus, we reasoned that this extreme case of a fast-folding extein should be maximally sensitive to detect any unproductive folding intermediates.
Under these circumstances, the CCs-enhanced intein resulted in a higher product/educt-ratio compared to the CCs-less counterpart. C-cleavage side products could only be detected upon overexposure and contrast enhancement. We did not detect any N-cleavage products.

Upon request of the Reviewer, we have now included full immunoblots from multiple clones showing essentially no unspliced products for tau. Only under extreme overexposure, weak

bands appear at densities of less than <1% of the spliced products, which most likely correspond to the *de novo* translated proteins (**Supplementary Fig. 7**). Even for minigene-versions of EXSISERS$_{MAPT\text{:10NLuc-11FLuc}}$, which are heavily overexpressed at unphysiological levels, we could barely detect any unspliced educt (**Supplementary Fig. 10c,d**).

In addition to our experiments with HEK293T cells, we have observed similar results from murine neuroblastoma cells (N2a) in which housekeeping gene (*Tubb3*) was intact (**Supplementary Fig. 18m**). Here, too, no unspliced educts could be detected.

R2P2:

2. In Fig.2, since the study is focusing on the exon-specific isoforms of tau protein, the authors should use an exon10 specific tau antibody (or pan antibody for tau) to calibrate the system. This is to make sure that the results obtained from luciferase measurement correlate well with direct measurement of tau isoforms.

Response to R2P2:

Reliable tau-specific antibodies are hard to get by. Still, we had screened several anti-tau antibodies and found that the best way to reliably identify 4R tau is by comparing a 3R-immunoblot to pan-tau immunoblots. We proved that this band is indeed the 4R band in **Supplemental Fig. 11a).** However, the S/N-ratio of this 4R-antibody (doi:10.1186/s13024-017-0229-1) is low, and we also needed to see the fractional inclusion of 4R from total tau. Thus, the anti-pan-tau antibody was the most informative tool for our requirements.

When WT HEK293T cells were treated with ITU known to increase 4R tau (doi:10.1111/febs.12411), the 0N4R band (2nd band from below in anti-pan-tau immunoblot, **Supplementary Fig. 11**) was clearly increasing while 0N3R was decreasing (1st band from below in the anti-pan-tau immunoblot, **Supplementary Fig. 11**). Similarly, the bioluminescent signal from EXSISERS$_{MAPT\text{:10NLuc-11FLuc}}$ increased by ~4-fold (**Fig. 2f, j**) and longitudinally over a period of 60 hours in **Fig. 2h**.

In a direct comparison from unmodified HEK293T cells and EXSISERS$_{MAPT\text{:10NLuc-11FLuc}}$ in the same immunoblot, increasing ITU concentration resulted in a fractional increase of 4R tau. In contrast, the total tau level decreased slightly (new **Supplementary Fig. 12**). As expected for EXSISERS$_{MAPT\text{:10NLuc-11FLuc}}$, the OLLAS-positive band for excised NLuc (=4R) was getting more prominent with increasing ITU concentrations (**Supplementary Fig. 12**).

Furthermore, **Fig. 2d** showed that the IVS10+16 c>t mutation caused an ~3–4-fold increase of 4R-tau in both, immunoblot and in luciferase signal (**Fig. 2d,e and h, and Supplementary Fig. 5,6, and 7**). Please note that although the size separation and spatial resolution of the tau bands is high compared to typical anti-tau immunoblots in the literature (doi:10.1186/s13024-017-0229-1, doi:10.3892/ijmm.2012.1025), precise quantification of tau isoforms by densitometry is extremely challenging.

R2P3:

3. In Fig.4, I feel that this part lacks an important analysis on transcriptome level for the MBNL1/2-KO cells and the exon 18b inclusion cells after blasticidin selection. MBNL1/2 are key regulator in RNA splicing, and knock-out of these two genes should cause significant change of splicing in the level of entire transcriptome. I am wondering whether knock-out of these two genes could cause more exon-specific protein changes besides FOXP1.

Response to R2P3:

We agree with the Reviewer that it is interesting to ask which impact perturbations of MBNL proteins have on the transcriptome.

In our manuscript, however, it was the goal to present EXSISERS as a screening tool for unbiased identification of splicing modulators. Indeed, without any prior knowledge, we re-identified MBNL1 as the main regulator of *FOXP1* exon 18b inclusion using an unbiased lentiviral CRISPR/Cas9 screen, which was impossible before. We then followed up with a knockout of independent sgRNA targeting *MBNL1* to validate the results in our system.

With respect to the effects of MBNL on the transcriptome, we would like to refer to the thorough work of Han *et al.*, 2013 (doi:10.1038/nature12270), where they use RNA-seq profiling to analyze the impact of MBNL perturbations mediated by siRNAs. They showed that MBNL proteins negatively influence the global AS network important for pluripotency maintenance, partially by repressing the ES-cell-specific FOXP1 isoform, a stimulator of a core pluripotency circuit, thus promoting transcriptome-wide switch towards differentiation.

R2P4:

4. I think this paper may present a powerful tool to track and study exon-specific protein isoform. However, the authors should use it to investigate on new biological questions rather than only to confirm the conclusion people have already made.

Response to R2P4:

We thank the Reviewer for sharing enthusiasm towards EXSISERS as a 'powerful tool' to investigate alternatively spliced protein isoforms. While the main weight of such a methodological paper must clearly lie on the careful validation of the new instrument on the various technical levels against well-established results, we have made a few interesting observations showing the robustness and convenience of EXSISERS technology:

We showed for the first time,

a) the longitudinal readout of isoform-specific expression with cellular resolution of an alternatively spliced exon from the original genomic site in living cells,
b) an improved targeting efficiency of Cas13d significantly by the extension of the spacer length from 22 nt to 30 nt,
c) the importance to optimize the precise site of action for each programmable intervention tool (Cas13d or b, or shRNA in the cytosol) since it has a massive impact on the isoform specificity, even if the same position is targeted,
d) that shRNA - if carefully designed using the latest design rules and using up-to-date pri-microRNA biogenesis-mimicking scaffolds - can compete with Cas13-based systems regarding potency and isoform-specificity,
e) an independent confirmation of a serendipitous scientific finding of *FOXP1* exon 18b regulation via MBNL1 using a novel unbiased approach.

These examples lay out precise recipes for biological discoveries and there are already several laboratories in our network that are actively using EXSISERS technology to test their preferred biological hypothesis.

R2P5 (Minor P1):

Minor concern:

Overall the figures are poorly prepared with low resolution and confusing color scheme, more specifically:

1. The picture quality of Fig.2c and Fig.2g should be improved. The color and style of this figure should be modified to make it more reader friendly. In addition, Fig.2c and 2g should be showed in color to help understand.

Response to R2P5 (Minor P1):

We apologize that the quality of our figures was apparently compromised during compression. We are sorry for the compression artifacts of **Fig. 2** that occurred in the last submission. All our original figures are high quality.

R2P6 (Minor P2):

2. The picture quality of Fig.4c and Fig.4d should be improved. And the part (Identification of regulators for isoform-specific expression) and Fig.4 need be carefully reviewed, because the figure and the main text are not consistent.

Response to R2P6 (Minor P2):

We are sorry for the compression artifacts of **Fig. 4** that we improved. Furthermore, we thank the Reviewer for pointing out the disparity between main text and **Fig. 4**, we carefully re-read the main text and corrected inconsistencies with the figure.

R2P7 (Minor P3):

3. Supplementary Fig.8b need to be updated, as the resolution is very low.

Response to R2P7 (Minor P3):

We are sorry for the low quality of the original **Supplementary Fig. 8** (now improved in **Supplementary Fig. 19**). Regarding subfigure **b**, the GFP channel **did not show any signal** since in contrast to luciferases, endogenous expression of 4R tau did not yield enough protein to be readily detected in a common epi-fluorescence microscope.

R2P8 (Minor P4):

4. Similar to Fig. 2c, the supplementary Fig.11 and Fig.13 should be improved.

Response to R2P8 (Minor P4):

We improved the quality of the respective figures.

Reviewer #3 (Remarks to the Author):

R3P1:

Truong et al. develop a minimally invasive isoform-specific expression reporter system (EXSISERS) that incorporates translated and subsequently excised fast-splicing inteins with CC-domains into genes of interest. The authors demonstrate the utility of EXSISERS in a number of applications, ranging from the optimization of RNA-targeting strategies for exon-specific RNA degradation of MAPT mRNA, to the quantification of ribosomal frameshift-mediated regulations unmeasurable by RT-qPCR, to a phenotypic readout for a high-throughput screen of FOXP1 exon 18b inclusion that validates existing literature. Altogether, the presented work is a valuable addition to the isoform-specific RNA monitoring toolkit. While the generation of EXSISERS may be an involved process, nevertheless for some applications it might prove more useful than alternative methodologies, such as minigenes. I have a few major criticisms.

Response to R3P1

We thank the Reviewer for acknowledging the value of EXSISERS for monitoring isoform-specific expression. We have compiled **Table R1**, to compare the features of EXSISERS as compared with other relevant methods for detecting isoform-specific expression.

	EXSISERS	minigenes	immunoblot	immuno-cytochemistry	RT-qPCR	RNA-FISH
at endogenous site	☑		☑	☑	☑	☑
protein-level readout	☑		☑	☑		
cellular resolution	☑	☑		☑		☑
coupling of effectors to exon inclusion	☑	☑				
repeated measures	☑	☑				
no cell line needed			☑	☑	☑	☑

Table R1 | Advantages of EXSISERS over other methods to detect isoform-specific expression

Although many important findings were made possible by minigenes, they may (**1**) suffer from untruthful readout, (**2**) cause alterations of endogenous splicing, while (**3**) still requiring the same effort on cloning and generation of stable cell lines.

(1) Minigenes may lead to <u>untruthful readout of endogenous splice-regulation of a gene of interest</u> because they - with a high probability - do not contain all relevant regulatory elements. This is especially true for tau, where it has been shown that basically the whole intronic region is required to reflect the true splicing behavior for exon 10 (doi:10.1111/j.1471-4159.2004.02477.x). Most importantly, it has been shown recently that many identified SNPs have their origin deeply embedded within introns, such as the rs242561 polymorphism, that is protective against Parkinsonian disorders (doi:10.1016/j.celrep.2016.03.068). This single nucleotide polymorphism is located within the first intron 13.2 kbp upstream of the 2nd coding exon and 55 kbp downstream of the first coding exon; the same is true for rs242557 which is also associated Parkinson's disease, which is located 48 and 20 kbp down- and upstream from the flanking exons (doi:10.1016/j.neulet.2010.10.015, doi:10.18632/oncotarget.16490) or rs2471738 that lies

11.6 kbp upstream of the alternatively spliced exon 10 and 2 kbp downstream of exon 9 (doi:10.18632/oncotarget.16490). Moreover, many vertebrate genes are recursively spliced which will not be recapitulated by minigenes (doi:10.1038/nature14466). Also, for other alternatively spliced genes such as CD44, the intron's length determines the inclusion efficiency of the alternatively spliced exon (doi:10.1128/mcb.18.10.5930). A mini-gene version that contains truncated introns would therefore inevitably lead to unphysiological splicing. Thus, it is essentially impossible to faithfully recapitulate the complex regulatory machinery outside the precise three-dimensional context of the endogenous sites.

(2) Minigenes are not applicable to unbiased screens for splice regulators (such as genome-wide CRISPR/Cas9 KO-screens) to enrich a certain population of cells with a defined genetic perturbation. Minigenes are normally used in a transient transfection assay and even if integrated into the genome, they lay outside of the endogenous site and are driven by constitutive promoters. They are, therefore, hiding effects of (co)-transcriptional regulations. Also, the truncated introns cannot reflect the physiological genomic context such that whole-genome screens would probably yield questionable results.

(3) Minigenes can cause alterations of endogenous splicing of other collateral genes by competitive binding of splicing factors to the constitutively overexpressed minigene. This results in depletion from endogenous sites. In the case of *MAPT*, the altered isoform ratios can even feed-back on the splicing process since the formation of aggregated neurofibrillary tangles leads to the co-depletion of the otherwise soluble spliceosomal components further increasing the aberrant change of the global cellular splicing pattern (doi:10.1016/j.celrep.2019.08.104).

(4) Minigenes require the same effort to establish as EXSISERS
We made sure that the production of the EXSISERS lines is as convenient as possible: we provide all EXSISERS reporters in a respective cloning vector, such that only a single cloning step is required to obtain a customized exon-specific EXSISERS vector (please see **Supplementary Fig. 3**). The CRISPR/Cas9 vector, improved with enhanced gene targeting efficiency, can also be cloned in a **single** step (please see **Supplementary Fig. 3**). Please also see our graphical abstract of the process (**Supplementary Fig. 4**), which shows hot an EXSISERS clonal cell line can be established in just ~4–6 weeks. Please also see our response R1P1.

With respect to the effort for making the respective cell lines, minigenes also require the assembly of different fragments of truncated exon-intron fragments and subsequent cloning into a mammalian expression vector. Usually, several minigene versions with different truncations need to be tested, since truncations can lead to the removal of essential regulatory sequences, which are important for the regulation of alternative splicing.

Furthermore, minigene systems that are not read out via RT-qPCR but via a reporter system - which is essential for high-throughput detection - require additional modifications in the alternatively spliced exons to include stop or start codons for fluorescent proteins or luciferases. Alternatively, a frameshift-based reporter to distinguish the ab- or presence of an exon can be used. This, however, requires also a deletion/insertion of 1 or 2 nucleotides, since normally an alternatively spliced exon contains a number of nucleotides divisible by 3 (Stoilov *et al.* (doi:10.1073/pnas.0801661105), Luo *et al.* (doi:10.1002/cbic.201402069)).

Also, random integration of the minigene into the genome introduces an unnecessary variability due to copy number variation, impact on neighboring genes, expression strength,

and splicing behavior (doi:10.1016/j.cell.2010.11.056). Additionally, screening compound libraries to alternate AS, library-scale minigene transfection for every condition would not be economically feasible.
In summary, also for minigenes it is recommended to knock-in into a well-defined safe-harbor locus (such as *AAVS1/PPP1R12C* in human and *Rosa26* locus in murine systems) using CRISPR/Cas9 (or TALENs, ZFNs) to minimize variability.

Please also see our detailed response to your request in R3P2 where we also carefully compared minigenes with EXSISERS.

R3P2:

Major points:

1. The authors do not perform any head-to-head comparisons of EXSISERS to minigenes, which are comparatively much simpler and faster to generate. This should be done. If there is no clear advantage of EXSISERS, then it is worth wondering whether other researchers will adopt the new methodology.

Response to R3P2

Thank you also for the constructive suggestion to perform a head-to-head comparison with minigenes.
To this end, we have carefully studied the elaborate minigene systems for *MAPT* by Yu *et al.*, (doi:10.1111/j.1471-4159.2004.02477.x) and Jiang *et al.* (10.1128/mcb.20.11.4036-4048.2000 to construct corresponding minigene systems.

Before we compare our results shown in **Supplementary Fig. 10**, we need to quickly review the pertinent findings from Yu *et al.*, which is a very careful study that, however, also demonstrates the complexity and potential pitfalls for obtaining truthful results with minigenes.
It can be seen from **Figures 1 and 2 in Yu *et al.*** (attached below with figure legend) that the authors laboriously tried out 10 different tau-4R minigenes with different intronic truncations but found that none of them showed physiological splicing behavior. Only a plasmid made from a construct with **full-length** introns of 17,485 bp (LI9/LI10) recapitulates the endogenous physiological ratio. Similar behavior for minigenes also could be observed by Jiang *et al.* (Fig. 2B vs. Fig. 2A, doi:10.1128/mcb.20.11.4036-4048.2000). Besides, using full-length introns in minigenes is technically very difficult, since those introns can easily reach 5-digit bp in length and thus require specialized PCR-protocols to be amplified. Equipped with a plasmid backbone of ~3 kbp, promoter elements, and the rest of the tau coding sequence, this plasmid would also easily exceed the 20 kbp limit for classic plasmid transfection (doi:10.1093/nar/27.19.3792, doi:10.1016/j.ab.2005.08.029). Also, for plasmids greater than 20 kbp, the increased risks of plasmid instabilities enforce the usage of bacterial artificial chromosomes (BAC) instead.
Aberrant splice behavior of minigene systems has also been reported for other genes than *MAPT*. For the *ABCA4* gene (128 kbp, 50 exons), which plays a role in the Stargardt disease, Sangermano *et al.* (doi:10.1101/gr.226621.117) *'[...] discovered that when using small minigenes lacking the proper genomic context, in vitro results do not correlate with splice defects observed in patient cells.'* They *'[...] therefore devised a novel strategy in which a bacterial artificial chromosome was employed to generate midigenes, splice vectors of varying lengths (up to 11.7 kb)* ***covering almost the entire ABCA4 gene.****'* Only under these circumstances, a similar splicing behavior as observed in patients could be recapitulated.

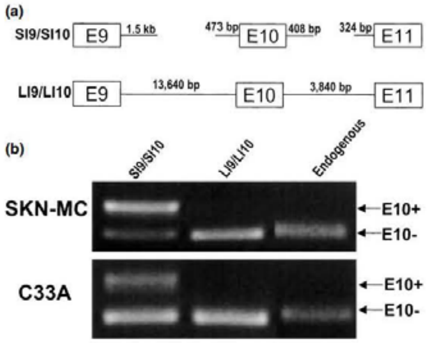

Fig. 1 Introns 9 and 10 affect splicing patterns of exon 10 in the *tau* gene. (a) Mini-gene constructs for splicing of exon 10 in the *tau* gene were generated in PCI-neo vector. The short previously published mini-gene SI9/SI10 includes exon 9, the first 1.5 kb and the last 473 bp of intron 9, exon 10, the first 408 bp and the last 324 bp of intron 10, and exon 11. The long mini-gene construct LI9/LI10 contains full length of both intron 9 and intron 10. (b) Mini-gene constructs were transfected into C33a or SKN-MC cells. Splicing patterns of exon 10 in mini-genes were examined by using RT–PCR. Splicing of exon 10 from the endogenous *tau* gene was detected in C33a cells or SKN-MC cells induced by 10 μM of sodium butyrate for 24 h.

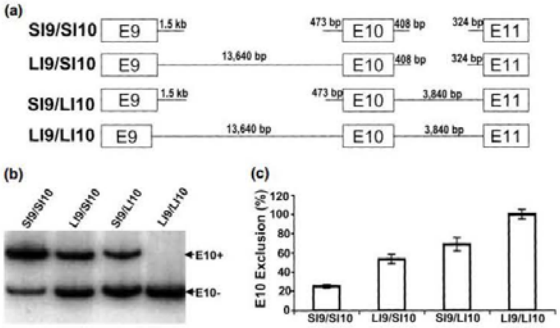

Fig. 2 Intron 9 and intron 10 additively contribute to correct splicing of exon 10 in the *tau* gene. (a) Constructs with a full-length intron 9 and a short intron 10 (LI9/SI10) or with a full-length intron 10 and a short intron 9 (SI9/LI10) were generated. The short intron 9 or short intron 10 was identical to that in SI9/SI10. (b) The constructs were transfected into SKN-MC cells. RT–PCR was used to determine splicing patterns of exon 10. (c) RT–PCR bands were quantitated using a phosphorImager. Bar represents the mean percentage of mRNA with exon 10 exclusion (E10 –) out of total mRNAs (E10 + and E10 –) from three separate transfection experiments. Error bars represent standard deviations of the means.

These results suggest that intronic truncations, an essential characteristic of minigenes, can be misleading, even if the minigene contained several hundred nucleotides of sequences down- and upstream of an exon of interest. Zheng *et al.* (doi:10.1101/gr.147546.112) also warned that *'[...] minigene reporters do not always recapitulate the regulation of endogenous exons. The minigene may not contain all of the relevant cis-regulatory elements for the test exon.'*
Recent reports (doi:10.1038/nature14466) also suggested that vertebrate introns, especially long ones, are often removed stepwise in a process called 'recursive splicing'. Thus, a minigene with truncated introns would inevitably lead to an altered RNA splicing behavior. Especially vertebrate introns can be larger than 100 kbp and can hardly be cloned fully in a minigene. Most importantly, those long introns are not just 'junk', which can be replaced by random nucleotide sequences.

For example, Wang *et al.* showed recently that the rs242561 polymorphism is protective against Parkinsonian disorders (doi:10.1016/j.celrep.2016.03.068). This single nucleotide polymorphism is located within the first intron 13.2 kbp upstream of the 2nd coding exon and 55 kbp downstream of the first coding exon; the same is valid for rs242557, which is also associated Parkinson's disease, which is located 48 and 20 kbp down- and upstream from the flanking exons (doi:10.1016/j.neulet.2010.10.015).
A stably integrated minigene is also preferred over transiently transfected plasmids, as Jiang *et al.* (doi:10.1128/mcb.20.11.4036-4048.2000) noted regarding the tau minigenes. They note '*[...] that transfected tau minigenes in these cells produced a slightly higher level of Tau4R compared to the endogenous tau expression pattern (Fig. 2), suggesting that overexpression of the tau minigene may titrate certain limiting factors controlling the ratio of Tau3R to Tau4R*'. Stoilov *et al.* (doi:10.1073/pnas.0801661105) also suggested that minigenes should be stably integrated: '*Note that transient expression of the reporters can lead to significant cell-to-cell variation in the protein signals, which we attribute to differences in the stability of the two proteins and in the amount of DNA taken up by each cell. This* ***variability is reduced in stable cell lines expressing the reporter*** *and with reporters where the stability of the two proteins is equalized*'.
Thus, the minigene systems are not easier to create, especially not as a version compatible with high-throughput screenings (*e.g.*, using terminally fused luciferases), which necessitates additional mutations have to be introduced into the coding sequence of the exon of interest.

Based on the luciferase minigene system described by Yu *et al.* (doi:10.1111/j.1471-4159.2004.02477.x), we build a minigene by amplifying the corresponding intronic regions with truncation that are of similar length as in Yu *et al.*, and Jiang *et. al.* (doi:10.1128/mcb.20.11.4036-4048.2000), to create EXSISERS-based 4R-minigenes (**Supplementary Fig. 10a**).
In accordance with Yu *et al.* and Jiang *et al.*, we noted an increased exon 10 inclusion level (~12%, **Supplemental Fig. 10c**) originating from minigenes as compared to the endogenous locus (~3–5%, **Supplemental Fig. 5b**).
For the mutation IVS10+16 c>t, 4R/pan-tau ratio further increased by roughly 2-fold to over 50%. In contrast, with integrated EXSISERS, we did not detect any significant difference between unmodified HEK293T cells, its clones, and $\mathrm{EXSISERS}_{\mathit{MAPT}\text{:10NLuc-11FLuc}}$ (**Fig. 2d, Supplementary 5, 6, 7, 8, 9, and 12**).
The reaction of $\mathrm{EXSISERS}_{\mathit{MAPT}\text{:10NLuc-11FLuc}}$ in response to small molecule perturbation (**Fig. 2f, h, j, and Supplementary 11 and 12**), and Cas13/microRNA-based modulation (**Fig. 3** *vs.* **Supplementary 14**) was similar to the reaction of unmodified HEK293T cells. Also, the disease-mimicking mutation IVS10+16 c>t lead to the expected 4-fold increase as reported in the literature (doi: 10.1074/jbc.274.21.15134, doi:10.1016/j.molbrainres.2005.02.014).

In summary, the head-to-head comparison of a minigene system and the EXISERS for *MAPT* showed clearly aberrant splicing behavior for the minigene but not EXSISERS as compared to unmodified cells. These findings are in line with several pieces of pertinent literature reviewed above.

R3P3:

2. The authors use CRISPR-Cas9 to integrate EXSISERS into areas of interest in the genome. When such knock-ins are performed and analyzed, typically researchers will generate multiple clonal cell lines, in case behavior in one cell line may be biased by unique Cas9-induced indel and/or template insertion off-target events. The authors should re-perform the experiments featured in Figures 3 and 4 (and associated supplemental figures) with at least one additional clonal cell line to demonstrate the generalizability of EXSISERS.

Response to R3P3:

We thank the Reviewer for this constructive criticism and agree that clonal lines may show different behavior in particular if SNPs, such as the *MAPT* IVS10+16 c>t mutation, are investigated. We have therefore included immunoblots to show that in all cases, homozygous c>t base transition in this regulatory intronic sequence led to an increase of the 4R/pan-tau inclusion-ratio in additional 9 clones (**Supplementary Fig. 6,7** in addition to the clonal line shown in **Fig. 2d**).

With respect to the experiments of **Fig. 3**, we validated the results regarding Cas13- or microRNA-mediated tau perturbation on unmodified HEK293T cells to exclude that the observed effects are artifacts on the post-translational level or by EXSISERS and performed an RNA-level quantification with RT-qPCR.
Using RT-qPCR, we confirmed that the extended 30 nt spacers are superior compared to the original 22 nt spacer in new **Supplementary Fig. 14a,** and the higher isoform specificity of targeting exon-junctions in new **Supplementary Fig. 14b**.

We also reproduced the minor effects of **Fig. 3f** in two independent $EXSISERS_{MAPT:10NLuc-11FLuc}$ clones (new **Supplementary Fig. 17**). In both clones, the combination of an exon 10 targeting crRNA together with a fusion of dRfxCas13d to the SR-rich domain of SC35 led to an increased 4R/pan-tau ratio. In contrast, the fusion to the Gly-rich domain of $hnRNPA1_{A1B}$ with a splice donor (SD) targeting crRNA decreased it (new **Supplementary Fig. 17**).

With respect to the experiments of **Fig. 4**, the results were already obtained from different clones. The lentiviral CRISPR/Cas9 KO library (**Fig. 4a and b**), as compared to the analyses in **Fig. 4c–f,** where an independent clone was used. We made this explicit into the caption of **Fig. 4.**

R3P4 (Minor P1):

Minor points:

1. The introduction would benefit from a reference to work on minigenes, as they are the main methodological competitor to EXSISERS.

Response to R3P4 (Minor P1):

We thank the Reviewer for this comment. We had already added references on minigenes in the main text in the introduction: *'Established methods for analyzing splicing isoforms either measure mRNA by endpoint-labeling (RT-qPCR, (sm)FISH[4], RNA-sequencing[5]), protein by immunochemistry (immunoblot analysis, immunofluorescence staining), or seek to mimic the genetic regulations via minigene analysis[6–8]'*

R3P5 (Minor P2):

2. The sentence should read "greater reduction": Expression of cytosolic PspCas13b-NES directed against the same region of exon 10 (Fig. 3e, orange bar) resulted in a greater reduction of FLuc as compared with the corresponding RfxCas13d-NLS (p<0.0001, post-hoc tests of one-way ANOVA) with comparable NLuc signal (p>0.05) (Fig. 3e, blue bar).

Response to R3P5 (Minor P2):

We thank the Reviewer for the suggestion, but indeed the knock-down (KD) of FLuc is 'less efficient' (leading to a 'weaker reduction' of FLuc) while NLuc depletion is as efficient as with Cas13d-NLS. We changed the whole sentence to: 'Expression of cytosolic PspCas13b-NES directed against the same region of exon 10 (**Fig. 3e, orange bar**) showed a better 4R-specificity due to decreased NLuc/FLuc-ratio compared with the corresponding RfxCas13d-NLS system ($p<0.001$, post-hoc tests of one-way ANOVA of $10/13d_{NLS}$ *vs.* $10/13b_{NES}$ *vs.* 9-10 amiRNA, **Fig. 3e, blue bar**).'

R3P6 (Minor P3):

3. The sentence should read "4f": Meanwhile, the enrichment of MBNL2 indels showed no dose-dependence (Fig. 4f).

Response to R3P6 (Minor P3):

We apologize for this mistake and corrected it.

Reviewer #4 (Remarks to the Author):

R4PA_B:

A. This work elegantly solves the current issues in quantifying protein expression levels by RNA-based approaches by incorporating a newly developed reporter system termed an exon-specific isoform expression reporter system (EXSISERS). The authors incorporated two EXSISERS into exons of interest (EOIs) by CRISPR/Cas9 and monitored the alternative splicing involved disease-associated exon inclusion of the patient-driven iPSC cells and screened RNA interference sequence for the isoform-specific expression to identify splice-regulators. Additionally, the authors similarly developed a survival reporter system for isoform-specific Blasticidin-S resistance marker. This article proposes the new exon-specific isoform expression reporter system would be a new tool for monitoring spatiotemporal exon-specific expression by imaging techniques.

B. This work is highly original and innovative with potential impacts in identifying splicing regulators and drug screening. Notably, this method could address the problems associated with protein expression level determined by RNA-based quantification methods. Thus, it is of significant importance and could be a game-changer for current RNA-based approaches if it is robust and reliable.

Response to R4PA_B:

We thank the Reviewer for acknowledging the advantages of EXSISERS' protein-level readout for drug screenings and basic research on identifying splicing regulators.

R4PC1:

C. In this system, there are several critical assumptions have not been controlled in this manuscript, which should be addressed in the manuscript before publications.

1. The manuscript is described as if protein trans-splicing has 100% efficiency (like Fig 2a, 2b). The splicing efficiency by protein trans-splicing is strongly affected by the junction sequence and the foreign exteins used. A single mutation near the junctions could abolish or deceased the splicing activity significantly, missing the controls to check the protein splicing efficiency.

Response to R4PC1:

We thank the Reviewer for this point regarding the efficiency of intein splicing.

In order to maximize efficiency, we chose fast-splicing inteins (doi:10.1038/s41467-020-15272-2, doi:10.1002/1873-3468.13909), which we further substantially enhanced with heterodimerization domains based on coiled-coils (CCs) (**Supplementary Fig. 1**). Recently, Bhagawati *et al.* (doi:10.1073/pnas.1909825116 showed in a similar approach, that intein splicing can be dramatically improved using a nanobody-antigen pair. By fusing an eGFP moiety to one half of a split-intein pair and an anti-GFP nanobody to the other split-intein counterpart, they could enable trans-splicing of a cysteine-free intein pair (important for extracellular protein splicing) that did not occur at all without the eGFP-nanobody interaction (please see their supplementary files Figure S10 vs. Figure S11).

These features enabled the very high splicing efficiency by immunoblot analysis of EXSISERS$_{\mathit{MAPT}\text{:10NLuc-11FLuc}}$ (**Supplementary Fig. 7**). Even when this construct was massively overexpressed via plasmid transfection, barely any unspliced proteins were detected (**Supplementary Fig. 2b**). Furthermore, the minigene version of this EXSISERS construct showed the same efficiency (**Supplementary Fig. 10c,d**).

As you requested in R4PF, the introduction of the terminal Asn→Ala mutation in the C-intein moiety completely disrupted protein splicing as expected (**Supplementary Fig. 2b**), thus indicating that the CCs-enhanced versions of the selected inteins are responsible for the exceptional high splicing efficiency.

With respect to considerations regarding the junction sequence, recent characterizations (doi:10.1002/1873-3468.13909) indicated that these 'ultrafast inteins' identified in metagenomic sources tolerate a broad spectrum of amino acids in heterologous settings very well (only proline is not tolerated heterologously and should be avoided). In conjunction with CCs, these efficiencies should increase even more.
In addition, we also now refer to the intein database in the method section under 'Application notes', which contains over 1000 inteins with known native extein sequences (maintained by the Iwaï lab, (InBase 2.0) https://inteins.biocenter.helsinki.fi/index.php), such that one can search for inteins with a desired native extein sequence to maximize the splicing efficiency.

R4PC2 and R4PC3:

2. Another assumption is similar to the previous one, FLuc and NLuc inserted in inteins fold into active equally with the same efficiency, yet having the same degradation rate in cells. The authors need to provide such experimental controls.
3. NLuc has 13-236 fold brighter than Fluc, according to the literature. All the data reported by normalized with the assumption, I believe.

Response to R4PC2 and R4PC3:

These assumptions do not have to be made. Instead, we measured the relative bioluminescence signal from *FLuc* and *NLuc* driven by a constitutive Pgk1 promoter at a 1:1 stoichiometry (**Supplementary Fig. 2a**). As seen in **Supplementary Fig. 2b**, the excision of NLuc/FLuc was very efficient. Moreover, we observed a linear relationship between the relative luminescence signals over 6 magnitudes and calculated NLuc yields 30 times more signal than FLuc (**Supplementary Fig. 2c**).

The Reviewer is also correct that for screening for modifiers of isoform expression, the bioluminescent signals were normalized to the control condition such that all relative differences between NLuc and FLuc are taken into account, and the effects of the perturbations can be directly seen. We have added additional notes in the figure legends and the manuscript to make the normalization procedure more explicit.

R4PC4:

4. The main caveat of this system easily overlooked by non-experts is the assumption that protein splicing by two split inteins has 100% or close to 100% efficiency. Particularly such high splicing activity for two orthogonal inteins has not been achieved in the past with an artificial system to my best knowledge. The reported efficiency of 95% in the cited ref.17 would result in the 90% efficiency for two orthogonal inteins. This assumption could determine the outcome of the analysis based on NLuc/FLuc quantification drastically.

Response to R4PC4:

As reported in subpoint R3PC1, we have used coiled-coil-enhanced fast-splicing inteins, and thus it is expected to have a greater efficiency than the reported value in the literature. We showed in **Supplementary Fig. 1** that CCs increased the protein splicing by nearly one magnitude (8.6-fold), which is exceptional considering the folding speed of the challenging surrogate extein mNeonGreen with less than 10 minutes (doi:10.1038/nmeth.2413). We have also conducted detailed immunoblot analysis of the dual-luciferase $EXSISERS_{MAPT:10NLuc-11FLuc}$

upon plasmid-based overexpression (new **Supplementary Fig. 2b**), when genomically integrated (new **Supplementary Fig. 5b**, new **Supplementary Figure 6 and 7**), and as overexpressed minigene variant (new **Supplementary Fig. 10c,d and** new **Supplementary Fig. 12**), and detected no relevant levels of unspliced products even not upon heavy overexpression and overexposure.

R4PD:

D. NLuc usually has 13-236 >times brighter than FLuc according to the literature, which is consistent with the data presented with Figure 2e. The NLuc/Fluc error bars cannot be smaller than each of them. However, Figure 2j and all other data presented in Figure 3 do not make any sense, statistically.

The error estimation (P-value analysis) needs to be reconsidered. There are two types of errors mixed: (1) Errors from the detection (readout values) and (2) errors from individual samples or measurements. Even when the calculated error estimated from 3 samples is small, the accuracy of the measurement cannot be better than the precision of the detection errors.

Response to R4PD:

NLuc is indeed ~30-fold brighter compared to FLuc in the dual-luciferase EXSISERS. Please see **Supplementary Fig. 2c** for the calibration we performed to adjust for the relative differences in the bioluminescent signal obtained from the two luciferases when expressed at 1:1 stoichiometry. We adjusted for those relative differences in brightness in **Fig. 2 and 3** by normalizing the relative luminescence units (RLU) to the reference condition (with *MAPT* induction but without perturbation), such that the relevant effects of the perturbation of exon-specific isoform expression can be more readily read from the figures. This procedure is described in the Figure legends, the Material and Methods section, and the Statistics section.

Concerning the error calculation, the purpose of the dual-luciferase EXSISERS is to extract a robust, ratiometric measure of isoform-specific expression (NLuc) corrected for overall gene expression of tau (FLuc). The range of isoform-specific expression is thus naturally dependent on the overall expression. The FLuc and NLuc signals are also experimentally dependent on the cell lysis step in the Promega detection workflow that we employed (https://www.promega.de/-/media/files/resources/protocols/technical-manuals/101/nanoglo-dual-luciferase-reporter-assay-protocol.pdf): FLuc substrate is provided together with a lysis buffer onto the cells, followed by the first measurement (FLuc); in the 2nd step, NLuc substrate is provided together with a FLuc inhibitor, followed by the 2nd measurement (NLuc). Thus, for every FLuc RLU data point, there is a matching NLuc data point (paired measurement).

To reduce the biological variability from pan-tau expression and experimental variability stemming from the lysis and detection procedure, it thus makes sense to take the NLuc/FLuc ratio from each sample's cell population and calculate the average and errors over cell populations.

Calculating the errors of NLuc and FLuc separately over the biological triplicates would instead discard the information that the NLuc/FLuc pair was obtained from the same sample and thus defeat the purpose of absorbing the main source of variability.

Although the main conclusions are supported by statistical analyses directly on the NLuc/FLuc ratios, we still find it informative to also display the FLuc and NLuc signals separately, to, *e.g.*, show the effects of tau induction for reference or show the effects of an extended crRNA spacer on pan-tau expression.

We have explained this aspect of data processing in the figure legend and the Statistics section.
For completion, we also show all individual data point on top of the bar graph and provide a comprehensive table showing detailed statistical results (**Supplementary Table 1**).

R4PE:

E.

As suggested in section C, D, and F, the validity of this system needs to be validated by additional controls. The authors should describe what would be potential pitfalls by the use of this reporter system. The current presentation does not provide sufficiently clear data to judge the validity and reliability of the system.

Response to R4PE:

We thank the Reviewer for the constructive suggestions of more data from control experiments to validate the experimental findings of the manuscript. We added RT-qPCR data (**Supplementary Fig. 14**) to confirm the key messages of **Fig. 3**. Furthermore, we added controls that the excision mechanism is indeed dependent on CCs-enhanced inteins by mutating the essential Asn of the C-inteins (C-gp41-1_{N37A} and C-NrdJ-1_{N40A}) (**Supplementary Fig. 2a,b**).
As requested, we have added paragraphs to the Materials and Methods section regarding the design criteria and potential pitfalls of EXSISERS constructs, the validation experiments to confirm efficient splicing of a given construct in analogy to our **Supplementary Figures 2,5,6,7, and 12**), a direct comparison to a minigene variant (**Supplementary Figure 10**), and detailed descriptions of how to generate clonal EXSISERS cell lines complementing **Supplementary Figures 3 and 4**.

R4PF:

F.

• There is no estimation of protein splicing efficiency for none of their protein splicing constructs except for mNG shown in Supplemental Fig. 1 by immunoblot. This data also does not give any estimate of the fully spliced vs by-products (non-spliced, N- and C-cleaved products). The supplemental Fig. 1 should be supplemented by immunoblotting and/or CBB-stained SDS-gels using, for example, anti-Ollas and Flag antibodies. The quantification by Nluc/Fluc ration will be strongly affected by the ligation efficiency, which is strongly dependent on the foreign extein and the splicing junctions.

Response to R4PF-part1

As requested, we updated **Supplementary Fig. 1.**, where we also now show an additional overexposed and contrast-enhanced image to detect all potential relevant side products. We also added full immunoblots in the new **Supplementary Fig. 7, Supplementary Fig. 10c,d and Supplementary Fig. 12**.

Regarding **Supplemental Fig. 1**, we deliberately chose mNeonGreen as a model Extein with extremely fast folding rates (<<10 minutes, doi:10.1038/nmeth.2413) to define a maximally high benchmark for the intein-splicing speed. We have now added a densitometric quantification of the immunoblot in **Supplemental Fig. 1**, which shows that the addition of coiled-coils as heterodimerization domains improves the product/educt ratio by ~9 fold.
We have also added a deliberately overexposed immunoblot on which a small amount of side-products from C-cleavage can be detected that, however, amount to only ~3%.

In comparison to this test system, we have conducted detailed analyses of the protein splicing in the dual-luciferase reporter system for exon 10 inclusion of MAPT ($EXSISERS_{MAPT:10NLuc-11FLuc}$). Full immunoblots from multiple clones show essentially no unspliced products for tau **(Supplementary Fig. 7)**. Only under extreme overexposure, weak bands appear at densities of less than <1% of the spliced products, which probably correspond to the de novo translated proteins.
Even when the dual-luciferase reporter construct was heavily overexpressed at unphysiological levels from a plasmid (**Supplementary Figure 2b**) or as a minigene-version (**Supplementary Fig. 10d**), we could barely detect any unspliced educt.

• What is the correlation between the quantification by immunoblotting (and/or mRNA quantification) vs NLuc/FLuc ratio for different constructs? Does it correlate well? if not, do they have a similar trend, which could be explained to some extent?

Response to R4PF-part2
We performed additional experiments for the key messages of **Fig. 3** in HEK293T cells and quantified them via RT-qPCR. The observed effects and quantities were comparable between luciferase-based readout of $EXSISERS_{MAPT:10NLuc-11FLuc}$ cells and RT-qPCR of unmodified HEK293T cells (see **Supplementary 15** *vs.* **Fig. 3**).
Densitometric analysis of **Fig. 2d** also correlated well with the luciferase-based readouts (see new **Supplementary Fig. 5b** *vs.* **Fig. 2e**).

• See also section D on the statistical data analysis.

Response to R4PF-part3
Please see R4PD regarding the statistical analysis.

• Fig.2d needs controls for protein-splicing deficient constructs by Ser-to-Ala and/or Asn-to-Ala.

Response to R4PF-part4
We added **Supplementary Fig. 2**, where we expressed the cloned 0N4R cDNA of $EXSISERS_{MAPT:10NLuc-11FLuc}$ with intein-inactivating mutations in the C-intein moiety. The results show that active inteins are indispensable for the generation of the desired unmodified WT 0N4R tau band.

• The authors claim "bio-orthogonal pair" of two inteins, but there is no such experimental evidence provided, including cited ref. 17. Trans-splicing is strongly dependent on the exteins, the authors could provide such data as a control, as this will affect the interpretation of the ratiometric data significantly. The orthogonality of two split intein should be demonstrated by using their systems because protein splicing by inteins is strongly extein-dependent.

Response to R4PF-part5
The inteins gp41-1 and NrdJ-1 have already been shown to be orthogonal by Pinto *et al.* (doi:10.1038/s41467-020-15272-2), which we cite in the main text.
We have not seen any mis-spliced products from these inteins, such as N-NrdJ-1- or C-gp41-1, which would have appeared as additional bands of lower molecular weight on the immunoblots (**Supplementary Fig. 2b,7, 10c, and 12**).

Moreover, the orthogonal pairs of coiled-coils, which likely dimerize already at the secondary structure level before any intein or extein segments can fold, add a second level of orthogonality.

• The author provided only one experimental data in Supplemental Fig 1 of immunoblotting and did not disclose any further sequence in detail. At least Supplemental Fig. 1 could be supplemented by covering all possible products using anti-Olla and Flag antibodies and provide the protein splicing efficiency quantitated for each of the two splicing steps. In theory, cleaved products might not interfere with NLuc/Fluc ratio. Do the authors have any evidence to assume that is the case?

Response to R4PF-part6
We updated **Supplementary Fig. 1** with an overexposed and contrast-enhanced immunoblot. We see a weak band for C-cleavage (~3%) using the fast-folding mNeonGreen as a surrogate extein sequence. Via densitometry, we could quantify that the addition of Coiled-Coils could enhance the protein splicing efficiency by ~9 fold. Please also see the full immunoblots in **Supplementary Fig. 7, 10c, and 12**, which show that the splicing efficiency was even higher for both inteins together with >99%.

• The main claims generally focus on the Ratio-metric assay using NLuc/Fluc, the survival system using BSD could be more confusing for readers than making it clear to understand the reporter system as currently written.

Response to R4PF-part7
We appreciate the Reviewer's suggestion but still find it valuable to showcase the versatility of the EXSISERS technology that goes beyond reporter signals. The capability to non-invasively couple the in- or exclusion of an exon to cell survival enables unbiased screenings for new splicing regulators, such as genome-wide CRISPR/Cas9-mediated KO screens. This powerful methodology was not possible before.
As an extension, one could also imagine to use dCas9-activator screens or instead use a triggerable toxin such as HSV-Tk, to screen for exon exclusion instead of inclusion.

R4PH:
H.
• The abstract is concise and clear.
• There are several misleading statements in the introduction, the authors claim "fast" protein splicing but no speed or relevant time scale is given. Protein splicing is strongly context-dependent, has to be investigated for each extein. This claim is thus not validated in the manuscript. Moreover, there is no information about "trace-less" because the authors do not disclose the protein sequence for junction regions. "Traceless" should mean the spliced sequence is identical to the original protein sequence without a single mutation. Is this the case?
• The current data is not sufficiently supporting the conclusion because of several assumptions and lacks critical controls to verify each of the critical assumptions.

Response to R4PH:
We have now added a series of additional control experiments to further support that the very efficient intein splicing does not alter the physiological isoform expression and are thus scarless.

To initially investigate and optimize the splicing efficiency of the inteins, we created a construct using mNeonGreen as an extein with folding rates of <10 minutes (please see Supplementary Fig. 1). Even under these extreme conditions, our final design, including coiled-coils (CCs) achieved a significantly greater extein to intein-extein ratio, indicating higher protein splicing efficiency (~9-fold increase in efficiency, **Supplementary Fig. 1**).

Nevertheless, the Reviewer is, of course, right that our measurements did not include precise timing and therefore we have changed the term from 'fast' to 'efficient' in the abstract and the introduction. Still, we used the term 'fast' in the beginning of the results section when we refer to gp41-1 and NrdJ-1 inteins since the literature described them as ultrafast splicing inteins (doi:10.1073/pnas.1701083114, doi:10.1021/jacs.7b02618).

Application of EXSISERS on *MAPT* showed a very high protein splicing efficiency (**Supplementary Fig. 7, Supplementary Fig. 10c, and Supplementary Fig. 12**). Please also refer to the detailed answer to R4PF. With the 'classic inteins', such as Ssp or Npu DnaE, intein splicing is highly dependent on the extein sequences, but with those 'ultrafast inteins' identified in metagenomic sources, the literature (doi:10.1002/1873-3468.13909) showed that they tolerate heterologous settings very well (only proline is not tolerated by all inteins in a heterologous context).

Besides the recently discovered classes of fast and efficient inteins, we like to refer to the nicely maintained database from the Iwaï lab (formerly maintained by New England Biolabs), where one can screen for inteins where the native extein sequences are identical or similar to the desired insertion site. As an example, we used this database, to search for inteins suitable to split Cas9 between position 573 and 574 (KIE|CFD), *Npu* intein with the native extein sequence (AEY|CFN) which critical +2 position fits to the intended Cas9 split-site (doi:10.1093/nar/gkv601). Notably, we did not see any difference in activity between WT Cas9 and Npu intein split-Cas9.

We neither introduced any extra Ser/Cys/Thr, nor did we change any amino acid to Ser/Cys/Thr, but merely used the natively occurring Ser/Cys/Thr of an exon, therefore we consider it justified to use the term 'traceless' or 'scarless'. Please also see the Materials and Methods section 'Generation of stable cell lines with tagged exons via CRISPR/Cas9', where we described how we inserted EXSISERS into the GOI.

We also added additional experimental controls, such as RT-qPCR on unmodified HEK293T cells (**Supplementary Fig. 14**) data to substantiate our data from **Fig. 3** in $EXSISERS_{\textit{MAPT}:10NLuc\text{-}11FLuc}$ cells. We also added additional dual-luciferase assays data from other clones to exclude clone-dependent artifacts (**Supplementary Fig. 17**). Moreover, we included additional full-range immunoblots to show the high protein splicing efficiency of the CCs-improved inteins (**Supplementary Fig. 7, Supplementary Fig. 10c,d,, and Supplementary Fig. 12**).

Decision Letter, second revision:

Date: 19th October 20 21:06:24
Last Sent: 19th October 20 21:06:24
Triggered By: Jie Wang
From: jie.wang@nature.com
To: gil.westmeyer@tum.de
Subject: Decision on Nature Cell Biology submission NCB-W40046B-Z
Message: Dear Professor Westmeyer,

Thank you for your email asking us to reconsider our decision on your manuscript, "Non-invasive and high-throughput interrogation of exon-specific isoform expression". We are always willing to hear the authors' perspective, but we must first prioritize decisions on new submissions. We appreciate your patience while we considered this appeal.

I have now discussed your manuscript, and the referees' comments and your rebuttal, in detail with my colleagues, and we would be willing to reconsider a revised manuscript provided the following issues can be addressed, and that nothing similar is accepted for publication at Nature Cell Biology or published elsewhere in the meantime.

Please pay close attention to our guidelines on statistical and methodological reporting (listed below) as failure to do so may delay the reconsideration of the revised manuscript. In particular please provide:

- a Supplementary Figure including unprocessed images of all gels/blots in the form of a multi-page pdf file. Please ensure that blots/gels are labeled and the sections presented in the figures are clearly indicated.

- a Supplementary Table including all numerical source data in Excel format (currently shown in PDF format), with data for different figures provided as different sheets within a single Excel file. The file should include source data giving rise to graphical representations and statistical descriptions in the paper and for all instances where the figures present representative experiments of multiple independent repeats, the source data of all repeats should be provided.

We cannot, of course, predict the outcome of the re-review process. Although our referees were very well placed to evaluate this work, if deemed necessary we may choose to involve an additional referee in the event of resubmission, and any additional points that this referee may raise would have to be addressed as well.

On resubmission please provide the completed Editorial Policy Checklist (found here https://www.nature.com/documents/nr-editorial-policy-checklist.pdf), and Reporting Summary (found here https://www.nature.com/documents/nr-reporting-

summary.pdf). This is essential for reconsideration of the manuscript and these documents will be available to editors and referees in the event of peer review. For more information see below. Please also ensure that the presentation of statistical information in the revised submission complies with Nature Cell Biology's statistical guidelines (see below).

Please use the link below to submit the complete manuscript files, and include a point-by-point response to the complete reviewer comments, verbatim as provided in their reports.

[REDACTED]

With kind regards,

Jie Wang

Jie Wang, PhD
Senior Editor
Nature Cell Biology

Tel: +44 (0) 207 843 4924
email: jie.wang@nature.com

GUIDELINES FOR EXPERIMENTAL AND STATISTICAL REPORTING

REPORTING REQUIREMENTS – To improve the quality of methods and statistics reporting in our papers we have recently revised the reporting checklist we introduced in 2013. We are now asking all life sciences authors to complete two items: an Editorial Policy Checklist (found here https://www.nature.com/documents/nr-editorial-policy-checklist.pdf) that verifies compliance with all required editorial policies and a reporting summary (found here https://www.nature.com/documents/nr-reporting-summary.pdf) that collects information on experimental design and reagents. These documents are available to referees to aid the evaluation of the manuscript. Please note that these forms are dynamic 'smart pdfs' and must therefore be downloaded and completed in Adobe Reader. We will then flatten them for ease of use by the reviewers. If you would like to reference the guidance text as you complete the template, please access these flattened versions at http://www.nature.com/authors/policies/availability.html.

STATISTICS – Wherever statistics have been derived the legend needs to provide the n number (i.e. the sample size used to derive statistics) as a precise value (not a range), and define what this value represents. Error bars need to be defined in the legends (e.g. SD, SEM) together with a measure of centre (e.g. mean, median). Box plots need to be defined in terms of minima, maxima, centre, and percentiles. Ranges are more appropriate than standard errors for small data sets. Wherever statistical significance has been derived, precise p values need to be provided and the statistical test used needs to be stated in the legend. Statistics

such as error bars must not be derived from n<3. For sample sizes of n<5 please plot the individual data points rather than providing bar graphs. Deriving statistics from technical replicate samples, rather than biological replicates is strongly discouraged. Wherever statistical significance has been derived, precise p values need to be provided and the statistical test stated in the legend.

Information on how many times each experiment was repeated independently with similar results needs to be provided in the legends and/or Methods for all experiments, and in particular wherever representative experiments are shown.

We strongly recommend the presentation of source data for graphical and statistical analyses as a separate Supplementary Table, and request that source data for all independent repeats are provided when representative experiments of multiple independent repeats, or averages of two independent experiments are presented. This supplementary table should be in Excel format, with data for different figures provided as different sheets within a single Excel file. It should be labelled and numbered as one of the supplementary tables, titled “Statistics Source Data”, and mentioned in all relevant figure legends.

Author Rebuttal, third revision:

Reviewer #1 (Remarks to the Author):

In this manuscript, Truong et al. describe a new tool, EXSISERS, to assess changes in splicing isoforms at the protein level and/or tag cells with specific protein splicing isoforms. The authors are taking advantage of the capacity of inteins to splice themselves out at the protein level, without affecting the RNA or coding sequence where there are integrated in. Using these inteins, they have shown with a wide set of examples, how they can insert at the endogenous level, in the alternatively spliced exon of choice, a reporter that can be spliced out at the protein level by specific inteins. With this system, by looking at expression of the protein reporter, which can be a luciferase protein, a blasticidine resistant gene, a fluorescent protein, an halo tag that goes to the membrane for cell sorting, one can identify, quantify, cell sort, live image cells expressing a specific splicing isoform of interest without the need of artificial reporters, splicing-specific antibodies, or the need to rely on RNA-based methodologies that most of the times are not impacting proteins at the same level. With this new system, one can assess the real splicing isoforms that exist at the protein level, follow them, manipulate them and even use them as a read out for CRISPR screening, imaging and sorting. It is extremely versatile and useful for studying many mechanisms and more importantly the biological relevance of a particular splicing variant at the protein level, and not the RNA level as we usually do (which underestimates all the post-transcriptional effects that could come from the new splice variant). Moreover, in the manuscript, the use of RfxCas13d and PspCas13b to specifically knock down one specific splicing isoform is also studied, bringing light to this also new and poorly understood tool. Key aspects of the crRNA design and if it is better to target the nascent pre-mRNA or the mature mRNA are shown.
Overall the manuscript is clear, robust and full of insightful new tools and recommendations to work with specific splicing isoforms at all possible levels. It is therefore of great interest for the scientific community and deserves publication if some concerns are addressed first.

Comments:

R1P1:

1) *Since this is a manuscript selling a new tool, it would be nice if the authors comment whether it is difficult to endogenously tag at the homozygous level such reporter sequences. Have they tried many different type of cells? Which is the size of the biggest reporter they successfully inserted? I say this, because it is known that not all cells are easy to CRISPR tag and it is even more difficult to tag the two alleles, and even more two regions of the same gene at the two alleles. What happens in cells with more than two alleles? Is it really important to tag all alleles? All this could be commented to reinforce feasibility.*

Response to R1P1:

We thank the Reviewer for acknowledging the value of EXSISERS for studying isoform-specific expression.
With respect to cell types, we tested HEK293T, Neuro-2a, and several human induced pluripotent stem cell lines. Homozygous knock-ins were also achieved by a collaboration partner using an unrelated gene in HepG2 and HCT116 cells.
Although the efficiency of CRISPR/Cas9 type of gene editing tools will surely further improve and make systems such as EXSISERS even more convenient to use in the future, we have already achieved high single-copy knock-in efficiency and also high homozygous knock-in efficiency using the constructs we describe in detail in **Supplementary Fig. 3 and 4**. As an example, out of randomly chosen 7 puromycin resistant clones, all were positive on at least one allele for EXSISERS$_{MAPT:10HaloTag}$ (new **Supplementary Fig. 21**).

$EXSISERS_{MAPT:10HaloTag}$ is our most complex construct (2.1 kbp without selection cassette and 4.4 kbp with selection cassette) containing two transmembrane segments and an extracellular HaloTag domain. Of the 7 positives, 3 were homozygous for $EXSISERS_{MAPT:10HaloTag}$.

With respect to ploidy, HEK293T cells, like many cell lines, are often triploid for most of the chromosomes, including chromosome 17, where *MAPT* is located. This property did not complicate CRISPR-Cas9-mediated genomic integration of the EXSISERS constructs.
Also, knock-in efficiency was good in human induced pluripotent stem cells (hiPSCs), which are known to be more difficult to modify by CRISPR, using the very same optimized components. When targeting exon 10, out of 21 picked clones, 14 (67%) were heterozygous, and 2 were homozygous (10%) for $EXSISERS_{MAPT:10NLuc}$, resulting in a total targeting efficiency of 76%. Similar targeting efficiency was achieved for exon 11 in hiPSCs for 15 picked clones with 11 clones being heterozygous (73%) and 3 clones (20%) being homozygous for $EXSISERS_{MAPT:11FLuc}$ (93% total efficiency).

As the Reviewer has already pointed out, homozygous targeting is indeed not necessary. We only used homozygous lines for subsequent analysis to show that EXSISERS is minimally invasive. Else, one could argue from the immunoblot analysis that the bands shown in, *e.g.*, **Fig. 2**, are from the untargeted WT allele. Thus, we can definitively conclude that the bands in our experiments are indeed the result of protein splicing. For standard experiments, heterozygous insertions can already be sufficient and can be obtained with high targeting efficiency.

R1P2:

2) *It is also important to prove that there is no effect on the endogenous transcript nor protein. That splicing occurs normally and the protein levels are not affected by insertion of these reporters and inteins. I don't think the authors have done this properly in the manuscript. Actually in Fig.2d, there is more 4R isoform in HEK than in the WT-EXISERS clone. Shouldn't these two cells be comparable? It is important to show that splicing patterns are not affected by insertion of these constructs, that protein levels are not affected, that function is not affected and that splicing could even change if necessary, such as in their iPS differentiation system. Also, can inteins have off target effects? This is not mentioned nor proved.*

Response to R1P2:

We have verified all EXSISERS lines carefully at the RNA and protein level and have added immunoblot (new **Supplementary Fig. 5,6,7,8,9 and 12**) and RT-qPCR (new **Supplementary Fig. 14**) data to show that there are no obvious detectable alterations in of the expressed isoforms and that all results from the EXSISERS reporters are in line with the data acquired on RNA level.

Concerning the variability of *MAPT* isoform patterns from different cells, it is important to mention that HEK293T is not a clonal cell line and showed some population variability. Analysis of HEK293T clones without *MAPT* modification showed only minor expression of 0N4R within a certain biological variation (**Supplementary Fig. 8**).

As per **Fig. 2d**, we have now performed densitometry on the 0N3R and 0N4R bands from an 16-bit uncompressed tiff file using the automated analysis from Image Lab (v6.1.0 build 7, Bio-Rad) and did not observe any obvious change in exon 10 inclusion between HEK293T

WT cells and EXSISERS$_{MAPT:10NLuc\text{-}11FLuc}$ cells, which both showed ~3% inclusion of 4R-tau (new **Supplementary Fig. 5b)**.

In comparison, the pathologic mutation IVS10+16 c>t increased the fractional inclusion by ~3.7-fold), which is comparable to what we see from dual-luciferase EXSISERS (~4-fold), new **Supplementary Fig. 10e**) and also in accordance with the literature (2–6-fold, DOI:10.1074/jbc.274.21.15134 and DOI:10.1016/j.molbrainres.2005.02.014).

In addition, we also included a new immunoblot where we showed that EXSISERS$_{MAPT:10NLuc\text{-}11FLuc}$ cells are also comparable to the parental HEK293T cells in its response towards small molecule splicing modulators, such as 5'-iodotubercidin (ITU) (new **Supplementary Fig. 11 and 12**).

As further evidence for the reporter lines' physiological state, we had shown in main **Fig. 2c** that the tau filaments are formed in EXSISERS$_{MAPT:10NLuc\text{-}11FLuc}$ cells. Since we chose to use only homozygous EXSISERS cell lines for all experiments, those filaments must be formed from tau proteins that underwent protein splicing.
Also, the functional aspects on RNA-level, such as the regulatory hairpin of *MAPT*, were functional after EXSISERS insertion, as the well-characterized hairpin-destabilizing IVS10+16 c>t mutation led to a dramatic increase of exon 10 inclusion. As seen in main **Fig. 2d,e, and h**, and the new **Supplementary Fig. 5b,6,7, and 8** all other clones of EXSISERS$_{MAPT\text{-}IVS10+16:10NLuc\text{-}11FLuc}$ IVS10+16 c>t always showed a more prominent inclusion of exon 10 (4R isoform) compared to the WT counterpart, unmodified HEK293T cells and their clones.

The behavior of the EXSISERS construct used to screen for splicing modulators of FOXP1 (EXSISERS$_{FOXP1:18b\text{-}BSD}$) also indicates that splicing was not affected, as cells with homozygous insertion of EXSISERS$_{BSD}$ exon 18b did not show a changed blasticidin S sensitivity compared to HEK293T WT cells (data not shown). Since a minimal lethal blasticidin S concentration of 3 µg/mL was applied, even a minor increase in exon 18b inclusion would result in a surviving population of cells. As the exon 18b inclusion rate was already 0 % for cells lacking a MBNL1/2 KO in EXSISERS$_{FOXP1:18b\text{-}BSD}$ and HEK293T WT cells (**Main Fig. 4d, e**), a decrease of the inclusion rate would not have been possible.

Importantly, in the case of EXSISERS, we do not have to predict where a splice modulator, such as MBNL1 could bind, as the entire gene locus is present.
This stands in stark contrast to minigenes, where only those parts of a gene that are suspected to be involved in the splicing regulation are included in an artificial reporter system, resulting in a biased or knowledge-based screen. Please also see the comparison of EXSISERS with minigenes as part of our response to Reviewer 3 (R3P1 and R3P2).

Regarding off-targets of intein-splicing: Inteins originated from prokaryotes, archaea, algal cells, yeast, and other fungi. The protein splicing mechanism relies on autocatalysis and thus does not use up or interfere with any host proteins, nucleic acids, or any other host factors. Inteins are used in all kingdoms of life for biotechnological applications such as heterologous utilization in vertebrates, including mammals that do not have any inteins in the genome natively. This heterologous usage in mammals, *e.g.*, to split Cas9 using protein trans-splicing in for rAAV delivery into pigs, did not show any side- or off-target effects on the organisms *in vivo* (doi:10.1038/s41551-019-0501-5 and doi:10.1038/s41591-019-0738-2). We have not made any observations in any of our EXSISERS implementations that would indicate such off-target effects.

R1P3:

3) A kind of related question: can you insert the NLuc/FLuc reporter anywhere in the exon regardless of the regulatory splicing sequences?
And how come increasing considerably the exon size has no effect on exon recognition and recruitment of the splicing machinery? As a splicing expert, it surprises me…

Response to R1P3:

In general, we carefully designed all EXSISERS constructs on the nucleotide level: we did use not only optimal mammalian codons but also avoided stable RNA secondary structures, and removed potential cryptic splice sites that may cause problems. We now included references to the software packages (Human Splice Finder v3.1 and NetGene2) in the Methods section under "Generation of stable EXSISERS cell lines with CRISPR/Cas9").

Regarding the insertion site, we emphasize the technical requirement for a Cys, Thr, or Ser in downstream of the insertion site (Ser and Thr are commonly found in regions containing loops and flexible linker amino acids). Furthermore, we paid attention to not modify any potential exonic splice enhancers and silencers/suppressors. For *MAPT* exon 10, there are 5 exonic splice modulators (doi: 10.1186/1750-1326-3-8), which were left intact upon insertion (see **Supplementary Fig. 9** for the insertion site of EXSISERS). We have also added a note to the method section that the insertion should be placed as distal as possible from exon-intron junctions to prevent undesired effects on RNA-splicing.

We also included data from an alternative insertion site (IS) of the alternatively spliced exon 10, which lies two amino acids (6 nt) downstream to the first IS. Again we took care not to disrupt known or potential splice enhancer/silencer motifs. The corresponding immunoblot did not reveal any obvious changes upon EXSISERS insertion at the 2nd site compared to unmodified HEK293T cells (**Supplementary Fig. 9**).

With respect to exon size, it has been suggested that large exon sizes are not a limiting factor in the identification of exons in alternative splicing (doi:10.1128/mcb.14.3.2140), which is in line with our experimental data. The prerequisite was that the inserted coding sequence did not contain any potential cryptic splice sites inducing aberrant splicing. In contrast, it has been suggested that the intron length has a major influence on alternative splicing, such as in the case of CD44 (doi:10.1128/mcb.18.10.5930).

We also designed our sgRNA in a way that the insertion of EXSISERS is sufficient to prevent Cas9 recutting, such that 'silent' synonymous codon substitutions are avoided, which can have unwanted side-effects as reported by Xiang *et al.* (10.1186/s13024-018-0280-6).

R1P4 and R1P5:

4) In Fig.2e, why there are equal levels of NLuc and FLuc in WT induced cells? If the exon is not included, NLuc should be lower than FLuc, right? Then with the use of 5-iodotubercidin, which induces e10 inclusion, in suppl Fig.6 there is increase of both 4R (+ex10) and 3R(-ex10) isoforms. How come? 3R should not increase…
5) Are the two splicing intein proteins equally efficient splicing out the Luc proteins (Gp41-1 and NrdJ-1)? Maybe Suppl Fig 5 was intended to study this, but I don't understand the results. Looks like for each NLuc signal there are 30 of FLuc, which makes FLuc more efficiently spliced. Was this corrected in the main figures? It is kind of important since usually we look at the relative levels of the alternatively spliced isoform vs total protein. If one intein

is more efficient than the other, it will affect interpretation of results. Also, can inteins splice out all the mRNAs translated? In a screening, can inteins be inhibited leading to indirect effects (no blasticidin not because there is no exon inclusion, but intein is inhibited or translation inhibited)?

Response to R1P4 and R1P5:

To adjust for the difference in the signal from FLuc and NLuc (due to differences in translation, half-life-time, enzyme activity, and brightness of the substrates), we expressed 0N4R-isoform from EXSISERS$_{MAPT:10NLuc-11FLuc}$ in which the two luciferases are driven at 1:1 stoichiometry by a Pgk1 promoter.

By transfecting increasing amounts of this plasmid, we established a linear relationship between the relative luminescence signals from FLuc and NLuc and determined that for our experimental settings, 30 RLUs of FLuc correspond to 1 RLU of NLuc, i.e., NLuc is 30-fold brighter than FLuc (original **Supplementary Figure 5b**, now **Supplementary Figure 2c**). As can be seen in the immunoblot (**Supplementary Fig. 2b**), this factor is not due to a difference in splice efficiency but rather a difference in substrate-dependent turnover rate and substrate/detection sensitivity.

In the new **Supplementary Fig. 5c**, we used this factor to adjust for the relative brightness and calculated the fraction of exon 10 inclusion to be ~5% in HEK293T-derived cells, in accordance with tau immunoblots (**Supplementary Fig. 5b**). The IVS10+16 c>t mutation led to a ~4-fold increase in exon 10 inclusion in the luciferase-based readout (**Supplemental Fig. 5c**), which matched the 3.7-fold increase, determined by immunoblot (**Supplemental Fig. 5b**).

Since the experiments of **Figure 3** are designed to show differential effects of pharmacological and genetic modulation of isoform expression, we have normalized all NLuc/FLuc data from EXSISERS$_{MAPT:10NLuc-11FLuc}$ to the control/baseline condition (induced *MAPT* but w/o perturbation), such that absolute differences in brightness are compensated, and differences due to the experimental perturbation can be directly read off the graphs.

We explained this normalization procedure in the figure legend, in the methods and statistics section.

Please also see our answers to R4PC2 and R4PC3.

With respect to your comment on the original Supplementary Fig. 6 (now embedded as **Supplementary Fig. 11a**), we thank the Reviewer for pointing out the inconsistency; indeed, the caption for this figure was mistakenly set. The caption was shifted by one position to the left; the legend has been corrected, and a new immunoblot has been inserted in the same as subfigure **b** with a finer titration of ITU. We are very sorry about this mistake and replaced the figure with a corrected version. Also, a similar immunoblot in direct comparison with EXSISERS$_{MAPT:10NLuc-11FLuc}$ has been inserted as new **Supplementary Fig. 12**.

R1P6:

6) Taking into consideration that the RNA is affected by using Cas13. It is important to show that the « protein » splicing effects observed with the inteins are also true at the RNA level by qRT-PCRs. e10 and total MAPT RNA levels should be affected accordingly in Fig.3. It is an important control.

Response to R1P6:

We have now performed RT-qPCRs experiments to validate all Cas13 key results of **Fig. 3** at the RNA level, *i.e.*,

a) Cas13d-NLS with an extended spacer is outperforming Cas13d-NLS with the originally published 22 nt spacer regarding general perturbation efficiency.

b) When Cas13d is applied in the nucleus using an isoform-specific spacer, it will still lead to a knock-down (KD) of all isoforms.

c) Cas13d applied on exon-junctions is more specific towards an isoform since it can only bind to the post-RNA-splicing mature mRNA.

d) shRNA is at least comparable if not superior to CRISPR/Cas13d or b, given that the latest miRNA scaffolds and the latest design rules are deployed. It also does not require the co-expression of two components (crRNA and Cas13).

R1P7:

7) In Fig.3c, why crRNA 10-11 is not affecting total MAPT levels but 9-10 is ? More puzzling, why the use of shRNAs to mimic miRNAs pathway has the opposite effect, it is the 9-10 that is more isoform specific than 10-11 ?

Response to R1P7:

We thank the Reviewer for this question regarding the details of **Fig. 3**.

In **Fig. 3c**, crRNA targeting exon 10-11 is clearly knocking-down 4R tau (NLuc) but seemingly not pan tau (FLuc). The reason is that the true fractional expression of 4R tau is very low (around 3-5%, please see R1P4 and R1P5 for details) compared to 3R tau (only very mature primary neurons in a complex 3D culture model are expressing a significant level of 4R tau (doi:10.1016/j.scr.2019.101541), thus even a 100% knock-down (KD) of 4R tau would just lead to an insignificant KD of pan tau.

The [2]9-10 crRNA is asymmetrically positioned on the 9-10 junction (=4R, **Fig. 3d**) and thus also matched almost perfectly on the 9-11 junction (3R, **Supplementary Fig. 16**) with only a single-nucleotide terminal mismatch (Cas13 systems tolerate single-nucleotide mismatches) resulting in the KD of all isoforms. For the 3rd generation shRNAs, the 9-10 microRNA (miR) was symmetrically positioned on the 9-10 junction (4R, **Fig. 3d**) and thus was specific for only 4R-tau since; an alignment of the 9-10 miR on the potential matching 9-11 junction (3R, **Supplementary Fig. 16**) showed 3 mismatches in the 5'-seed region (position 2–7) and thus was not activating the RNA-induced silencing complex (RISC) when accidentally bound to 3R.

In contrast, the 9-10 junction targeting miR was asymmetrically positioned onto the 9-10 junction (4R, **Fig. 3d**) due to design constraints of microRNAs and thus was also matching perfectly with its 5'-seed region (position 2–7) onto the 9-11 junction (3R, **Supplementary Fig. 16)** with only mismatches in its 3'-end that is tolerant towards mispairings.

Expectedly, the KD of 3R tau (crRNA targeting 9-11 junction) led to a clear decrease of pan tau signal (FLuc) without changing the 4R tau level (NLuc) in main **Fig. 3c**. This also has been confirmed in RT-qPCR in unmodified 293T cells in the new **Supplementary Fig. 14b**.
In summary, a strong depletion of pan tau (FLuc) in this cell line while trying knocking down 4R tau is clearly a side effect of lack of isoform specificity that can be observed for the exon 10 targeting crRNA and for the asymmetrical 9-10 junction targeting crRNA (^{2}9-10), while the crRNA targeting the 9-10 junction symmetrically (^{1}9-10) and the 10-11 junction are more specific.

R1P8:
8) Fig3f, dCasRX-SR effect is just 1,6x-fold. I don't think this is going to be biologically meaningful. The control in which there is dCasRx-SR or dCasRX-hnRNPA1 but not crRNA is missing (to make sure there are no indirect effects).

Response to R1P8:
The main objective of **Figure 3** is to show how EXSISERS technology can be used to optimize programmable effectors at the RNA level for modulating isoform-specific expression. We found a strong effect of the length of the guide RNA and the localization of the Cas13-effectors, while amiRNA was also very competitive.
To complete the picture, we also added data on the use of dead Cas13 systems for splicing modulation, because it is an application that is not possible with amiRNA.

We have now replicated the results on two independent clones, including the requested non-targeting controls (NTC) on another WT clone and also a clone carrying the IVS10+16 c>t mutation (**Supplementary Fig. 17**).
These results show that also small changes in isoform-specific expression can be quantified reliably with EXSISERS.
We did not express any opinion on whether the observed effects are biologically meaningful but simply suggest that EXSISERS can help to characterize and optimize systems that alter isoform-specific expression.

R1P9:

9) Again, the effect on Suppl Fig 12 seems very low too, 1,5x-fold. Is this sufficient to claim what the authors claim?

Response to R1P9:

We applied EXSISERS on a ribosomal-frameshifting-regulated gene to show EXSISERS' unique capability to monitor co-translational regulations, where RT-qPCR would fail. However, we did not claim a new finding. The observed effects are concentration-dependent and were independently confirmed with two complementary methods (fluorescence-activated cell scanning (FACS) and immunoblot analysis).

R1P10:

10) Why are the IFs in Fig2c and Supplementary Figure 11d,f so dotted at the nuclear level? Is this related to the reporter?

Response to R1P10:

Given that also unmodified HEK293T cells showed the 'nuclear dots' (new **Supplementary Fig. 5a**), they are likely a result of some unspecific binding of the pan-tau antibody (TAU-1 alias PC1C6) to nucleolar proteins in our immunofluorescence staining protocols.

R1P11:

11) For Fig.4, the CRSPR screening, it is important to know how many clones resisted to the blasticidin to know the false-positive rate of the system. The authors only show the positive MBNL1 clone, but this was already well known. Was the finding straightforward? It does not invalidate the proof-of-concept but it can give perspective on the feasibility of the system. It is known that some cells can escape the blasticidin selection. Were the authors using a higher amount of antibiotic than what is used for clone selection (1-10 ug/mL depending on the cell type)?

Response to R1P11:

We performed the experiment with a theoretical ~400-fold coverage of every sgRNA. The library contained ~80,000 sgRNAs against ~20,000 coding genes, including non-targeting control sgRNAs, resulting in 4 sgRNAs per gene. To achieve a ~400-fold coverage, we infected 100×10^6 cells with the lentiviral library with a multiplicity of infection (MOI) of ~0.3.
At least several hundred clones survived the most-stringent blasticidin selection condition (5 µg/ml). NGS analysis revealed that in this condition, 28.4% of the clones contained aN *MBNL1*-targeting lentiviral vector (composed of 18.8% and 9.6% of two different sgRNAs targeting *MBNL1*). Under low-pressure selection with the minimal inhibitory concentration of 3 µg/ml blasticidin-S, the flasks were confluent after the same timeframe. Still, based on the NGS analysis, 1.4% of the confluent population contained a lentivirus with a sgRNA targeting MBNL1. Also, based on NGS, only 0.0001% of the unselected control condition contained the same sgRNAs targeting MBNL1. This results in a 4 magnitudes of fractional enrichment in the 3 µg/ml blasticidin S condition and >5 magnitudes fractional enrichment for the more stringent 5 µg/ml blasticidin S condition. In other words, by simply subcloning the PCR product (instead of NGS) of the integrated lentiviral sgRNA expression cassette of the most stringent condition (5 µg/ml), followed by a standard Sanger sequencing of at least 20 clones, one would already expect 5-6 bacterial clones containing an *MBNL1*-targeting sgRNA. We emphasize that two independent sgRNAs targeting MBNL1 were independently enriched by 3 magnitudes (3 µg/ml blasticidin-S) and 4 magnitudes (5 µg/mL blasticidin-S) over the median sgRNA population. Importantly, we validated the screen on a different

EXSISERS$_{FOXP1:18b\text{-}BSD}$ clone using a 3rd independent sgRNA (different from the two enriched *MBNL1*-targeting sgRNAs of the library) targeting a constitutive *MBNL1* coding exon in parallel with a sgRNA targeting *MBNL2,* followed by blasticidin-S selection. Only the condition targeting *MBNL* genes led to blasticidin-S-resistant cells but targeting the control *AAVS1* locus did not. Moreover, when analyzing the surviving population via sequence decomposition of Sanger sequencing results, a dose-dependent accumulation of mutations in MBNL1 with increasing blasticidin-S concentration was indicative of functional coupling of the MBNL1-FOXP1-18b-Bsd-axis. With WT cells expectedly, we could not detect any resistant cells independently of any selection conditions and independently of the gene that was targeted. As described in **Fig. 4**, we used blasticidin-S in a concentration range the Reviewer indicated (3 µg/ml and 5 µg/ml are exactly in the range of 1-10 µg/ml).

Reviewer #2 (Remarks to the Author):

R2P0:

In this manuscript, the authors developed a new type of cell-based reporter system, exon-specific isoform expression reporter system (EXSISERS), which enables non-invasive detection of alternative splicing and exon-specific translation via intein-mediated protein splicing. They construct generated dual-luciferase (Nluc and Fluc) EXSISERS lines for ratiometric monitoring of different Tau protein isoforms, 3R-tau and 4R-tau. As designed, the system can recapitulate the expected change of different tau protein isoforms. The application of this reporter system was further demonstrated in several scenarios: 1. Screening of the effective guide RNAs in CRISPR/Cas-13 system that can achieve isoform-specific gene silencing; 2. Testing the activity of designer splicing enhancer or suppressor using the dCas-13 fusion protein containing SR domain or Gly-rich domain; 3. Measuring the co-translation ribosomal frameshift regulation. Finally, they generated an EXSISERS reporter for alternative splicing of exon 18b in FOXP1 and use the reporter to identify the regulators for isoform-specific expression of this exon via genome-wide CRISPR/Cas9 screen. Given their results the authors propose that it will be possible for an unbiased and non-invasive functional screening for splice modulators.

Overall I find the approaches employed in this study is valuable for characterizing and manipulating the intrinsic functionality of the exon-specific protein isoforms. However, the system is cumbersome to use and require a large amount of time for consecutive steps of CRISPR-cas insertion, which will limit its usefulness. In addition, some of the application did not perform as efficiently as previous system that was much simpler to generate. For example, the designer splicing enhancer and silencer using aCas-13 in EXSISERS reporter (Fig. 3f and 3g) was not as efficient as the engineered splicing factors using PUF fusion proteins (Wang Y et al, 2009 Nature Method, Wang Y et al 2013 NSMB), which is much simpler system to use. The authors should acknowledge such limitation and compare their system with previous system.

Response to R2P0:

We thank the Reviewer for acknowledging the value of EXSISERS to assess exon-specific protein isoform expression.
As we show in **Table R1**, EXSISERS has a unique set of advantages over other methods.

	EXSISERS	minigenes	immunoblot	immuno-cytochemistry	RT-qPCR	RNA-FISH
at endogenous site	☑		☑	☑	☑	☑
protein-level readout	☑		☑	☑		
cellular resolution	☑	☑		☑		☑
coupling of effectors to exon inclusion	☑	☑				
repeated measures	☑	☑				
no cell line needed			☑	☑	☑	☑

Table R1| Advantages of EXSISERS over alternative methods to detect isoform-specific expression

Although it is required to generate stable EXSISERS cell lines to ensure that isoform-specific expression is monitored at physiological levels, it is **not more cumbersome to generate those lines than it is to generate adequate minigenes**. Minigenes also have to be

integrated into the genome to not unphysiologically overload the splicing/expression machinery, which will lead to aberrant alternative splicing behavior, as reported for, *e.g.,* MAPT.
Please see a comparative analysis of two minigene systems for *MAPT* in our response to R3P2.

To ensure maximal convenience in producing EXSISERS lines, we have streamlined the process such that only a **single cloning step** is necessary to generate the all-in-one CRISPR/Cas9 plasmid and the targeting plasmid, that can be inserted into the genome within **2-3 days** (please see **Supplementary Fig. 3**, previous Supplementary Fig. 2). The CRISPR/Cas9-mediated insertion is sufficiently efficient with the plasmids we provide, such that within just **2 months**, clonal EXSISERS cell lines can be generated (**Supplementary Fig. 4**).
With respect to efficiencies using CRISPR-Cas9-mediated insertions, please see the detailed response to R1P1 for targeting efficiencies of EXSISERS.

With respect to Pumilio/PUF-based splicing modulators, we agree that they are powerful and we, therefore, had already cited Wang, Y., Cheong, C., Tanaka Hall, T. *et al*. "Engineering splicing factors with designed specificities." Nat Methods 6, 825–830 (2009), doi:10.1038/nmeth.1379 in our original submission.
Since Cas13-based splice modulators are still currently of broad interest, chose this system to show that EXSISERS can be used to optimize it, but the same is, of course, goes for Pumilio/PUF-based splicing modulators.

R2P1:

Specific concerns:
1. The intein used in this study were shown to have high splicing efficiency (Supplementary Fig. 1) in their system, however I am curious about how efficiently the intein works in different cell lines. Additional quantification should be performed to measure the intein excision rather than assuming it is always 100% excised.

Response to R2P1:

Inteins have indeed been shown to be effective upon heterologous expression in several mammalian cell types *in vitro* and *in vivo*. Most importantly, applications in mammals, such as splitting Cas9 to circumvent the limited packaging capacity of recombinant adeno-associated viruses (rAAVs), a commonly used viral vehicle for gene therapy, by harnessing trans-splicing inteins ('protein ligation' of two co-expressed polypeptides), were effective *in vivo* in pig and mouse models (doi:10.1038/s41591-019-0738-2, doi:10.1038/s41551-019-0501-5).
We have further improved the high splicing efficiency of the fast-splicing inteins (doi:10.1074/jbc.M112.372680) by adding coiled-coils (CCs) to support cooperative folding of the cis-splicing intein halves and its excision. We updated **Supplementary Fig. 1** with data for which we used mNeonGreen as extein as it is known to fold extremely rapidly in much less than 10 minutes. Thus, we reasoned that this extreme case of a fast-folding extein should be maximally sensitive to detect any unproductive folding intermediates.
Under these circumstances, the CCs-enhanced intein resulted in a higher product/educt-ratio compared to the CCs-less counterpart. C-cleavage side products could only be detected upon overexposure and contrast enhancement. We did not detect any N-cleavage products.

Upon request of the Reviewer, we have now included full immunoblots from multiple clones showing essentially no unspliced products for tau. Only under extreme overexposure, weak

bands appear at densities of less than <1% of the spliced products, which most likely correspond to the *de novo* translated proteins (**Supplementary Fig. 7**). Even for minigene-versions of EXSISERS$_{MAPT\text{:10NLuc-11FLuc}}$, which are heavily overexpressed at unphysiological levels, we could barely detect any unspliced educt (**Supplementary Fig. 10c,d**).

In addition to our experiments with HEK293T cells, we have observed similar results from murine neuroblastoma cells (N2a) in which housekeeping gene (*Tubb3*) was intact (**Supplementary Fig. 18m**). Here, too, no unspliced educts could be detected.

R2P2:

2. In Fig.2, since the study is focusing on the exon-specific isoforms of tau protein, the authors should use an exon10 specific tau antibody (or pan antibody for tau) to calibrate the system. This is to make sure that the results obtained from luciferase measurement correlate well with direct measurement of tau isoforms.

Response to R2P2:

Reliable tau-specific antibodies are hard to get by. Still, we had screened several anti-tau antibodies and found that the best way to reliably identify 4R tau is by comparing a 3R-immunoblot to pan-tau immunoblots. We proved that this band is indeed the 4R band in **Supplemental Fig. 11a).** However, the S/N-ratio of this 4R-antibody (doi:10.1186/s13024-017-0229-1) is low, and we also needed to see the fractional inclusion of 4R from total tau. Thus, the anti-pan-tau antibody was the most informative tool for our requirements.

When WT HEK293T cells were treated with ITU known to increase 4R tau (doi:10.1111/febs.12411), the 0N4R band (2nd band from below in anti-pan-tau immunoblot, **Supplementary Fig. 11**) was clearly increasing while 0N3R was decreasing (1st band from below in the anti-pan-tau immunoblot, **Supplementary Fig. 11**). Similarly, the bioluminescent signal from EXSISERS$_{MAPT\text{:10NLuc-11FLuc}}$ increased by ~4-fold (**Fig. 2f, j**) and longitudinally over a period of 60 hours in **Fig. 2h**.

In a direct comparison from unmodified HEK293T cells and EXSISERS$_{MAPT\text{:10NLuc-11FLuc}}$ in the same immunoblot, increasing ITU concentration resulted in a fractional increase of 4R tau. In contrast, the total tau level decreased slightly (new **Supplementary Fig. 12**). As expected for EXSISERS$_{MAPT\text{:10NLuc-11FLuc}}$, the OLLAS-positive band for excised NLuc (=4R) was getting more prominent with increasing ITU concentrations (**Supplementary Fig. 12**).

Furthermore, **Fig. 2d** showed that the IVS10+16 c>t mutation caused an ~3–4-fold increase of 4R-tau in both, immunoblot and in luciferase signal (**Fig. 2d,e and h, and Supplementary Fig. 5,6, and 7**). Please note that although the size separation and spatial resolution of the tau bands is high compared to typical anti-tau immunoblots in the literature (doi:10.1186/s13024-017-0229-1, doi:10.3892/ijmm.2012.1025), precise quantification of tau isoforms by densitometry is extremely challenging.

R2P3:

3. In Fig.4, I feel that this part lacks an important analysis on transcriptome level for the MBNL1/2-KO cells and the exon 18b inclusion cells after blasticidin selection. MBNL1/2 are key regulator in RNA splicing, and knock-out of these two genes should cause significant change of splicing in the level of entire transcriptome. I am wondering whether knock-out of these two genes could cause more exon-specific protein changes besides FOXP1.

Response to R2P3:

We agree with the Reviewer that it is interesting to ask which impact perturbations of MBNL proteins have on the transcriptome.

In our manuscript, however, it was the goal to present EXSISERS as a screening tool for unbiased identification of splicing modulators. Indeed, without any prior knowledge, we re-identified MBNL1 as the main regulator of *FOXP1* exon 18b inclusion using an unbiased lentiviral CRISPR/Cas9 screen, which was impossible before. We then followed up with a knockout of independent sgRNA targeting *MBNL1* to validate the results in our system.

With respect to the effects of MBNL on the transcriptome, we would like to refer to the thorough work of Han *et al.*, 2013 (doi:10.1038/nature12270), where they use RNA-seq profiling to analyze the impact of MBNL perturbations mediated by siRNAs. They showed that MBNL proteins negatively influence the global AS network important for pluripotency maintenance, partially by repressing the ES-cell-specific FOXP1 isoform, a stimulator of a core pluripotency circuit, thus promoting transcriptome-wide switch towards differentiation.

R2P4:

4. I think this paper may present a powerful tool to track and study exon-specific protein isoform. However, the authors should use it to investigate on new biological questions rather than only to confirm the conclusion people have already made.

Response to R2P4:

We thank the Reviewer for sharing enthusiasm towards EXSISERS as a 'powerful tool' to investigate alternatively spliced protein isoforms. While the main weight of such a methodological paper must clearly lie on the careful validation of the new instrument on the various technical levels against well-established results, we have made a few interesting observations showing the robustness and convenience of EXSISERS technology:

We showed for the first time,

a) the longitudinal readout of isoform-specific expression with cellular resolution of an alternatively spliced exon from the original genomic site in living cells,
b) an improved targeting efficiency of Cas13d significantly by the extension of the spacer length from 22 nt to 30 nt,
c) the importance to optimize the precise site of action for each programmable intervention tool (Cas13d or b, or shRNA in the cytosol) since it has a massive impact on the isoform specificity, even if the same position is targeted,
d) that shRNA - if carefully designed using the latest design rules and using up-to-date pri-microRNA biogenesis-mimicking scaffolds - can compete with Cas13-based systems regarding potency and isoform-specificity,
e) an independent confirmation of a serendipitous scientific finding of *FOXP1* exon 18b regulation via MBNL1 using a novel unbiased approach.

These examples lay out precise recipes for biological discoveries and there are already several laboratories in our network that are actively using EXSISERS technology to test their preferred biological hypothesis.

R2P5 (Minor P1):

Minor concern:

Overall the figures are poorly prepared with low resolution and confusing color scheme, more specifically:

1. The picture quality of Fig.2c and Fig.2g should be improved. The color and style of this figure should be modified to make it more reader friendly. In addition, Fig.2c and 2g should be showed in color to help understand.

Response to R2P5 (Minor P1):

We apologize that the quality of our figures was apparently compromised during compression. We are sorry for the compression artifacts of **Fig. 2** that occurred in the last submission. All our original figures are high quality.

R2P6 (Minor P2):

2. The picture quality of Fig.4c and Fig.4d should be improved. And the part (Identification of regulators for isoform-specific expression) and Fig.4 need be carefully reviewed, because the figure and the main text are not consistent.

Response to R2P6 (Minor P2):

We are sorry for the compression artifacts of **Fig. 4** that we improved. Furthermore, we thank the Reviewer for pointing out the disparity between main text and **Fig. 4**, we carefully re-read the main text and corrected inconsistencies with the figure.

R2P7 (Minor P3):

3. Supplementary Fig.8b need to be updated, as the resolution is very low.

Response to R2P7 (Minor P3):

We are sorry for the low quality of the original **Supplementary Fig. 8** (now improved in **Supplementary Fig. 19**). Regarding subfigure **b**, the GFP channel **did not show any signal** since in contrast to luciferases, endogenous expression of 4R tau did not yield enough protein to be readily detected in a common epi-fluorescence microscope.

R2P8 (Minor P4):

4. Similar to Fig. 2c, the supplementary Fig.11 and Fig.13 should be improved.

Response to R2P8 (Minor P4):

We improved the quality of the respective figures.

Reviewer #3 (Remarks to the Author):

R3P1:

Truong et al. develop a minimally invasive isoform-specific expression reporter system (EXSISERS) that incorporates translated and subsequently excised fast-splicing inteins with CC-domains into genes of interest. The authors demonstrate the utility of EXSISERS in a number of applications, ranging from the optimization of RNA-targeting strategies for exon-specific RNA degradation of MAPT mRNA, to the quantification of ribosomal frameshift-mediated regulations unmeasurable by RT-qPCR, to a phenotypic readout for a high-throughput screen of FOXP1 exon 18b inclusion that validates existing literature. Altogether, the presented work is a valuable addition to the isoform-specific RNA monitoring toolkit. While the generation of EXSISERS may be an involved process, nevertheless for some applications it might prove more useful than alternative methodologies, such as minigenes. I have a few major criticisms.

Response to R3P1

We thank the Reviewer for acknowledging the value of EXSISERS for monitoring isoform-specific expression. We have compiled **Table R1**, to compare the features of EXSISERS as compared with other relevant methods for detecting isoform-specific expression.

	EXSISERS	minigenes	immunoblot	immuno-cytochemistry	RT-qPCR	RNA-FISH
at endogenous site	☑		☑	☑	☑	☑
protein-level readout	☑		☑	☑		
cellular resolution	☑	☑		☑		☑
coupling of effectors to exon inclusion	☑	☑				
repeated measures	☑	☑				
no cell line needed			☑	☑	☑	☑

Table R1 | Advantages of EXSISERS over other methods to detect isoform-specific expression

Although many important findings were made possible by minigenes, they may (**1**) suffer from untruthful readout, (**2**) cause alterations of endogenous splicing, while (**3**) still requiring the same effort on cloning and generation of stable cell lines.

(1) Minigenes may lead to <u>untruthful readout of endogenous splice-regulation of a gene of interest</u> because they - with a high probability - do not contain all relevant regulatory elements. This is especially true for tau, where it has been shown that basically the whole intronic region is required to reflect the true splicing behavior for exon 10 (doi:10.1111/j.1471-4159.2004.02477.x). Most importantly, it has been shown recently that many identified SNPs have their origin deeply embedded within introns, such as the rs242561 polymorphism, that is protective against Parkinsonian disorders (doi:10.1016/j.celrep.2016.03.068). This single nucleotide polymorphism is located within the first intron 13.2 kbp upstream of the 2nd coding exon and 55 kbp downstream of the first coding exon; the same is true for rs242557 which is also associated Parkinson's disease, which is located 48 and 20 kbp down- and upstream from the flanking exons (doi:10.1016/j.neulet.2010.10.015, doi:10.18632/oncotarget.16490) or rs2471738 that lies

11.6 kbp upstream of the alternatively spliced exon 10 and 2 kbp downstream of exon 9 (doi:10.18632/oncotarget.16490). Moreover, many vertebrate genes are recursively spliced which will not be recapitulated by minigenes (doi:10.1038/nature14466). Also, for other alternatively spliced genes such as CD44, the intron's length determines the inclusion efficiency of the alternatively spliced exon (doi:10.1128/mcb.18.10.5930). A mini-gene version that contains truncated introns would therefore inevitably lead to unphysiological splicing. Thus, it is essentially impossible to faithfully recapitulate the complex regulatory machinery outside the precise three-dimensional context of the endogenous sites.

(2) Minigenes are not applicable to unbiased screens for splice regulators (such as genome-wide CRISPR/Cas9 KO-screens) to enrich a certain population of cells with a defined genetic perturbation. Minigenes are normally used in a transient transfection assay and even if integrated into the genome, they lay outside of the endogenous site and are driven by constitutive promoters. They are, therefore, hiding effects of (co)-transcriptional regulations. Also, the truncated introns cannot reflect the physiological genomic context such that whole-genome screens would probably yield questionable results.

(3) Minigenes can cause alterations of endogenous splicing of other collateral genes by competitive binding of splicing factors to the constitutively overexpressed minigene. This results in depletion from endogenous sites. In the case of *MAPT*, the altered isoform ratios can even feed-back on the splicing process since the formation of aggregated neurofibrillary tangles leads to the co-depletion of the otherwise soluble spliceosomal components further increasing the aberrant change of the global cellular splicing pattern (doi:10.1016/j.celrep.2019.08.104).

(4) Minigenes require the same effort to establish as EXSISERS

We made sure that the production of the EXSISERS lines is as convenient as possible: we provide all EXSISERS reporters in a respective cloning vector, such that only a single cloning step is required to obtain a customized exon-specific EXSISERS vector (please see **Supplementary Fig. 3**). The CRISPR/Cas9 vector, improved with enhanced gene targeting efficiency, can also be cloned in a **single** step (please see **Supplementary Fig. 3**). Please also see our graphical abstract of the process (**Supplementary Fig. 4**), which shows hot an EXSISERS clonal cell line can be established in just ~4–6 weeks. Please also see our response **R1P1**.

With respect to the effort for making the respective cell lines, minigenes also require the assembly of different fragments of truncated exon-intron fragments and subsequent cloning into a mammalian expression vector. Usually, several minigene versions with different truncations need to be tested, since truncations can lead to the removal of essential regulatory sequences, which are important for the regulation of alternative splicing.

Furthermore, minigene systems that are not read out via RT-qPCR but via a reporter system - which is essential for high-throughput detection - require additional modifications in the alternatively spliced exons to include stop or start codons for fluorescent proteins or luciferases. Alternatively, a frameshift-based reporter to distinguish the ab- or presence of an exon can be used. This, however, requires also a deletion/insertion of 1 or 2 nucleotides, since normally an alternatively spliced exon contains a number of nucleotides divisible by 3 (Stoilov *et al.* (doi:10.1073/pnas.0801661105), Luo *et al.* (doi:10.1002/cbic.201402069)).

Also, random integration of the minigene into the genome introduces an unnecessary variability due to copy number variation, impact on neighboring genes, expression strength,

and splicing behavior (doi:10.1016/j.cell.2010.11.056). Additionally, screening compound libraries to alternate AS, library-scale minigene transfection for every condition would not be economically feasible.
In summary, also for minigenes it is recommended to knock-in into a well-defined safe-harbor locus (such as *AAVS1/PPP1R12C* in human and *Rosa26* locus in murine systems) using CRISPR/Cas9 (or TALENs, ZFNs) to minimize variability.

Please also see our detailed response to your request in R3P2 where we also carefully compared minigenes with EXSISERS.

R3P2:

Major points:

1. The authors do not perform any head-to-head comparisons of EXSISERS to minigenes, which are comparatively much simpler and faster to generate. This should be done. If there is no clear advantage of EXSISERS, then it is worth wondering whether other researchers will adopt the new methodology.

Response to R3P2

Thank you also for the constructive suggestion to perform a head-to-head comparison with minigenes.
To this end, we have carefully studied the elaborate minigene systems for *MAPT* by Yu *et al.*, (doi:10.1111/j.1471-4159.2004.02477.x) and Jiang *et al.* (10.1128/mcb.20.11.4036-4048.2000 to construct corresponding minigene systems.

Before we compare our results shown in **Supplementary Fig. 10**, we need to quickly review the pertinent findings from Yu *et al.*, which is a very careful study that, however, also demonstrates the complexity and potential pitfalls for obtaining truthful results with minigenes.
It can be seen from **Figures 1 and 2 in Yu *et al.*** (attached below with figure legend) that the authors laboriously tried out 10 different tau-4R minigenes with different intronic truncations but found that none of them showed physiological splicing behavior. Only a plasmid made from a construct with **full-length** introns of 17,485 bp (LI9/LI10) recapitulates the endogenous physiological ratio. Similar behavior for minigenes also could be observed by Jiang *et al.* (Fig. 2B vs. Fig. 2A, doi:10.1128/mcb.20.11.4036-4048.2000). Besides, using full-length introns in minigenes is technically very difficult, since those introns can easily reach 5-digit bp in length and thus require specialized PCR-protocols to be amplified. Equipped with a plasmid backbone of ~3 kbp, promoter elements, and the rest of the tau coding sequence, this plasmid would also easily exceed the 20 kbp limit for classic plasmid transfection (doi:10.1093/nar/27.19.3792, doi:10.1016/j.ab.2005.08.029). Also, for plasmids greater than 20 kbp, the increased risks of plasmid instabilities enforce the usage of bacterial artificial chromosomes (BAC) instead.
Aberrant splice behavior of minigene systems has also been reported for other genes than *MAPT*. For the *ABCA4* gene (128 kbp, 50 exons), which plays a role in the Stargardt disease, Sangermano *et al.* (doi:10.1101/gr.226621.117) *'[...] discovered that when using small minigenes lacking the proper genomic context, in vitro results do not correlate with splice defects observed in patient cells.'* They *'[...] therefore devised a novel strategy in which a bacterial artificial chromosome was employed to generate midigenes, splice vectors of varying lengths (up to 11.7 kb)* ***covering almost the entire ABCA4 gene.'*** Only under these circumstances, a similar splicing behavior as observed in patients could be recapitulated.

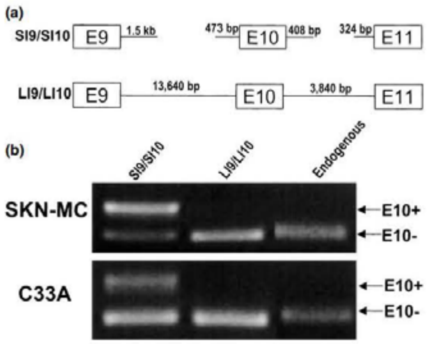

Fig. 1 Introns 9 and 10 affect splicing patterns of exon 10 in the *tau* gene. (a) Mini-gene constructs for splicing of exon 10 in the *tau* gene were generated in PCI-neo vector. The short previously published mini-gene SI9/SI10 includes exon 9, the first 1.5 kb and the last 473 bp of intron 9, exon 10, the first 408 bp and the last 324 bp of intron 10, and exon 11. The long mini-gene construct LI9/LI10 contains full length of both intron 9 and intron 10. (b) Mini-gene constructs were transfected into C33a or SKN-MC cells. Splicing patterns of exon 10 in mini-genes were examined by using RT–PCR. Splicing of exon 10 from the endogenous *tau* gene was detected in C33a cells or SKN-MC cells induced by 10 μM of sodium butyrate for 24 h.

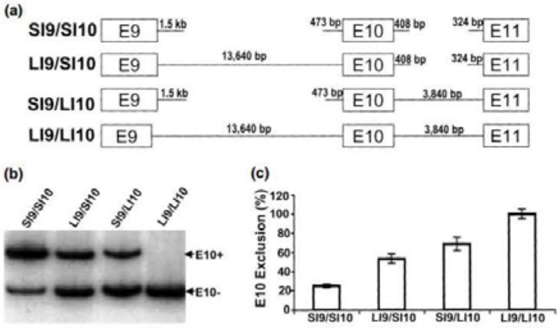

Fig. 2 Intron 9 and intron 10 additively contribute to correct splicing of exon 10 in the *tau* gene. (a) Constructs with a full-length intron 9 and a short intron 10 (LI9/SI10) or with a full-length intron 10 and a short intron 9 (SI9/LI10) were generated. The short intron 9 or short intron 10 was identical to that in SI9/SI10. (b) The constructs were transfected into SKN-MC cells. RT–PCR was used to determine splicing patterns of exon 10. (c) RT–PCR bands were quantitated using a phosphorImager. Bar represents the mean percentage of mRNA with exon 10 exclusion (E10 –) out of total mRNAs (E10 + and E10 –) from three separate transfection experiments. Error bars represent standard deviations of the means.

These results suggest that intronic truncations, an essential characteristic of minigenes, can be misleading, even if the minigene contained several hundred nucleotides of sequences down- and upstream of an exon of interest. Zheng *et al.* (doi:10.1101/gr.147546.112) also warned that *'[...] minigene reporters do not always recapitulate the regulation of endogenous exons. The minigene may not contain all of the relevant cis-regulatory elements for the test exon.'*
Recent reports (doi:10.1038/nature14466) also suggested that vertebrate introns, especially long ones, are often removed stepwise in a process called 'recursive splicing'. Thus, a minigene with truncated introns would inevitably lead to an altered RNA splicing behavior. Especially vertebrate introns can be larger than 100 kbp and can hardly be cloned fully in a minigene. Most importantly, those long introns are not just 'junk', which can be replaced by random nucleotide sequences.

For example, Wang *et al*. showed recently that the rs242561 polymorphism is protective against Parkinsonian disorders (doi:10.1016/j.celrep.2016.03.068). This single nucleotide polymorphism is located within the first intron 13.2 kbp upstream of the 2nd coding exon and 55 kbp downstream of the first coding exon; the same is valid for rs242557, which is also associated Parkinson's disease, which is located 48 and 20 kbp down- and upstream from the flanking exons (doi:10.1016/j.neulet.2010.10.015).
A stably integrated minigene is also preferred over transiently transfected plasmids, as Jiang *et al*. (doi:10.1128/mcb.20.11.4036-4048.2000) noted regarding the tau minigenes. They note '*[...] that transfected tau minigenes in these cells produced a slightly higher level of Tau4R compared to the endogenous tau expression pattern (Fig. 2), suggesting that overexpression of the tau minigene may titrate certain limiting factors controlling the ratio of Tau3R to Tau4R*'. Stoilov *et al*. (doi:10.1073/pnas.0801661105) also suggested that minigenes should be stably integrated: '*Note that transient expression of the reporters can lead to significant cell-to-cell variation in the protein signals, which we attribute to differences in the stability of the two proteins and in the amount of DNA taken up by each cell. This* ***variability is reduced in stable cell lines expressing the reporter*** *and with reporters where the stability of the two proteins is equalized*'.
Thus, the minigene systems are not easier to create, especially not as a version compatible with high-throughput screenings (*e.g.*, using terminally fused luciferases), which necessitates additional mutations have to be introduced into the coding sequence of the exon of interest.

Based on the luciferase minigene system described by Yu *et al*. (doi:10.1111/j.1471-4159.2004.02477.x), we build a minigene by amplifying the corresponding intronic regions with truncation that are of similar length as in Yu *et al*., and Jiang *et. al*. (doi:10.1128/mcb.20.11.4036-4048.2000), to create EXSISERS-based 4R-minigenes (**Supplementary Fig. 10a**).
In accordance with Yu *et al*. and Jiang *et al*., we noted an increased exon 10 inclusion level (~12%, **Supplemental Fig. 10c**) originating from minigenes as compared to the endogenous locus (~3–5%, **Supplemental Fig. 5b**).
For the mutation IVS10+16 c>t, 4R/pan-tau ratio further increased by roughly 2-fold to over 50%. In contrast, with integrated EXSISERS, we did not detect any significant difference between unmodified HEK293T cells, its clones, and EXSISERS$_{\mathit{MAPT}\text{:10NLuc-11FLuc}}$ (**Fig. 2d, Supplementary 5, 6, 7, 8, 9, and 12**).
The reaction of EXSISERS$_{\mathit{MAPT}\text{:10NLuc-11FLuc}}$ in response to small molecule perturbation (**Fig. 2f, h, j, and Supplementary 11 and 12**), and Cas13/microRNA-based modulation (**Fig. 3** *vs.* **Supplementary 14**) was similar to the reaction of unmodified HEK293T cells. Also, the disease-mimicking mutation IVS10+16 c>t lead to the expected 4-fold increase as reported in the literature (doi: 10.1074/jbc.274.21.15134, doi:10.1016/j.molbrainres.2005.02.014).

In summary, the head-to-head comparison of a minigene system and the EXISERS for *MAPT* showed clearly aberrant splicing behavior for the minigene but not EXSISERS as compared to unmodified cells. These findings are in line with several pieces of pertinent literature reviewed above.

R3P3:

2. The authors use CRISPR-Cas9 to integrate EXSISERS into areas of interest in the genome. When such knock-ins are performed and analyzed, typically researchers will generate multiple clonal cell lines, in case behavior in one cell line may be biased by unique Cas9-induced indel and/or template insertion off-target events. The authors should re-perform the experiments featured in Figures 3 and 4 (and associated supplemental figures) with at least one additional clonal cell line to demonstrate the generalizability of EXSISERS.

Response to R3P3:

We thank the Reviewer for this constructive criticism and agree that clonal lines may show different behavior in particular if SNPs, such as the *MAPT* IVS10+16 c>t mutation, are investigated. We have therefore included immunoblots to show that in all cases, homozygous c>t base transition in this regulatory intronic sequence led to an increase of the 4R/pan-tau inclusion-ratio in additional 9 clones (**Supplementary Fig. 6,7** in addition to the clonal line shown in **Fig. 2d**).

With respect to the experiments of **Fig. 3**, we validated the results regarding Cas13- or microRNA-mediated tau perturbation on unmodified HEK293T cells to exclude that the observed effects are artifacts on the post-translational level or by EXSISERS and performed an RNA-level quantification with RT-qPCR.
Using RT-qPCR, we confirmed that the extended 30 nt spacers are superior compared to the original 22 nt spacer in new **Supplementary Fig. 14a,** and the higher isoform specificity of targeting exon-junctions in new **Supplementary Fig. 14b**.

We also reproduced the minor effects of **Fig. 3f** in two independent $\text{EXSISERS}_{\text{MAPT:10NLuc-11FLuc}}$ clones (new **Supplementary Fig. 17**). In both clones, the combination of an exon 10 targeting crRNA together with a fusion of dRfxCas13d to the SR-rich domain of SC35 led to an increased 4R/pan-tau ratio. In contrast, the fusion to the Gly-rich domain of $\text{hnRNPA1}_{\text{A1B}}$ with a splice donor (SD) targeting crRNA decreased it (new **Supplementary Fig. 17**).

With respect to the experiments of **Fig. 4**, the results were already obtained from different clones. The lentiviral CRISPR/Cas9 KO library (**Fig. 4a and b**), as compared to the analyses in **Fig. 4c–f,** where an independent clone was used. We made this explicit into the caption of **Fig. 4.**

R3P4 (Minor P1):

Minor points:

1. The introduction would benefit from a reference to work on minigenes, as they are the main methodological competitor to EXSISERS.

Response to R3P4 (Minor P1):

We thank the Reviewer for this comment. We had already added references on minigenes in the main text in the introduction: *'Established methods for analyzing splicing isoforms either measure mRNA by endpoint-labeling (RT-qPCR, (sm)FISH[4], RNA-sequencing[5]), protein by immunochemistry (immunoblot analysis, immunofluorescence staining), or seek to mimic the genetic regulations via minigene analysis[6–8]'*

R3P5 (Minor P2):

2. The sentence should read "greater reduction": Expression of cytosolic PspCas13b-NES directed against the same region of exon 10 (Fig. 3e, orange bar) resulted in a greater reduction of FLuc as compared with the corresponding RfxCas13d-NLS (p<0.0001, post-hoc tests of one-way ANOVA) with comparable NLuc signal (p>0.05) (Fig. 3e, blue bar).

Response to R3P5 (Minor P2):

We thank the Reviewer for the suggestion, but indeed the knock-down (KD) of FLuc is 'less efficient' (leading to a 'weaker reduction' of FLuc) while NLuc depletion is as efficient as with Cas13d-NLS. We changed the whole sentence to: 'Expression of cytosolic PspCas13b-NES directed against the same region of exon 10 (**Fig. 3e, orange bar**) showed a better 4R-specificity due to decreased NLuc/FLuc-ratio compared with the corresponding RfxCas13d-NLS system (p<0.001, post-hoc tests of one-way ANOVA of $10/13d_{NLS}$ *vs.* $10/13b_{NES}$ *vs.* 9-10 amiRNA, **Fig. 3e, blue bar**).'

R3P6 (Minor P3):

3. The sentence should read "4f": Meanwhile, the enrichment of MBNL2 indels showed no dose-dependence (Fig. 4f).

Response to R3P6 (Minor P3):

We apologize for this mistake and corrected it.

Reviewer #4 (Remarks to the Author):

R4PA_B:

A. This work elegantly solves the current issues in quantifying protein expression levels by RNA-based approaches by incorporating a newly developed reporter system termed an exon-specific isoform expression reporter system (EXSISERS). The authors incorporated two EXSISERS into exons of interest (EOIs) by CRISPR/Cas9 and monitored the alternative splicing involved disease-associated exon inclusion of the patient-driven iPSC cells and screened RNA interference sequence for the isoform-specific expression to identify splice-regulators. Additionally, the authors similarly developed a survival reporter system for isoform-specific Blasticidin-S resistance marker. This article proposes the new exon-specific isoform expression reporter system would be a new tool for monitoring spatiotemporal exon-specific expression by imaging techniques.

B. This work is highly original and innovative with potential impacts in identifying splicing regulators and drug screening. Notably, this method could address the problems associated with protein expression level determined by RNA-based quantification methods. Thus, it is of significant importance and could be a game-changer for current RNA-based approaches if it is robust and reliable.

Response to R4PA_B:

We thank the Reviewer for acknowledging the advantages of EXSISERS' protein-level readout for drug screenings and basic research on identifying splicing regulators.

R4PC1:

C. In this system, there are several critical assumptions have not been controlled in this manuscript, which should be addressed in the manuscript before publications.

1. The manuscript is described as if protein trans-splicing has 100% efficiency (like Fig 2a, 2b). The splicing efficiency by protein trans-splicing is strongly affected by the junction sequence and the foreign exteins used. A single mutation near the junctions could abolish or deceased the splicing activity significantly, missing the controls to check the protein splicing efficiency.

Response to R4PC1:

We thank the Reviewer for this point regarding the efficiency of intein splicing.

In order to maximize efficiency, we chose fast-splicing inteins (doi:10.1038/s41467-020-15272-2, doi:10.1002/1873-3468.13909), which we further substantially enhanced with heterodimerization domains based on coiled-coils (CCs) (**Supplementary Fig. 1**). Recently, Bhagawati *et al.* (doi:10.1073/pnas.1909825116 showed in a similar approach, that intein splicing can be dramatically improved using a nanobody-antigen pair. By fusing an eGFP moiety to one half of a split-intein pair and an anti-GFP nanobody to the other split-intein counterpart, they could enable trans-splicing of a cysteine-free intein pair (important for extracellular protein splicing) that did not occur at all without the eGFP-nanobody interaction (please see their supplementary files Figure S10 vs. Figure S11).

These features enabled the very high splicing efficiency by immunoblot analysis of EXSISERS$_{\mathit{MAPT}\text{:10NLuc-11FLuc}}$ (**Supplementary Fig. 7**). Even when this construct was massively overexpressed via plasmid transfection, barely any unspliced proteins were detected (**Supplementary Fig. 2b**). Furthermore, the minigene version of this EXSISERS construct showed the same efficiency (**Supplementary Fig. 10c,d**).

As you requested in R4PF, the introduction of the terminal Asn→Ala mutation in the C-intein moiety completely disrupted protein splicing as expected (**Supplementary Fig. 2b**), thus indicating that the CCs-enhanced versions of the selected inteins are responsible for the exceptional high splicing efficiency.

With respect to considerations regarding the junction sequence, recent characterizations (doi:10.1002/1873-3468.13909) indicated that these 'ultrafast inteins' identified in metagenomic sources tolerate a broad spectrum of amino acids in heterologous settings very well (only proline is not tolerated heterologously and should be avoided). In conjunction with CCs, these efficiencies should increase even more.
In addition, we also now refer to the intein database in the method section under 'Application notes', which contains over 1000 inteins with known native extein sequences (maintained by the Iwaï lab, (InBase 2.0) https://inteins.biocenter.helsinki.fi/index.php), such that one can search for inteins with a desired native extein sequence to maximize the splicing efficiency.

R4PC2 and R4PC3:

2. Another assumption is similar to the previous one, FLuc and NLuc inserted in inteins fold into active equally with the same efficiency, yet having the same degradation rate in cells. The authors need to provide such experimental controls.
3. NLuc has 13-236 fold brighter than Fluc, according to the literature. All the data reported by normalized with the assumption, I believe.

Response to R4PC2 and R4PC3:

These assumptions do not have to be made. Instead, we measured the relative bioluminescence signal from *FLuc* and *NLuc* driven by a constitutive Pgk1 promoter at a 1:1 stoichiometry (**Supplementary Fig. 2a**). As seen in **Supplementary Fig. 2b**, the excision of NLuc/FLuc was very efficient. Moreover, we observed a linear relationship between the relative luminescence signals over 6 magnitudes and calculated NLuc yields 30 times more signal than FLuc (**Supplementary Fig. 2c**).

The Reviewer is also correct that for screening for modifiers of isoform expression, the bioluminescent signals were normalized to the control condition such that all relative differences between NLuc and FLuc are taken into account, and the effects of the perturbations can be directly seen. We have added additional notes in the figure legends and the manuscript to make the normalization procedure more explicit.

R4PC4:

4. The main caveat of this system easily overlooked by non-experts is the assumption that protein splicing by two split inteins has 100% or close to 100% efficiency. Particularly such high splicing activity for two orthogonal inteins has not been achieved in the past with an artificial system to my best knowledge. The reported efficiency of 95% in the cited ref.17 would result in the 90% efficiency for two orthogonal inteins. This assumption could determine the outcome of the analysis based on NLuc/FLuc quantification drastically.

Response to R4PC4:

As reported in subpoint R3PC1, we have used coiled-coil-enhanced fast-splicing inteins, and thus it is expected to have a greater efficiency than the reported value in the literature. We showed in **Supplementary Fig. 1** that CCs increased the protein splicing by nearly one magnitude (8.6-fold), which is exceptional considering the folding speed of the challenging surrogate extein mNeonGreen with less than 10 minutes (doi:10.1038/nmeth.2413). We have also conducted detailed immunoblot analysis of the dual-luciferase $EXSISERS_{MAPT:10NLuc-11FLuc}$

upon plasmid-based overexpression (new **Supplementary Fig. 2b**), when genomically integrated (new **Supplementary Fig. 5b**, new **Supplementary Figure 6 and 7**), and as overexpressed minigene variant (new **Supplementary Fig. 10c,d and** new **Supplementary Fig. 12**), and detected no relevant levels of unspliced products even not upon heavy overexpression and overexposure.

R4PD:

D. NLuc usually has 13-236 >times brighter than FLuc according to the literature, which is consistent with the data presented with Figure 2e. The NLuc/Fluc error bars cannot be smaller than each of them. However, Figure 2j and all other data presented in Figure 3 do not make any sense, statistically.
The error estimation (P-value analysis) needs to be reconsidered. There are two types of errors mixed: (1) Errors from the detection (readout values) and (2) errors from individual samples or measurements. Even when the calculated error estimated from 3 samples is small, the accuracy of the measurement cannot be better than the precision of the detection errors.

Response to R4PD:

NLuc is indeed ~30-fold brighter compared to FLuc in the dual-luciferase EXSISERS. Please see **Supplementary Fig. 2c** for the calibration we performed to adjust for the relative differences in the bioluminescent signal obtained from the two luciferases when expressed at 1:1 stoichiometry. We adjusted for those relative differences in brightness in **Fig. 2 and 3** by normalizing the relative luminescence units (RLU) to the reference condition (with *MAPT* induction but without perturbation), such that the relevant effects of the perturbation of exon-specific isoform expression can be more readily read from the figures. This procedure is described in the Figure legends, the Material and Methods section, and the Statistics section.

Concerning the error calculation, the purpose of the dual-luciferase EXSISERS is to extract a robust, ratiometric measure of isoform-specific expression (NLuc) corrected for overall gene expression of tau (FLuc). The range of isoform-specific expression is thus naturally dependent on the overall expression. The FLuc and NLuc signals are also experimentally dependent on the cell lysis step in the Promega detection workflow that we employed (https://www.promega.de/-/media/files/resources/protocols/technical-manuals/101/nanoglo-dual-luciferase-reporter-assay-protocol.pdf): FLuc substrate is provided together with a lysis buffer onto the cells, followed by the first measurement (FLuc); in the 2nd step, NLuc substrate is provided together with a FLuc inhibitor, followed by the 2nd measurement (NLuc). Thus, for every FLuc RLU data point, there is a matching NLuc data point (paired measurement).

To reduce the biological variability from pan-tau expression and experimental variability stemming from the lysis and detection procedure, it thus makes sense to take the NLuc/FLuc ratio from each sample's cell population and calculate the average and errors over cell populations.
Calculating the errors of NLuc and FLuc separately over the biological triplicates would instead discard the information that the NLuc/FLuc pair was obtained from the same sample and thus defeat the purpose of absorbing the main source of variability.
Although the main conclusions are supported by statistical analyses directly on the NLuc/FLuc ratios, we still find it informative to also display the FLuc and NLuc signals separately, to, *e.g.*, show the effects of tau induction for reference or show the effects of an extended crRNA spacer on pan-tau expression.

We have explained this aspect of data processing in the figure legend and the Statistics section.
For completion, we also show all individual data point on top of the bar graph and provide a comprehensive table showing all raw data and detailed statistical results (**Supplementary Table 1**).

R4PE:
E.
As suggested in section C, D, and F, the validity of this system needs to be validated by additional controls. The authors should describe what would be potential pitfalls by the use of this reporter system. The current presentation does not provide sufficiently clear data to judge the validity and reliability of the system.

Response to R4PE:
We thank the Reviewer for the constructive suggestions of more data from control experiments to validate the experimental findings of the manuscript. We added RT-qPCR data (**Supplementary Fig. 14**) to confirm the key messages of **Fig. 3**. Furthermore, we added controls that the excision mechanism is indeed dependent on CCs-enhanced inteins by mutating the essential Asn of the C-inteins (C-gp41-1_{N37A} and C-NrdJ-1_{N40A}) (**Supplementary Fig. 2a,b**).
As requested, we have added paragraphs to the Materials and Methods section regarding the design criteria and potential pitfalls of EXSISERS constructs, the validation experiments to confirm efficient splicing of a given construct in analogy to our **Supplementary Figures 2,5,6,7, and 12**), a direct comparison to a minigene variant (**Supplementary Figure 10**), and detailed descriptions of how to generate clonal EXSISERS cell lines complementing **Supplementary Figures 3 and 4**.

R4PF:
F.
• There is no estimation of protein splicing efficiency for none of their protein splicing constructs except for mNG shown in Supplemental Fig. 1 by immunoblot. This data also does not give any estimate of the fully spliced vs by-products (non-spliced, N- and C-cleaved products). The supplemental Fig. 1 should be supplemented by immunoblotting and/or CBB-stained SDS-gels using, for example, anti-Ollas and Flag antibodies. The quantification by Nluc/Fluc ration will be strongly affected by the ligation efficiency, which is strongly dependent on the foreign extein and the splicing junctions.

Response to R4PF-part1
As requested, we updated **Supplementary Fig. 1.**, where we also now show an additional overexposed and contrast-enhanced image to detect all potential relevant side products. We also added full immunoblots in the new **Supplementary Fig. 7, Supplementary Fig. 10c,d and Supplementary Fig. 12**.

Regarding **Supplemental Fig. 1**, we deliberately chose mNeonGreen as a model Extein with extremely fast folding rates (<<10 minutes, doi:10.1038/nmeth.2413) to define a maximally high benchmark for the intein-splicing speed. We have now added a densitometric quantification of the immunoblot in **Supplemental Fig. 1**, which shows that the addition of coiled-coils as heterodimerization domains improves the product/educt ratio by ~9 fold.
We have also added a deliberately overexposed immunoblot on which a small amount of side-products from C-cleavage can be detected that, however, amount to only ~3%.

In comparison to this test system, we have conducted detailed analyses of the protein splicing in the dual-luciferase reporter system for exon 10 inclusion of MAPT (EXSISERS$_{MAPT:10NLuc-11FLuc}$). Full immunoblots from multiple clones show essentially no unspliced products for tau **(Supplementary Fig. 7)**. Only under extreme overexposure, weak bands appear at densities of less than <1% of the spliced products, which probably correspond to the de novo translated proteins.
Even when the dual-luciferase reporter construct was heavily overexpressed at unphysiological levels from a plasmid (**Supplementary Figure 2b**) or as a minigene-version (**Supplementary Fig. 10d**), we could barely detect any unspliced educt.

• What is the correlation between the quantification by immunoblotting (and/or mRNA quantification) vs NLuc/FLuc ratio for different constructs? Does it correlate well? if not, do they have a similar trend, which could be explained to some extent?

Response to R4PF-part2

We performed additional experiments for the key messages of **Fig. 3** in HEK293T cells and quantified them via RT-qPCR. The observed effects and quantities were comparable between luciferase-based readout of EXSISERS$_{MAPT:10NLuc-11FLuc}$ cells and RT-qPCR of unmodified HEK293T cells (see **Supplementary 15** *vs.* **Fig. 3**).
Densitometric analysis of **Fig. 2d** also correlated well with the luciferase-based readouts (see new **Supplementary Fig. 5b** *vs.* **Fig. 2e**).

• See also section D on the statistical data analysis.

Response to R4PF-part3

Please see R4PD regarding the statistical analysis.

• Fig.2d needs controls for protein-splicing deficient constructs by Ser-to-Ala and/or Asn-to-Ala.

Response to R4PF-part4

We added **Supplementary Fig. 2**, where we expressed the cloned 0N4R cDNA of EXSISERS$_{MAPT:10NLuc-11FLuc}$ with intein-inactivating mutations in the C-intein moiety. The results show that active inteins are indispensable for the generation of the desired unmodified WT 0N4R tau band.

• The authors claim "bio-orthogonal pair" of two inteins, but there is no such experimental evidence provided, including cited ref. 17. Trans-splicing is strongly dependent on the exteins, the authors could provide such data as a control, as this will affect the interpretation of the ratiometric data significantly. The orthogonality of two split intein should be demonstrated by using their systems because protein splicing by inteins is strongly extein-dependent.

Response to R4PF-part5

The inteins gp41-1 and NrdJ-1 have already been shown to be orthogonal by Pinto *et al.* (doi:10.1038/s41467-020-15272-2), which we cite in the main text.
We have not seen any mis-spliced products from these inteins, such as N-NrdJ-1- or C-gp41-1, which would have appeared as additional bands of lower molecular weight on the immunoblots (**Supplementary Fig. 2b,7, 10c, and 12**).

Moreover, the orthogonal pairs of coiled-coils, which likely dimerize already at the secondary structure level before any intein or extein segments can fold, add a second level of orthogonality.

• *The author provided only one experimental data in Supplemental Fig 1 of immunoblotting and did not disclose any further sequence in detail. At least Supplemental Fig. 1 could be supplemented by covering all possible products using anti-Olla and Flag antibodies and provide the protein splicing efficiency quantitated for each of the two splicing steps. In theory, cleaved products might not interfere with NLuc/Fluc ratio. Do the authors have any evidence to assume that is the case?*

Response to R4PF-part6

We updated **Supplementary Fig. 1** with an overexposed and contrast-enhanced immunoblot. We see a weak band for C-cleavage (~3%) using the fast-folding mNeonGreen as a surrogate extein sequence. Via densitometry, we could quantify that the addition of Coiled-Coils could enhance the protein splicing efficiency by ~9 fold. Please also see the full immunoblots in **Supplementary Fig. 7, 10c, and 12**, which show that the splicing efficiency was even higher for both inteins together with >99%.

• *The main claims generally focus on the Ratio-metric assay using NLuc/Fluc, the survival system using BSD could be more confusing for readers than making it clear to understand the reporter system as currently written.*

Response to R4PF-part7

We appreciate the Reviewer's suggestion but still find it valuable to showcase the versatility of the EXSISERS technology that goes beyond reporter signals. The capability to non-invasively couple the in- or exclusion of an exon to cell survival enables unbiased screenings for new splicing regulators, such as genome-wide CRISPR/Cas9-mediated KO screens. This powerful methodology was not possible before.

As an extension, one could also imagine to use dCas9-activator screens or instead use a triggerable toxin such as HSV-Tk, to screen for exon exclusion instead of inclusion.

R4PH:

H.

• *The abstract is concise and clear.*

• *There are several misleading statements in the introduction, the authors claim "fast" protein splicing but no speed or relevant time scale is given. Protein splicing is strongly context-dependent, has to be investigated for each extein. This claim is thus not validated in the manuscript. Moreover, there is no information about "trace-less" because the authors do not disclose the protein sequence for junction regions. "Traceless" should mean the spliced sequence is identical to the original protein sequence without a single mutation. Is this the case?*

• *The current data is not sufficiently supporting the conclusion because of several assumptions and lacks critical controls to verify each of the critical assumptions.*

Response to R4PH:

We have now added a series of additional control experiments to further support that the very efficient intein splicing does not alter the physiological isoform expression and are thus scarless.

To initially investigate and optimize the splicing efficiency of the inteins, we created a construct using mNeonGreen as an extein with folding rates of <10 minutes (please see Supplementary Fig. 1). Even under these extreme conditions, our final design, including coiled-coils (CCs) achieved a significantly greater extein to intein-extein ratio, indicating higher protein splicing efficiency (~9-fold increase in efficiency, **Supplementary Fig. 1**).

Nevertheless, the Reviewer is, of course, right that our measurements did not include precise timing and therefore we have changed the term from 'fast' to 'efficient' in the abstract and the introduction. Still, we used the term 'fast' in the beginning of the results section when we refer to gp41-1 and NrdJ-1 inteins since the literature described them as ultrafast splicing inteins (doi:10.1073/pnas.1701083114, doi:10.1021/jacs.7b02618).

Application of EXSISERS on *MAPT* showed a very high protein splicing efficiency (**Supplementary Fig. 7, Supplementary Fig. 10c, and Supplementary Fig. 12**). Please also refer to the detailed answer to R4PF. With the 'classic inteins', such as Ssp or Npu DnaE, intein splicing is highly dependent on the extein sequences, but with those 'ultrafast inteins' identified in metagenomic sources, the literature (doi:10.1002/1873-3468.13909) showed that they tolerate heterologous settings very well (only proline is not tolerated by all inteins in a heterologous context).

Besides the recently discovered classes of fast and efficient inteins, we like to refer to the nicely maintained database from the Iwaï lab (formerly maintained by New England Biolabs), where one can screen for inteins where the native extein sequences are identical or similar to the desired insertion site. As an example, we used this database, to search for inteins suitable to split Cas9 between position 573 and 574 (KIE|CFD), *Npu* intein with the native extein sequence (AEY|CFN) which critical +2 position fits to the intended Cas9 split-site (doi:10.1093/nar/gkv601). Notably, we did not see any difference in activity between WT Cas9 and Npu intein split-Cas9.

We neither introduced any extra Ser/Cys/Thr, nor did we change any amino acid to Ser/Cys/Thr, but merely used the natively occurring Ser/Cys/Thr of an exon, therefore we consider it justified to use the term 'traceless' or 'scarless'. Please also see the Materials and Methods section 'Generation of stable cell lines with tagged exons via CRISPR/Cas9', where we described how we inserted EXSISERS into the GOI.

We also added additional experimental controls, such as RT-qPCR on unmodified HEK293T cells (**Supplementary Fig. 14**) data to substantiate our data from **Fig. 3** in $\text{EXSISERS}_{\textit{MAPT}\text{:10NLuc-11FLuc}}$ cells. We also added additional dual-luciferase assays data from other clones to exclude clone-dependent artifacts (**Supplementary Fig. 17**). Moreover, we included additional full-range immunoblots to show the high protein splicing efficiency of the CCs-improved inteins (**Supplementary Fig. 7, Supplementary Fig. 10c,d,, and Supplementary Fig. 12**).

Decision Letter, third revision:

Date: 16th December 20 09:06:14
Last Sent: 16th December 20 09:06:14
Triggered By: Jie Wang
From: jie.wang@nature.com
To: gil.westmeyer@tum.de
Subject: Decision on Nature Cell Biology submission NCB-W40046C
Message: *Please delete the link to your author homepage if you wish to forward this email to co-authors.

Dear Professor Westmeyer,

Please accept our sincerest apologies for the length of time your manuscript has been under consideration at our journal. This is because referee 4 was unable to review the manuscript due to unforeseen circumstances, and we had to invite another reviewer (referee 5) to secure a full panel of referees with expertise covering the key aspects of the study. We thank you very much for your patience during the process.

Your manuscript, "Non-invasive and high-throughput interrogation of exon-specific isoform expression", has now been seen by three of our original referees (referee 1-3) and a new referee with expertise in inteins (referee 5). As you will see from their comments (attached below), while referees 1, 2 and 5 are satisfied with the revision, referee 3 continues to question the technical advance of EXSISERS. We believe that additional experiments and textual changes will be required to address his/her concerns before we can consider publication in Nature Cell Biology.

Nature Cell Biology editors discuss the referee reports in detail within the editorial team, including the chief editor, to identify key referee points that should be addressed with priority, and requests that are overruled as being beyond the scope of the current study. To guide the scope of the revisions, I have listed these points below. We are committed to providing a fair and constructive peer-review process, so please feel free to contact me if you would like to discuss any of the referee comments further.

In particular, it would be essential to:

a) demonstrate the advance of EXSISERS in identification of new regulators in CRIPSR/Cas9 screens by following up and validating top hits other than MBNL1/2 that are known to regulate Foxp1 splicing, as noted by referee 3:

‘Seemingly the real benefit of EXSISERS is in high-throughput applications, though this notion is also dubious—even in high-throughput screening applications it is likely not necessary to model the endogenous locus perfectly. If the authors believe that screening a recursively spliced locus is an exception, then they should

demonstrate that application with EXSISERS. It is plausible in theory but doubtful that such a capability would generate much enthusiasm without such a clear demonstration.

If the authors were to make an unpublished high-throughout discovery with EXSISERS, that might be grounds for acceptance. As it stands, a natural conclusion for their story would be to follow up and validate top hits other than MBNL1/2 from their genome-wide CRISPR screen. But so far they have not demonstrated anything new, and therefore it is unclear that they would find anything new.'

b) further clarify the technical advance over existing methods such as minigene, as noted by referee 3:

'The current text and the author's response to the review present EXSISERS as a desirable, general replacement for minigenes etc., which is not fully supported. While they do not entirely recapitulate the nuances of splicing, mini-genes are much faster to generate than ~4-6 weeks. For most applications where nuance matters, established endogenous locus RNA and protein techniques will be far easier and more informative than a relatively complex new method like EXSISERS. The manuscript should state that this method is attractive when matching baseline molecular phenotypes to endogenous levels is important.'

c) All other referee concerns pertaining to methodological details, clarifications and textual changes, should also be addressed.

d) Finally please pay close attention to our guidelines on statistical and methodological reporting (listed below) as failure to do so may delay the reconsideration of the revised manuscript.In particular please provide:

- a Supplementary Figure including unprocessed images of all gels/blots in the form of a multi-page pdf file. Please ensure that blots/gels are labeled and the sections presented in the figures are clearly indicated.

- a Supplementary Table including all numerical source data in Excel format, with data for different figures provided as different sheets within a single Excel file. The file should include source data giving rise to graphical representations and statistical descriptions in the paper and for all instances where the figures present representative experiments of multiple independent repeats, the source data of all repeats should be provided.

We therefore invite you to take these points into account when revising the manuscript. In addition, when preparing the revision please:

- ensure that it conforms to our format instructions and publication policies (see below and www.nature.com/nature/authors/).

- provide a point-by-point rebuttal to the full referee reports verbatim, as provided at the end of this letter.

- provide the completed Editorial Policy Checklist (found

here https://www.nature.com/authors/policies/Policy.pdf), and Reporting Summary (found here https://www.nature.com/authors/policies/ReportingSummary.pdf). This is essential for reconsideration of the manuscript and these documents will be available to editors and referees in the event of peer review. For more information see http://www.nature.com/authors/policies/availability.html or contact me.

Nature Cell Biology is committed to improving transparency in authorship. As part of our efforts in this direction, we are now requesting that all authors identified as 'corresponding author' on published papers create and link their Open Researcher and Contributor Identifier (ORCID) with their account on the Manuscript Tracking System (MTS), prior to acceptance. ORCID helps the scientific community achieve unambiguous attribution of all scholarly contributions. You can create and link your ORCID from the home page of the MTS by clicking on 'Modify my Springer Nature account'. For more information please visit please visit www.springernature.com/orcid.

Please submit the revised manuscript files and the point-by-point rebuttal to the referee comments using this link:

[REDACTED]

*This url links to your confidential home page and associated information about manuscripts you may have submitted or be reviewing for us. If you wish to forward this email to co-authors, please delete the link to your homepage.

We would like to receive the revision within four weeks. If submitted within this time period, reconsideration of the revised manuscript will not be affected by related studies published elsewhere, or accepted for publication in Nature Cell Biology in the meantime. We would be happy to consider a revision even after this timeframe, but in that case we will consider the published literature at the time of resubmission when assessing the file.

We hope that you will find our referees' comments, and editorial guidance helpful. Please do not hesitate to contact me if there is anything you would like to discuss.

Best wishes,

Jie Wang

Jie Wang, PhD
Senior Editor
Nature Cell Biology

Tel: +44 (0) 207 843 4924
email: jie.wang@nature.com

Reviewers' Comments:

Reviewer #1:
Remarks to the Author:
The authors have addressed all my concerns. This is a tremendous amount of work with appropriate controls and methodological details. I have no more concerns for publication.

Reviewer #2:
Remarks to the Author:
In the revised manuscript, the authors conducted a series of experiments to address most of my concerns, and also give a detailed explanation on some of the point that they did not addressed with additional experiments. I think they made serious efforts to improve the paper. While I still have concerns on lack of new findings when they applied EXSISERS in this work, I think the method itself is valuable for further application, especially in the potential application on genome-wide CRISPR/Cas9 screen. Therefore I am generally satisfied with their revision.

Reviewer #3:
Remarks to the Author:
The authors responded well to previous comments, but (as other reviewers have noted) nevertheless they have not demonstrated that EXSISERS technology is broad enough of a tool to generate novel biological findings unattainable by other methodologies.

The current text and the author's response to the review present EXSISERS as a desirable, general replacement for minigenes etc., which is not fully supported. While they do not entirely recapitulate the nuances of splicing, mini-genes are much faster to generate than ~4-6 weeks. For most applications where nuance matters, established endogenous locus RNA and protein techniques will be far easier and more informative than a relatively complex new method like EXSISERS. The manuscript should state that this method is attractive when matching baseline molecular phenotypes to endogenous levels is important.

Seemingly the real benefit of EXSISERS is in high-throughput applications, though this notion is also dubious—even in high-throughput screening applications it is likely not necessary to model the endogenous locus perfectly. If the authors believe that screening a recursively spliced locus is an exception, then they should demonstrate that application with EXSISERS. It is plausible in theory but doubtful that such a capability would generate much enthusiasm without such a clear demonstration.

If the authors were to make an unpublished high-throughout discovery with EXSISERS, that might be grounds for acceptance. As it stands, a natural conclusion for their story would be to follow up and validate top hits other than MBNL1/2 from their genome-wide CRISPR screen. But so far they have not demonstrated anything new, and therefore it is unclear that they would find anything new.

Reviewer #5:
Remarks to the Author:
Having looked in detail at the responses by the authors to the comments raised by the original referee 4, I think the authors have adequately addressed the concerns/points raised and the additioanl controls requested regarding validating the intein splicing efficiency and orthogonality.

However, I did not note the annotated sequences of the key genetic constructs were available. I suggest the authors to make them available either as in the supplementary or by uploading to a public database. In addition, it would be beneficial to the wide community to deposit the key constructs from the study in public repositories like Addgene.

-Baojun Wang
University of Edinburgh

GUIDELINES FOR SUBMISSION OF NATURE CELL BIOLOGY TECHNICAL REPORTS

READABILITY OF MANUSCRIPTS – Nature Cell Biology is read by cell biologists from diverse backgrounds, many of whom are not native English speakers. Authors should aim to communicate their findings clearly, explaining technical jargon that might be unfamiliar to non-specialists, and avoiding non-standard abbreviations. Titles and abstracts should concisely communicate the main findings of the study, and the background, rationale, results and conclusions should be clearly explained in the manuscript in a manner accessible to a broad cell biology audience. Nature Cell Biology uses British spelling.

TECHNICAL REPORT FORMAT

TITLE – should be no more than 100 characters including spaces, without punctuation and avoiding technical terms, abbreviations, and active verbs.

AUTHOR NAMES – should be given in full.

AUTHOR AFFILIATIONS – should be denoted with numerical superscripts (not symbols) preceding the names. Full addresses should be included, with US states in full and providing zip/post codes. The corresponding author is denoted by: "Correspondence should be addressed to [initials]."

ABSTRACT – should not exceed 150 words and should be unreferenced. This paragraph is the most visible part of the paper and should briefly outline the background and rationale for the work, and accurately summarize the main results and conclusions. Key genes, proteins and organisms should be specified to ensure discoverability of the paper in online searches.

TEXT – the main text consists of the Introduction, Results, and Discussion sections and must not exceed 3000 words including the abstract. The Introduction should expand on the background relating to the work. The Results should be divided in subsections with subheadings, and should provide a concise and accurate description of the experimental findings. The Discussion should expand on the findings and their implications. All relevant primary literature should be cited, in particular when discussing the background and specific findings.

ACKNOWLEDGEMENTS – should be kept brief. Professional titles and affiliations are unnecessary. Grant numbers can be listed.

AUTHOR CONTRIBUTIONS – must be included after the Acknowledgements, detailing the contributions of each author to the paper (e.g. experimental work, project planning, data analysis etc.). Each author should be listed by his/her initials.

FINANCIAL AND NON-FINANCIAL COMPETING INTERESTS – the authors must include one of three declarations: (1) that they have no financial and non-financial competing interests; (2) that they have financial and non-financial competing interests; or (3) that they decline to respond, after the Author Contributions section. This statement will be published with the article, and in cases where financial and non-financial competing interests are declared, these will be itemized in a web supplement to the article. For further details please see https://www.nature.com/licenceforms/nrg/competing-interests.pdf.

REFERENCES – are limited to a total of 40 in the main text and Methods combined (although they could be extended at the discretion of the editor). They must be numbered sequentially as they appear in the main text, tables and figure legends and Methods and must follow the precise style of Nature Cell Biology references. References only cited in the Methods should be numbered consecutively following the last reference cited in the main text. References only associated with Supplementary Information (e.g. in supplementary legends) do not count toward the total reference limit and do not need to be cited in numerical continuity with references in the main text. Only published papers can be cited, and each publication cited should be included in the numbered reference list, which should include the manuscript titles. Footnotes are not permitted.

METHODS – Nature Cell Biology publishes methods online. The methods section should be provided as a separate Word document, which will be copyedited and appended to the manuscript PDF, and incorporated within the HTML format of the paper.

Methods should be written concisely, but should contain all elements necessary to allow interpretation and replication of the results. As a guideline, Methods sections

typically do not exceed 3,000 words. The Methods should be divided into subsections listing reagents and techniques. When citing previous methods, accurate references should be provided and any alterations should be noted. Information must be provided about: antibody dilutions, company names, catalogue numbers and clone numbers for monoclonal antibodies; sequences of RNAi and cDNA probes/primers or company names and catalogue numbers if reagents are commercial; cell line names, sources and information on cell line identity and authentication. Animal studies and experiments involving human subjects must be reported in detail, identifying the committees approving the protocols. For studies involving human subjects/samples, a statement must be included confirming that informed consent was obtained. Statistical analyses and information on the reproducibility of experimental results should be provided in a section titled “Statistics and Reproducibility”.

All Nature Cell Biology manuscripts submitted on or after March 21 2016, must include a Data availability statement at the end of the Methods section. For Springer Nature policies on data availability see http://www.nature.com/authors/policies/availability.html; for more information on this particular policy see http://www.nature.com/authors/policies/data/data-availability-statements-data-citations.pdf. The Data availability statement should include:

• Accession codes for primary datasets (generated during the study under consideration and designated as "primary accessions") and secondary datasets (published datasets reanalysed during the study under consideration, designated as "referenced accessions"). For primary accessions data should be made public to coincide with publication of the manuscript. A list of data types for which submission to community-endorsed public repositories is mandated (including sequence, structure, microarray, deep sequencing data) can be found here http://www.nature.com/authors/policies/availability.html#data.

• Unique identifiers (accession codes, DOIs or other unique persistent identifier) and hyperlinks for datasets deposited in an approved repository, but for which data deposition is not mandated (see here for details http://www.nature.com/sdata/data-policies/repositories).

• At a minimum, please include a statement confirming that all relevant data are available from the authors, and/or are included with the manuscript (e.g. as source data or supplementary information), listing which data are included (e.g. by figure panels and data types) and mentioning any restrictions on availability.

• If a dataset has a Digital Object Identifier (DOI) as its unique identifier, we strongly encourage including this in the Reference list and citing the dataset in the Methods.

We recommend that you upload the step-by-step protocols used in this manuscript to the Protocol Exchange. More details can found at www.nature.com/protocolexchange/about.

DISPLAY ITEMS – main display items are limited to 6-8 main figures and/or main

tables. For Supplementary Information see below.

FIGURES – Colour figure publication costs $620 for the first, and $310 for each subsequent colour figure. All panels of a multi-panel figure must be logically connected and arranged as they would appear in the final version. Unnecessary figures and figure panels should be avoided (e.g. data presented in small tables could be stated briefly in the text instead).

All imaging data should be accompanied by scale bars, which should be defined in the legend.
Cropped images of gels/blots are acceptable, but need to be accompanied by size markers, and to retain visible background signal within the linear range (i.e. should not be saturated). The boundaries of panels with low background have to be demarked with black lines. Splicing of panels should only be considered if unavoidable, and must be clearly marked on the figure, and noted in the legend with a statement on whether the samples were obtained and processed simultaneously. Quantitative comparisons between samples on different gels/blots are discouraged; if this is unavoidable, it should only be performed for samples derived from the same experiment with gels/blots were processed in parallel, which needs to be stated in the legend.

Figures should be provided at approximately the size that they are to be printed at (single column is 86 mm, double column is 170 mm) and should not exceed an A4 page (8.5 x 11"). Reduction to the scale that will be used on the page is not necessary, but multi-panel figures should be sized so that the whole figure can be reduced by the same amount at the smallest size at which essential details in each panel are visible. In the interest of our colour-blind readers we ask that you avoid using red and green for contrast in figures. Replacing red with magenta and green with turquoise are two possible colour-safe alternatives. Lines with widths of less than 1 point should be avoided. Sans serif typefaces, such as Helvetica (preferred) or Arial should be used. All text that forms part of a figure should be rewritable and removable.

We accept files from the following graphics packages in either PC or Macintosh format:

- For line art, graphs, charts and schematics we prefer Adobe Illustrator (.AI), Encapsulated PostScript (.EPS) or Portable Document Format (.PDF). Files should be saved or exported as such directly from the application in which they were made, to allow us to restyle them according to our journal house style.

- We accept PowerPoint (.PPT) files if they are fully editable. However, please refrain from adding PowerPoint graphical effects to objects, as this results in them outputting poor quality raster art. Text used for PowerPoint figures should be Helvetica (preferred) or Arial.

- We do not recommend using Adobe Photoshop for designing figures, but we can accept Photoshop generated (.PSD or .TIFF) files only if each element included in the figure (text, labels, pictures, graphs, arrows and scale bars) are on separate layers. All text should be editable in ‘type layers' and line-art such as graphs and other simple schematics should be preserved and embedded within 'vector smart

objects' - not flattened raster/bitmap graphics.

- Some programs can generate Postscript by 'printing to file' (found in the Print dialogue). If using an application not listed above, save the file in PostScript format or email our Art Editor, Allen Beattie for advice (a.beattie@nature.com).

Regardless of format, all figures must be vector graphic compatible files, not supplied in a flattened raster/bitmap graphics format, but should be fully editable, allowing us to highlight/copy/paste all text and move individual parts of the figures (i.e. arrows, lines, x and y axes, graphs, tick marks, scale bars etc). The only parts of the figure that should be in pixel raster/bitmap format are photographic images or 3D rendered graphics/complex technical illustrations.

All placed images (i.e. a photo incorporated into a figure) should be on a separate layer and independent from any superimposed scale bars or text. Individual photographic images must be a minimum of 300+ DPI (at actual size) or kept constant from the original picture acquisition and not decreased in resolution post image acquisition. All colour artwork should be RGB format.

FIGURE LEGENDS – must not exceed 350 words for each figure to allow fit on a single printed NCB page together with the figure. They must include a brief title for the whole figure, and short descriptions of each panel with definitions of the symbols used, but without detailing methodology.

TABLES – main tables should be provided as individual Word files, together with a brief title and legend. For supplementary tables see below.

SUPPLEMENTARY INFORMATION – Supplementary information is material directly relevant to the conclusion of a paper, but which cannot be included in the printed version in order to keep the manuscript concise and accessible to the general reader. Supplementary information is an integral part of a Nature Cell Biology publication, and should be prepared and presented with as much care as the main display item, but it must not include non-essential data or text, which may be removed at the editor's discretion. All supplementary material is fully peer-reviewed and published online as part of the HTML version of the manuscript. Supplementary Figures and Supplementary Notes are appended at the end of the main PDF of the published manuscript.

Supplementary items should relate to a main text figure, wherever possible, and should be mentioned sequentially in the main manuscript, designated as Supplementary Figure, Table, Video, or Note, and numbered continuously (e.g. Supplementary Figure 1, Supplementary Figure 2, Supplementary Table 1, Supplementary Table 2 etc.).

Unprocessed scans of all key data generated through electrophoretic separation techniques need to be presented in a supplementary figure that should be labeled and numbered as the final supplementary figure, and should be mentioned in every relevant figure legend. This figure does not count towards the total number of figures and is the only figure that can be displayed over multiple pages, but should

be provided as a single file, in PDF or TIFF format. Data in this figure can be displayed in a relatively informal style, but size markers and the figures panels corresponding to the presented data must be indicated.

The total number of Supplementary Figures (not including the “unprocessed scans” Supplementary Figure) should not exceed the number of main display items (figures and/or tables (see our Guide to Authors and March 2012 editorial http://www.nature.com/ncb/authors/submit/index.html#suppinfo; http://www.nature.com/ncb/journal/v14/n3/index.html#ed). No restrictions apply to Supplementary Tables or Videos, but we advise authors to be selective in including supplemental data.

Each Supplementary Figure should be provided as a single page and as an individual file in one of our accepted figure formats and should be presented according to our figure guidelines (see above). Supplementary Tables should be provided as individual Excel files. Supplementary Videos should be provided as .avi or .mov files up to 50 MB in size. Supplementary Figures, Tables and Videos much be accompanied by a separate Word document including titles and legends.

GUIDELINES FOR EXPERIMENTAL AND STATISTICAL REPORTING

REPORTING REQUIREMENTS – To improve the quality of methods and statistics reporting in our papers we have recently revised the reporting checklist we introduced in 2013. We are now asking all life sciences authors to complete two items: an Editorial Policy Checklist (found here https://www.nature.com/authors/policies/Policy.pdf) that verifies compliance with all required editorial policies and a Reporting Summary (found here https://www.nature.com/authors/policies/ReportingSummary.pdf) that collects information on experimental design and reagents. These documents are available to referees to aid the evaluation of the manuscript. Please note that these forms are dynamic ‘smart pdfs’ and must therefore be downloaded and completed in Adobe Reader. We will then flatten them for ease of use by the reviewers. If you would like to reference the guidance text as you complete the template, please access these flattened versions at http://www.nature.com/authors/policies/availability.html.

STATISTICS – Wherever statistics have been derived the legend needs to provide the n number (i.e. the sample size used to derive statistics) as a precise value (not a range), and define what this value represents. Error bars need to be defined in the legends (e.g. SD, SEM) together with a measure of centre (e.g. mean, median). Box plots need to be defined in terms of minima, maxima, centre, and percentiles. Ranges are more appropriate than standard errors for small data sets. Wherever statistical significance has been derived, precise p values need to be provided and the statistical test used needs to be stated in the legend. Statistics such as error bars must not be derived from n<3. For sample sizes of n<5 please plot the individual data points rather than providing bar graphs. Deriving statistics from technical replicate samples, rather than biological replicates is strongly discouraged. Wherever statistical significance has been derived, precise p values need to be provided and the statistical test stated in the legend.

Information on how many times each experiment was repeated independently with similar results needs to be provided in the legends and/or Methods for all experiments, and in particular wherever representative experiments are shown.

We strongly recommend the presentation of source data for graphical and statistical analyses as a separate Supplementary Table, and request that source data for all independent repeats are provided when representative experiments of multiple independent repeats, or averages of two independent experiments are presented. This supplementary table should be in Excel format, with data for different figures provided as different sheets within a single Excel file. It should be labelled and numbered as one of the supplementary tables, titled “Statistics Source Data”, and mentioned in all relevant figure legends.

Author Rebuttal, fourth revision:

Response to Reviewers' Comments on the revised manuscript NCB-W40046C, now NCB-W40046D.

Reviewer #1 (on revised manuscript):

Remarks to the Author:

The authors have addressed all my concerns. This is a tremendous amount of work with appropriate controls and methodological details. I have no more concerns for publication.

Reply to Reviewer #1

We thank the reviewer for the constructive criticism and the compliment on our revisions.

Reviewer #2 (on revised manuscript):

Remarks to the Author:

In the revised manuscript, the authors conducted a series of experiments to address most of my concerns, and also give a detailed explanation on some of the point that they did not addressed with additional experiments. I think they made serious efforts to improve the paper. While I still have concerns on lack of new findings when they applied EXSISERS in this work, I think the method itself is valuable for further application, especially in the potential application on genome-wide CRISPR/Cas9 screen. Therefore I am generally satisfied with their revision.

Reply to Reviewer #2

We thank the reviewer for the thoughtful comments that helped to improve our manuscript.
We have now also followed up on a secondary hit in our EXSISERS CRISPR screen for splice modulators of *FOXP1* and validated that *MOV10*, an RNA helicase, is a previously unrecognized factor that favors the exclusion of FOXP1 exon18b (**Supplementary Fig. 21a,b,c**).

Reviewer #3 (on revised manuscript):

Remarks to the Author:

The authors responded well to previous comments, but (as other reviewers have noted) nevertheless they have not demonstrated that EXSISERS technology is broad enough of a tool to generate novel biological findings unattainable by other methodologies.

Reply to Reviewer #3, point 1:

We thank the reviewer for the additional comments on our Technical Report.
As suggested by the Reviewer, we have now conducted another in-depth analysis of the unbiased EXSISERS CRISPR screen and have found that *MOV10*, an RNA helicase, is a previously unknown factor promoting *FOXP1 exon18b* exclusion.
Please see our detailed response to point 3 below.

We have now also expanded on the EXSISERS$_{MAPT}$ reporter in patient-derived iPSCs and monitored 4R isoform expression during the differentiation process into cortical neurons in wt and IVS10+16 c>t genotype, to find clearly aberrant isoform expression already in undifferentiated cell states at levels clearly below the detection limit of immunoblot.
Please see our detailed response to point 2 below.

Reviewer #3, point 2:

The current text and the author's response to the review present EXSISERS as a desirable, general replacement for minigenes etc., which is not fully supported. While they do not entirely recapitulate the nuances of splicing, mini-genes are much faster to generate than ~4-6 weeks. For most applications where nuance matters, established endogenous locus RNA and protein techniques will be far easier and more informative than a relatively complex new method like EXSISERS. The manuscript should state that this method is attractive when matching baseline molecular phenotypes to endogenous levels is important.

Reply to Reviewer #3, point 2:

Nowhere in the texts had we made the unnecessarily broad claim that EXSISERS is a "general replacement for minigenes."
To clarify this, we now emphasize - exactly along the line of the Reviewer- that minigenes are certainly powerful and valuable tools that have contributed substantial insights into alternative splicing but may not always reflect all nuances of splicing for obvious reasons.

We write in a modified paragraph of the introduction:

> Although this method can efficiently give valuable insights into alternative splicing, it may not always reflect the physiological processes, because partial intron/exon motifs may be overexpressed at unnatural levels, while essential regulatory sequences may be truncated.

Instead of a general claim, we focused on specific cases of strong biomedical interest in which "matching baseline molecular phenotypes to endogenous levels is important":
(1) monitoring of *MAPT* isoform expression in patient-derived cells and
(2) unbiased screening for *FOXP1* splice modulation (point 3 below).

For *MAPT*, there is converging evidence that transiently expressed minigenes do not reflect all aspects of the physiological splicing behavior, especially if they are just transiently overexpressed and not stably integrated, which we have streamlined for EXSISERS via a convenient double-selection process (**Supplementary Fig. 4)**.
Upon the Reviewer's previous request (please see point R3P2: below), we confirmed this possible constraint directly by conducting a head-to-head comparison of EXSISERS$_{MAPT}$ to *MAPT* minigenes.
We found that the minigene-based reporter incorrectly reported the true tau isoform-ratio by a factor of ~4 (please compare **Supplementary Fig. 5c** vs. **Supplementary Fig. 10e**), while EXSISERS$_{MAPT}$ showed a truthful depiction of the endogenous splice-ratio.

To again demonstrate the advantages of EXSISERS, we have thus also worked further with the EXSISERS$_{MAPT}$ reporter in patient-derived IPSC and monitored tau isoform expression over the differentiation process from neural precursor cells into cortical neurons lasting 3 months.

This state-of-the-art cell culture model is valuable for studying Frontotemporal lobar degeneration (FTLD), which affects cortical structures and not subcortical structures as in Parkinson's disease.
Thanks to the very high sensitivity of the dual-luciferase system, we could detect the fractional 4R isoform expression already during the early phases of the differentiation far below the sensitivity of immunoblot. Usually, only 3R tau is detected during the differentiation process. (doi: 10.1093/hmg/ddv246).
Thus, we could find that the 4R isoform expression of the mutant is already strongly (4-fold) elevated in the undifferentiated state. The expression then proceeds along a non-monotonical trajectory in several "waves" as opposed to the gradual increase in the wildtype.
This is an interesting observation as it points to a more complex splicing behavior in different time windows during which one can now seek to intervene pharmacologically to approximate the wild-type condition.

The ratiometric, highly sensitive readout enables using a minuscule amount of cell material to report relative changes in exon inclusion level on the protein level, allowing affordable high-throughput screenings, already non-matured smNPCs before they are detectable by classical protein-level detection methods.
Thus, the EXSISERS reporter neurons, differentiated from patient-derived IPSC, will be a valuable resource to monitor *MAPT* regulation close to the physiological condition.

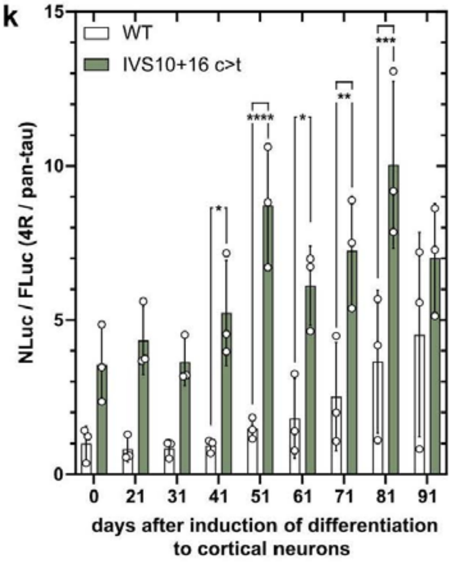

New Figure 2k | WT and IVS10+16 c>t iPSCs iPSCs were differentiated into cortical neurons over a time course of 3 months. Depicted are NLuc/FLuc ratios normalized to WT at day 0. Error bars and dotted lines represent standard deviation (*n* = 3). Only selected results of ANOVA post-hoc tests are shown with **, ***, and **** denoting p-values smaller than 0.01, 0.001, and 0.0001 respectively (full statistical results are available in **Supplementary Table 1**)

Reviewer #3, point 3:

Seemingly the real benefit of EXSISERS is in high-throughput applications, though this notion is also dubious—even in high-throughput screening applications it is likely not necessary to model the endogenous locus perfectly. If the authors believe that screening a recursively spliced locus is an exception, then they should demonstrate that application with EXSISERS. It is plausible in theory but doubtful that such a capability would generate much enthusiasm without such a clear demonstration.

If the authors were to make an unpublished high-throughout discovery with EXSISERS, that might be grounds for acceptance. As it stands, a natural conclusion for their story would be to follow up and validate top hits other than MBNL1/2 from their genome-wide CRISPR screen. But so far they have not demonstrated anything new, and therefore it is unclear that they would find anything new.

Reply to Reviewer #3, point 3:

Upon the suggestion of the reviewer, we have now followed-up on a secondary hit from the EXSISERS CRISPR screen that was enriched in both selection conditions of the survival screen: *MOV10* (**Supplementary Fig. 21a**.

We then confirmed *MOV10* via an independent sgRNA (#2) not previously used in the screen, which again lead to the survival of $\text{EXSISERS}_{\text{FOXP1:18bBSD}}$ colonies as compared to a control sgRNA (**Supplementary Fig. 21b**).

We then further confirmed via RT-PCR that *FOXP1* exon 18b was included in *MOV10*-KO cells in a blasticidin S concentration-dependent manner (**Supplementary Fig. 21c**). These data demonstrate that the RNA helicase MOV10 is a further factor for exon 18b suppression, albeit much weaker than MBNL1.

MOV10 is an interesting finding because it is not expressed in stem cells but upregulated upon differentiation (doi:10.1093/nar/gkaa1054). As an RNA Helicase, it may regulate access of splice regulators such as MBNL1 to the pre-mRNA, which may invite in-depth mechanistic studies, which are, however, clearly beyond our **Technical Report**.

These additional data show that, while *MBNL1* was found by an educated guess (doi:10.1038/nature12270), our comprehensive EXSISERS screen proved that MBNL1 is the main proteinogenic suppressors of FOXP exon 18b and identified an additional auxiliary factor that was previously not associated with splice regulation.

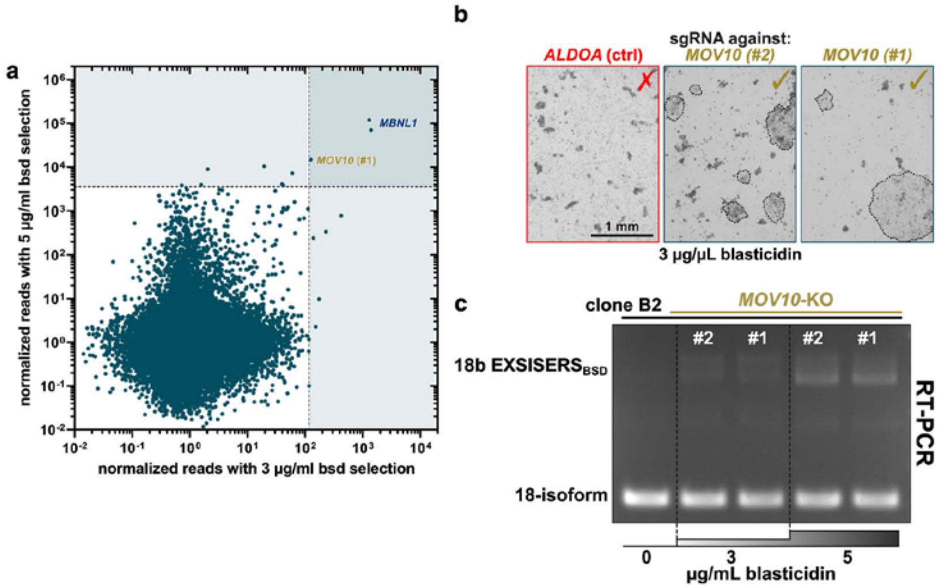

New Supplementary Fig. 21 | RNA helicase MOV10 is involved in *FOXP1* exon 18b suppression. a, Scatterplot of the reads (normalized to pre-selection reads) from both selection conditions (3 and 5 µg/ml blasticidin S). The areas highlighted in blue indicate the most strongly enriched sgRNAs for each selection condition. **b,** The enrichment of *MOV10* in the screen was confirmed by an independent *MOV10*-targeting sgRNA (#2) not used in the screen. Shown are colonies on a T75 flask 2 weeks after selection with 3 µg/ml blasticidin S and after transfection with CRISPR/Cas9 components against *ALDOA* (unrelated control gene, <10 colonies), the independent sgRNA #2 (>200 colonies in T75 flask)), and the sgRNAs against *MOV10* #1 used in the screen (>100 colonies). **c,** RT-PCR showing the blasticidin-concentration-dependent inclusion of *FOXP1* exon 18b from the colonies surviving blasticidin S selection shown in b, labeled with the respective sgRNAs.

We would like to emphasize again that we had, upon recommendation of the editor based on our pre-submission inquiry, submitted the current manuscript as a **Technical Report**, "which may involve a new biological discovery to prove the usefulness of the technique, but this is not a requirement."

We thus think that thanks to the Reviewers' constructive criticism, we have now characterized in great detail the technical advances of EXSISERS and have demonstrated the type of high-throughput reporter measurements and whole-genome survival screens, which EXSISERS enables to generate biomedical insights on isoform expression.

Reviewer #5:

Remarks to the Author:

Having looked in detail at the responses by the authors to the comments raised by the original referee 4, I think the authors have adequately addressed the concerns/points raised and the additioanl controls requested regarding validating the intein splicing efficiency and orthogonality.

However, I did not note the annotated sequences of the key genetic constructs were available. I suggest the authors to make them available either as in the supplementary or by uploading to a public database. In addition, it would be beneficial to the wide community to deposit the key constructs from the study in public repositories like Addgene.

Reply to Reviewer #5

We thank the additional reviewer for the positive feedback.
We have now combined all sequences of the EXSISERS components in the extensive **Supplementary Table 1** and will, of course, be happy to share them publicly.

Response to Reviewers' Comments on the initially submitted manuscript

Reviewer #1 (Remarks to the Author):

In this manuscript, Truong et al. describe a new tool, EXSISERS, to assess changes in splicing isoforms at the protein level and/or tag cells with specific protein splicing isoforms. The authors are taking advantage of the capacity of inteins to splice themselves out at the protein level, without affecting the RNA or coding sequence where there are integrated in. Using these inteins, they have shown with a wide set of examples, how they can insert at the endogenous level, in the alternatively spliced exon of choice, a reporter that can be spliced out at the protein level by specific inteins. With this system, by looking at expression of the protein reporter, which can be a luciferase protein, a blasticidine resistant gene, a fluorescent protein, an halo tag that goes to the membrane for cell sorting, one can identify, quantify, cell sort, live image cells expressing a specific splicing isoform of interest without the need of artificial reporters, splicing-specific antibodies, or the need to rely on RNA-based methodologies that most of the times are not impacting proteins at the same level. With this new system, one can assess the real splicing isoforms that exist at the protein level, follow them, manipulate them and even use them as a read out for CRISPR screening, imaging and sorting. It is extremely versatile and useful for studying many mechanisms and more importantly the biological relevance of a particular splicing variant at the protein level, and not the RNA level as we usually do (which underestimates all the post-transcriptional effects that could come from the new splice variant). Moreover, in the manuscript, the use of RfxCas13d and PspCas13b to specifically knock down one specific splicing isoform is also studied, bringing light to this also new and poorly understood tool. Key aspects of the crRNA design and if it is better to target the nascent pre-mRNA or the mature mRNA are shown.
Overall the manuscript is clear, robust and full of insightful new tools and recommendations to work with specific splicing isoforms at all possible levels. It is therefore of great interest for the scientific community and deserves publication if some concerns are addressed first.

Comments:

R1P1:

***1)** Since this is a manuscript selling a new tool, it would be nice if the authors comment whether it is difficult to endogenously tag at the homozygous level such reporter sequences. Have they tried many different type of cells? Which is the size of the biggest reporter they successfully inserted? I say this, because it is known that not all cells are easy to CRISPR tag and it is even more difficult to tag the two alleles, and even more two regions of the same gene at the two alleles. What happens in cells with more than two alleles? Is it really important to tag all alleles? All this could be commented to reinforce feasibility.*

Response to R1P1:

We thank the Reviewer for acknowledging the value of EXSISERS for studying isoform-specific expression.
With respect to cell types, we tested HEK293T, Neuro-2a, and several human induced pluripotent stem cell lines. Homozygous knock-ins were also achieved by a collaboration partner using an unrelated gene in HepG2 and HCT116 cells.
Although the efficiency of CRISPR/Cas9 type of gene editing tools will surely further improve and make systems such as EXSISERS even more convenient to use in the future, we have already achieved high single-copy knock-in efficiency and also high homozygous knock-in efficiency using the constructs we describe in detail in **Supplementary Fig. 3 and 4**. As an

example, out of randomly chosen 7 puromycin resistant clones, all were positive on at least one allele for EXSISERS$_{MAPT:10HaloTag}$ (new **Supplementary Fig. 22**).
EXSISERS$_{MAPT:10HaloTag}$ is our most complex construct (2.1 kbp without selection cassette and 4.4 kbp with selection cassette) containing two transmembrane segments and an extracellular HaloTag domain. Of the 7 positives, 3 were homozygous for EXSISERS$_{MAPT:10HaloTag}$.

With respect to ploidy, HEK293T cells, like many cell lines, are often triploid for most of the chromosomes, including chromosome 17, where *MAPT* is located. This property did not complicate CRISPR-Cas9-mediated genomic integration of the EXSISERS constructs.
Also, knock-in efficiency was good in human induced pluripotent stem cells (hiPSCs), which are known to be more difficult to modify by CRISPR, using the very same optimized components. When targeting exon 10, out of 21 picked clones, 14 (67%) were heterozygous, and 2 were homozygous (10%) for EXSISERS$_{MAPT:10NLuc}$, resulting in a total targeting efficiency of 76%. Similar targeting efficiency was achieved for exon 11 in hiPSCs for 15 picked clones with 11 clones being heterozygous (73%) and 3 clones (20%) being homozygous for EXSISERS$_{MAPT:11FLuc}$ (93% total efficiency).

As the Reviewer has already pointed out, homozygous targeting is indeed not necessary. We only used homozygous lines for subsequent analysis to show that EXSISERS is minimally invasive. Else, one could argue from the immunoblot analysis that the bands shown in, *e.g.*, **Fig. 2**, are from the untargeted WT allele. Thus, we can definitively conclude that the bands in our experiments are indeed the result of protein splicing. For standard experiments, heterozygous insertions can already be sufficient and can be obtained with high targeting efficiency.

R1P2:

2) *It is also important to prove that there is no effect on the endogenous transcript nor protein. That splicing occurs normally and the protein levels are not affected by insertion of these reporters and inteins. I don't think the authors have done this properly in the manuscript. Actually in Fig.2d, there is more 4R isoform in HEK than in the WT-EXISERS clone. Shouldn't these two cells be comparable? It is important to show that splicing patterns are not affected by insertion of these constructs, that protein levels are not affected, that function is not affected and that splicing could even change if necessary, such as in their iPS differentiation system. Also, can inteins have off target effects? This is not mentioned nor proved.*

Response to R1P2:

We have verified all EXSISERS lines carefully at the RNA and protein level and have added immunoblot (new **Supplementary Fig. 5,6,7,8,9 and 12**) and RT-qPCR (new **Supplementary Fig. 14**) data to show that there are no obvious detectable alterations in of the expressed isoforms and that all results from the EXSISERS reporters are in line with the data acquired on RNA level.

Concerning the variability of *MAPT* isoform patterns from different cells, it is important to mention that HEK293T is not a clonal cell line and showed some population variability. Analysis of HEK293T clones without *MAPT* modification showed only minor expression of 0N4R within a certain biological variation (**Supplementary Fig. 8**).

As per **Fig. 2d**, we have now performed densitometry on the 0N3R and 0N4R bands from an 16-bit uncompressed tiff file using the automated analysis from Image Lab (v6.1.0 build 7, Bio-Rad) and did not observe any obvious change in exon 10 inclusion between HEK293T WT cells and EXSISERS$_{\mathit{MAPT}\text{:10NLuc-11FLuc}}$ cells, which both showed ~3% inclusion of 4R-tau (new **Supplementary Fig. 5b**).

In comparison, the pathologic mutation IVS10+16 c>t increased the fractional inclusion by ~3.7-fold), which is comparable to what we see from dual-luciferase EXSISERS (~4-fold), new **Supplementary Fig. 10e**) and also in accordance with the literature (2–6-fold, DOI:10.1074/jbc.274.21.15134 and DOI:10.1016/j.molbrainres.2005.02.014).

In addition, we also included a new immunoblot where we showed that EXSISERS$_{\mathit{MAPT}\text{:10NLuc-11FLuc}}$ cells are also comparable to the parental HEK293T cells in its response towards small molecule splicing modulators, such as 5'-iodotubercidin (ITU) (new **Supplementary Fig. 11 and 12**).

As further evidence for the reporter lines' physiological state, we had shown in main **Fig. 2c** that the tau filaments are formed in EXSISERS$_{\mathit{MAPT}\text{:10NLuc-11FLuc}}$ cells. Since we chose to use only homozygous EXSISERS cell lines for all experiments, those filaments must be formed from tau proteins that underwent protein splicing.
Also, the functional aspects on RNA-level, such as the regulatory hairpin of *MAPT*, were functional after EXSISERS insertion, as the well-characterized hairpin-destabilizing IVS10+16 c>t mutation led to a dramatic increase of exon 10 inclusion. As seen in main **Fig. 2d,e, and h**, and the new **Supplementary Fig. 5b,6,7, and 8** all other clones of EXSISERS$_{\mathit{MAPT\text{-}IVS10+16}\text{:10NLuc-11FLuc}}$ IVS10+16 c>t always showed a more prominent inclusion of exon 10 (4R isoform) compared to the WT counterpart, unmodified HEK293T cells and their clones.

The behavior of the EXSISERS construct used to screen for splicing modulators of FOXP1 (EXSISERS$_{\mathit{FOXP1}\text{:18b-BSD}}$) also indicates that splicing was not affected, as cells with homozygous insertion of EXSISERS$_{\text{BSD}}$ exon 18b did not show a changed blasticidin S sensitivity compared to HEK293T WT cells (data not shown). Since a minimal lethal blasticidin S concentration of 3 µg/mL was applied, even a minor increase in exon 18b inclusion would result in a surviving population of cells. As the exon 18b inclusion rate was already 0 % for cells lacking a MBNL1/2 KO in EXSISERS$_{\mathit{FOXP1}\text{:18b-BSD}}$ and HEK293T WT cells (**Main Fig. 4d, e**), a decrease of the inclusion rate would not have been possible.

Importantly, in the case of EXSISERS, we do not have to predict where a splice modulator, such as MBNL1 could bind, as the entire gene locus is present.
This stands in stark contrast to minigenes, where only those parts of a gene that are suspected to be involved in the splicing regulation are included in an artificial reporter system, resulting in a biased or knowledge-based screen. Please also see the comparison of EXSISERS with minigenes as part of our response to Reviewer 3 (R3P1 and R3P2).

Regarding off-targets of intein-splicing: Inteins originated from prokaryotes, archaea, algal cells, yeast, and other fungi. The protein splicing mechanism relies on autocatalysis and thus does not use up or interfere with any host proteins, nucleic acids, or any other host factors. Inteins are used in all kingdoms of life for biotechnological applications such as heterologous utilization in vertebrates, including mammals that do not have any inteins in the genome

natively. This heterologous usage in mammals, *e.g.*, to split Cas9 using protein trans-splicing in for rAAV delivery into pigs, did not show any side- or off-target effects on the organisms *in vivo* (doi:10.1038/s41551-019-0501-5 and doi:10.1038/s41591-019-0738-2). We have not made any observations in any of our EXSISERS implementations that would indicate such off-target effects.

R1P3:

3) A kind of related question: can you insert the NLuc/FLuc reporter anywhere in the exon regardless of the regulatory splicing sequences?

And how come increasing considerably the exon size has no effect on exon recognition and recruitment of the splicing machinery? As a splicing expert, it surprises me…

Response to R1P3:

In general, we carefully designed all EXSISERS constructs on the nucleotide level: we did use not only optimal mammalian codons but also avoided stable RNA secondary structures, and removed potential cryptic splice sites that may cause problems. We now included references to the software packages (Human Splice Finder v3.1 and NetGene2) in the Methods section under "Generation of stable EXSISERS cell lines with CRISPR/Cas9").

Regarding the insertion site, we emphasize the technical requirement for a Cys, Thr, or Ser in downstream of the insertion site (Ser and Thr are commonly found in regions containing loops and flexible linker amino acids). Furthermore, we paid attention to not modify any potential exonic splice enhancers and silencers/suppressors. For *MAPT* exon 10, there are 5 exonic splice modulators (doi: 10.1186/1750-1326-3-8), which were left intact upon insertion (see **Supplementary Fig. 9** for the insertion site of EXSISERS). We have also added a note to the method section that the insertion should be placed as distal as possible from exon-intron junctions to prevent undesired effects on RNA-splicing.

We also included data from an alternative insertion site (IS) of the alternatively spliced exon 10, which lies two amino acids (6 nt) downstream to the first IS. Again we took care not to disrupt known or potential splice enhancer/silencer motifs. The corresponding immunoblot did not reveal any obvious changes upon EXSISERS insertion at the 2nd site compared to unmodified HEK293T cells (**Supplementary Fig. 9**).

With respect to exon size, it has been suggested that large exon sizes are not a limiting factor in the identification of exons in alternative splicing (doi:10.1128/mcb.14.3.2140), which is in line with our experimental data. The prerequisite was that the inserted coding sequence did not contain any potential cryptic splice sites inducing aberrant splicing. In contrast, it has been suggested that the intron length has a major influence on alternative splicing, such as in the case of CD44 (doi:10.1128/mcb.18.10.5930).

We also designed our sgRNA in a way that the insertion of EXSISERS is sufficient to prevent Cas9 recutting, such that 'silent' synonymous codon substitutions are avoided, which can have unwanted side-effects as reported by Xiang *et al.* (10.1186/s13024-018-0280-6).

R1P4 and R1P5:

4) In Fig.2e, why there are equal levels of NLuc and FLuc in WT induced cells? If the exon is not included, NLuc should be lower than FLuc, right? Then with the use of 5-iodotubercidin,

which induces e10 inclusion, in suppl Fig.6 there is increase of both 4R (+ex10) and 3R(-ex10) isoforms. How come? 3R should not increase…
5) Are the two splicing intein proteins equally efficient splicing out the Luc proteins (Gp41-1 and NrdJ-1)? Maybe Suppl Fig 5 was intended to study this, but I don't understand the results. Looks like for each NLuc signal there are 30 of FLuc, which makes FLuc more efficiently spliced. Was this corrected in the main figures? It is kind of important since usually we look at the relative levels of the alternatively spliced isoform vs total protein. If one intein is more efficient than the other, it will affect interpretation of results. Also, can inteins splice out all the mRNAs translated? In a screening, can inteins be inhibited leading to indirect effects (no blasticidin not because there is no exon inclusion, but intein is inhibited or translation inhibited)?

Response to R1P4 and R1P5:
To adjust for the difference in the signal from FLuc and NLuc (due to differences in translation, half-life-time, enzyme activity, and brightness of the substrates), we expressed 0N4R-isoform from EXSISERS$_{MAPT:10NLuc-11FLuc}$ in which the two luciferases are driven at 1:1 stoichiometry by a Pgk1 promoter.
By transfecting increasing amounts of this plasmid, we established a linear relationship between the relative luminescence signals from FLuc and NLuc and determined that for our experimental settings, 30 RLUs of FLuc correspond to 1 RLU of NLuc, i.e., NLuc is 30-fold brighter than FLuc (original **Supplementary Figure 5b**, now **Supplementary Figure 2c**). As can be seen in the immunoblot (**Supplementary Fig. 2b**), this factor is not due to a difference in splice efficiency but rather a difference in substrate-dependent turnover rate and substrate/detection sensitivity.
In the new **Supplementary Fig. 5c**, we used this factor to adjust for the relative brightness and calculated the fraction of exon 10 inclusion to be ~5% in HEK293T-derived cells, in accordance with tau immunoblots (**Supplementary Fig. 5b**). The IVS10+16 c>t mutation led to a ~4-fold increase in exon 10 inclusion in the luciferase-based readout (**Supplemental Fig. 5c**), which matched the 3.7-fold increase, determined by immunoblot (**Supplemental Fig. 5b**).

Since the experiments of **Figure 3** are designed to show differential effects of pharmacological and genetic modulation of isoform expression, we have normalized all NLuc/FLuc data from EXSISERS$_{MAPT:10NLuc-11FLuc}$ to the control/baseline condition (induced *MAPT* but w/o perturbation), such that absolute differences in brightness are compensated, and differences due to the experimental perturbation can be directly read off the graphs.
We explained this normalization procedure in the figure legend, in the methods and statistics section.
Please also see our answers to R4PC2 and R4PC3.

With respect to your comment on the original Supplementary Fig. 6 (now embedded as **Supplementary Fig. 11a**), we thank the Reviewer for pointing out the inconsistency; indeed, the caption for this figure was mistakenly set. The caption was shifted by one position to the left; the legend has been corrected, and a new immunoblot has been inserted in the same as subfigure **b** with a finer titration of ITU. We are very sorry about this mistake and replaced the figure with a corrected version. Also, a similar immunoblot in direct comparison with EXSISERS$_{MAPT:10NLuc-11FLuc}$ has been inserted as new **Supplementary Fig. 12**.

R1P6:

6) Taking into consideration that the RNA is affected by using Cas13. It is important to show that the « protein » splicing effects observed with the inteins are also true at the RNA level by qRT-PCRs. e10 and total MAPT RNA levels should be affected accordingly in Fig.3. It is an important control.

Response to R1P6:

We have now performed RT-qPCRs experiments to validate all Cas13 key results of **Fig. 3** at the RNA level, *i.e.*,

a) Cas13d-NLS with an extended spacer is outperforming Cas13d-NLS with the originally published 22 nt spacer regarding general perturbation efficiency.

b) When Cas13d is applied in the nucleus using an isoform-specific spacer, it will still lead to a knock-down (KD) of all isoforms.

c) Cas13d applied on exon-junctions is more specific towards an isoform since it can only bind to the post-RNA-splicing mature mRNA.

d) shRNA is at least comparable if not superior to CRISPR/Cas13d or b, given that the latest miRNA scaffolds and the latest design rules are deployed. It also does not require the co-expression of two components (crRNA and Cas13).

R1P7:

7) In Fig.3c, why crRNA 10-11 is not affecting total MAPT levels but 9-10 is ? More puzzling, why the use of shRNAs to mimic miRNAs pathway has the opposite effect, it is the 9-10 that is more isoform specific than 10-11 ?

Response to R1P7:

We thank the Reviewer for this question regarding the details of **Fig. 3**.

In **Fig. 3c**, crRNA targeting exon 10-11 is clearly knocking-down 4R tau (NLuc) but seemingly not pan tau (FLuc). The reason is that the true fractional expression of 4R tau is very low (around 3-5%, please see R1P4 and R1P5 for details) compared to 3R tau (only very mature primary neurons in a complex 3D culture model are expressing a significant level of 4R tau (doi:10.1016/j.scr.2019.101541), thus even a 100% knock-down (KD) of 4R tau would just lead to an insignificant KD of pan tau.

The [2]9-10 crRNA is asymmetrically positioned on the 9-10 junction (=4R, **Fig. 3d**) and thus also matched almost perfectly on the 9-11 junction (3R, **Supplementary Fig. 16**) with only a single-nucleotide terminal mismatch (Cas13 systems tolerate single-nucleotide mismatches) resulting in the KD of all isoforms. For the 3rd generation shRNAs, the 9-10 microRNA (miR) was symmetrically positioned on the 9-10 junction (4R, **Fig. 3d**) and thus was specific for only 4R-tau since; an alignment of the 9-10 miR on the potential matching 9-11 junction (3R, **Supplementary Fig. 16**) showed 3 mismatches in the 5'-seed region (position 2–7) and thus was not activating the RNA-induced silencing complex (RISC) when accidentally bound to 3R.

In contrast, the 9-10 junction targeting miR was asymmetrically positioned onto the 9-10 junction (4R, **Fig. 3d**) due to design constraints of microRNAs and thus was also matching perfectly with its 5'-seed region (position 2–7) onto the 9-11 junction (3R, **Supplementary Fig. 16)** with only mismatches in its 3'-end that is tolerant towards mispairings.

Expectedly, the KD of 3R tau (crRNA targeting 9-11 junction) led to a clear decrease of pan tau signal (FLuc) without changing the 4R tau level (NLuc) in main **Fig. 3c**. This also has been confirmed in RT-qPCR in unmodified 293T cells in the new **Supplementary Fig. 14b**.
In summary, a strong depletion of pan tau (FLuc) in this cell line while trying knocking down 4R tau is clearly a side effect of lack of isoform specificity that can be observed for the exon 10 targeting crRNA and for the asymmetrical 9-10 junction targeting crRNA ([2]9-10), while the crRNA targeting the 9-10 junction symmetrically ([1]9-10) and the 10-11 junction are more specific.

R1P8:

8) Fig3f, dCasRX-SR effect is just 1,6x-fold. I don't think this is going to be biologically meaningful. The control in which there is dCasRx-SR or dCasRX-hnRNPA1 but not crRNA is missing (to make sure there are no indirect effects).

Response to R1P8:

The main objective of **Figure 3** is to show how EXSISERS technology can be used to optimize programmable effectors at the RNA level for modulating isoform-specific expression. We found a strong effect of the length of the guide RNA and the localization of the Cas13-effectors, while amiRNA was also very competitive.
To complete the picture, we also added data on the use of dead Cas13 systems for splicing modulation, because it is an application that is not possible with amiRNA.

We have now replicated the results on two independent clones, including the requested non-targeting controls (NTC) on another WT clone and also a clone carrying the IVS10+16 c>t mutation (**Supplementary Fig. 17**).
These results show that also small changes in isoform-specific expression can be quantified reliably with EXSISERS.
We did not express any opinion on whether the observed effects are biologically meaningful but simply suggest that EXSISERS can help to characterize and optimize systems that alter isoform-specific expression.

R1P9:

9) Again, the effect on Suppl Fig 12 seems very low too, 1,5x-fold. Is this sufficient to claim what the authors claim?

Response to R1P9:

We applied EXSISERS on a ribosomal-frameshifting-regulated gene to show EXSISERS' unique capability to monitor co-translational regulations, where RT-qPCR would fail. However, we did not claim a new finding. The observed effects are concentration-dependent and were independently confirmed with two complementary methods (fluorescence-activated cell scanning (FACS) and immunoblot analysis).

R1P10:

10) Why are the IFs in Fig2c and Supplementary Figure 11d,f so dotted at the nuclear level? Is this related to the reporter?

Response to R1P10:

Given that also unmodified HEK293T cells showed the 'nuclear dots' (new **Supplementary Fig. 5a**), they are likely a result of some unspecific binding of the pan-tau antibody (TAU-1 alias PC1C6) to nucleolar proteins in our immunofluorescence staining protocols.

R1P11:

11) For Fig.4, the CRSPR screening, it is important to know how many clones resisted to the blasticidin to know the false-positive rate of the system. The authors only show the positive MBNL1 clone, but this was already well known. Was the finding straightforward? It does not invalidate the proof-of-concept but it can give perspective on the feasibility of the system. It is known that some cells can escape the blasticidin selection. Were the authors using a higher amount of antibiotic than what is used for clone selection (1-10 ug/mL depending on the cell type)?

Response to R1P11:

We performed the experiment with a theoretical ~400-fold coverage of every sgRNA. The library contained ~80,000 sgRNAs against ~20,000 coding genes, including non-targeting control sgRNAs, resulting in 4 sgRNAs per gene. To achieve a ~400-fold coverage, we infected 100×10^6 cells with the lentiviral library with a multiplicity of infection (MOI) of ~0.3.
At least several hundred clones survived the most-stringent blasticidin selection condition (5 µg/ml). NGS analysis revealed that in this condition, 28.4% of the clones contained aN *MBNL1*-targeting lentiviral vector (composed of 18.8% and 9.6% of two different sgRNAs targeting *MBNL1*). Under low-pressure selection with the minimal inhibitory concentration of 3 µg/ml blasticidin-S, the flasks were confluent after the same timeframe. Still, based on the NGS analysis, 1.4% of the confluent population contained a lentivirus with a sgRNA targeting MBNL1. Also, based on NGS, only 0.0001% of the unselected control condition contained the same sgRNAs targeting MBNL1. This results in a 4 magnitudes of fractional enrichment in the 3 µg/ml blasticidin S condition and >5 magnitudes fractional enrichment for the more stringent 5 µg/ml blasticidin S condition. In other words, by simply subcloning the PCR product (instead of NGS) of the integrated lentiviral sgRNA expression cassette of the most stringent condition (5 µg/ml), followed by a standard Sanger sequencing of at least 20 clones, one would already expect 5-6 bacterial clones containing an *MBNL1*-targeting sgRNA. We emphasize that two independent sgRNAs targeting MBNL1 were independently

enriched by 3 magnitudes (3 µg/ml blasticidin-S) and 4 magnitudes (5 µg/mL blasticidin-S) over the median sgRNA population. Importantly, we validated the screen on a different $\text{EXSISERS}_{\textit{FOXP1}\text{:18b-BSD}}$ clone using a 3rd independent sgRNA (different from the two enriched *MBNL1*-targeting sgRNAs of the library) targeting a constitutive *MBNL1* coding exon in parallel with a sgRNA targeting *MBNL2,* followed by blasticidin-S selection. Only the condition targeting *MBNL* genes led to blasticidin-S-resistant cells but targeting the control *AAVS1* locus did not. Moreover, when analyzing the surviving population via sequence decomposition of Sanger sequencing results, a dose-dependent accumulation of mutations in MBNL1 with increasing blasticidin-S concentration was indicative of functional coupling of the MBNL1-FOXP1-18b-Bsd-axis. With WT cells expectedly, we could not detect any resistant cells independently of any selection conditions and independently of the gene that was targeted. As described in **Fig. 4**, we used blasticidin-S in a concentration range the Reviewer indicated (3 µg/ml and 5 µg/ml are exactly in the range of 1-10 µg/ml).

Reviewer #2 (Remarks to the Author):

R2P0:

In this manuscript, the authors developed a new type of cell-based reporter system, exon-specific isoform expression reporter system (EXSISERS), which enables non-invasive detection of alternative splicing and exon-specific translation via intein-mediated protein splicing. They construct generated dual-luciferase (Nluc and Fluc) EXSISERS lines for ratiometric monitoring of different Tau protein isoforms, 3R-tau and 4R-tau. As designed, the system can recapitulate the expected change of different tau protein isoforms. The application of this reporter system was further demonstrated in several scenarios: 1. Screening of the effective guide RNAs in CRISPR/Cas-13 system that can achieve isoform-specific gene silencing; 2. Testing the activity of designer splicing enhancer or suppressor using the dCas-13 fusion protein containing SR domain or Gly-rich domain; 3. Measuring the co-translation ribosomal frameshift regulation. Finally, they generated an EXSISERS reporter for alternative splicing of exon 18b in FOXP1 and use the reporter to identify the regulators for isoform-specific expression of this exon via genome-wide CRISPR/Cas9 screen. Given their results the authors propose that it will be possible for an unbiased and non-invasive functional screening for splice modulators.

Overall I find the approaches employed in this study is valuable for characterizing and manipulating the intrinsic functionality of the exon-specific protein isoforms. However, the system is cumbersome to use and require a large amount of time for consecutive steps of CRISPR-cas insertion, which will limit its usefulness. In addition, some of the application did not perform as efficiently as previous system that was much simpler to generate. For example, the designer splicing enhancer and silencer using aCas-13 in EXSISERS reporter (Fig. 3f and 3g) was not as efficient as the engineered splicing factors using PUF fusion proteins (Wang Y et al, 2009 Nature Method, Wang Y et al 2013 NSMB), which is much simpler system to use. The authors should acknowledge such limitation and compare their system with previous system.

Response to R2P0:

We thank the Reviewer for acknowledging the value of EXSISERS to assess exon-specific protein isoform expression.

As we show in **Table R1**, EXSISERS has a unique set of advantages over other methods.

	EXSISERS	minigenes	immunoblot	immuno-cytochemistry	RT-qPCR	RNA-FISH
at endogenous site	☑		☑	☑	☑	☑
protein-level readout	☑		☑	☑		
cellular resolution	☑	☑		☑		☑
coupling of effectors to exon inclusion	☑	☑				
repeated measures	☑	☑				
no cell line needed			☑	☑	☑	☑

Table R1| Advantages of EXSISERS over alternative methods to detect isoform-specific expression

Although it is required to generate stable EXSISERS cell lines to ensure that isoform-specific expression is monitored at physiological levels, it is **not more cumbersome to generate those lines than it is to generate adequate minigenes**. Minigenes also have to be integrated into the genome to not unphysiologically overload the splicing/expression machinery, which will lead to aberrant alternative splicing behavior, as reported for, *e.g.*, MAPT.
Please see a comparative analysis of two minigene systems for *MAPT* in our response to R3P2.

To ensure maximal convenience in producing EXSISERS lines, we have streamlined the process such that only a **single cloning step** is necessary to generate the all-in-one CRISPR/Cas9 plasmid and the targeting plasmid, that can be inserted into the genome within **2-3 days** (please see **Supplementary Fig. 3**, previous Supplementary Fig. 2). The CRISPR/Cas9-mediated insertion is sufficiently efficient with the plasmids we provide, such that within just **2 months**, clonal EXSISERS cell lines can be generated (**Supplementary Fig. 4**).
With respect to efficiencies using CRISPR-Cas9-mediated insertions, please see the detailed response to R1P1 for targeting efficiencies of EXSISERS.

With respect to Pumilio/PUF-based splicing modulators, we agree that they are powerful and we, therefore, had already cited Wang, Y., Cheong, C., Tanaka Hall, T. *et al*. "Engineering splicing factors with designed specificities." Nat Methods 6, 825–830 (2009), doi:10.1038/nmeth.1379 in our original submission.
Since Cas13-based splice modulators are still currently of broad interest, chose this system to show that EXSISERS can be used to optimize it, but the same is, of course, goes for Pumilio/PUF-based splicing modulators.

R2P1:

Specific concerns:

1. The intein used in this study were shown to have high splicing efficiency (Supplementary Fig. 1) in their system, however I am curious about how efficiently the intein works in different cell lines. Additional quantification should be performed to measure the intein excision rather than assuming it is always 100% excised.

Response to R2P1:

Inteins have indeed been shown to be effective upon heterologous expression in several mammalian cell types *in vitro* and *in vivo*. Most importantly, applications in mammals, such as splitting Cas9 to circumvent the limited packaging capacity of recombinant adeno-associated viruses (rAAVs), a commonly used viral vehicle for gene therapy, by harnessing trans-splicing inteins ('protein ligation' of two co-expressed polypeptides), were effective *in vivo* in pig and mouse models (doi:10.1038/s41591-019-0738-2, doi:10.1038/s41551-019-0501-5).
We have further improved the high splicing efficiency of the fast-splicing inteins (doi:10.1074/jbc.M112.372680) by adding coiled-coils (CCs) to support cooperative folding of the cis-splicing intein halves and its excision. We updated **Supplementary Fig. 1** with data for which we used mNeonGreen as extein as it is known to fold extremely rapidly in much less than 10 minutes. Thus, we reasoned that this extreme case of a fast-folding extein should be maximally sensitive to detect any unproductive folding intermediates.

Under these circumstances, the CCs-enhanced intein resulted in a higher product/educt-ratio compared to the CCs-less counterpart. C-cleavage side products could only be detected upon overexposure and contrast enhancement. We did not detect any N-cleavage products.

Upon request of the Reviewer, we have now included full immunoblots from multiple clones showing essentially no unspliced products for tau. Only under extreme overexposure, weak bands appear at densities of less than <1% of the spliced products, which most likely correspond to the *de novo* translated proteins (**Supplementary Fig. 7**). Even for minigene-versions of EXSISERS$_{MAPT:10NLuc\text{-}11FLuc}$, which are heavily overexpressed at unphysiological levels, we could barely detect any unspliced educt (**Supplementary Fig. 10c,d**).

In addition to our experiments with HEK293T cells, we have observed similar results from murine neuroblastoma cells (N2a) in which housekeeping gene (*Tubb3*) was intact (**Supplementary Fig. 18m**). Here, too, no unspliced educts could be detected.

R2P2:

2. In Fig.2, since the study is focusing on the exon-specific isoforms of tau protein, the authors should use an exon10 specific tau antibody (or pan antibody for tau) to calibrate the system. This is to make sure that the results obtained from luciferase measurement correlate well with direct measurement of tau isoforms.

Response to R2P2:

Reliable tau-specific antibodies are hard to get by. Still, we had screened several anti-tau antibodies and found that the best way to reliably identify 4R tau is by comparing a 3R-immunoblot to pan-tau immunoblots. We proved that this band is indeed the 4R band in **Supplemental Fig. 11a).** However, the S/N-ratio of this 4R-antibody (doi:10.1186/s13024-017-0229-1) is low, and we also needed to see the fractional inclusion of 4R from total tau. Thus, the anti-pan-tau antibody was the most informative tool for our requirements.

When WT HEK293T cells were treated with ITU known to increase 4R tau (doi:10.1111/febs.12411), the 0N4R band (2nd band from below in anti-pan-tau immunoblot, **Supplementary Fig. 11**) was clearly increasing while 0N3R was decreasing (1st band from below in the anti-pan-tau immunoblot, **Supplementary Fig. 11**). Similarly, the bioluminescent signal from EXSISERS$_{MAPT:10NLuc\text{-}11FLuc}$ increased by ~4-fold (**Fig. 2f, j)** and longitudinally over a period of 60 hours in **Fig. 2h**.

In a direct comparison from unmodified HEK293T cells and EXSISERS$_{MAPT:10NLuc\text{-}11FLuc}$ in the same immunoblot, increasing ITU concentration resulted in a fractional increase of 4R tau. In contrast, the total tau level decreased slightly (new **Supplementary Fig. 12**). As expected for EXSISERS$_{MAPT:10NLuc\text{-}11FLuc}$, the OLLAS-positive band for excised NLuc (=4R) was getting more prominent with increasing ITU concentrations (**Supplementary Fig. 12**).

Furthermore, **Fig. 2d** showed that the IVS10+16 c>t mutation caused an ~3–4-fold increase of 4R-tau in both, immunoblot and in luciferase signal (**Fig. 2d,e and h, and Supplementary Fig. 5,6, and 7**). Please note that although the size separation and spatial resolution of the tau bands is high compared to typical anti-tau immunoblots in the literature (doi:10.1186/s13024-017-0229-1, doi:10.3892/ijmm.2012.1025), precise quantification of tau isoforms by densitometry is extremely challenging.

R2P3:

3. In Fig.4, I feel that this part lacks an important analysis on transcriptome level for the MBNL1/2-KO cells and the exon 18b inclusion cells after blasticidin selection. MBNL1/2 are key regulator in RNA splicing, and knock-out of these two genes should cause significant change of splicing in the level of entire transcriptome. I am wondering whether knock-out of these two genes could cause more exon-specific protein changes besides FOXP1.

Response to R2P3:

We agree with the Reviewer that it is interesting to ask which impact perturbations of MBNL proteins have on the transcriptome.

In our manuscript, however, it was the goal to present EXSISERS as a screening tool for unbiased identification of splicing modulators. Indeed, without any prior knowledge, we re-identified MBNL1 as the main regulator of *FOXP1* exon 18b inclusion using an unbiased lentiviral CRISPR/Cas9 screen, which was impossible before. We then followed up with a knockout of independent sgRNA targeting *MBNL1* to validate the results in our system.

With respect to the effects of MBNL on the transcriptome, we would like to refer to the thorough work of Han *et al.*, 2013 (doi:10.1038/nature12270), where they use RNA-seq profiling to analyze the impact of MBNL perturbations mediated by siRNAs. They showed that MBNL proteins negatively influence the global AS network important for pluripotency maintenance, partially by repressing the ES-cell-specific FOXP1 isoform, a stimulator of a core pluripotency circuit, thus promoting transcriptome-wide switch towards differentiation.

R2P4:

4. I think this paper may present a powerful tool to track and study exon-specific protein isoform. However, the authors should use it to investigate on new biological questions rather than only to confirm the conclusion people have already made.

Response to R2P4:

We thank the Reviewer for sharing enthusiasm towards EXSISERS as a 'powerful tool' to investigate alternatively spliced protein isoforms. While the main weight of such a methodological paper must clearly lie on the careful validation of the new instrument on the various technical levels against well-established results, we have made a few interesting observations showing the robustness and convenience of EXSISERS technology:

We showed for the first time,

a) the longitudinal readout of isoform-specific expression with cellular resolution of an alternatively spliced exon from the original genomic site in living cells,
b) an improved targeting efficiency of Cas13d significantly by the extension of the spacer length from 22 nt to 30 nt,
c) the importance to optimize the precise site of action for each programmable intervention tool (Cas13d or b, or shRNA in the cytosol) since it has a massive impact on the isoform specificity, even if the same position is targeted,
d) that shRNA - if carefully designed using the latest design rules and using up-to-date pri-microRNA biogenesis-mimicking scaffolds - can compete with Cas13-based systems regarding potency and isoform-specificity,
e) an independent confirmation of a serendipitous scientific finding of *FOXP1* exon 18b regulation via MBNL1 using a novel unbiased approach.

These examples lay out precise recipes for biological discoveries and there are already several laboratories in our network that are actively using EXSISERS technology to test their preferred biological hypothesis.

R2P5 (Minor P1):

Minor concern:

Overall the figures are poorly prepared with low resolution and confusing color scheme, more specifically:

1. The picture quality of Fig.2c and Fig.2g should be improved. The color and style of this figure should be modified to make it more reader friendly. In addition, Fig.2c and 2g should be showed in color to help understand.

Response to R2P5 (Minor P1):

We apologize that the quality of our figures was apparently compromised during compression. We are sorry for the compression artifacts of **Fig. 2** that occurred in the last submission. All our original figures are high quality.

R2P6 (Minor P2):

2. The picture quality of Fig.4c and Fig.4d should be improved. And the part (Identification of regulators for isoform-specific expression) and Fig.4 need be carefully reviewed, because the figure and the main text are not consistent.

Response to R2P6 (Minor P2):

We are sorry for the compression artifacts of **Fig. 4** that we improved. Furthermore, we thank the Reviewer for pointing out the disparity between main text and **Fig. 4**, we carefully re-read the main text and corrected inconsistencies with the figure.

R2P7 (Minor P3):

3. Supplementary Fig.8b need to be updated, as the resolution is very low.

Response to R2P7 (Minor P3):

We are sorry for the low quality of the original **Supplementary Fig. 8** (now improved in **Supplementary Fig. 19**). Regarding subfigure **b**, the GFP channel **did not show any signal** since in contrast to luciferases, endogenous expression of 4R tau did not yield enough protein to be readily detected in a common epi-fluorescence microscope.

R2P8 (Minor P4):

4. Similar to Fig. 2c, the supplementary Fig.11 and Fig.13 should be improved.

Response to R2P8 (Minor P4):

We improved the quality of the respective figures.

Reviewer #3 (Remarks to the Author):

R3P1:

Truong et al. develop a minimally invasive isoform-specific expression reporter system (EXSISERS) that incorporates translated and subsequently excised fast-splicing inteins with CC-domains into genes of interest. The authors demonstrate the utility of EXSISERS in a number of applications, ranging from the optimization of RNA-targeting strategies for exon-specific RNA degradation of MAPT mRNA, to the quantification of ribosomal frameshift-mediated regulations unmeasurable by RT-qPCR, to a phenotypic readout for a high-throughput screen of FOXP1 exon 18b inclusion that validates existing literature. Altogether, the presented work is a valuable addition to the isoform-specific RNA monitoring toolkit. While the generation of EXSISERS may be an involved process, nevertheless for some applications it might prove more useful than alternative methodologies, such as minigenes. I have a few major criticisms.

Response to R3P1

We thank the Reviewer for acknowledging the value of EXSISERS for monitoring isoform-specific expression. We have compiled **Table R1**, to compare the features of EXSISERS as compared with other relevant methods for detecting isoform-specific expression.

	EXSISERS	minigenes	immunoblot	immuno-cytochemistry	RT-qPCR	RNA-FISH
at endogenous site	☑		☑	☑	☑	☑
protein-level readout	☑		☑	☑		
cellular resolution	☑	☑		☑		☑
coupling of effectors to exon inclusion	☑	☑				
repeated measures	☑	☑				
no cell line needed			☑	☑	☑	☑

Table R1 | Advantages of EXSISERS over other methods to detect isoform-specific expression

Although many important findings were made possible by minigenes, they may (**1**) suffer from untruthful readout, (**2**) cause alterations of endogenous splicing, while (**3**) still requiring the same effort on cloning and generation of stable cell lines.

(1) Minigenes may lead to <u>untruthful readout of endogenous splice-regulation of a gene of interest</u> because they - with a high probability - do not contain all relevant regulatory elements. This is especially true for tau, where it has been shown that basically the whole intronic region is required to reflect the true splicing behavior for exon 10 (doi:10.1111/j.1471-4159.2004.02477.x). Most importantly, it has been shown recently that many identified SNPs have their origin deeply embedded within introns, such as the rs242561 polymorphism, that is protective against Parkinsonian disorders (doi:10.1016/j.celrep.2016.03.068). This single nucleotide polymorphism is located within the first intron 13.2 kbp upstream of the 2nd coding exon and 55 kbp downstream of the first coding exon; the same is true for rs242557 which is also associated Parkinson's disease,

which is located 48 and 20 kbp down- and upstream from the flanking exons (doi:10.1016/j.neulet.2010.10.015, doi:10.18632/oncotarget.16490) or rs2471738 that lies 11.6 kbp upstream of the alternatively spliced exon 10 and 2 kbp downstream of exon 9 (doi:10.18632/oncotarget.16490). Moreover, many vertebrate genes are recursively spliced which will not be recapitulated by minigenes (doi:10.1038/nature14466). Also, for other alternatively spliced genes such as CD44, the intron's length determines the inclusion efficiency of the alternatively spliced exon (doi:10.1128/mcb.18.10.5930). A mini-gene version that contains truncated introns would therefore inevitably lead to unphysiological splicing. Thus, it is essentially impossible to faithfully recapitulate the complex regulatory machinery outside the precise three-dimensional context of the endogenous sites.

(2) Minigenes are not applicable to unbiased screens for splice regulators (such as genome-wide CRISPR/Cas9 KO-screens) to enrich a certain population of cells with a defined genetic perturbation. Minigenes are normally used in a transient transfection assay and even if integrated into the genome, they lay outside of the endogenous site and are driven by constitutive promoters. They are, therefore, hiding effects of (co)-transcriptional regulations. Also, the truncated introns cannot reflect the physiological genomic context such that whole-genome screens would probably yield questionable results.

(3) Minigenes can **cause alterations of endogenous splicing of other collateral genes** by competitive binding of splicing factors to the constitutively overexpressed minigene. This results in depletion from endogenous sites. In the case of *MAPT*, the altered isoform ratios can even feed-back on the splicing process since the formation of aggregated neurofibrillary tangles leads to the co-depletion of the otherwise soluble spliceosomal components further increasing the aberrant change of the global cellular splicing pattern (doi:10.1016/j.celrep.2019.08.104).

(4) Minigenes require the same effort to establish as EXSISERS
We made sure that the production of the EXSISERS lines is as convenient as possible: we provide all EXSISERS reporters in a respective cloning vector, such that only a single cloning step is required to obtain a customized exon-specific EXSISERS vector (please see **Supplementary Fig. 3**). The CRISPR/Cas9 vector, improved with enhanced gene targeting efficiency, can also be cloned in a **single** step (please see **Supplementary Fig. 3**). Please also see our graphical abstract of the process (**Supplementary Fig. 4**), which shows hot an EXSISERS clonal cell line can be established in just ~4–6 weeks. Please also see our response R1P1.

With respect to the effort for making the respective cell lines, minigenes also require the assembly of different fragments of truncated exon-intron fragments and subsequent cloning into a mammalian expression vector. Usually, several minigene versions with different truncations need to be tested, since truncations can lead to the removal of essential regulatory sequences, which are important for the regulation of alternative splicing.

Furthermore, minigene systems that are not read out via RT-qPCR but via a reporter system - which is essential for high-throughput detection - require additional modifications in the alternatively spliced exons to include stop or start codons for fluorescent proteins or luciferases. Alternatively, a frameshift-based reporter to distinguish the ab- or presence of an exon can be used. This, however, requires also a deletion/insertion of 1 or 2 nucleotides,

since normally an alternatively spliced exon contains a number of nucleotides divisible by 3 (Stoilov *et al.* (doi:10.1073/pnas.0801661105), Luo *et al.* (doi:10.1002/cbic.201402069)).

Also, random integration of the minigene into the genome introduces an unnecessary variability due to copy number variation, impact on neighboring genes, expression strength, and splicing behavior (doi:10.1016/j.cell.2010.11.056). Additionally, screening compound libraries to alternate AS, library-scale minigene transfection for every condition would not be economically feasible.
In summary, also for minigenes it is recommended to knock-in into a well-defined safe-harbor locus (such as *AAVS1/PPP1R12C* in human and *Rosa26* locus in murine systems) using CRISPR/Cas9 (or TALENs, ZFNs) to minimize variability.

Please also see our detailed response to your request in R3P2 where we also carefully compared minigenes with EXSISERS.

R3P2:
Major points:
1. The authors do not perform any head-to-head comparisons of EXSISERS to minigenes, which are comparatively much simpler and faster to generate. This should be done. If there is no clear advantage of EXSISERS, then it is worth wondering whether other researchers will adopt the new methodology.

Response to R3P2
Thank you also for the constructive suggestion to perform a head-to-head comparison with minigenes.
To this end, we have carefully studied the elaborate minigene systems for *MAPT* by Yu *et al*., (doi:10.1111/j.1471-4159.2004.02477.x) and Jiang *et al.* (10.1128/mcb.20.11.4036-4048.2000 to construct corresponding minigene systems.

Before we compare our results shown in **Supplementary Fig. 10**, we need to quickly review the pertinent findings from Yu *et al*., which is a very careful study that, however, also demonstrates the complexity and potential pitfalls for obtaining truthful results with minigenes.
It can be seen from **Figures 1 and 2 in Yu *et al.*** (attached below with figure legend) that the authors laboriously tried out 10 different tau-4R minigenes with different intronic truncations but found that none of them showed physiological splicing behavior. Only a plasmid made from a construct with **full-length** introns of 17,485 bp (LI9/LI10) recapitulates the endogenous physiological ratio. Similar behavior for minigenes also could be observed by Jiang *et al*. (Fig. 2B vs. Fig. 2A, doi:10.1128/mcb.20.11.4036-4048.2000). Besides, using full-length introns in minigenes is technically very difficult, since those introns can easily reach 5-digit bp in length and thus require specialized PCR-protocols to be amplified. Equipped with a plasmid backbone of ~3 kbp, promoter elements, and the rest of the tau coding sequence, this plasmid would also easily exceed the 20 kbp limit for classic plasmid transfection (doi:10.1093/nar/27.19.3792, doi:10.1016/j.ab.2005.08.029). Also, for plasmids greater than 20 kbp, the increased risks of plasmid instabilities enforce the usage of bacterial artificial chromosomes (BAC) instead.
Aberrant splice behavior of minigene systems has also been reported for other genes than *MAPT*. For the *ABCA4* gene (128 kbp, 50 exons), which plays a role in the Stargardt disease, Sangermano *et al.* (doi:10.1101/gr.226621.117) *'[...] discovered that when using*

small minigenes lacking the proper genomic context, in vitro results do not correlate with splice defects observed in patient cells.' They *'[...] therefore devised a novel strategy in which a bacterial artificial chromosome was employed to generate midigenes, splice vectors of varying lengths (up to 11.7 kb)* ***covering almost the entire ABCA4 gene.'*** Only under these circumstances, a similar splicing behavior as observed in patients could be recapitulated.

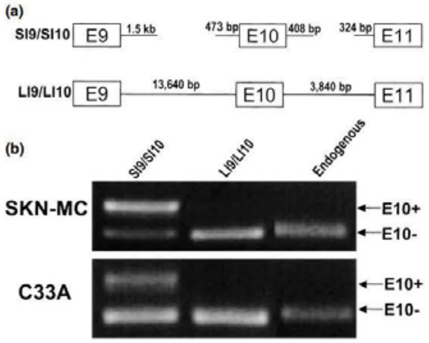

Fig. 1 Introns 9 and 10 affect splicing patterns of exon 10 in the *tau* gene. (a) Mini-gene constructs for splicing of exon 10 in the *tau* gene were generated in PCI-neo vector. The short previously published mini-gene SI9/SI10 includes exon 9, the first 1.5 kb and the last 473 bp of intron 9, exon 10, the first 408 bp and the last 324 bp of intron 10, and exon 11. The long mini-gene construct LI9/LI10 contains full length of both intron 9 and intron 10. (b) Mini-gene constructs were transfected into C33a or SKN-MC cells. Splicing patterns of exon 10 in mini-genes were examined by using RT–PCR. Splicing of exon 10 from the endogenous *tau* gene was detected in C33a cells or SKN-MC cells induced by 10 μM of sodium butyrate for 24 h.

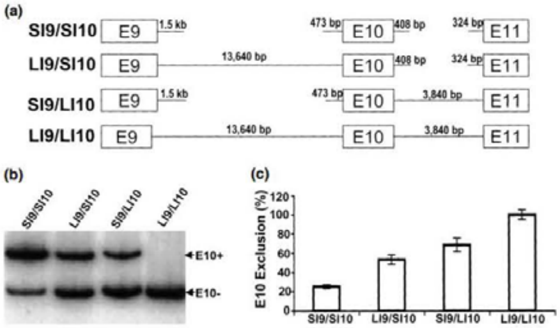

Fig. 2 Intron 9 and intron 10 additively contribute to correct splicing of exon 10 in the *tau* gene. (a) Constructs with a full-length intron 9 and a short intron 10 (LI9/SI10) or with a full-length intron 10 and a short intron 9 (SI9/LI10) were generated. The short intron 9 or short intron 10 was identical to that in SI9/SI10. (b) The constructs were transfected into SKN-MC cells. RT–PCR was used to determine splicing patterns of exon 10. (c) RT–PCR bands were quantitated using a phosphorImager. Bar represents the mean percentage of mRNA with exon 10 exclusion (E10 –) out of total mRNAs (E10 + and E10 –) from three separate transfection experiments. Error bars represent standard deviations of the means.

These results suggest that intronic truncations, an essential characteristic of minigenes, can be misleading, even if the minigene contained several hundred nucleotides of sequences down- and upstream of an exon of interest. Zheng *et al.* (doi:10.1101/gr.147546.112) also warned that *'[...] minigene reporters do not always recapitulate the regulation of endogenous*

exons. The minigene may not contain all of the relevant cis-regulatory elements for the test exon.'

Recent reports (doi:10.1038/nature14466) also suggested that vertebrate introns, especially long ones, are often removed stepwise in a process called 'recursive splicing'. Thus, a minigene with truncated introns would inevitably lead to an altered RNA splicing behavior. Especially vertebrate introns can be larger than 100 kbp and can hardly be cloned fully in a minigene. Most importantly, those long introns are not just 'junk', which can be replaced by random nucleotide sequences.

For example, Wang *et al*. showed recently that the rs242561 polymorphism is protective against Parkinsonian disorders (doi:10.1016/j.celrep.2016.03.068). This single nucleotide polymorphism is located within the first intron 13.2 kbp upstream of the 2nd coding exon and 55 kbp downstream of the first coding exon; the same is valid for rs242557, which is also associated Parkinson's disease, which is located 48 and 20 kbp down- and upstream from the flanking exons (doi:10.1016/j.neulet.2010.10.015).

A stably integrated minigene is also preferred over transiently transfected plasmids, as Jiang *et al*. (doi:10.1128/mcb.20.11.4036-4048.2000) noted regarding the tau minigenes. They note '*[...] that transfected tau minigenes in these cells produced a slightly higher level of Tau4R compared to the endogenous tau expression pattern (Fig. 2), suggesting that overexpression of the tau minigene may titrate certain limiting factors controlling the ratio of Tau3R to Tau4R*'. Stoilov *et al*. (doi:10.1073/pnas.0801661105) also suggested that minigenes should be stably integrated: '*Note that transient expression of the reporters can lead to significant cell-to-cell variation in the protein signals, which we attribute to differences in the stability of the two proteins and in the amount of DNA taken up by each cell. This **variability is reduced in stable cell lines expressing the reporter** and with reporters where the stability of the two proteins is equalized*'.

Thus, the minigene systems are not easier to create, especially not as a version compatible with high-throughput screenings (*e.g.*, using terminally fused luciferases), which necessitates additional mutations have to be introduced into the coding sequence of the exon of interest.

Based on the luciferase minigene system described by Yu *et al*. (doi:10.1111/j.1471-4159.2004.02477.x), we build a minigene by amplifying the corresponding intronic regions with truncation that are of similar length as in Yu *et al.*, and Jiang *et. al.* (doi:10.1128/mcb.20.11.4036-4048.2000), to create EXSISERS-based 4R-minigenes (**Supplementary Fig. 10a**).

In accordance with Yu *et al.* and Jiang *et al.*, we noted an increased exon 10 inclusion level (~12%, **Supplemental Fig. 10c**) originating from minigenes as compared to the endogenous locus (~3–5%, **Supplemental Fig. 5b**).

For the mutation IVS10+16 c>t, 4R/pan-tau ratio further increased by roughly 2-fold to over 50%. In contrast, with integrated EXSISERS, we did not detect any significant difference between unmodified HEK293T cells, its clones, and EXSISERS$_{MAPT:10NLuc\text{-}11FLuc}$ (**Fig. 2d, Supplementary 5, 6, 7, 8, 9, and 12**).

The reaction of EXSISERS$_{MAPT:10NLuc\text{-}11FLuc}$ in response to small molecule perturbation (**Fig. 2f, h, j, and Supplementary 11 and 12**), and Cas13/microRNA-based modulation (**Fig. 3** *vs.* **Supplementary 14**) was similar to the reaction of unmodified HEK293T cells. Also, the disease-mimicking mutation IVS10+16 c>t lead to the expected 4-fold increase as reported in the literature (doi: 10.1074/jbc.274.21.15134, doi:10.1016/j.molbrainres.2005.02.014).

In summary, the head-to-head comparison of a minigene system and the EXISERS for *MAPT* showed clearly aberrant splicing behavior for the minigene but not EXSISERS as

compared to unmodified cells. These findings are in line with several pieces of pertinent literature reviewed above.

R3P3:

2. The authors use CRISPR-Cas9 to integrate EXSISERS into areas of interest in the genome. When such knock-ins are performed and analyzed, typically researchers will generate multiple clonal cell lines, in case behavior in one cell line may be biased by unique Cas9-induced indel and/or template insertion off-target events. The authors should re-perform the experiments featured in Figures 3 and 4 (and associated supplemental figures) with at least one additional clonal cell line to demonstrate the generalizability of EXSISERS.

Response to R3P3:

We thank the Reviewer for this constructive criticism and agree that clonal lines may show different behavior in particular if SNPs, such as the *MAPT* IVS10+16 c>t mutation, are investigated. We have therefore included immunoblots to show that in all cases, homozygous c>t base transition in this regulatory intronic sequence led to an increase of the 4R/pan-tau inclusion-ratio in additional 9 clones (**Supplementary Fig. 6,7** in addition to the clonal line shown in **Fig. 2d**).

With respect to the experiments of **Fig. 3**, we validated the results regarding Cas13- or microRNA-mediated tau perturbation on unmodified HEK293T cells to exclude that the observed effects are artifacts on the post-translational level or by EXSISERS and performed an RNA-level quantification with RT-qPCR.
Using RT-qPCR, we confirmed that the extended 30 nt spacers are superior compared to the original 22 nt spacer in new **Supplementary Fig. 14a,** and the higher isoform specificity of targeting exon-junctions in new **Supplementary Fig. 14b**.

We also reproduced the minor effects of **Fig. 3f** in two independent $EXSISERS_{MAPT:10NLuc-11FLuc}$ clones (new **Supplementary Fig. 17**). In both clones, the combination of an exon 10 targeting crRNA together with a fusion of dRfxCas13d to the SR-rich domain of SC35 led to an increased 4R/pan-tau ratio. In contrast, the fusion to the Gly-rich domain of $hnRNPA1_{A1B}$ with a splice donor (SD) targeting crRNA decreased it (new **Supplementary Fig. 17**).

With respect to the experiments of **Fig. 4**, the results were already obtained from different clones. The lentiviral CRISPR/Cas9 KO library (**Fig. 4a and b**), as compared to the analyses in **Fig. 4c–f,** where an independent clone was used. We made this explicit into the caption of **Fig. 4.**

R3P4 (Minor P1):

Minor points:

1. The introduction would benefit from a reference to work on minigenes, as they are the main methodological competitor to EXSISERS.

Response to R3P4 (Minor P1):

We thank the Reviewer for this comment. We had already added references on minigenes in the main text in the introduction: '*Established methods for analyzing splicing isoforms either measure mRNA by endpoint-labeling (RT-qPCR, (sm)FISH[4], RNA-sequencing[5]), protein by immunochemistry (immunoblot analysis, immunofluorescence staining), or seek to mimic the genetic regulations via minigene analysis[6–8]*'

R3P5 (Minor P2):

2. The sentence should read "greater reduction": Expression of cytosolic PspCas13b-NES directed against the same region of exon 10 (Fig. 3e, orange bar) resulted in a greater reduction of FLuc as compared with the corresponding RfxCas13d-NLS (p<0.0001, post-hoc tests of one-way ANOVA) with comparable NLuc signal (p>0.05) (Fig. 3e, blue bar).

Response to R3P5 (Minor P2):

We thank the Reviewer for the suggestion, but indeed the knock-down (KD) of FLuc is 'less efficient' (leading to a 'weaker reduction' of FLuc) while NLuc depletion is as efficient as with Cas13d-NLS. We changed the whole sentence to: 'Expression of cytosolic PspCas13b-NES directed against the same region of exon 10 (**Fig. 3e, orange bar**) showed a better 4R-specificity due to decreased NLuc/FLuc-ratio compared with the corresponding RfxCas13d-NLS system (*p*<0.001, post-hoc tests of one-way ANOVA of $10/13d_{NLS}$ *vs.* $10/13b_{NES}$ *vs.* 9-10 amiRNA, **Fig. 3e, blue bar**).'

R3P6 (Minor P3):

3. The sentence should read "4f": Meanwhile, the enrichment of MBNL2 indels showed no dose-dependence (Fig. 4f).

Response to R3P6 (Minor P3):

We apologize for this mistake and corrected it.

Reviewer #4 (Remarks to the Author):

R4PA_B:

A. This work elegantly solves the current issues in quantifying protein expression levels by RNA-based approaches by incorporating a newly developed reporter system termed an exon-specific isoform expression reporter system (EXSISERS). The authors incorporated two EXSISERS into exons of interest (EOIs) by CRISPR/Cas9 and monitored the alternative splicing involved disease-associated exon inclusion of the patient-driven iPSC cells and screened RNA interference sequence for the isoform-specific expression to identify splice-regulators. Additionally, the authors similarly developed a survival reporter system for isoform-specific Blasticidin-S resistance marker. This article proposes the new exon-specific isoform expression reporter system would be a new tool for monitoring spatiotemporal exon-specific expression by imaging techniques.

B. This work is highly original and innovative with potential impacts in identifying splicing regulators and drug screening. Notably, this method could address the problems associated with protein expression level determined by RNA-based quantification methods. Thus, it is of significant importance and could be a game-changer for current RNA-based approaches if it is robust and reliable.

Response to R4PA_B:

We thank the Reviewer for acknowledging the advantages of EXSISERS' protein-level readout for drug screenings and basic research on identifying splicing regulators.

R4PC1:

C. In this system, there are several critical assumptions have not been controlled in this manuscript, which should be addressed in the manuscript before publications.

1. *The manuscript is described as if protein trans-splicing has 100% efficiency (like Fig 2a, 2b). The splicing efficiency by protein trans-splicing is strongly affected by the junction sequence and the foreign exteins used. A single mutation near the junctions could abolish or deceased the splicing activity significantly, missing the controls to check the protein splicing efficiency.*

Response to R4PC1:

We thank the Reviewer for this point regarding the efficiency of intein splicing.

In order to maximize efficiency, we chose fast-splicing inteins (doi:10.1038/s41467-020-15272-2, doi:10.1002/1873-3468.13909), which we further substantially enhanced with heterodimerization domains based on coiled-coils (CCs) (**Supplementary Fig. 1**). Recently, Bhagawati *et al.* (doi:10.1073/pnas.1909825116 showed in a similar approach, that intein splicing can be dramatically improved using a nanobody-antigen pair. By fusing an eGFP moiety to one half of a split-intein pair and an anti-GFP nanobody to the other split-intein counterpart, they could enable trans-splicing of a cysteine-free intein pair (important for extracellular protein splicing) that did not occur at all without the eGFP-nanobody interaction (please see their supplementary files Figure S10 vs. Figure S11).

These features enabled the very high splicing efficiency by immunoblot analysis of EXSISERS$_{MAPT\text{:}10NLuc\text{-}11FLuc}$ (**Supplementary Fig. 7**). Even when this construct was massively overexpressed via plasmid transfection, barely any unspliced proteins were detected (**Supplementary Fig. 2b**). Furthermore, the minigene version of this EXSISERS construct showed the same efficiency (**Supplementary Fig. 10c,d**).

As you requested in R4PF, the introduction of the terminal Asn→Ala mutation in the C-intein moiety completely disrupted protein splicing as expected (**Supplementary Fig. 2b**), thus indicating that the CCs-enhanced versions of the selected inteins are responsible for the exceptional high splicing efficiency.

With respect to considerations regarding the junction sequence, recent characterizations (doi:10.1002/1873-3468.13909) indicated that these 'ultrafast inteins' identified in metagenomic sources tolerate a broad spectrum of amino acids in heterologous settings very well (only proline is not tolerated heterologously and should be avoided). In conjunction with CCs, these efficiencies should increase even more.
In addition, we also now refer to the intein database in the method section under 'Application notes', which contains over 1000 inteins with known native extein sequences (maintained by the Iwaï lab, (InBase 2.0) https://inteins.biocenter.helsinki.fi/index.php), such that one can search for inteins with a desired native extein sequence to maximize the splicing efficiency.

R4PC2 and R4PC3:

2. Another assumption is similar to the previous one, FLuc and NLuc inserted in inteins fold into active equally with the same efficiency, yet having the same degradation rate in cells. The authors need to provide such experimental controls.
3. NLuc has 13-236 fold brighter than Fluc, according to the literature. All the data reported by normalized with the assumption, I believe.

Response to R4PC2 and R4PC3:

These assumptions do not have to be made. Instead, we measured the relative bioluminescence signal from *FLuc* and *NLuc* driven by a constitutive Pgk1 promoter at a 1:1 stoichiometry (**Supplementary Fig. 2a**). As seen in **Supplementary Fig. 2b**, the excision of NLuc/FLuc was very efficient. Moreover, we observed a linear relationship between the relative luminescence signals over 6 magnitudes and calculated NLuc yields 30 times more signal than FLuc (**Supplementary Fig. 2c**).

The Reviewer is also correct that for screening for modifiers of isoform expression, the bioluminescent signals were normalized to the control condition such that all relative differences between NLuc and FLuc are taken into account, and the effects of the perturbations can be directly seen. We have added additional notes in the figure legends and the manuscript to make the normalization procedure more explicit.

R4PC4:

4. The main caveat of this system easily overlooked by non-experts is the assumption that protein splicing by two split inteins has 100% or close to 100% efficiency. Particularly such high splicing activity for two orthogonal inteins has not been achieved in the past with an artificial system to my best knowledge. The reported efficiency of 95% in the cited ref.17 would result in the 90% efficiency for two orthogonal inteins. This assumption could determine the outcome of the analysis based on NLuc/FLuc quantification drastically.

Response to R4PC4:

As reported in subpoint R3PC1, we have used coiled-coil-enhanced fast-splicing inteins, and thus it is expected to have a greater efficiency than the reported value in the literature. We showed in **Supplementary Fig. 1** that CCs increased the protein splicing by nearly one

magnitude (8.6-fold), which is exceptional considering the folding speed of the challenging surrogate extein mNeonGreen with less than 10 minutes (doi:10.1038/nmeth.2413). We have also conducted detailed immunoblot analysis of the dual-luciferase EXSISERS$_{MAPT:10NLuc-11FLuc}$ upon plasmid-based overexpression (new **Supplementary Fig. 2b**), when genomically integrated (new **Supplementary Fig. 5b**, new **Supplementary Figure 6 and 7**), and as overexpressed minigene variant (new **Supplementary Fig. 10c,d and** new **Supplementary Fig. 12**), and detected no relevant levels of unspliced products even not upon heavy overexpression and overexposure.

R4PD:

D. NLuc usually has 13-236 >times brighter than FLuc according to the literature, which is consistent with the data presented with Figure 2e. The NLuc/Fluc error bars cannot be smaller than each of them. However, Figure 2j and all other data presented in Figure 3 do not make any sense, statistically.

The error estimation (P-value analysis) needs to be reconsidered. There are two types of errors mixed: (1) Errors from the detection (readout values) and (2) errors from individual samples or measurements. Even when the calculated error estimated from 3 samples is small, the accuracy of the measurement cannot be better than the precision of the detection errors.

Response to R4PD:

NLuc is indeed ~30-fold brighter compared to FLuc in the dual-luciferase EXSISERS. Please see **Supplementary Fig. 2c** for the calibration we performed to adjust for the relative differences in the bioluminescent signal obtained from the two luciferases when expressed at 1:1 stoichiometry. We adjusted for those relative differences in brightness in **Fig. 2 and 3** by normalizing the relative luminescence units (RLU) to the reference condition (with *MAPT* induction but without perturbation), such that the relevant effects of the perturbation of exon-specific isoform expression can be more readily read from the figures. This procedure is described in the Figure legends, the Material and Methods section, and the Statistics section.

Concerning the error calculation, the purpose of the dual-luciferase EXSISERS is to extract a robust, ratiometric measure of isoform-specific expression (NLuc) corrected for overall gene expression of tau (FLuc). The range of isoform-specific expression is thus naturally dependent on the overall expression. The FLuc and NLuc signals are also experimentally dependent on the cell lysis step in the Promega detection workflow that we employed (https://www.promega.de/-/media/files/resources/protocols/technical-manuals/101/nanoglo-dual-luciferase-reporter-assay-protocol.pdf): FLuc substrate is provided together with a lysis buffer onto the cells, followed by the first measurement (FLuc); in the 2nd step, NLuc substrate is provided together with a FLuc inhibitor, followed by the 2nd measurement (NLuc). Thus, for every FLuc RLU data point, there is a matching NLuc data point (paired measurement).

To reduce the biological variability from pan-tau expression and experimental variability stemming from the lysis and detection procedure, it thus makes sense to take the NLuc/FLuc ratio from each sample's cell population and calculate the average and errors over cell populations.

Calculating the errors of NLuc and FLuc separately over the biological triplicates would instead discard the information that the NLuc/FLuc pair was obtained from the same sample and thus defeat the purpose of absorbing the main source of variability.
Although the main conclusions are supported by statistical analyses directly on the NLuc/FLuc ratios, we still find it informative to also display the FLuc and NLuc signals separately, to, *e.g.*, show the effects of tau induction for reference or show the effects of an extended crRNA spacer on pan-tau expression.

We have explained this aspect of data processing in the figure legend and the Statistics section.
For completion, we also show all individual data point on top of the bar graph and provide a comprehensive table showing all raw data and detailed statistical results (**Supplementary Table 1**).

R4PE:

E.

As suggested in section C, D, and F, the validity of this system needs to be validated by additional controls. The authors should describe what would be potential pitfalls by the use of this reporter system. The current presentation does not provide sufficiently clear data to judge the validity and reliability of the system.

Response to R4PE:

We thank the Reviewer for the constructive suggestions of more data from control experiments to validate the experimental findings of the manuscript. We added RT-qPCR data (**Supplementary Fig. 14**) to confirm the key messages of **Fig. 3**. Furthermore, we added controls that the excision mechanism is indeed dependent on CCs-enhanced inteins by mutating the essential Asn of the C-inteins (C-gp41-1_{N37A} and C-NrdJ-1_{N40A}) (**Supplementary Fig. 2a,b**).
As requested, we have added paragraphs to the Materials and Methods section regarding the design criteria and potential pitfalls of EXSISERS constructs, the validation experiments to confirm efficient splicing of a given construct in analogy to our **Supplementary Figures 2,5,6,7, and 12**), a direct comparison to a minigene variant (**Supplementary Figure 10**), and detailed descriptions of how to generate clonal EXSISERS cell lines complementing **Supplementary Figures 3 and 4**.

R4PF:

F.

• *There is no estimation of protein splicing efficiency for none of their protein splicing constructs except for mNG shown in Supplemental Fig. 1 by immunoblot. This data also does not give any estimate of the fully spliced vs by-products (non-spliced, N- and C-cleaved products). The supplemental Fig. 1 should be supplemented by immunoblotting and/or CBB-stained SDS-gels using, for example, anti-Ollas and Flag antibodies. The quantification by Nluc/Fluc ration will be strongly affected by the ligation efficiency, which is strongly dependent on the foreign extein and the splicing junctions.*

Response to R4PF-part1

As requested, we updated **Supplementary Fig. 1.**, where we also now show an additional overexposed and contrast-enhanced image to detect all potential relevant side products. We

also added full immunoblots in the new **Supplementary Fig. 7, Supplementary Fig. 10c,d and Supplementary Fig. 12**.

Regarding **Supplemental Fig. 1**, we deliberately chose mNeonGreen as a model Extein with extremely fast folding rates (<<10 minutes, doi:10.1038/nmeth.2413) to define a maximally high benchmark for the intein-splicing speed. We have now added a densitometric quantification of the immunoblot in **Supplemental Fig. 1**, which shows that the addition of coiled-coils as heterodimerization domains improves the product/educt ratio by ~9 fold.
We have also added a deliberately overexposed immunoblot on which a small amount of side-products from C-cleavage can be detected that, however, amount to only ~3%.

In comparison to this test system, we have conducted detailed analyses of the protein splicing in the dual-luciferase reporter system for exon 10 inclusion of MAPT ($EXSISERS_{MAPT:10NLuc-11FLuc}$). Full immunoblots from multiple clones show essentially no unspliced products for tau **(Supplementary Fig. 7)**. Only under extreme overexposure, weak bands appear at densities of less than <1% of the spliced products, which probably correspond to the de novo translated proteins.
Even when the dual-luciferase reporter construct was heavily overexpressed at unphysiological levels from a plasmid (**Supplementary Figure 2b**) or as a minigene-version (**Supplementary Fig. 10d**), we could barely detect any unspliced educt.

• *What is the correlation between the quantification by immunoblotting (and/or mRNA quantification) vs NLuc/FLuc ratio for different constructs? Does it correlate well? if not, do they have a similar trend, which could be explained to some extent?*

Response to R4PF-part2

We performed additional experiments for the key messages of **Fig. 3** in HEK293T cells and quantified them via RT-qPCR. The observed effects and quantities were comparable between luciferase-based readout of $EXSISERS_{MAPT:10NLuc-11FLuc}$ cells and RT-qPCR of unmodified HEK293T cells (see **Supplementary 15** *vs.* **Fig. 3**).
Densitometric analysis of **Fig. 2d** also correlated well with the luciferase-based readouts (see new **Supplementary Fig. 5b** *vs.* **Fig. 2e**).

• *See also section D on the statistical data analysis.*

Response to R4PF-part3

Please see R4PD regarding the statistical analysis.

• *Fig.2d needs controls for protein-splicing deficient constructs by Ser-to-Ala and/or Asn-to-Ala.*

Response to R4PF-part4

We added **Supplementary Fig. 2**, where we expressed the cloned 0N4R cDNA of $EXSISERS_{MAPT:10NLuc-11FLuc}$ with intein-inactivating mutations in the C-intein moiety. The results show that active inteins are indispensable for the generation of the desired unmodified WT 0N4R tau band.

• *The authors claim "bio-orthogonal pair" of two inteins, but there is no such experimental evidence provided, including cited ref. 17. Trans-splicing is strongly dependent on the*

extein, the authors could provide such data as a control, as this will affect the interpretation of the ratiometric data significantly. The orthogonality of two split intein should be demonstrated by using their systems because protein splicing by inteins is strongly extein-dependent.

Response to R4PF-part5

The inteins gp41-1 and NrdJ-1 have already been shown to be orthogonal by Pinto *et al.* (doi:10.1038/s41467-020-15272-2), which we cite in the main text.

We have not seen any mis-spliced products from these inteins, such as N-NrdJ-1- or C-gp41-1, which would have appeared as additional bands of lower molecular weight on the immunoblots (**Supplementary Fig. 2b,7, 10c, and 12**).

Moreover, the orthogonal pairs of coiled-coils, which likely dimerize already at the secondary structure level before any intein or extein segments can fold, add a second level of orthogonality.

• The author provided only one experimental data in Supplemental Fig 1 of immunoblotting and did not disclose any further sequence in detail. At least Supplemental Fig. 1 could be supplemented by covering all possible products using anti-Olla and Flag antibodies and provide the protein splicing efficiency quantitated for each of the two splicing steps. In theory, cleaved products might not interfere with NLuc/Fluc ratio. Do the authors have any evidence to assume that is the case?

Response to R4PF-part6

We updated **Supplementary Fig. 1** with an overexposed and contrast-enhanced immunoblot. We see a weak band for C-cleavage (~3%) using the fast-folding mNeonGreen as a surrogate extein sequence. Via densitometry, we could quantify that the addition of Coiled-Coils could enhance the protein splicing efficiency by ~9 fold. Please also see the full immunoblots in **Supplementary Fig. 7, 10c, and 12**, which show that the splicing efficiency was even higher for both inteins together with >99%.

• The main claims generally focus on the Ratio-metric assay using NLuc/Fluc, the survival system using BSD could be more confusing for readers than making it clear to understand the reporter system as currently written.

Response to R4PF-part7

We appreciate the Reviewer's suggestion but still find it valuable to showcase the versatility of the EXSISERS technology that goes beyond reporter signals. The capability to non-invasively couple the in- or exclusion of an exon to cell survival enables unbiased screenings for new splicing regulators, such as genome-wide CRISPR/Cas9-mediated KO screens. This powerful methodology was not possible before.

As an extension, one could also imagine to use dCas9-activator screens or instead use a triggerable toxin such as HSV-Tk, to screen for exon exclusion instead of inclusion.

R4PH:

H.

• The abstract is concise and clear.

• There are several misleading statements in the introduction, the authors claim "fast" protein splicing but no speed or relevant time scale is given. Protein splicing is strongly context-dependent, has to be investigated for each extein. This claim is thus not validated in

the manuscript. Moreover, there is no information about "trace-less" because the authors do not disclose the protein sequence for junction regions. "Traceless" should mean the spliced sequence is identical to the original protein sequence without a single mutation. Is this the case?
• The current data is not sufficiently supporting the conclusion because of several assumptions and lacks critical controls to verify each of the critical assumptions.

Response to R4PH:
We have now added a series of additional control experiments to further support that the very efficient intein splicing does not alter the physiological isoform expression and are thus scarless.

To initially investigate and optimize the splicing efficiency of the inteins, we created a construct using mNeonGreen as an extein with folding rates of <10 minutes (please see Supplementary Fig. 1). Even under these extreme conditions, our final design, including coiled-coils (CCs) achieved a significantly greater extein to intein-extein ratio, indicating higher protein splicing efficiency (~9-fold increase in efficiency, **Supplementary Fig. 1**).

Nevertheless, the Reviewer is, of course, right that our measurements did not include precise timing and therefore we have changed the term from 'fast' to 'efficient' in the abstract and the introduction. Still, we used the term 'fast' in the beginning of the results section when we refer to gp41-1 and NrdJ-1 inteins since the literature described them as ultrafast splicing inteins (doi:10.1073/pnas.1701083114, doi:10.1021/jacs.7b02618).

Application of EXSISERS on *MAPT* showed a very high protein splicing efficiency (**Supplementary Fig. 7, Supplementary Fig. 10c, and Supplementary Fig. 12**). Please also refer to the detailed answer to R4PF. With the 'classic inteins', such as Ssp or Npu DnaE, intein splicing is highly dependent on the extein sequences, but with those 'ultrafast inteins' identified in metagenomic sources, the literature (doi:10.1002/1873-3468.13909) showed that they tolerate heterologous settings very well (only proline is not tolerated by all inteins in a heterologous context).

Besides the recently discovered classes of fast and efficient inteins, we like to refer to the nicely maintained database from the Iwaï lab (formerly maintained by New England Biolabs), where one can screen for inteins where the native extein sequences are identical or similar to the desired insertion site. As an example, we used this database, to search for inteins suitable to split Cas9 between position 573 and 574 (KIE|CFD), *Npu* intein with the native extein sequence (AEY|CFN) which critical +2 position fits to the intended Cas9 split-site (doi:10.1093/nar/gkv601). Notably, we did not see any difference in activity between WT Cas9 and Npu intein split-Cas9.

We neither introduced any extra Ser/Cys/Thr, nor did we change any amino acid to Ser/Cys/Thr, but merely used the natively occurring Ser/Cys/Thr of an exon, therefore we consider it justified to use the term 'traceless' or 'scarless'. Please also see the Materials and Methods section 'Generation of stable cell lines with tagged exons via CRISPR/Cas9', where we described how we inserted EXSISERS into the GOI.

We also added additional experimental controls, such as RT-qPCR on unmodified HEK293T cells (**Supplementary Fig. 14**) data to substantiate our data from **Fig. 3** in

EXSISERS$_{MAPT:10NLuc-11FLuc}$ cells. We also added additional dual-luciferase assays data from other clones to exclude clone-dependent artifacts (**Supplementary Fig. 17**). Moreover, we included additional full-range immunoblots to show the high protein splicing efficiency of the CCs-improved inteins (**Supplementary Fig. 7, Supplementary Fig. 10c,d,, and Supplementary Fig. 12**).

Decision Letter, fourth revision:

Date: 8th March 21 06:32:53
Last Sent: 8th March 21 06:32:53
Triggered By: Edmund Irwin
From: ncb@nature.com
To: gil.westmeyer@tum.de
CC: ncb@springernature.com, Jie.wang@nature.com
Subject: NCB: Your manuscript, NCB-W40046D
Message: Our ref: NCB-W40046D

8th March 2021

Dear Dr. Westmeyer,

Thank you for your patience as we've prepared the guidelines for final submission of your Nature Cell Biology manuscript, "Non-invasive and high-throughput interrogation of exon-specific isoform expression" (NCB-W40046D). Please carefully follow the step-by-step instructions provided in the personalised checklist attached, to ensure that your revised manuscript can be swiftly handed over to our production team.

We need to receive your revised paper, with all of the requested files and forms, within 5 business days, by 15th March 2021. Owing to strict production deadlines, failure to submit by this date will result in a delay in formal acceptance and publication. Please get in contact with us immediately if you anticipate delays, and provide us with an estimate regarding when you will submit these files.

When you upload your final materials, please include a point-by-point response to any remaining reviewer comments.

If you have not done so already, please alert us to any related manuscripts from your group that are under consideration or in press at other journals, or are being written up for submission to other journals (see: https://www.nature.com/nature-research/editorial-policies/plagiarism#policy-on-duplicate-publication for details).

In recognition of the time and expertise our reviewers provide to Nature Cell Biology's editorial process, we would like to formally acknowledge their contribution to the external peer review of your manuscript entitled "Non-invasive and high-throughput interrogation of exon-specific isoform expression". For those reviewers who give their assent, we will be publishing their names alongside the published article.

natureresearch

Nature Cell Biology offers a Transparent Peer Review option for new original research manuscripts submitted after December 1st, 2019. As part of this initiative, we encourage our authors to support increased transparency into the peer review process by agreeing to have the reviewer comments, author rebuttal letters, and editorial decision letters published as a Supplementary item. When you submit your final files please clearly state in your cover letter whether or not you would like to participate in this initiative. Please note that failure to state your preference will result in delays in accepting your manuscript for publication.

Cover suggestions

As you prepare your final files we encourage you to consider whether you have any images or illustrations that may be appropriate for use on the cover of Nature Cell Biology.

Covers should be both aesthetically appealing and scientifically relevant, and should be supplied at the best quality available. Due to the prominence of these images, we do not generally select images featuring faces, children, text, graphs, schematic drawings, or collages on our covers.

We accept TIFF, JPEG, PNG or PSD file formats (a layered PSD file would be ideal), and the image should be at least 300ppi resolution (preferably 600-1200 ppi), in CMYK colour mode.

If your image is selected, we may also use it on the journal website as a banner image, and may need to make artistic alterations to fit our journal style.

Please submit your suggestions, clearly labeled, along with your final files. We'll be in touch if more information is needed.

Nature Cell Biology has now transitioned to a unified Rights Collection system which will allow our Author Services team to quickly and easily collect the rights and permissions required to publish your work. Approximately 10 days after your paper is formally accepted, you will receive an email in providing you with a link to complete the grant of rights. If your paper is eligible for Open Access, our Author Services team will also be in touch regarding any additional information that may be required to arrange payment for your article.

Please note that you will not receive your proofs until the publishing agreement has been received through our system.

For information regarding our different publishing models please see our Transformative Journals page. If you have any questions about costs, Open Access requirements, or our legal forms, please contact ASJournals@springernature.com.

Please use the following link for uploading these materials:
[REDACTED]

If you have any further questions, please feel free to contact me.

Best regards,

Jie Wang, PhD
Senior Editor
Nature Cell Biology

Tel: +44 (0) 207 843 4924
email: jie.wang@nature.com

Author Rebuttal, fifth revision:

natureresearch

Responses to Referees

Non-invasive and high-throughput interrogation of exon-specific isoform expression

Dong-Jiunn Jeffery Truong[1,2], Teeradon Phlairaharn[1,2], Bianca Eßwein[3], Christoph Gruber[3], Deniz Tümen[1], Enikö Baligács[1,2], Niklas Armbrust[1,2], Francesco Leandro Vaccaro[1,2], Eva-Maria Lederer[1,2], Eva Magdalena Beck[1,2], Julian Geilenkeuser[1,2], Simone Göppert[1,2], Luisa Krumwiede[1,2], Christian Grätz[1,2], Gerald Raffl[1,2], Dominic Schwarz[1,2], Martin Zirngibl[1,2], Milica Živanić[1,2], Maren Beyer[1,2], Johann Dietmar Körner[1,2], Tobias Santl[1,2], Valentin Evsyukov[4,5], Tabea Strauß[4,5], Sigrid C. Schwarz[4,5], Günter U. Höglinger[4,5,6], Peter Heutink[7,8], Sebastian Doll[3], Marcus Conrad[3], Florian Giesert[3], Wolfgang Wurst[3,5,6,9], Gil Gregor Westmeyer[1,2†]

[1]Institute for Synthetic Biomedicine, Helmholtz Zentrum München, 85764 Oberschleißheim, Germany
[2]Department of Chemistry and TUM School of Medicine, Technical University of Munich, 81675 Munich, Germany
[3]Institute of Developmental Genetics, Helmholtz Zentrum München, 85764 Oberschleißheim, Germany
[4]Department of Neurology, Technical University of Munich, 81377 Munich, Germany
[5]German Center for Neurodegenerative Diseases (DZNE), 81377 Munich, Germany
[6]Munich Cluster for Systems Neurology (SyNergy), Adolf-Butenandt-Institut, Ludwig-Maximilians-Universität München, 81377 Munich, Germany
[7]Department for Neurodegenerative Diseases, Hertie Institute for Clinical Brain Research, University of Tübingen, 72076 Tübingen, Germany
[8]German Center for Neurodegenerative Diseases (DZNE), 72076 Tübingen, Germany
[9]Chair of Developmental Genetics, TUM School of Life Sciences, Technical University of Munich, 85354 Freising, Germany.
†corresponding author

Note: Since the figures were substantially restructured and reordered for the final submission as per editorial request, we here provide a lookup table specifying the mapping of previous to final figure indices.

Figure indices for Responses to Referees	Figure indices of Final Submission
Fig. 1	Fig. 1d,e partially moved to Fig. 2a
Fig. 2a,b	Fig. 1a,b
Fig. 2c	Supplementary Fig. 3a (left)
Fig. 2d	Fig. 1c
Fig. 2e	Fig. 1d
Fig. 2f	Fig. 2d
Fig. 2g BLI	Fig. 2b
Fig. 2g histogram	Fig. 2c
Fig. 2h	Fig. 2e
Fig. 2i	Fig. 3a
Fig. 2j	Fig. 3c
Fig. 2k	Fig. 3b
Fig. 3a	Fig. 4b
Fig. 3b	Fig. 4a
Fig. 3c	Fig. 4d
Fig. 3d	Fig. 4c, Fig. 5a
Fig. 3e	Fig. 5b
Fig. 3f	Fig. 5e
Fig. 3g	simplified to Fig. 5c,d
Fig. 4a	Fig. 6a
Fig. 4b	Fig. 6c
Fig. 4c	Fig. 6d
Fig. 4c	Fig. 6d
Fig. 4d	Fig. 6f
Fig. 4e	Fig. 6e
Fig. 4f	Extended Data Fig. 10
Supplementary Fig. 1	Extended Data Fig. 1
Supplementary Fig. 2a	Extended Data Fig. 2a
Supplementary Fig. 2b	Extended Data Fig. 2b
Supplementary Fig. 2c	Extended Data Fig. 2c
Supplementary Fig. 3	Supplementary Fig. 1
Supplementary Fig. 4	Supplementary Fig. 2
Supplementary Fig. 5a	Supplementary Fig. 3a (right)
Supplementary Fig. 5c	Supplementary Fig. 3c
Supplementary Fig. 6	Supplementary Fig. 4
Supplementary Fig. 7	Supplementary Fig. 5
Supplementary Fig. 8	Supplementary Fig. 6
Supplementary Fig. 9	Supplementary Fig. 7
Supplementary Fig. 9	Supplementary Fig. 7
Supplementary Fig. 10a	Extended Data Fig. 3a
Supplementary Fig. 10b	Extended Data Fig. 3b
Supplementary Fig. 10c	Extended Data Fig. 3c
Supplementary Fig. 10d	Extended Data Fig. 3d
Supplementary Fig. 10e	Extended Data Fig. 3e
Supplementary Fig. 11a	Supplementary Fig. 8a

Supplementary Fig. 11b	Supplementary Fig. 8b
Supplementary Fig. 12	Extended Data Fig. 4
Supplementary Fig. 13	Extended Data Fig. 5
Supplementary Fig. 14a	Extended Data Fig. 6a
Supplementary Fig. 14b	Extended Data Fig. 6b
Supplementary Fig. 14c	Extended Data Fig. 6c
Supplementary Fig. 15	Supplementary Fig. 9
Supplementary Fig. 16	Extended Data Fig. 7
Supplementary Fig. 17a	Supplementary Fig. 10a
Supplementary Fig. 17b	Supplementary Fig. 10b
Supplementary Fig. 17c	Supplementary Fig. 10c
Supplementary Fig. 18a	Extended Data Fig. 8a
Supplementary Fig. 18b	simplified to Extended Data Fig. 8b
Supplementary Fig. 18c	Extended Data Fig. 8d
Supplementary Fig. 18d	Extended Data Fig. 8e
Supplementary Fig. 18e	Extended Data Fig. 8c
Supplementary Fig. 18f	Extended Data Fig. 8f
Supplementary Fig. 18g	Extended Data Fig. 8g
Supplementary Fig. 18h (left)	Extended Data Fig. 8h
Supplementary Fig. 18h (right)	Extended Data Fig. 8i
Supplementary Fig. 18i	Extended Data Fig. 8j
Supplementary Fig. 18j	Extended Data Fig. 8k
Supplementary Fig. 18k	Extended Data Fig. 8l
Supplementary Fig. 18l	Extended Data Fig. 8n
Supplementary Fig. 18m	Extended Data Fig. 8m
Supplementary Fig. 19a	Supplementary Fig. 11a
Supplementary Fig. 19b	Supplementary Fig. 11b
Supplementary Fig. 20a	Extended Data Fig. 9a
Supplementary Fig. 20b	Extended Data Fig. 9b
Supplementary Fig. 20c	Extended Data Fig. 9c
Supplementary Fig. 20d	Extended Data Fig. 9d
Supplementary Fig. 21a	Fig. 7a
Supplementary Fig. 21b	Fig. 7b
Supplementary Fig. 21c	Fig. 7c
Supplementary Fig. 22	Supplementary Fig. 12
Supplementary Fig. 23a	Supplementary Fig. 13a
Supplementary Fig. 23b	Supplementary Fig. 13b
Supplementary Fig. 23c	Supplementary Fig. 13c
Supplementary Fig. 24	Supplementary Fig. 14
Supplementary Fig. 24	Supplementary Fig. 15

Table R0 I Lookup table to map previous to final figure indices.

Response to Reviewers' Comments on the revised manuscript NCB-W40046C, now NCB-W40046D.

Reviewer #1 (on revised manuscript):

Remarks to the Author:

The authors have addressed all my concerns. This is a tremendous amount of work with appropriate controls and methodological details. I have no more concerns for publication.

Reply to Reviewer #1

We thank the reviewer for the constructive criticism and the compliment on our revisions.

Reviewer #2 (on revised manuscript):

Remarks to the Author:

In the revised manuscript, the authors conducted a series of experiments to address most of my concerns, and also give a detailed explanation on some of the point that they did not addressed with additional experiments. I think they made serious efforts to improve the paper. While I still have concerns on lack of new findings when they applied EXSISERS in this work, I think the method itself is valuable for further application, especially in the potential application on genome-wide CRISPR/Cas9 screen. Therefore I am generally satisfied with their revision.

Reply to Reviewer #2

We thank the reviewer for the thoughtful comments that helped to improve our manuscript.
We have now also followed up on a secondary hit in our EXSISERS CRISPR screen for splice modulators of *FOXP1* and validated that *MOV10*, an RNA helicase, is a previously unrecognized factor that favors the exclusion of FOXP1 exon18b (**Supplementary Fig. 21a,b,c**).

Reviewer #3 (on revised manuscript):

Remarks to the Author:

The authors responded well to previous comments, but (as other reviewers have noted) nevertheless they have not demonstrated that EXSISERS technology is broad enough of a tool to generate novel biological findings unattainable by other methodologies.

Reply to Reviewer #3, point 1:

We thank the reviewer for the additional comments on our Technical Report.
As suggested by the Reviewer, we have now conducted another in-depth analysis of the unbiased EXSISERS CRISPR screen and have found that *MOV10*, an RNA helicase, is a previously unknown factor promoting *FOXP1 exon18b* exclusion.
Please see our detailed response to point 3 below.

We have now also expanded on the EXSISERS$_{MAPT}$ reporter in patient-derived iPSCs and monitored 4R isoform expression during the differentiation process into cortical neurons in wt and IVS10+16 c>t genotype, to find clearly aberrant isoform expression already in undifferentiated cell states at levels clearly below the detection limit of immunoblot.
Please see our detailed response to point 2 below.

Reviewer #3, point 2:
The current text and the author's response to the review present EXSISERS as a desirable, general replacement for minigenes etc., which is not fully supported. While they do not entirely recapitulate the nuances of splicing, mini-genes are much faster to generate than ~4-6 weeks. For most applications where nuance matters, established endogenous locus RNA and protein techniques will be far easier and more informative than a relatively complex new method like EXSISERS. The manuscript should state that this method is attractive when matching baseline molecular phenotypes to endogenous levels is important.

Reply to Reviewer #3, point 2:
Nowhere in the texts had we made the unnecessarily broad claim that EXSISERS is a "general replacement for minigenes."
To clarify this, we now emphasize - exactly along the line of the Reviewer- that minigenes are certainly powerful and valuable tools that have contributed substantial insights into alternative splicing but may not always reflect all nuances of splicing for obvious reasons.

We write in a modified paragraph of the introduction:

> Although this method can efficiently give valuable insights into alternative splicing, it may not always reflect the physiological processes, because partial intron/exon motifs may be overexpressed at unnatural levels, while essential regulatory sequences may be truncated.

Instead of a general claim, we focused on specific cases of strong biomedical interest in which "matching baseline molecular phenotypes to endogenous levels is important":
(1) monitoring of *MAPT* isoform expression in patient-derived cells and
(2) unbiased screening for *FOXP1* splice modulation (point 3 below).

For *MAPT*, there is converging evidence that transiently expressed minigenes do not reflect all aspects of the physiological splicing behavior, especially if they are just transiently overexpressed and not stably integrated, which we have streamlined for EXSISERS via a convenient double-selection process (**Supplementary Fig. 4**).
Upon the Reviewer's previous request (please see point R3P2: below), we confirmed this possible constraint directly by conducting a head-to-head comparison of EXSISERS$_{MAPT}$ to *MAPT* minigenes.
We found that the minigene-based reporter incorrectly reported the true tau isoform-ratio by a factor of ~4 (please compare **Supplementary Fig. 5c** vs. **Supplementary Fig. 10e**), while EXSISERS$_{MAPT}$ showed a truthful depiction of the endogenous splice-ratio.

To again demonstrate the advantages of EXSISERS, we have thus also worked further with the EXSISERS$_{MAPT}$ reporter in patient-derived IPSC and monitored tau isoform expression over the differentiation process from neural precursor cells into cortical neurons lasting 3 months.

This state-of-the-art cell culture model is valuable for studying Frontotemporal lobar degeneration (FTLD), which affects cortical structures and not subcortical structures as in Parkinson's disease.
Thanks to the very high sensitivity of the dual-luciferase system, we could detect the fractional 4R isoform expression already during the early phases of the differentiation far below the sensitivity of immunoblot. Usually, only 3R tau is detected during the differentiation process. (doi: 10.1093/hmg/ddv246).
Thus, we could find that the 4R isoform expression of the mutant is already strongly (4-fold) elevated in the undifferentiated state. The expression then proceeds along a non-monotonical trajectory in several "waves" as opposed to the gradual increase in the wildtype.
This is an interesting observation as it points to a more complex splicing behavior in different time windows during which one can now seek to intervene pharmacologically to approximate the wild-type condition.

The ratiometric, highly sensitive readout enables using a minuscule amount of cell material to report relative changes in exon inclusion level on the protein level, allowing affordable high-throughput screenings, already non-matured smNPCs before they are detectable by classical protein-level detection methods.
Thus, the EXSISERS reporter neurons, differentiated from patient-derived IPSC, will be a valuable resource to monitor *MAPT* regulation close to the physiological condition.

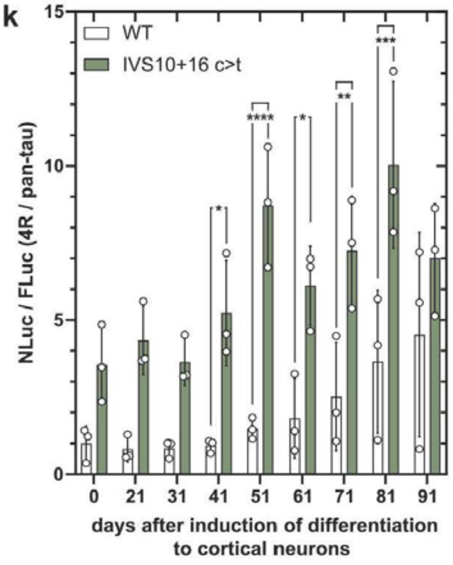

New Figure 2k | WT and IVS10+16 c>t iPSCs iPSCs were differentiated into cortical neurons over a time course of 3 months. Depicted are NLuc/FLuc ratios normalized to WT at day 0. Error bars and dotted lines represent standard deviation (n = 3). Only selected results of ANOVA post-hoc tests are shown with **, ***, and **** denoting p-values smaller than 0.01, 0.001, and 0.0001 respectively (full statistical results are available in **Supplementary Table 1**)

Reviewer #3, point 3:

Seemingly the real benefit of EXSISERS is in high-throughput applications, though this notion is also dubious—even in high-throughput screening applications it is likely not necessary to model the endogenous locus perfectly. If the authors believe that screening a recursively spliced locus is an exception, then they should demonstrate that application with EXSISERS. It is plausible in theory but doubtful that such a capability would generate much enthusiasm without such a clear demonstration.

If the authors were to make an unpublished high-throughout discovery with EXSISERS, that might be grounds for acceptance. As it stands, a natural conclusion for their story would be to follow up and validate top hits other than MBNL1/2 from their genome-wide CRISPR screen. But so far they have not demonstrated anything new, and therefore it is unclear that they would find anything new.

Reply to Reviewer #3, point 3:

Upon the suggestion of the reviewer, we have now followed-up on a secondary hit from the EXSISERS CRISPR screen that was enriched in both selection conditions of the survival screen: *MOV10* (**Supplementary Fig. 21a**.

We then confirmed *MOV10* via an independent sgRNA (#2) not previously used in the screen, which again lead to the survival of $\text{EXSISERS}_{\text{FOXP1:18bBSD}}$ colonies as compared to a control sgRNA (**Supplementary Fig. 21b**).

We then further confirmed via RT-PCR that *FOXP1* exon 18b was included in *MOV10*-KO cells in a blasticidin S concentration-dependent manner (**Supplementary Fig. 21c**). These data demonstrate that the RNA helicase MOV10 is a further factor for exon 18b suppression, albeit much weaker than MBNL1.

MOV10 is an interesting finding because it is not expressed in stem cells but upregulated upon differentiation (doi:10.1093/nar/gkaa1054). As an RNA Helicase, it may regulate access of splice regulators such as MBNL1 to the pre-mRNA, which may invite in-depth mechanistic studies, which are, however, clearly beyond our **Technical Report**.

These additional data show that, while *MBNL1* was found by an educated guess (doi:10.1038/nature12270), our comprehensive EXSISERS screen proved that MBNL1 is the main proteinogenic suppressors of FOXP exon 18b and identified an additional auxiliary factor that was previously not associated with splice regulation.

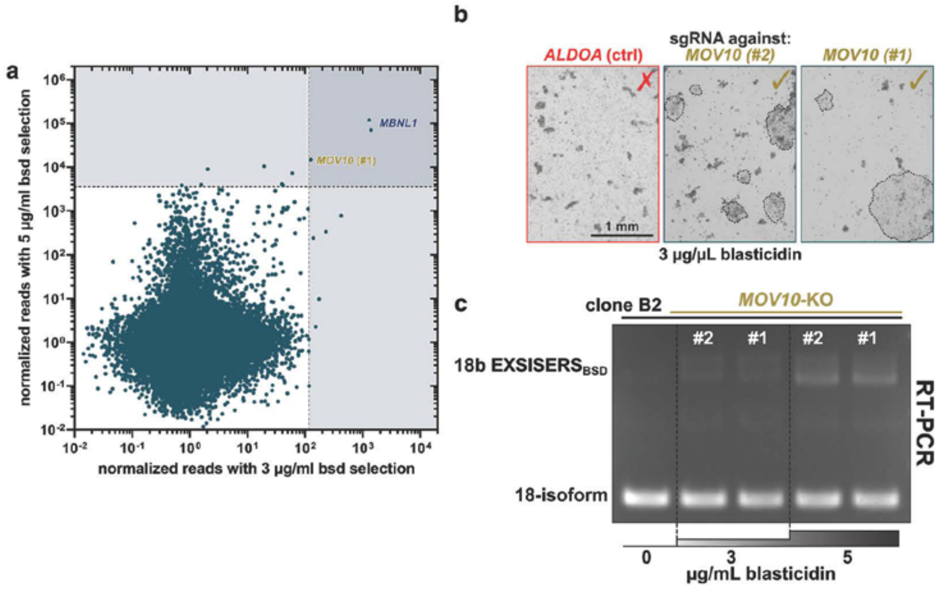

New Supplementary Fig. 21 | RNA helicase MOV10 is involved in *FOXP1* exon 18b suppression. a, Scatterplot of the reads (normalized to pre-selection reads) from both selection conditions (3 and 5 µg/ml blasticidin S). The areas highlighted in blue indicate the most strongly enriched sgRNAs for each selection condition. **b,** The enrichment of *MOV10* in the screen was confirmed by an independent *MOV10*-targeting sgRNA (#2) not used in the screen. Shown are colonies on a T75 flask 2 weeks after selection with 3 µg/ml blasticidin S and after transfection with CRISPR/Cas9 components against *ALDOA* (unrelated control gene, <10 colonies), the independent sgRNA #2 (>200 colonies in T75 flask)), and the sgRNAs against *MOV10* #1 used in the screen (>100 colonies). **c,** RT-PCR showing the blasticidin-concentration-dependent inclusion of *FOXP1* exon 18b from the colonies surviving blasticidin S selection shown in b, labeled with the respective sgRNAs.

We would like to emphasize again that we had, upon recommendation of the editor based on our pre-submission inquiry, submitted the current manuscript as a **Technical Report**, "which may involve a new biological discovery to prove the usefulness of the technique, but this is not a requirement."

We thus think that thanks to the Reviewers' constructive criticism, we have now characterized in great detail the technical advances of EXSISERS and have demonstrated the type of high-throughput reporter measurements and whole-genome survival screens, which EXSISERS enables to generate biomedical insights on isoform expression.

Reviewer #5:

Remarks to the Author:

Having looked in detail at the responses by the authors to the comments raised by the original referee 4, I think the authors have adequately addressed the concerns/points raised and the additioanl controls requested regarding validating the intein splicing efficiency and orthogonality.

However, I did not note the annotated sequences of the key genetic constructs were available. I suggest the authors to make them available either as in the supplementary or by uploading to a public database. In addition, it would be beneficial to the wide community to deposit the key constructs from the study in public repositories like Addgene.

Reply to Reviewer #5

We thank the additional reviewer for the positive feedback.
We have now combined all sequences of the EXSISERS components in the extensive **Supplementary Table 1** and will, of course, be happy to share them publicly.

Response to Reviewers' Comments on the initially submitted manuscript

Reviewer #1 (Remarks to the Author):

In this manuscript, Truong et al. describe a new tool, EXSISERS, to assess changes in splicing isoforms at the protein level and/or tag cells with specific protein splicing isoforms. The authors are taking advantage of the capacity of inteins to splice themselves out at the protein level, without affecting the RNA or coding sequence where there are integrated in. Using these inteins, they have shown with a wide set of examples, how they can insert at the endogenous level, in the alternatively spliced exon of choice, a reporter that can be spliced out at the protein level by specific inteins. With this system, by looking at expression of the protein reporter, which can be a luciferase protein, a blasticidine resistant gene, a fluorescent protein, an halo tag that goes to the membrane for cell sorting, one can identify, quantify, cell sort, live image cells expressing a specific splicing isoform of interest without the need of artificial reporters, splicing-specific antibodies, or the need to rely on RNA-based methodologies that most of the times are not impacting proteins at the same level. With this new system, one can assess the real splicing isoforms that exist at the protein level, follow them, manipulate them and even use them as a read out for CRISPR screening, imaging and sorting. It is extremely versatile and useful for studying many mechanisms and more importantly the biological relevance of a particular splicing variant at the protein level, and not the RNA level as we usually do (which underestimates all the post-transcriptional effects that could come from the new splice variant). Moreover, in the manuscript, the use of RfxCas13d and PspCas13b to specifically knock down one specific splicing isoform is also studied, bringing light to this also new and poorly understood tool. Key aspects of the crRNA design and if it is better to target the nascent pre-mRNA or the mature mRNA are shown.
Overall the manuscript is clear, robust and full of insightful new tools and recommendations to work with specific splicing isoforms at all possible levels. It is therefore of great interest for the scientific community and deserves publication if some concerns are addressed first.

Comments:

R1P1:

***1)** Since this is a manuscript selling a new tool, it would be nice if the authors comment whether it is difficult to endogenously tag at the homozygous level such reporter sequences. Have they tried many different type of cells? Which is the size of the biggest reporter they successfully inserted? I say this, because it is known that not all cells are easy to CRISPR tag and it is even more difficult to tag the two alleles, and even more two regions of the same gene at the two alleles. What happens in cells with more than two alleles? Is it really important to tag all alleles? All this could be commented to reinforce feasibility.*

Response to R1P1:

We thank the Reviewer for acknowledging the value of EXSISERS for studying isoform-specific expression.
With respect to cell types, we tested HEK293T, Neuro-2a, and several human induced pluripotent stem cell lines. Homozygous knock-ins were also achieved by a collaboration partner using an unrelated gene in HepG2 and HCT116 cells.
Although the efficiency of CRISPR/Cas9 type of gene editing tools will surely further improve and make systems such as EXSISERS even more convenient to use in the future, we have already achieved high single-copy knock-in efficiency and also high homozygous knock-in efficiency using the constructs we describe in detail in **Supplementary Fig. 3 and 4**. As an

example, out of randomly chosen 7 puromycin resistant clones, all were positive on at least one allele for $EXSISERS_{MAPT:10HaloTag}$ (new **Supplementary Fig. 22**).

$EXSISERS_{MAPT:10HaloTag}$ is our most complex construct (2.1 kbp without selection cassette and 4.4 kbp with selection cassette) containing two transmembrane segments and an extracellular HaloTag domain. Of the 7 positives, 3 were homozygous for $EXSISERS_{MAPT:10HaloTag}$.

With respect to ploidy, HEK293T cells, like many cell lines, are often triploid for most of the chromosomes, including chromosome 17, where *MAPT* is located. This property did not complicate CRISPR-Cas9-mediated genomic integration of the EXSISERS constructs.

Also, knock-in efficiency was good in human induced pluripotent stem cells (hiPSCs), which are known to be more difficult to modify by CRISPR, using the very same optimized components. When targeting exon 10, out of 21 picked clones, 14 (67%) were heterozygous, and 2 were homozygous (10%) for $EXSISERS_{MAPT:10NLuc}$, resulting in a total targeting efficiency of 76%. Similar targeting efficiency was achieved for exon 11 in hiPSCs for 15 picked clones with 11 clones being heterozygous (73%) and 3 clones (20%) being homozygous for $EXSISERS_{MAPT:11FLuc}$ (93% total efficiency).

As the Reviewer has already pointed out, homozygous targeting is indeed not necessary. We only used homozygous lines for subsequent analysis to show that EXSISERS is minimally invasive. Else, one could argue from the immunoblot analysis that the bands shown in, *e.g.*, **Fig. 2**, are from the untargeted WT allele. Thus, we can definitively conclude that the bands in our experiments are indeed the result of protein splicing. For standard experiments, heterozygous insertions can already be sufficient and can be obtained with high targeting efficiency.

R1P2:

2) *It is also important to prove that there is no effect on the endogenous transcript nor protein. That splicing occurs normally and the protein levels are not affected by insertion of these reporters and inteins. I don't think the authors have done this properly in the manuscript. Actually in Fig.2d, there is more 4R isoform in HEK than in the WT-EXISERS clone. Shouldn't these two cells be comparable? It is important to show that splicing patterns are not affected by insertion of these constructs, that protein levels are not affected, that function is not affected and that splicing could even change if necessary, such as in their iPS differentiation system. Also, can inteins have off target effects? This is not mentioned nor proved.*

Response to R1P2:

We have verified all EXSISERS lines carefully at the RNA and protein level and have added immunoblot (new **Supplementary Fig. 5,6,7,8,9 and 12**) and RT-qPCR (new **Supplementary Fig. 14**) data to show that there are no obvious detectable alterations in of the expressed isoforms and that all results from the EXSISERS reporters are in line with the data acquired on RNA level.

Concerning the variability of *MAPT* isoform patterns from different cells, it is important to mention that HEK293T is not a clonal cell line and showed some population variability. Analysis of HEK293T clones without *MAPT* modification showed only minor expression of 0N4R within a certain biological variation (**Supplementary Fig. 8**).

As per **Fig. 2d**, we have now performed densitometry on the 0N3R and 0N4R bands from an 16-bit uncompressed tiff file using the automated analysis from Image Lab (v6.1.0 build 7, Bio-Rad) and did not observe any obvious change in exon 10 inclusion between HEK293T WT cells and EXSISERS$_{MAPT:10NLuc-11FLuc}$ cells, which both showed ~3% inclusion of 4R-tau (new **Supplementary Fig. 5b)**.

In comparison, the pathologic mutation IVS10+16 c>t increased the fractional inclusion by ~3.7-fold), which is comparable to what we see from dual-luciferase EXSISERS (~4-fold), new **Supplementary Fig. 10e**) and also in accordance with the literature (2–6-fold, DOI:10.1074/jbc.274.21.15134 and DOI:10.1016/j.molbrainres.2005.02.014).

In addition, we also included a new immunoblot where we showed that EXSISERS$_{MAPT:10NLuc-11FLuc}$ cells are also comparable to the parental HEK293T cells in its response towards small molecule splicing modulators, such as 5'-iodotubercidin (ITU) (new **Supplementary Fig. 11 and 12**).

As further evidence for the reporter lines' physiological state, we had shown in main **Fig. 2c** that the tau filaments are formed in EXSISERS$_{MAPT:10NLuc-11FLuc}$ cells. Since we chose to use only homozygous EXSISERS cell lines for all experiments, those filaments must be formed from tau proteins that underwent protein splicing.
Also, the functional aspects on RNA-level, such as the regulatory hairpin of *MAPT*, were functional after EXSISERS insertion, as the well-characterized hairpin-destabilizing IVS10+16 c>t mutation led to a dramatic increase of exon 10 inclusion. As seen in main **Fig. 2d,e, and h**, and the new **Supplementary Fig. 5b,6,7, and 8** all other clones of EXSISERS$_{MAPT\text{-}IVS10+16:10NLuc-11FLuc}$ IVS10+16 c>t always showed a more prominent inclusion of exon 10 (4R isoform) compared to the WT counterpart, unmodified HEK293T cells and their clones.

The behavior of the EXSISERS construct used to screen for splicing modulators of FOXP1 (EXSISERS$_{FOXP1:18b\text{-}BSD}$) also indicates that splicing was not affected, as cells with homozygous insertion of EXSISERS$_{BSD}$ exon 18b did not show a changed blasticidin S sensitivity compared to HEK293T WT cells (data not shown). Since a minimal lethal blasticidin S concentration of 3 µg/mL was applied, even a minor increase in exon 18b inclusion would result in a surviving population of cells. As the exon 18b inclusion rate was already 0 % for cells lacking a MBNL1/2 KO in EXSISERS$_{FOXP1:18b\text{-}BSD}$ and HEK293T WT cells (**Main Fig. 4d, e**), a decrease of the inclusion rate would not have been possible.

Importantly, in the case of EXSISERS, we do not have to predict where a splice modulator, such as MBNL1 could bind, as the entire gene locus is present.
This stands in stark contrast to minigenes, where only those parts of a gene that are suspected to be involved in the splicing regulation are included in an artificial reporter system, resulting in a biased or knowledge-based screen. Please also see the comparison of EXSISERS with minigenes as part of our response to Reviewer 3 (R3P1 and R3P2).

Regarding off-targets of intein-splicing: Inteins originated from prokaryotes, archaea, algal cells, yeast, and other fungi. The protein splicing mechanism relies on autocatalysis and thus does not use up or interfere with any host proteins, nucleic acids, or any other host factors. Inteins are used in all kingdoms of life for biotechnological applications such as heterologous utilization in vertebrates, including mammals that do not have any inteins in the genome

natively. This heterologous usage in mammals, *e.g.*, to split Cas9 using protein trans-splicing in for rAAV delivery into pigs, did not show any side- or off-target effects on the organisms *in vivo* (doi:10.1038/s41551-019-0501-5 and doi:10.1038/s41591-019-0738-2). We have not made any observations in any of our EXSISERS implementations that would indicate such off-target effects.

R1P3:

3) A kind of related question: can you insert the NLuc/FLuc reporter anywhere in the exon regardless of the regulatory splicing sequences?
And how come increasing considerably the exon size has no effect on exon recognition and recruitment of the splicing machinery? As a splicing expert, it surprises me…

Response to R1P3:

In general, we carefully designed all EXSISERS constructs on the nucleotide level: we did use not only optimal mammalian codons but also avoided stable RNA secondary structures, and removed potential cryptic splice sites that may cause problems. We now included references to the software packages (Human Splice Finder v3.1 and NetGene2) in the Methods section under "Generation of stable EXSISERS cell lines with CRISPR/Cas9").

Regarding the insertion site, we emphasize the technical requirement for a Cys, Thr, or Ser in downstream of the insertion site (Ser and Thr are commonly found in regions containing loops and flexible linker amino acids). Furthermore, we paid attention to not modify any potential exonic splice enhancers and silencers/suppressors. For *MAPT* exon 10, there are 5 exonic splice modulators (doi: 10.1186/1750-1326-3-8), which were left intact upon insertion (see **Supplementary Fig. 9** for the insertion site of EXSISERS). We have also added a note to the method section that the insertion should be placed as distal as possible from exon-intron junctions to prevent undesired effects on RNA-splicing.

We also included data from an alternative insertion site (IS) of the alternatively spliced exon 10, which lies two amino acids (6 nt) downstream to the first IS. Again we took care not to disrupt known or potential splice enhancer/silencer motifs. The corresponding immunoblot did not reveal any obvious changes upon EXSISERS insertion at the 2nd site compared to unmodified HEK293T cells (**Supplementary Fig. 9**).

With respect to exon size, it has been suggested that large exon sizes are not a limiting factor in the identification of exons in alternative splicing (doi:10.1128/mcb.14.3.2140), which is in line with our experimental data. The prerequisite was that the inserted coding sequence did not contain any potential cryptic splice sites inducing aberrant splicing. In contrast, it has been suggested that the intron length has a major influence on alternative splicing, such as in the case of CD44 (doi:10.1128/mcb.18.10.5930).

We also designed our sgRNA in a way that the insertion of EXSISERS is sufficient to prevent Cas9 recutting, such that 'silent' synonymous codon substitutions are avoided, which can have unwanted side-effects as reported by Xiang *et al.* (10.1186/s13024-018-0280-6).

R1P4 and R1P5:

4) In Fig.2e, why there are equal levels of NLuc and FLuc in WT induced cells? If the exon is not included, NLuc should be lower than FLuc, right? Then with the use of 5-iodotubercidin,

which induces e10 inclusion, in suppl Fig.6 there is increase of both 4R (+ex10) and 3R(-ex10) isoforms. How come? 3R should not increase…
5) Are the two splicing intein proteins equally efficient splicing out the Luc proteins (Gp41-1 and NrdJ-1)? Maybe Suppl Fig 5 was intended to study this, but I don't understand the results. Looks like for each NLuc signal there are 30 of FLuc, which makes FLuc more efficiently spliced. Was this corrected in the main figures? It is kind of important since usually we look at the relative levels of the alternatively spliced isoform vs total protein. If one intein is more efficient than the other, it will affect interpretation of results. Also, can inteins splice out all the mRNAs translated? In a screening, can inteins be inhibited leading to indirect effects (no blasticidin not because there is no exon inclusion, but intein is inhibited or translation inhibited)?

Response to R1P4 and R1P5:
To adjust for the difference in the signal from FLuc and NLuc (due to differences in translation, half-life-time, enzyme activity, and brightness of the substrates), we expressed 0N4R-isoform from EXSISERS$_{MAPT:10NLuc-11FLuc}$ in which the two luciferases are driven at 1:1 stoichiometry by a Pgk1 promoter.
By transfecting increasing amounts of this plasmid, we established a linear relationship between the relative luminescence signals from FLuc and NLuc and determined that for our experimental settings, 30 RLUs of FLuc correspond to 1 RLU of NLuc, i.e., NLuc is 30-fold brighter than FLuc (original **Supplementary Figure 5b**, now **Supplementary Figure 2c**). As can be seen in the immunoblot (**Supplementary Fig. 2b**), this factor is not due to a difference in splice efficiency but rather a difference in substrate-dependent turnover rate and substrate/detection sensitivity.
In the new **Supplementary Fig. 5c**, we used this factor to adjust for the relative brightness and calculated the fraction of exon 10 inclusion to be ~5% in HEK293T-derived cells, in accordance with tau immunoblots (**Supplementary Fig. 5b**). The IVS10+16 c>t mutation led to a ~4-fold increase in exon 10 inclusion in the luciferase-based readout (**Supplemental Fig. 5c**), which matched the 3.7-fold increase, determined by immunoblot (**Supplemental Fig. 5b**).

Since the experiments of **Figure 3** are designed to show differential effects of pharmacological and genetic modulation of isoform expression, we have normalized all NLuc/FLuc data from EXSISERS$_{MAPT:10NLuc-11FLuc}$ to the control/baseline condition (induced *MAPT* but w/o perturbation), such that absolute differences in brightness are compensated, and differences due to the experimental perturbation can be directly read off the graphs.
We explained this normalization procedure in the figure legend, in the methods and statistics section.
Please also see our answers to R4PC2 and R4PC3.

With respect to your comment on the original Supplementary Fig. 6 (now embedded as **Supplementary Fig. 11a**), we thank the Reviewer for pointing out the inconsistency; indeed, the caption for this figure was mistakenly set. The caption was shifted by one position to the left; the legend has been corrected, and a new immunoblot has been inserted in the same as subfigure **b** with a finer titration of ITU. We are very sorry about this mistake and replaced the figure with a corrected version. Also, a similar immunoblot in direct comparison with EXSISERS$_{MAPT:10NLuc-11FLuc}$ has been inserted as new **Supplementary Fig. 12**.

R1P6:

6) Taking into consideration that the RNA is affected by using Cas13. It is important to show that the « protein » splicing effects observed with the inteins are also true at the RNA level by qRT-PCRs. e10 and total MAPT RNA levels should be affected accordingly in Fig.3. It is an important control.

Response to R1P6:

We have now performed RT-qPCRs experiments to validate all Cas13 key results of **Fig. 3** at the RNA level, *i.e.*,

a) Cas13d-NLS with an extended spacer is outperforming Cas13d-NLS with the originally published 22 nt spacer regarding general perturbation efficiency.

b) When Cas13d is applied in the nucleus using an isoform-specific spacer, it will still lead to a knock-down (KD) of all isoforms.

c) Cas13d applied on exon-junctions is more specific towards an isoform since it can only bind to the post-RNA-splicing mature mRNA.

d) shRNA is at least comparable if not superior to CRISPR/Cas13d or b, given that the latest miRNA scaffolds and the latest design rules are deployed. It also does not require the co-expression of two components (crRNA and Cas13).

R1P7:

7) In Fig.3c, why crRNA 10-11 is not affecting total MAPT levels but 9-10 is ? More puzzling, why the use of shRNAs to mimic miRNAs pathway has the opposite effect, it is the 9-10 that is more isoform specific than 10-11 ?

Response to R1P7:

We thank the Reviewer for this question regarding the details of **Fig. 3**.

In **Fig. 3c**, crRNA targeting exon 10-11 is clearly knocking-down 4R tau (NLuc) but seemingly not pan tau (FLuc). The reason is that the true fractional expression of 4R tau is very low (around 3-5%, please see R1P4 and R1P5 for details) compared to 3R tau (only very mature primary neurons in a complex 3D culture model are expressing a significant level of 4R tau (doi:10.1016/j.scr.2019.101541), thus even a 100% knock-down (KD) of 4R tau would just lead to an insignificant KD of pan tau.

The [2]9-10 crRNA is asymmetrically positioned on the 9-10 junction (=4R, **Fig. 3d**) and thus also matched almost perfectly on the 9-11 junction (3R, **Supplementary Fig. 16**) with only a single-nucleotide terminal mismatch (Cas13 systems tolerate single-nucleotide mismatches) resulting in the KD of all isoforms. For the 3rd generation shRNAs, the 9-10 microRNA (miR) was symmetrically positioned on the 9-10 junction (4R, **Fig. 3d**) and thus was specific for only 4R-tau since; an alignment of the 9-10 miR on the potential matching 9-11 junction (3R, **Supplementary Fig. 16**) showed 3 mismatches in the 5'-seed region (position 2–7) and thus was not activating the RNA-induced silencing complex (RISC) when accidentally bound to 3R.

In contrast, the 9-10 junction targeting miR was asymmetrically positioned onto the 9-10 junction (4R, **Fig. 3d**) due to design constraints of microRNAs and thus was also matching perfectly with its 5'-seed region (position 2–7) onto the 9-11 junction (3R, **Supplementary Fig. 16)** with only mismatches in its 3'-end that is tolerant towards mispairings.

Expectedly, the KD of 3R tau (crRNA targeting 9-11 junction) led to a clear decrease of pan tau signal (FLuc) without changing the 4R tau level (NLuc) in main **Fig. 3c**. This also has been confirmed in RT-qPCR in unmodified 293T cells in the new **Supplementary Fig. 14b**.
In summary, a strong depletion of pan tau (FLuc) in this cell line while trying knocking down 4R tau is clearly a side effect of lack of isoform specificity that can be observed for the exon 10 targeting crRNA and for the asymmetrical 9-10 junction targeting crRNA ([2]9-10), while the crRNA targeting the 9-10 junction symmetrically ([1]9-10) and the 10-11 junction are more specific.

R1P8:

8) Fig3f, dCasRX-SR effect is just 1,6x-fold. I don't think this is going to be biologically meaningful. The control in which there is dCasRx-SR or dCasRX-hnRNPA1 but not crRNA is missing (to make sure there are no indirect effects).

Response to R1P8:

The main objective of **Figure 3** is to show how EXSISERS technology can be used to optimize programmable effectors at the RNA level for modulating isoform-specific expression. We found a strong effect of the length of the guide RNA and the localization of the Cas13-effectors, while amiRNA was also very competitive.
To complete the picture, we also added data on the use of dead Cas13 systems for splicing modulation, because it is an application that is not possible with amiRNA.

We have now replicated the results on two independent clones, including the requested non-targeting controls (NTC) on another WT clone and also a clone carrying the IVS10+16 c>t mutation (**Supplementary Fig. 17**).
These results show that also small changes in isoform-specific expression can be quantified reliably with EXSISERS.
We did not express any opinion on whether the observed effects are biologically meaningful but simply suggest that EXSISERS can help to characterize and optimize systems that alter isoform-specific expression.

R1P9:

9) Again, the effect on Suppl Fig 12 seems very low too, 1,5x-fold. Is this sufficient to claim what the authors claim?

Response to R1P9:

We applied EXSISERS on a ribosomal-frameshifting-regulated gene to show EXSISERS' unique capability to monitor co-translational regulations, where RT-qPCR would fail. However, we did not claim a new finding. The observed effects are concentration-dependent and were independently confirmed with two complementary methods (fluorescence-activated cell scanning (FACS) and immunoblot analysis).

R1P10:

10) Why are the IFs in Fig2c and Supplementary Figure 11d,f so dotted at the nuclear level? Is this related to the reporter?

Response to R1P10:

Given that also unmodified HEK293T cells showed the 'nuclear dots' (new **Supplementary Fig. 5a**), they are likely a result of some unspecific binding of the pan-tau antibody (TAU-1 alias PC1C6) to nucleolar proteins in our immunofluorescence staining protocols.

R1P11:

11) For Fig.4, the CRSPR screening, it is important to know how many clones resisted to the blasticidin to know the false-positive rate of the system. The authors only show the positive MBNL1 clone, but this was already well known. Was the finding straightforward? It does not invalidate the proof-of-concept but it can give perspective on the feasibility of the system. It is known that some cells can escape the blasticidin selection. Were the authors using a higher amount of antibiotic than what is used for clone selection (1-10 ug/mL depending on the cell type)?

Response to R1P11:

We performed the experiment with a theoretical ~400-fold coverage of every sgRNA. The library contained ~80,000 sgRNAs against ~20,000 coding genes, including non-targeting control sgRNAs, resulting in 4 sgRNAs per gene. To achieve a ~400-fold coverage, we infected 100×10^6 cells with the lentiviral library with a multiplicity of infection (MOI) of ~0.3.

At least several hundred clones survived the most-stringent blasticidin selection condition (5 µg/ml). NGS analysis revealed that in this condition, 28.4% of the clones contained aN *MBNL1*-targeting lentiviral vector (composed of 18.8% and 9.6% of two different sgRNAs targeting *MBNL1*). Under low-pressure selection with the minimal inhibitory concentration of 3 µg/ml blasticidin-S, the flasks were confluent after the same timeframe. Still, based on the NGS analysis, 1.4% of the confluent population contained a lentivirus with a sgRNA targeting MBNL1. Also, based on NGS, only 0.0001% of the unselected control condition contained the same sgRNAs targeting MBNL1. This results in a 4 magnitudes of fractional enrichment in the 3 µg/ml blasticidin S condition and >5 magnitudes fractional enrichment for the more stringent 5 µg/ml blasticidin S condition. In other words, by simply subcloning the PCR product (instead of NGS) of the integrated lentiviral sgRNA expression cassette of the most stringent condition (5 µg/ml), followed by a standard Sanger sequencing of at least 20 clones, one would already expect 5-6 bacterial clones containing an *MBNL1*-targeting sgRNA. We emphasize that two independent sgRNAs targeting MBNL1 were independently

enriched by 3 magnitudes (3 µg/ml blasticidin-S) and 4 magnitudes (5 µg/mL blasticidin-S) over the median sgRNA population. Importantly, we validated the screen on a different EXSISERS$_{FOXP1:18b\text{-}BSD}$ clone using a 3rd independent sgRNA (different from the two enriched *MBNL1*-targeting sgRNAs of the library) targeting a constitutive *MBNL1* coding exon in parallel with a sgRNA targeting *MBNL2,* followed by blasticidin-S selection. Only the condition targeting *MBNL* genes led to blasticidin-S-resistant cells but targeting the control *AAVS1* locus did not. Moreover, when analyzing the surviving population via sequence decomposition of Sanger sequencing results, a dose-dependent accumulation of mutations in MBNL1 with increasing blasticidin-S concentration was indicative of functional coupling of the MBNL1-FOXP1-18b-Bsd-axis. With WT cells expectedly, we could not detect any resistant cells independently of any selection conditions and independently of the gene that was targeted. As described in **Fig. 4**, we used blasticidin-S in a concentration range the Reviewer indicated (3 µg/ml and 5 µg/ml are exactly in the range of 1-10 µg/ml).

Reviewer #2 (Remarks to the Author):

R2P0:

In this manuscript, the authors developed a new type of cell-based reporter system, exon-specific isoform expression reporter system (EXSISERS), which enables non-invasive detection of alternative splicing and exon-specific translation via intein-mediated protein splicing. They construct generated dual-luciferase (Nluc and Fluc) EXSISERS lines for ratiometric monitoring of different Tau protein isoforms, 3R-tau and 4R-tau. As designed, the system can recapitulate the expected change of different tau protein isoforms. The application of this reporter system was further demonstrated in several scenarios: 1. Screening of the effective guide RNAs in CRISPR/Cas-13 system that can achieve isoform-specific gene silencing; 2. Testing the activity of designer splicing enhancer or suppressor using the dCas-13 fusion protein containing SR domain or Gly-rich domain; 3. Measuring the co-translation ribosomal frameshift regulation. Finally, they generated an EXSISERS reporter for alternative splicing of exon 18b in FOXP1 and use the reporter to identify the regulators for isoform-specific expression of this exon via genome-wide CRISPR/Cas9 screen. Given their results the authors propose that it will be possible for an unbiased and non-invasive functional screening for splice modulators.

Overall I find the approaches employed in this study is valuable for characterizing and manipulating the intrinsic functionality of the exon-specific protein isoforms. However, the system is cumbersome to use and require a large amount of time for consecutive steps of CRISPR-cas insertion, which will limit its usefulness. In addition, some of the application did not perform as efficiently as previous system that was much simpler to generate. For example, the designer splicing enhancer and silencer using aCas-13 in EXSISERS reporter (Fig. 3f and 3g) was not as efficient as the engineered splicing factors using PUF fusion proteins (Wang Y et al, 2009 Nature Method, Wang Y et al 2013 NSMB), which is much simpler system to use. The authors should acknowledge such limitation and compare their system with previous system.

Response to R2P0:

We thank the Reviewer for acknowledging the value of EXSISERS to assess exon-specific protein isoform expression.

As we show in **Table R1**, EXSISERS has a unique set of advantages over other methods.

	EXSISERS	minigenes	immunoblot	immuno-cytochemistry	RT-qPCR	RNA-FISH
at endogenous site	☑		☑	☑	☑	☑
protein-level readout	☑		☑	☑		
cellular resolution	☑	☑		☑		☑
coupling of effectors to exon inclusion	☑	☑				
repeated measures	☑	☑				
no cell line needed			☑	☑	☑	☑

Table R1| Advantages of EXSISERS over alternative methods to detect isoform-specific expression

Although it is required to generate stable EXSISERS cell lines to ensure that isoform-specific expression is monitored at physiological levels, it is **not more cumbersome to generate those lines than it is to generate adequate minigenes**. Minigenes also have to be integrated into the genome to not unphysiologically overload the splicing/expression machinery, which will lead to aberrant alternative splicing behavior, as reported for, *e.g.*, MAPT.

Please see a comparative analysis of two minigene systems for *MAPT* in our response to R3P2.

To ensure maximal convenience in producing EXSISERS lines, we have streamlined the process such that only a **single cloning step** is necessary to generate the all-in-one CRISPR/Cas9 plasmid and the targeting plasmid, that can be inserted into the genome within **2-3 days** (please see **Supplementary Fig. 3**, previous Supplementary Fig. 2). The CRISPR/Cas9-mediated insertion is sufficiently efficient with the plasmids we provide, such that within just **2 months**, clonal EXSISERS cell lines can be generated (**Supplementary Fig. 4**).

With respect to efficiencies using CRISPR-Cas9-mediated insertions, please see the detailed response to R1P1 for targeting efficiencies of EXSISERS.

With respect to Pumilio/PUF-based splicing modulators, we agree that they are powerful and we, therefore, had already cited Wang, Y., Cheong, C., Tanaka Hall, T. *et al*. "Engineering splicing factors with designed specificities." Nat Methods 6, 825–830 (2009), doi:10.1038/nmeth.1379 in our original submission.

Since Cas13-based splice modulators are still currently of broad interest, chose this system to show that EXSISERS can be used to optimize it, but the same is, of course, goes for Pumilio/PUF-based splicing modulators.

R2P1:

Specific concerns:

1. The intein used in this study were shown to have high splicing efficiency (Supplementary Fig. 1) in their system, however I am curious about how efficiently the intein works in different cell lines. Additional quantification should be performed to measure the intein excision rather than assuming it is always 100% excised.

Response to R2P1:

Inteins have indeed been shown to be effective upon heterologous expression in several mammalian cell types *in vitro* and *in vivo*. Most importantly, applications in mammals, such as splitting Cas9 to circumvent the limited packaging capacity of recombinant adeno-associated viruses (rAAVs), a commonly used viral vehicle for gene therapy, by harnessing trans-splicing inteins ('protein ligation' of two co-expressed polypeptides), were effective *in vivo* in pig and mouse models (doi:10.1038/s41591-019-0738-2, doi:10.1038/s41551-019-0501-5).

We have further improved the high splicing efficiency of the fast-splicing inteins (doi:10.1074/jbc.M112.372680) by adding coiled-coils (CCs) to support cooperative folding of the cis-splicing intein halves and its excision. We updated **Supplementary Fig. 1** with data for which we used mNeonGreen as extein as it is known to fold extremely rapidly in much less than 10 minutes. Thus, we reasoned that this extreme case of a fast-folding extein should be maximally sensitive to detect any unproductive folding intermediates.

Under these circumstances, the CCs-enhanced intein resulted in a higher product/educt-ratio compared to the CCs-less counterpart. C-cleavage side products could only be detected upon overexposure and contrast enhancement. We did not detect any N-cleavage products.

Upon request of the Reviewer, we have now included full immunoblots from multiple clones showing essentially no unspliced products for tau. Only under extreme overexposure, weak bands appear at densities of less than <1% of the spliced products, which most likely correspond to the *de novo* translated proteins (**Supplementary Fig. 7**). Even for minigene-versions of EXSISERS$_{\textit{MAPT}:\text{10NLuc-11FLuc}}$, which are heavily overexpressed at unphysiological levels, we could barely detect any unspliced educt (**Supplementary Fig. 10c,d**).

In addition to our experiments with HEK293T cells, we have observed similar results from murine neuroblastoma cells (N2a) in which housekeeping gene (*Tubb3*) was intact (**Supplementary Fig. 18m**). Here, too, no unspliced educts could be detected.

R2P2:

2. In Fig.2, since the study is focusing on the exon-specific isoforms of tau protein, the authors should use an exon10 specific tau antibody (or pan antibody for tau) to calibrate the system. This is to make sure that the results obtained from luciferase measurement correlate well with direct measurement of tau isoforms.

Response to R2P2:

Reliable tau-specific antibodies are hard to get by. Still, we had screened several anti-tau antibodies and found that the best way to reliably identify 4R tau is by comparing a 3R-immunoblot to pan-tau immunoblots. We proved that this band is indeed the 4R band in **Supplemental Fig. 11a).** However, the S/N-ratio of this 4R-antibody (doi:10.1186/s13024-017-0229-1) is low, and we also needed to see the fractional inclusion of 4R from total tau. Thus, the anti-pan-tau antibody was the most informative tool for our requirements.

When WT HEK293T cells were treated with ITU known to increase 4R tau (doi:10.1111/febs.12411), the 0N4R band (2nd band from below in anti-pan-tau immunoblot, **Supplementary Fig. 11**) was clearly increasing while 0N3R was decreasing (1st band from below in the anti-pan-tau immunoblot, **Supplementary Fig. 11**). Similarly, the bioluminescent signal from EXSISERS$_{\textit{MAPT}:\text{10NLuc-11FLuc}}$ increased by ~4-fold (**Fig. 2f, j)** and longitudinally over a period of 60 hours in **Fig. 2h**.

In a direct comparison from unmodified HEK293T cells and EXSISERS$_{\textit{MAPT}:\text{10NLuc-11FLuc}}$ in the same immunoblot, increasing ITU concentration resulted in a fractional increase of 4R tau. In contrast, the total tau level decreased slightly (new **Supplementary Fig. 12**). As expected for EXSISERS$_{\textit{MAPT}:\text{10NLuc-11FLuc}}$, the OLLAS-positive band for excised NLuc (=4R) was getting more prominent with increasing ITU concentrations (**Supplementary Fig. 12**).

Furthermore, **Fig. 2d** showed that the IVS10+16 c>t mutation caused an ~3–4-fold increase of 4R-tau in both, immunoblot and in luciferase signal (**Fig. 2d,e and h, and Supplementary Fig. 5,6, and 7**). Please note that although the size separation and spatial resolution of the tau bands is high compared to typical anti-tau immunoblots in the literature (doi:10.1186/s13024-017-0229-1, doi:10.3892/ijmm.2012.1025), precise quantification of tau isoforms by densitometry is extremely challenging.

R2P3:

3. In Fig.4, I feel that this part lacks an important analysis on transcriptome level for the MBNL1/2-KO cells and the exon 18b inclusion cells after blasticidin selection. MBNL1/2 are key regulator in RNA splicing, and knock-out of these two genes should cause significant change of splicing in the level of entire transcriptome. I am wondering whether knock-out of these two genes could cause more exon-specific protein changes besides FOXP1.

Response to R2P3:

We agree with the Reviewer that it is interesting to ask which impact perturbations of MBNL proteins have on the transcriptome.

In our manuscript, however, it was the goal to present EXSISERS as a screening tool for unbiased identification of splicing modulators. Indeed, without any prior knowledge, we re-identified MBNL1 as the main regulator of *FOXP1* exon 18b inclusion using an unbiased lentiviral CRISPR/Cas9 screen, which was impossible before. We then followed up with a knockout of independent sgRNA targeting *MBNL1* to validate the results in our system.

With respect to the effects of MBNL on the transcriptome, we would like to refer to the thorough work of Han *et al.*, 2013 (doi:10.1038/nature12270), where they use RNA-seq profiling to analyze the impact of MBNL perturbations mediated by siRNAs. They showed that MBNL proteins negatively influence the global AS network important for pluripotency maintenance, partially by repressing the ES-cell-specific FOXP1 isoform, a stimulator of a core pluripotency circuit, thus promoting transcriptome-wide switch towards differentiation.

R2P4:

4. I think this paper may present a powerful tool to track and study exon-specific protein isoform. However, the authors should use it to investigate on new biological questions rather than only to confirm the conclusion people have already made.

Response to R2P4:

We thank the Reviewer for sharing enthusiasm towards EXSISERS as a 'powerful tool' to investigate alternatively spliced protein isoforms. While the main weight of such a methodological paper must clearly lie on the careful validation of the new instrument on the various technical levels against well-established results, we have made a few interesting observations showing the robustness and convenience of EXSISERS technology:

We showed for the first time,

a) the longitudinal readout of isoform-specific expression with cellular resolution of an alternatively spliced exon from the original genomic site in living cells,
b) an improved targeting efficiency of Cas13d significantly by the extension of the spacer length from 22 nt to 30 nt,
c) the importance to optimize the precise site of action for each programmable intervention tool (Cas13d or b, or shRNA in the cytosol) since it has a massive impact on the isoform specificity, even if the same position is targeted,
d) that shRNA - if carefully designed using the latest design rules and using up-to-date pri-microRNA biogenesis-mimicking scaffolds - can compete with Cas13-based systems regarding potency and isoform-specificity,
e) an independent confirmation of a serendipitous scientific finding of *FOXP1* exon 18b regulation via MBNL1 using a novel unbiased approach.

These examples lay out precise recipes for biological discoveries and there are already several laboratories in our network that are actively using EXSISERS technology to test their preferred biological hypothesis.

R2P5 (Minor P1):

Minor concern:

Overall the figures are poorly prepared with low resolution and confusing color scheme, more specifically:

1. The picture quality of Fig.2c and Fig.2g should be improved. The color and style of this figure should be modified to make it more reader friendly. In addition, Fig.2c and 2g should be showed in color to help understand.

Response to R2P5 (Minor P1):

We apologize that the quality of our figures was apparently compromised during compression. We are sorry for the compression artifacts of **Fig. 2** that occurred in the last submission. All our original figures are high quality.

R2P6 (Minor P2):

2. The picture quality of Fig.4c and Fig.4d should be improved. And the part (Identification of regulators for isoform-specific expression) and Fig.4 need be carefully reviewed, because the figure and the main text are not consistent.

Response to R2P6 (Minor P2):

We are sorry for the compression artifacts of **Fig. 4** that we improved. Furthermore, we thank the Reviewer for pointing out the disparity between main text and **Fig. 4**, we carefully re-read the main text and corrected inconsistencies with the figure.

R2P7 (Minor P3):

3. Supplementary Fig.8b need to be updated, as the resolution is very low.

Response to R2P7 (Minor P3):

We are sorry for the low quality of the original **Supplementary Fig. 8** (now improved in **Supplementary Fig. 19**). Regarding subfigure **b**, the GFP channel **did not show any signal** since in contrast to luciferases, endogenous expression of 4R tau did not yield enough protein to be readily detected in a common epi-fluorescence microscope.

R2P8 (Minor P4):

4. Similar to Fig. 2c, the supplementary Fig.11 and Fig.13 should be improved.

Response to R2P8 (Minor P4):

We improved the quality of the respective figures.

Reviewer #3 (Remarks to the Author):

R3P1:

Truong et al. develop a minimally invasive isoform-specific expression reporter system (EXSISERS) that incorporates translated and subsequently excised fast-splicing inteins with CC-domains into genes of interest. The authors demonstrate the utility of EXSISERS in a number of applications, ranging from the optimization of RNA-targeting strategies for exon-specific RNA degradation of MAPT mRNA, to the quantification of ribosomal frameshift-mediated regulations unmeasurable by RT-qPCR, to a phenotypic readout for a high-throughput screen of FOXP1 exon 18b inclusion that validates existing literature. Altogether, the presented work is a valuable addition to the isoform-specific RNA monitoring toolkit. While the generation of EXSISERS may be an involved process, nevertheless for some applications it might prove more useful than alternative methodologies, such as minigenes. I have a few major criticisms.

Response to R3P1

We thank the Reviewer for acknowledging the value of EXSISERS for monitoring isoform-specific expression. We have compiled **Table R1**, to compare the features of EXSISERS as compared with other relevant methods for detecting isoform-specific expression.

	EXSISERS	minigenes	immunoblot	immuno-cytochemistry	RT-qPCR	RNA-FISH
at endogenous site	☑		☑	☑	☑	☑
protein-level readout	☑		☑	☑		
cellular resolution	☑	☑		☑		☑
coupling of effectors to exon inclusion	☑	☑				
repeated measures	☑	☑				
no cell line needed			☑	☑	☑	☑

Table R1 | Advantages of EXSISERS over other methods to detect isoform-specific expression

Although many important findings were made possible by minigenes, they may (**1**) suffer from untruthful readout, (**2**) cause alterations of endogenous splicing, while (**3**) still requiring the same effort on cloning and generation of stable cell lines.

(1) Minigenes may lead to <u>untruthful readout of endogenous splice-regulation of a gene of interest</u> because they - with a high probability - do not contain all relevant regulatory elements. This is especially true for tau, where it has been shown that basically the whole intronic region is required to reflect the true splicing behavior for exon 10 (doi:10.1111/j.1471-4159.2004.02477.x). Most importantly, it has been shown recently that many identified SNPs have their origin deeply embedded within introns, such as the rs242561 polymorphism, that is protective against Parkinsonian disorders (doi:10.1016/j.celrep.2016.03.068). This single nucleotide polymorphism is located within the first intron 13.2 kbp upstream of the 2nd coding exon and 55 kbp downstream of the first coding exon; the same is true for rs242557 which is also associated Parkinson's disease,

which is located 48 and 20 kbp down- and upstream from the flanking exons (doi:10.1016/j.neulet.2010.10.015, doi:10.18632/oncotarget.16490) or rs2471738 that lies 11.6 kbp upstream of the alternatively spliced exon 10 and 2 kbp downstream of exon 9 (doi:10.18632/oncotarget.16490). Moreover, many vertebrate genes are recursively spliced which will not be recapitulated by minigenes (doi:10.1038/nature14466). Also, for other alternatively spliced genes such as CD44, the intron's length determines the inclusion efficiency of the alternatively spliced exon (doi:10.1128/mcb.18.10.5930). A mini-gene version that contains truncated introns would therefore inevitably lead to unphysiological splicing. Thus, it is essentially impossible to faithfully recapitulate the complex regulatory machinery outside the precise three-dimensional context of the endogenous sites.

(2) Minigenes are not applicable to unbiased screens for splice regulators (such as genome-wide CRISPR/Cas9 KO-screens) to enrich a certain population of cells with a defined genetic perturbation. Minigenes are normally used in a transient transfection assay and even if integrated into the genome, they lay outside of the endogenous site and are driven by constitutive promoters. They are, therefore, hiding effects of (co)-transcriptional regulations. Also, the truncated introns cannot reflect the physiological genomic context such that whole-genome screens would probably yield questionable results.

(3) Minigenes can **cause alterations of endogenous splicing of other collateral genes** by competitive binding of splicing factors to the constitutively overexpressed minigene. This results in depletion from endogenous sites. In the case of *MAPT*, the altered isoform ratios can even feed-back on the splicing process since the formation of aggregated neurofibrillary tangles leads to the co-depletion of the otherwise soluble spliceosomal components further increasing the aberrant change of the global cellular splicing pattern (doi:10.1016/j.celrep.2019.08.104).

(4) Minigenes require the same effort to establish as EXSISERS
We made sure that the production of the EXSISERS lines is as convenient as possible: we provide all EXSISERS reporters in a respective cloning vector, such that only a single cloning step is required to obtain a customized exon-specific EXSISERS vector (please see **Supplementary Fig. 3**). The CRISPR/Cas9 vector, improved with enhanced gene targeting efficiency, can also be cloned in a **single** step (please see **Supplementary Fig. 3**). Please also see our graphical abstract of the process (**Supplementary Fig. 4**), which shows hot an EXSISERS clonal cell line can be established in just ~4–6 weeks. Please also see our response R1P1.

With respect to the effort for making the respective cell lines, minigenes also require the assembly of different fragments of truncated exon-intron fragments and subsequent cloning into a mammalian expression vector. Usually, several minigene versions with different truncations need to be tested, since truncations can lead to the removal of essential regulatory sequences, which are important for the regulation of alternative splicing.

Furthermore, minigene systems that are not read out via RT-qPCR but via a reporter system - which is essential for high-throughput detection - require additional modifications in the alternatively spliced exons to include stop or start codons for fluorescent proteins or luciferases. Alternatively, a frameshift-based reporter to distinguish the ab- or presence of an exon can be used. This, however, requires also a deletion/insertion of 1 or 2 nucleotides,

since normally an alternatively spliced exon contains a number of nucleotides divisible by 3 (Stoilov *et al.* (doi:10.1073/pnas.0801661105), Luo *et al.* (doi:10.1002/cbic.201402069)).

Also, random integration of the minigene into the genome introduces an unnecessary variability due to copy number variation, impact on neighboring genes, expression strength, and splicing behavior (doi:10.1016/j.cell.2010.11.056). Additionally, screening compound libraries to alternate AS, library-scale minigene transfection for every condition would not be economically feasible.
In summary, also for minigenes it is recommended to knock-in into a well-defined safe-harbor locus (such as *AAVS1/PPP1R12C* in human and *Rosa26* locus in murine systems) using CRISPR/Cas9 (or TALENs, ZFNs) to minimize variability.

Please also see our detailed response to your request in R3P2 where we also carefully compared minigenes with EXSISERS.

R3P2:
Major points:
1. The authors do not perform any head-to-head comparisons of EXSISERS to minigenes, which are comparatively much simpler and faster to generate. This should be done. If there is no clear advantage of EXSISERS, then it is worth wondering whether other researchers will adopt the new methodology.

Response to R3P2
Thank you also for the constructive suggestion to perform a head-to-head comparison with minigenes.
To this end, we have carefully studied the elaborate minigene systems for *MAPT* by Yu *et al.*, (doi:10.1111/j.1471-4159.2004.02477.x) and Jiang *et al.* (10.1128/mcb.20.11.4036-4048.2000 to construct corresponding minigene systems.

Before we compare our results shown in **Supplementary Fig. 10**, we need to quickly review the pertinent findings from Yu *et al.*, which is a very careful study that, however, also demonstrates the complexity and potential pitfalls for obtaining truthful results with minigenes.
It can be seen from **Figures 1 and 2 in Yu *et al.*** (attached below with figure legend) that the authors laboriously tried out 10 different tau-4R minigenes with different intronic truncations but found that none of them showed physiological splicing behavior. Only a plasmid made from a construct with **full-length** introns of 17,485 bp (LI9/LI10) recapitulates the endogenous physiological ratio. Similar behavior for minigenes also could be observed by Jiang *et al.* (Fig. 2B vs. Fig. 2A, doi:10.1128/mcb.20.11.4036-4048.2000). Besides, using full-length introns in minigenes is technically very difficult, since those introns can easily reach 5-digit bp in length and thus require specialized PCR-protocols to be amplified. Equipped with a plasmid backbone of ~3 kbp, promoter elements, and the rest of the tau coding sequence, this plasmid would also easily exceed the 20 kbp limit for classic plasmid transfection (doi:10.1093/nar/27.19.3792, doi:10.1016/j.ab.2005.08.029). Also, for plasmids greater than 20 kbp, the increased risks of plasmid instabilities enforce the usage of bacterial artificial chromosomes (BAC) instead.
Aberrant splice behavior of minigene systems has also been reported for other genes than *MAPT*. For the *ABCA4* gene (128 kbp, 50 exons), which plays a role in the Stargardt disease, Sangermano *et al.* (doi:10.1101/gr.226621.117) *'[...] discovered that when using*

small minigenes lacking the proper genomic context, in vitro results do not correlate with splice defects observed in patient cells.' They *'[...] therefore devised a novel strategy in which a bacterial artificial chromosome was employed to generate midigenes, splice vectors of varying lengths (up to 11.7 kb)* ***covering almost the entire ABCA4 gene.'*** Only under these circumstances, a similar splicing behavior as observed in patients could be recapitulated.

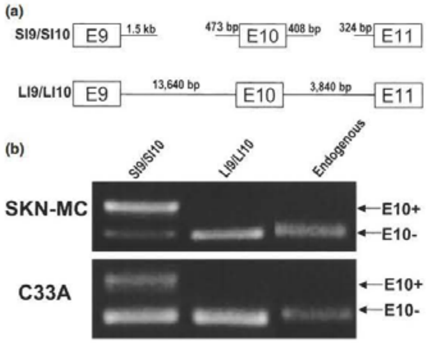

Fig. 1 Introns 9 and 10 affect splicing patterns of exon 10 in the *tau* gene. (a) Mini-gene constructs for splicing of exon 10 in the *tau* gene were generated in PCI-neo vector. The short previously published mini-gene SI9/SI10 includes exon 9, the first 1.5 kb and the last 473 bp of intron 9, exon 10, the first 408 bp and the last 324 bp of intron 10, and exon 11. The long mini-gene construct LI9/LI10 contains full length of both intron 9 and intron 10. (b) Mini-gene constructs were transfected into C33a or SKN-MC cells. Splicing patterns of exon 10 in mini-genes were examined by using RT–PCR. Splicing of exon 10 from the endogenous *tau* gene was detected in C33a cells or SKN-MC cells induced by 10 μM of sodium butyrate for 24 h.

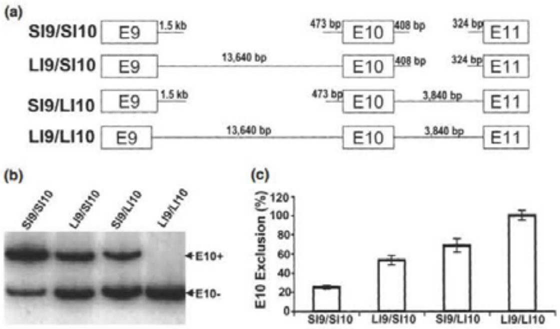

Fig. 2 Intron 9 and intron 10 additively contribute to correct splicing of exon 10 in the *tau* gene. (a) Constructs with a full-length intron 9 and a short intron 10 (LI9/SI10) or with a full-length intron 10 and a short intron 9 (SI9/LI10) were generated. The short intron 9 or short intron 10 was identical to that in SI9/SI10. (b) The constructs were transfected into SKN-MC cells. RT–PCR was used to determine splicing patterns of exon 10. (c) RT–PCR bands were quantitated using a phosphorImager. Bar represents the mean percentage of mRNA with exon 10 exclusion (E10 –) out of total mRNAs (E10 + and E10 –) from three separate transfection experiments. Error bars represent standard deviations of the means.

These results suggest that intronic truncations, an essential characteristic of minigenes, can be misleading, even if the minigene contained several hundred nucleotides of sequences down- and upstream of an exon of interest. Zheng *et al.* (doi:10.1101/gr.147546.112) also warned that *'[...] minigene reporters do not always recapitulate the regulation of endogenous*

exons. The minigene may not contain all of the relevant cis-regulatory elements for the test exon.'

Recent reports (doi:10.1038/nature14466) also suggested that vertebrate introns, especially long ones, are often removed stepwise in a process called 'recursive splicing'. Thus, a minigene with truncated introns would inevitably lead to an altered RNA splicing behavior. Especially vertebrate introns can be larger than 100 kbp and can hardly be cloned fully in a minigene. Most importantly, those long introns are not just 'junk', which can be replaced by random nucleotide sequences.

For example, Wang *et al*. showed recently that the rs242561 polymorphism is protective against Parkinsonian disorders (doi:10.1016/j.celrep.2016.03.068). This single nucleotide polymorphism is located within the first intron 13.2 kbp upstream of the 2nd coding exon and 55 kbp downstream of the first coding exon; the same is valid for rs242557, which is also associated Parkinson's disease, which is located 48 and 20 kbp down- and upstream from the flanking exons (doi:10.1016/j.neulet.2010.10.015).

A stably integrated minigene is also preferred over transiently transfected plasmids, as Jiang *et al*. (doi:10.1128/mcb.20.11.4036-4048.2000) noted regarding the tau minigenes. They note '*[...] that transfected tau minigenes in these cells produced a slightly higher level of Tau4R compared to the endogenous tau expression pattern (Fig. 2), suggesting that overexpression of the tau minigene may titrate certain limiting factors controlling the ratio of Tau3R to Tau4R*'. Stoilov *et al*. (doi:10.1073/pnas.0801661105) also suggested that minigenes should be stably integrated: '*Note that transient expression of the reporters can lead to significant cell-to-cell variation in the protein signals, which we attribute to differences in the stability of the two proteins and in the amount of DNA taken up by each cell. This* ***variability is reduced in stable cell lines expressing the reporter*** *and with reporters where the stability of the two proteins is equalized*'.

Thus, the minigene systems are not easier to create, especially not as a version compatible with high-throughput screenings (*e.g.*, using terminally fused luciferases), which necessitates additional mutations have to be introduced into the coding sequence of the exon of interest.

Based on the luciferase minigene system described by Yu *et al*. (doi:10.1111/j.1471-4159.2004.02477.x), we build a minigene by amplifying the corresponding intronic regions with truncation that are of similar length as in Yu *et al.*, and Jiang *et. al.* (doi:10.1128/mcb.20.11.4036-4048.2000), to create EXSISERS-based 4R-minigenes (**Supplementary Fig. 10a**).

In accordance with Yu *et al.* and Jiang *et al.*, we noted an increased exon 10 inclusion level (~12%, **Supplemental Fig. 10c**) originating from minigenes as compared to the endogenous locus (~3–5%, **Supplemental Fig. 5b**).

For the mutation IVS10+16 c>t, 4R/pan-tau ratio further increased by roughly 2-fold to over 50%. In contrast, with integrated EXSISERS, we did not detect any significant difference between unmodified HEK293T cells, its clones, and EXSISERS$_{MAPT:10NLuc\text{-}11FLuc}$ (**Fig. 2d, Supplementary 5, 6, 7, 8, 9, and 12**).

The reaction of EXSISERS$_{MAPT:10NLuc\text{-}11FLuc}$ in response to small molecule perturbation (**Fig. 2f, h, j, and Supplementary 11 and 12**), and Cas13/microRNA-based modulation (**Fig. 3** *vs.* **Supplementary 14**) was similar to the reaction of unmodified HEK293T cells. Also, the disease-mimicking mutation IVS10+16 c>t lead to the expected 4-fold increase as reported in the literature (doi: 10.1074/jbc.274.21.15134, doi:10.1016/j.molbrainres.2005.02.014).

In summary, the head-to-head comparison of a minigene system and the EXISERS for *MAPT* showed clearly aberrant splicing behavior for the minigene but not EXSISERS as

compared to unmodified cells. These findings are in line with several pieces of pertinent literature reviewed above.

R3P3:

2. The authors use CRISPR-Cas9 to integrate EXSISERS into areas of interest in the genome. When such knock-ins are performed and analyzed, typically researchers will generate multiple clonal cell lines, in case behavior in one cell line may be biased by unique Cas9-induced indel and/or template insertion off-target events. The authors should re-perform the experiments featured in Figures 3 and 4 (and associated supplemental figures) with at least one additional clonal cell line to demonstrate the generalizability of EXSISERS.

Response to R3P3:

We thank the Reviewer for this constructive criticism and agree that clonal lines may show different behavior in particular if SNPs, such as the *MAPT* IVS10+16 c>t mutation, are investigated. We have therefore included immunoblots to show that in all cases, homozygous c>t base transition in this regulatory intronic sequence led to an increase of the 4R/pan-tau inclusion-ratio in additional 9 clones (**Supplementary Fig. 6,7** in addition to the clonal line shown in **Fig. 2d**).

With respect to the experiments of **Fig. 3**, we validated the results regarding Cas13- or microRNA-mediated tau perturbation on unmodified HEK293T cells to exclude that the observed effects are artifacts on the post-translational level or by EXSISERS and performed an RNA-level quantification with RT-qPCR.
Using RT-qPCR, we confirmed that the extended 30 nt spacers are superior compared to the original 22 nt spacer in new **Supplementary Fig. 14a,** and the higher isoform specificity of targeting exon-junctions in new **Supplementary Fig. 14b**.

We also reproduced the minor effects of **Fig. 3f** in two independent $\text{EXSISERS}_{\text{MAPT:10NLuc-11FLuc}}$ clones (new **Supplementary Fig. 17**). In both clones, the combination of an exon 10 targeting crRNA together with a fusion of dRfxCas13d to the SR-rich domain of SC35 led to an increased 4R/pan-tau ratio. In contrast, the fusion to the Gly-rich domain of $\text{hnRNPA1}_{\text{A1B}}$ with a splice donor (SD) targeting crRNA decreased it (new **Supplementary Fig. 17**).

With respect to the experiments of **Fig. 4**, the results were already obtained from different clones. The lentiviral CRISPR/Cas9 KO library (**Fig. 4a and b**), as compared to the analyses in **Fig. 4c–f,** where an independent clone was used. We made this explicit into the caption of **Fig. 4.**

R3P4 (Minor P1):

Minor points:

1. The introduction would benefit from a reference to work on minigenes, as they are the main methodological competitor to EXSISERS.

Response to R3P4 (Minor P1):

We thank the Reviewer for this comment. We had already added references on minigenes in the main text in the introduction: *'Established methods for analyzing splicing isoforms either measure mRNA by endpoint-labeling (RT-qPCR, (sm)FISH[4], RNA-sequencing[5]), protein by immunochemistry (immunoblot analysis, immunofluorescence staining), or seek to mimic the genetic regulations via minigene analysis[6–8]'*

R3P5 (Minor P2):

2. The sentence should read "greater reduction": Expression of cytosolic PspCas13b-NES directed against the same region of exon 10 (Fig. 3e, orange bar) resulted in a greater reduction of FLuc as compared with the corresponding RfxCas13d-NLS (p<0.0001, post-hoc tests of one-way ANOVA) with comparable NLuc signal (p>0.05) (Fig. 3e, blue bar).

Response to R3P5 (Minor P2):

We thank the Reviewer for the suggestion, but indeed the knock-down (KD) of FLuc is 'less efficient' (leading to a 'weaker reduction' of FLuc) while NLuc depletion is as efficient as with Cas13d-NLS. We changed the whole sentence to: 'Expression of cytosolic PspCas13b-NES directed against the same region of exon 10 (**Fig. 3e, orange bar**) showed a better 4R-specificity due to decreased NLuc/FLuc-ratio compared with the corresponding RfxCas13d-NLS system (*p*<0.001, post-hoc tests of one-way ANOVA of $10/13d_{NLS}$ *vs.* $10/13b_{NES}$ *vs.* 9-10 amiRNA, **Fig. 3e, blue bar**).'

R3P6 (Minor P3):

3. The sentence should read "4f": Meanwhile, the enrichment of MBNL2 indels showed no dose-dependence (Fig. 4f).

Response to R3P6 (Minor P3):

We apologize for this mistake and corrected it.

Reviewer #4 (Remarks to the Author):

R4PA_B:

A. This work elegantly solves the current issues in quantifying protein expression levels by RNA-based approaches by incorporating a newly developed reporter system termed an exon-specific isoform expression reporter system (EXSISERS). The authors incorporated two EXSISERS into exons of interest (EOIs) by CRISPR/Cas9 and monitored the alternative splicing involved disease-associated exon inclusion of the patient-driven iPSC cells and screened RNA interference sequence for the isoform-specific expression to identify splice-regulators. Additionally, the authors similarly developed a survival reporter system for isoform-specific Blasticidin-S resistance marker. This article proposes the new exon-specific isoform expression reporter system would be a new tool for monitoring spatiotemporal exon-specific expression by imaging techniques.

B. This work is highly original and innovative with potential impacts in identifying splicing regulators and drug screening. Notably, this method could address the problems associated with protein expression level determined by RNA-based quantification methods. Thus, it is of significant importance and could be a game-changer for current RNA-based approaches if it is robust and reliable.

Response to R4PA_B:

We thank the Reviewer for acknowledging the advantages of EXSISERS' protein-level readout for drug screenings and basic research on identifying splicing regulators.

R4PC1:

C. In this system, there are several critical assumptions have not been controlled in this manuscript, which should be addressed in the manuscript before publications.

1. The manuscript is described as if protein trans-splicing has 100% efficiency (like Fig 2a, 2b). The splicing efficiency by protein trans-splicing is strongly affected by the junction sequence and the foreign exteins used. A single mutation near the junctions could abolish or deceased the splicing activity significantly, missing the controls to check the protein splicing efficiency.

Response to R4PC1:

We thank the Reviewer for this point regarding the efficiency of intein splicing.

In order to maximize efficiency, we chose fast-splicing inteins (doi:10.1038/s41467-020-15272-2, doi:10.1002/1873-3468.13909), which we further substantially enhanced with heterodimerization domains based on coiled-coils (CCs) (**Supplementary Fig. 1**). Recently, Bhagawati *et al.* (doi:10.1073/pnas.1909825116 showed in a similar approach, that intein splicing can be dramatically improved using a nanobody-antigen pair. By fusing an eGFP moiety to one half of a split-intein pair and an anti-GFP nanobody to the other split-intein counterpart, they could enable trans-splicing of a cysteine-free intein pair (important for extracellular protein splicing) that did not occur at all without the eGFP-nanobody interaction (please see their supplementary files Figure S10 vs. Figure S11).

These features enabled the very high splicing efficiency by immunoblot analysis of $\text{EXSISERS}_{\mathit{MAPT}:\text{10NLuc-11FLuc}}$ (**Supplementary Fig. 7**). Even when this construct was massively overexpressed via plasmid transfection, barely any unspliced proteins were detected (**Supplementary Fig. 2b**). Furthermore, the minigene version of this EXSISERS construct showed the same efficiency (**Supplementary Fig. 10c,d**).

As you requested in R4PF, the introduction of the terminal Asn→Ala mutation in the C-intein moiety completely disrupted protein splicing as expected (**Supplementary Fig. 2b**), thus indicating that the CCs-enhanced versions of the selected inteins are responsible for the exceptional high splicing efficiency.

With respect to considerations regarding the junction sequence, recent characterizations (doi:10.1002/1873-3468.13909) indicated that these 'ultrafast inteins' identified in metagenomic sources tolerate a broad spectrum of amino acids in heterologous settings very well (only proline is not tolerated heterologously and should be avoided). In conjunction with CCs, these efficiencies should increase even more.
In addition, we also now refer to the intein database in the method section under 'Application notes', which contains over 1000 inteins with known native extein sequences (maintained by the Iwaï lab, (InBase 2.0) https://inteins.biocenter.helsinki.fi/index.php), such that one can search for inteins with a desired native extein sequence to maximize the splicing efficiency.

R4PC2 and R4PC3:

2. Another assumption is similar to the previous one, FLuc and NLuc inserted in inteins fold into active equally with the same efficiency, yet having the same degradation rate in cells. The authors need to provide such experimental controls.
3. NLuc has 13-236 fold brighter than Fluc, according to the literature. All the data reported by normalized with the assumption, I believe.

Response to R4PC2 and R4PC3:

These assumptions do not have to be made. Instead, we measured the relative bioluminescence signal from *FLuc* and *NLuc* driven by a constitutive Pgk1 promoter at a 1:1 stoichiometry (**Supplementary Fig. 2a**). As seen in **Supplementary Fig. 2b**, the excision of NLuc/FLuc was very efficient. Moreover, we observed a linear relationship between the relative luminescence signals over 6 magnitudes and calculated NLuc yields 30 times more signal than FLuc (**Supplementary Fig. 2c**).

The Reviewer is also correct that for screening for modifiers of isoform expression, the bioluminescent signals were normalized to the control condition such that all relative differences between NLuc and FLuc are taken into account, and the effects of the perturbations can be directly seen. We have added additional notes in the figure legends and the manuscript to make the normalization procedure more explicit.

R4PC4:

4. The main caveat of this system easily overlooked by non-experts is the assumption that protein splicing by two split inteins has 100% or close to 100% efficiency. Particularly such high splicing activity for two orthogonal inteins has not been achieved in the past with an artificial system to my best knowledge. The reported efficiency of 95% in the cited ref.17 would result in the 90% efficiency for two orthogonal inteins. This assumption could determine the outcome of the analysis based on NLuc/FLuc quantification drastically.

Response to R4PC4:

As reported in subpoint R3PC1, we have used coiled-coil-enhanced fast-splicing inteins, and thus it is expected to have a greater efficiency than the reported value in the literature. We showed in **Supplementary Fig. 1** that CCs increased the protein splicing by nearly one

magnitude (8.6-fold), which is exceptional considering the folding speed of the challenging surrogate extein mNeonGreen with less than 10 minutes (doi:10.1038/nmeth.2413). We have also conducted detailed immunoblot analysis of the dual-luciferase EXSISERS$_{MAPT:10NLuc-11FLuc}$ upon plasmid-based overexpression (new **Supplementary Fig. 2b**), when genomically integrated (new **Supplementary Fig. 5b**, new **Supplementary Figure 6 and 7**), and as overexpressed minigene variant (new **Supplementary Fig. 10c,d and** new **Supplementary Fig. 12**), and detected no relevant levels of unspliced products even not upon heavy overexpression and overexposure.

R4PD:

D. NLuc usually has 13-236 >times brighter than FLuc according to the literature, which is consistent with the data presented with Figure 2e. The NLuc/Fluc error bars cannot be smaller than each of them. However, Figure 2j and all other data presented in Figure 3 do not make any sense, statistically.

The error estimation (P-value analysis) needs to be reconsidered. There are two types of errors mixed: (1) Errors from the detection (readout values) and (2) errors from individual samples or measurements. Even when the calculated error estimated from 3 samples is small, the accuracy of the measurement cannot be better than the precision of the detection errors.

Response to R4PD:

NLuc is indeed ~30-fold brighter compared to FLuc in the dual-luciferase EXSISERS. Please see **Supplementary Fig. 2c** for the calibration we performed to adjust for the relative differences in the bioluminescent signal obtained from the two luciferases when expressed at 1:1 stoichiometry. We adjusted for those relative differences in brightness in **Fig. 2 and 3** by normalizing the relative luminescence units (RLU) to the reference condition (with *MAPT* induction but without perturbation), such that the relevant effects of the perturbation of exon-specific isoform expression can be more readily read from the figures. This procedure is described in the Figure legends, the Material and Methods section, and the Statistics section.

Concerning the error calculation, the purpose of the dual-luciferase EXSISERS is to extract a robust, ratiometric measure of isoform-specific expression (NLuc) corrected for overall gene expression of tau (FLuc). The range of isoform-specific expression is thus naturally dependent on the overall expression. The FLuc and NLuc signals are also experimentally dependent on the cell lysis step in the Promega detection workflow that we employed (https://www.promega.de/-/media/files/resources/protocols/technical-manuals/101/nanoglo-dual-luciferase-reporter-assay-protocol.pdf): FLuc substrate is provided together with a lysis buffer onto the cells, followed by the first measurement (FLuc); in the 2nd step, NLuc substrate is provided together with a FLuc inhibitor, followed by the 2nd measurement (NLuc). Thus, for every FLuc RLU data point, there is a matching NLuc data point (paired measurement).

To reduce the biological variability from pan-tau expression and experimental variability stemming from the lysis and detection procedure, it thus makes sense to take the NLuc/FLuc ratio from each sample's cell population and calculate the average and errors over cell populations.

Calculating the errors of NLuc and FLuc separately over the biological triplicates would instead discard the information that the NLuc/FLuc pair was obtained from the same sample and thus defeat the purpose of absorbing the main source of variability.
Although the main conclusions are supported by statistical analyses directly on the NLuc/FLuc ratios, we still find it informative to also display the FLuc and NLuc signals separately, to, *e.g.*, show the effects of tau induction for reference or show the effects of an extended crRNA spacer on pan-tau expression.

We have explained this aspect of data processing in the figure legend and the Statistics section.
For completion, we also show all individual data point on top of the bar graph and provide a comprehensive table showing all raw data and detailed statistical results (**Supplementary Table 1**).

R4PE:

E.

As suggested in section C, D, and F, the validity of this system needs to be validated by additional controls. The authors should describe what would be potential pitfalls by the use of this reporter system. The current presentation does not provide sufficiently clear data to judge the validity and reliability of the system.

Response to R4PE:

We thank the Reviewer for the constructive suggestions of more data from control experiments to validate the experimental findings of the manuscript. We added RT-qPCR data (**Supplementary Fig. 14**) to confirm the key messages of **Fig. 3**. Furthermore, we added controls that the excision mechanism is indeed dependent on CCs-enhanced inteins by mutating the essential Asn of the C-inteins (C-gp41-1_{N37A} and C-NrdJ-1_{N40A}) (**Supplementary Fig. 2a,b**).
As requested, we have added paragraphs to the Materials and Methods section regarding the design criteria and potential pitfalls of EXSISERS constructs, the validation experiments to confirm efficient splicing of a given construct in analogy to our **Supplementary Figures 2,5,6,7, and 12**), a direct comparison to a minigene variant (**Supplementary Figure 10**), and detailed descriptions of how to generate clonal EXSISERS cell lines complementing **Supplementary Figures 3 and 4**.

R4PF:

F.

• There is no estimation of protein splicing efficiency for none of their protein splicing constructs except for mNG shown in Supplemental Fig. 1 by immunoblot. This data also does not give any estimate of the fully spliced vs by-products (non-spliced, N- and C-cleaved products). The supplemental Fig. 1 should be supplemented by immunoblotting and/or CBB-stained SDS-gels using, for example, anti-Ollas and Flag antibodies. The quantification by Nluc/Fluc ration will be strongly affected by the ligation efficiency, which is strongly dependent on the foreign extein and the splicing junctions.

Response to R4PF-part1

As requested, we updated **Supplementary Fig. 1.**, where we also now show an additional overexposed and contrast-enhanced image to detect all potential relevant side products. We

also added full immunoblots in the new **Supplementary Fig. 7, Supplementary Fig. 10c,d and Supplementary Fig. 12**.

Regarding **Supplemental Fig. 1**, we deliberately chose mNeonGreen as a model Extein with extremely fast folding rates (<<10 minutes, doi:10.1038/nmeth.2413) to define a maximally high benchmark for the intein-splicing speed. We have now added a densitometric quantification of the immunoblot in **Supplemental Fig. 1**, which shows that the addition of coiled-coils as heterodimerization domains improves the product/educt ratio by ~9 fold.
We have also added a deliberately overexposed immunoblot on which a small amount of side-products from C-cleavage can be detected that, however, amount to only ~3%.

In comparison to this test system, we have conducted detailed analyses of the protein splicing in the dual-luciferase reporter system for exon 10 inclusion of MAPT (EXSISERS$_{MAPT:10NLuc\text{-}11FLuc}$). Full immunoblots from multiple clones show essentially no unspliced products for tau **(Supplementary Fig. 7)**. Only under extreme overexposure, weak bands appear at densities of less than <1% of the spliced products, which probably correspond to the de novo translated proteins.
Even when the dual-luciferase reporter construct was heavily overexpressed at unphysiological levels from a plasmid (**Supplementary Figure 2b**) or as a minigene-version (**Supplementary Fig. 10d**), we could barely detect any unspliced educt.

• *What is the correlation between the quantification by immunoblotting (and/or mRNA quantification) vs NLuc/FLuc ratio for different constructs? Does it correlate well? if not, do they have a similar trend, which could be explained to some extent?*

Response to R4PF-part2
We performed additional experiments for the key messages of **Fig. 3** in HEK293T cells and quantified them via RT-qPCR. The observed effects and quantities were comparable between luciferase-based readout of EXSISERS$_{MAPT:10NLuc\text{-}11FLuc}$ cells and RT-qPCR of unmodified HEK293T cells (see **Supplementary 15** *vs.* **Fig. 3**).
Densitometric analysis of **Fig. 2d** also correlated well with the luciferase-based readouts (see new **Supplementary Fig. 5b** *vs.* **Fig. 2e**).

• *See also section D on the statistical data analysis.*

Response to R4PF-part3
Please see R4PD regarding the statistical analysis.

• *Fig.2d needs controls for protein-splicing deficient constructs by Ser-to-Ala and/or Asn-to-Ala.*

Response to R4PF-part4
We added **Supplementary Fig. 2**, where we expressed the cloned 0N4R cDNA of EXSISERS$_{MAPT:10NLuc\text{-}11FLuc}$ with intein-inactivating mutations in the C-intein moiety. The results show that active inteins are indispensable for the generation of the desired unmodified WT 0N4R tau band.

• *The authors claim "bio-orthogonal pair" of two inteins, but there is no such experimental evidence provided, including cited ref. 17. Trans-splicing is strongly dependent on the*

exteins, the authors could provide such data as a control, as this will affect the interpretation of the ratiometric data significantly. The orthogonality of two split intein should be demonstrated by using their systems because protein splicing by inteins is strongly extein-dependent.

Response to R4PF-part5

The inteins gp41-1 and NrdJ-1 have already been shown to be orthogonal by Pinto *et al.* (doi:10.1038/s41467-020-15272-2), which we cite in the main text.

We have not seen any mis-spliced products from these inteins, such as N-NrdJ-1- or C-gp41-1, which would have appeared as additional bands of lower molecular weight on the immunoblots (**Supplementary Fig. 2b,7, 10c, and 12**).

Moreover, the orthogonal pairs of coiled-coils, which likely dimerize already at the secondary structure level before any intein or extein segments can fold, add a second level of orthogonality.

• The author provided only one experimental data in Supplemental Fig 1 of immunoblotting and did not disclose any further sequence in detail. At least Supplemental Fig. 1 could be supplemented by covering all possible products using anti-Olla and Flag antibodies and provide the protein splicing efficiency quantitated for each of the two splicing steps. In theory, cleaved products might not interfere with NLuc/Fluc ratio. Do the authors have any evidence to assume that is the case?

Response to R4PF-part6

We updated **Supplementary Fig. 1** with an overexposed and contrast-enhanced immunoblot. We see a weak band for C-cleavage (~3%) using the fast-folding mNeonGreen as a surrogate extein sequence. Via densitometry, we could quantify that the addition of Coiled-Coils could enhance the protein splicing efficiency by ~9 fold. Please also see the full immunoblots in **Supplementary Fig. 7, 10c, and 12**, which show that the splicing efficiency was even higher for both inteins together with >99%.

• The main claims generally focus on the Ratio-metric assay using NLuc/Fluc, the survival system using BSD could be more confusing for readers than making it clear to understand the reporter system as currently written.

Response to R4PF-part7

We appreciate the Reviewer's suggestion but still find it valuable to showcase the versatility of the EXSISERS technology that goes beyond reporter signals. The capability to non-invasively couple the in- or exclusion of an exon to cell survival enables unbiased screenings for new splicing regulators, such as genome-wide CRISPR/Cas9-mediated KO screens. This powerful methodology was not possible before.

As an extension, one could also imagine to use dCas9-activator screens or instead use a triggerable toxin such as HSV-Tk, to screen for exon exclusion instead of inclusion.

R4PH:

H.

• The abstract is concise and clear.

• There are several misleading statements in the introduction, the authors claim "fast" protein splicing but no speed or relevant time scale is given. Protein splicing is strongly context-dependent, has to be investigated for each extein. This claim is thus not validated in

the manuscript. Moreover, there is no information about "trace-less" because the authors do not disclose the protein sequence for junction regions. "Traceless" should mean the spliced sequence is identical to the original protein sequence without a single mutation. Is this the case?

• The current data is not sufficiently supporting the conclusion because of several assumptions and lacks critical controls to verify each of the critical assumptions.

Response to R4PH:
We have now added a series of additional control experiments to further support that the very efficient intein splicing does not alter the physiological isoform expression and are thus scarless.

To initially investigate and optimize the splicing efficiency of the inteins, we created a construct using mNeonGreen as an extein with folding rates of <10 minutes (please see Supplementary Fig. 1). Even under these extreme conditions, our final design, including coiled-coils (CCs) achieved a significantly greater extein to intein-extein ratio, indicating higher protein splicing efficiency (~9-fold increase in efficiency, **Supplementary Fig. 1**).

Nevertheless, the Reviewer is, of course, right that our measurements did not include precise timing and therefore we have changed the term from 'fast' to 'efficient' in the abstract and the introduction. Still, we used the term 'fast' in the beginning of the results section when we refer to gp41-1 and NrdJ-1 inteins since the literature described them as ultrafast splicing inteins (doi:10.1073/pnas.1701083114, doi:10.1021/jacs.7b02618).

Application of EXSISERS on *MAPT* showed a very high protein splicing efficiency (**Supplementary Fig. 7, Supplementary Fig. 10c, and Supplementary Fig. 12**). Please also refer to the detailed answer to R4PF. With the 'classic inteins', such as Ssp or Npu DnaE, intein splicing is highly dependent on the extein sequences, but with those 'ultrafast inteins' identified in metagenomic sources, the literature (doi:10.1002/1873-3468.13909) showed that they tolerate heterologous settings very well (only proline is not tolerated by all inteins in a heterologous context).

Besides the recently discovered classes of fast and efficient inteins, we like to refer to the nicely maintained database from the Iwaï lab (formerly maintained by New England Biolabs), where one can screen for inteins where the native extein sequences are identical or similar to the desired insertion site. As an example, we used this database, to search for inteins suitable to split Cas9 between position 573 and 574 (KIE|CFD), *Npu* intein with the native extein sequence (AEY|CFN) which critical +2 position fits to the intended Cas9 split-site (doi:10.1093/nar/gkv601). Notably, we did not see any difference in activity between WT Cas9 and Npu intein split-Cas9.

We neither introduced any extra Ser/Cys/Thr, nor did we change any amino acid to Ser/Cys/Thr, but merely used the natively occurring Ser/Cys/Thr of an exon, therefore we consider it justified to use the term 'traceless' or 'scarless'. Please also see the Materials and Methods section 'Generation of stable cell lines with tagged exons via CRISPR/Cas9', where we described how we inserted EXSISERS into the GOI.

We also added additional experimental controls, such as RT-qPCR on unmodified HEK293T cells (**Supplementary Fig. 14**) data to substantiate our data from **Fig. 3** in

EXSISERS$_{MAPT:10NLuc-11FLuc}$ cells. We also added additional dual-luciferase assays data from other clones to exclude clone-dependent artifacts (**Supplementary Fig. 17**). Moreover, we included additional full-range immunoblots to show the high protein splicing efficiency of the CCs-improved inteins (**Supplementary Fig. 7, Supplementary Fig. 10c,d,, and Supplementary Fig. 12**).

Responses to Referees

Non-invasive and high-throughput interrogation of exon-specific isoform expression

Dong-Jiunn Jeffery Truong[1,2], Teeradon Phlairaharn[1,2], Bianca Eßwein[3], Christoph Gruber[3], Deniz Tümen[1], Enikő Baligács[1,2], Niklas Armbrust[1,2], Francesco Leandro Vaccaro[1,2], Eva-Maria Lederer[1,2], Eva Magdalena Beck[1,2], Julian Geilenkeuser[1,2], Simone Göppert[1,2], Luisa Krumwiede[1,2], Christian Grätz[1,2], Gerald Raffl[1,2], Dominic Schwarz[1,2], Martin Zirngibl[1,2], Milica Živanić[1,2], Maren Beyer[1,2], Johann Dietmar Körner[1,2], Tobias Santl[1,2], Valentin Evsyukov[4,5], Tabea Strauß[4,5], Sigrid C. Schwarz[4,5], Günter U. Höglinger[4,5,6], Peter Heutink[7,8], Sebastian Doll[3], Marcus Conrad[3], Florian Giesert[3], Wolfgang Wurst[3,5,6,9], Gil Gregor Westmeyer[1,2†]

[1]Institute for Synthetic Biomedicine, Helmholtz Zentrum München, 85764 Oberschleißheim, Germany
[2]Department of Chemistry and TUM School of Medicine, Technical University of Munich, 81675 Munich, Germany
[3]Institute of Developmental Genetics, Helmholtz Zentrum München, 85764 Oberschleißheim, Germany
[4]Department of Neurology, Technical University of Munich, 81377 Munich, Germany
[5]German Center for Neurodegenerative Diseases (DZNE), 81377 Munich, Germany
[6]Munich Cluster for Systems Neurology (SyNergy), Adolf-Butenandt-Institut, Ludwig-Maximilians-Universität München, 81377 Munich, Germany
[7]Department for Neurodegenerative Diseases, Hertie Institute for Clinical Brain Research, University of Tübingen, 72076 Tübingen, Germany
[8]German Center for Neurodegenerative Diseases (DZNE), 72076 Tübingen, Germany
[9]Chair of Developmental Genetics, TUM School of Life Sciences, Technical University of Munich, 85354 Freising, Germany.
[†]corresponding author

Note: Since the figures were substantially restructured and reordered for the final submission as per editorial request, we here
provide a lookup table specifying the mapping of previous to final figure indices.

**Figure indices for Responses to Referees**	**Figure indices of Final Submission**
Fig. 1	Fig. 1d,e partially moved to Fig. 2a
Fig. 2a,b	Fig. 1a,b
Fig. 2c	Supplementary Fig. 3a (left)
Fig. 2d	Fig. 1c
Fig. 2e	Fig. 1d
Fig. 2f	Fig. 2d
Fig. 2g BLI	Fig. 2b
Fig. 2g histogram	Fig. 2c
Fig. 2h	Fig. 2e
Fig. 2i	Fig. 3a
Fig. 2j	Fig. 3c
Fig. 2k	Fig. 3b
Fig. 3a	Fig. 4b
Fig. 3b	Fig. 4a
Fig. 3c	Fig. 4d
Fig. 3d	Fig. 4c, Fig. 5a
Fig. 3e	Fig. 5b
Fig. 3f	Fig. 5e
Fig. 3g	simplified to Fig. 5c,d
Fig. 4a	Fig. 6a
Fig. 4b	Fig. 6c
Fig. 4c	Fig. 6d
Fig. 4c	Fig. 6d
Fig. 4d	Fig. 6f
Fig. 4e	Fig. 6e
Fig. 4f	Extended Data Fig. 10
Supplementary Fig. 1	Extended Data Fig. 1
Supplementary Fig. 2a	Extended Data Fig. 2a
Supplementary Fig. 2b	Extended Data Fig. 2b
Supplementary Fig. 2c	Extended Data Fig. 2c
Supplementary Fig. 3	Supplementary Fig. 1
Supplementary Fig. 4	Supplementary Fig. 2
Supplementary Fig. 5a	Supplementary Fig. 3a (right)

Supplementary Fig. 5c	Supplementary Fig. 3c
Supplementary Fig. 6	Supplementary Fig. 4
Supplementary Fig. 7	Supplementary Fig. 5
Supplementary Fig. 8	Supplementary Fig. 6
Supplementary Fig. 9	Supplementary Fig. 7
Supplementary Fig. 9	Supplementary Fig. 7
Supplementary Fig. 10a	Extended Data Fig. 3a
Supplementary Fig. 10b	Extended Data Fig. 3b
Supplementary Fig. 10c	Extended Data Fig. 3c
Supplementary Fig. 10d	Extended Data Fig. 3d
Supplementary Fig. 10e	Extended Data Fig. 3e
Supplementary Fig. 11a	Supplementary Fig. 8a
Supplementary Fig. 11b	Supplementary Fig. 8b
Supplementary Fig. 12	Extended Data Fig. 4
Supplementary Fig. 13	Extended Data Fig. 5
Supplementary Fig. 14a	Extended Data Fig. 6a
Supplementary Fig. 14b	Extended Data Fig. 6b
Supplementary Fig. 14c	Extended Data Fig. 6c
Supplementary Fig. 15	Supplementary Fig. 9
Supplementary Fig. 16	Extended Data Fig. 7
Supplementary Fig. 17a	Supplementary Fig. 10a
Supplementary Fig. 17b	Supplementary Fig. 10b
Supplementary Fig. 17c	Supplementary Fig. 10c
Supplementary Fig. 18a	Extended Data Fig. 8a
Supplementary Fig. 18b	simplified to Extended Data Fig. 8b
Supplementary Fig. 18c	Extended Data Fig. 8d
Supplementary Fig. 18d	Extended Data Fig. 8e
Supplementary Fig. 18e	Extended Data Fig. 8c
Supplementary Fig. 18f	Extended Data Fig. 8f
Supplementary Fig. 18g	Extended Data Fig. 8g
Supplementary Fig. 18h (left)	Extended Data Fig. 8h
Supplementary Fig. 18h (right)	Extended Data Fig. 8i
Supplementary Fig. 18i	Extended Data Fig. 8j
Supplementary Fig. 18j	Extended Data Fig. 8k
Supplementary Fig. 18k	Extended Data Fig. 8l
Supplementary Fig. 18l	Extended Data Fig. 8n
Supplementary Fig. 18m	Extended Data Fig. 8m
Supplementary Fig. 19a	Supplementary Fig. 11a
Supplementary Fig. 19b	Supplementary Fig. 11b
Supplementary Fig. 20a	Extended Data Fig. 9a
Supplementary Fig. 20b	Extended Data Fig. 9b
Supplementary Fig. 20c	Extended Data Fig. 9c
Supplementary Fig. 20d	Extended Data Fig. 9d
Supplementary Fig. 21a	Fig. 7a
Supplementary Fig. 21b	Fig. 7b
Supplementary Fig. 21c	Fig. 7c
Supplementary Fig. 22	Supplementary Fig. 12
Supplementary Fig. 23a	Supplementary Fig. 13a
Supplementary Fig. 23b	Supplementary Fig. 13b
Supplementary Fig. 23c	Supplementary Fig. 13c
Supplementary Fig. 24	Supplementary Fig. 14
Supplementary Fig. 24	Supplementary Fig. 15

Table E1 | Lookup table to map previous to final figure indices.

Final Decision Letter:

Date: 1st April 21 12:38:53

Last Sent: 1st April 21 12:38:53

Triggered By: Christina Kary

From: christina.kary@us.nature.com

To: gil.westmeyer@tum.de

CC: ncb@springernature.com

BCC: rjsproduction@springernature.com;rjsart@springernature.com

Subject: Decision on Nature Cell Biology submission NCB-W40046E

Message: Dear Dr. Westmeyer,

I am writing on behalf of my colleague, Dr. Jie Wang, who is out of the office.

I am pleased to inform you that your manuscript, "Non-invasive and high-throughput interrogation of exon-specific isoform expression", has now been accepted for publication in Nature Cell Biology.

Thank you for sending us the final manuscript files to be processed for print and online production, and for returning the manuscript checklists and other forms. Your manuscript will now be passed to our production team who will be in contact with you if there are any questions with the production quality of supplied figures and text.

In approximately 10 business days you will receive an email with a link to choose the appropriate publishing options for your paper and our Author Services team will be in touch regarding any additional information that may be required.

You will not receive your proofs until the publishing agreement has been received through our system.

If you have any questions about our publishing options, costs, Open Access requirements, or our legal forms, please contact ASJournals@springernature.com

Before the manuscript is sent to our printers, we will make changes in the text that may be necessary either to make it conform with house style or to make it intelligible to our wide readership. We look particularly carefully at the titles of all

papers to ensure that indexing will be accurate and that they are not unreasonably long. We will ask your approval before the copy is finalized, and you will soon receive the edited proofs. Please check the text and figures carefully. Once your manuscript is typeset and you have completed the appropriate grant of rights, you will receive a link to your electronic proof via email with a request to make any corrections within 48 hours. If, when you receive your proof, you cannot meet this deadline, please inform us at rjsproduction@springernature.com immediately.

Once your paper has been scheduled for online publication, the Nature press office will be in touch to confirm the details. An online order form for reprints of your paper is available at https://www.nature.com/reprints/author-reprints.html. All co-authors, authors' institutions and authors' funding agencies can order reprints using the form appropriate to their geographical region.

Publication is conditional on the manuscript not being published elsewhere and on there being no announcement of this work to any media outlet until the online publication date in Nature Cell Biology.

Please note that *Nature Cell Biology* is a Transformative Journal (TJ). Authors may publish their research with us through the traditional subscription access route or make their paper immediately open access through payment of an article-processing charge (APC). Authors will not be required to make a final decision about access to their article until it has been accepted. Find out more about Transformative Journals

Authors may need to take specific actions to achieve compliance with funder and institutional open access mandates. For submissions from January 2021, if your research is supported by a funder that requires immediate open access (e.g. according to Plan S principles) then you should select the gold OA route, and we will direct you to the compliant route where possible. For authors selecting the subscription publication route our standard licensing terms will need to be accepted, including our self-archiving policies. Those standard licensing terms will supersede any other terms that the author or any third party may assert apply to any version of the manuscript.

To assist our authors in disseminating their research to the broader community, our SharedIt initiative provides you with a unique shareable link that will allow anyone (with or without a subscription) to read the published article. Recipients of the link with a subscription will also be able to download and print the PDF.

If your paper includes color figures, please be aware that in order to help cover some of the additional cost of four-color reproduction, Nature Research charges our authors a fee for the printing of their color figures. Please contact our offices for exact pricing and details.

As soon as your article is published, you will receive an automated email with your

shareable link.

If you have not already done so, we strongly recommend that you upload the step-by-step protocols used in this manuscript to the Protocol Exchange (www.nature.com/protocolexchange), an open online resource established by Nature Protocols that allows researchers to share their detailed experimental know-how. All uploaded protocols are made freely available, assigned DOIs for ease of citation and are fully searchable through nature.com. Protocols and the Nature and Nature research journal papers in which they are used can be linked to one another, and this link is clearly and prominently visible in the online versions of both papers. Authors who performed the specific experiments can act as primary authors for the Protocol as they will be best placed to share the methodology details, but the Corresponding Author of the present research paper should be included as one of the authors. By uploading your Protocols to Protocol Exchange, you are enabling researchers to more readily reproduce or adapt the methodology you use, as well as increasing the visibility of your protocols and papers. You can also establish a dedicated page to collect your lab Protocols. Further information can be found at www.nature.com/protocolexchange/about

You can use a single sign-on for all your accounts, view the status of all your manuscript submissions and reviews, access usage statistics for your published articles and download a record of your refereeing activity for the Nature journals.

Please feel free to contact us if you have any questions.

All the best,

Christina

--
Christina Kary, PhD
Chief Editor
Nature Cell Biology
1 New York Plaza
Tel: +44 (0) 207 843 4924
